# Patient Abuse, Neglect, and Exploitation: Why Physicians Need to Be Trauma-Informed

**DOI:** 10.15766/mep_2374-8265.11391

**Published:** 2024-04-23

**Authors:** Kathleen Franchek-Roa, Aarti Vala, Jennifer Goldman, Adam Dell, Angela P. Presson, Kaleb Eppich, Wendy L. Hobson

**Affiliations:** 1 Associate Professor, Department of Pediatrics, University of Utah School of Medicine; 2 Lead Physician, Pediatrics, Mission Neighborhood Health Center, San Francisco, CA; 3 Adjunct Associate Professor, Department of Pediatrics, University of Utah School of Medicine; 4 Research Professor, Division of Epidemiology, University of Utah School of Medicine; 5 Biostatistician, Department of Internal Medicine, University of Utah School of Medicine; 6 Professor, Department of Pediatrics, and Associate Vice President of Health Sciences Education, University of Utah School of Medicine

**Keywords:** Human Trafficking, Sexual Violence, Trauma-Informed Care, Vulnerable Adult Abuse, Child Abuse, Elder Abuse, Intimate Partner Violence, Social Determinants of Health, Diversity, Equity, Inclusion

## Abstract

**Introduction:**

Many people experience trauma, and its cumulative effects throughout the life span can alter health, development, and well-being. Despite this, few publications focusing on interpersonal trauma include a holistic understanding of the nature and widespread exposure of trauma experiences for patients. We developed an educational resource to teach residents about identifying and intervening with patients who experience trauma across the life span using a trauma-informed care (TIC) perspective.

**Methods:**

We created a 4-hour educational session for residents that included didactics, a virtual visit with a domestic violence shelter, a discussion with a person who had experienced trauma, and role-playing. A pretest/posttest retrospective survey assessed resident confidence level in identifying and intervening with patients who may have experienced trauma. We used the Wilcoxon signed rank test to compare pretest and posttest scores and the Kruskal-Wallis test to compare responses by residency type and year. Free-text questions were analyzed for thematic content.

**Results:**

During the 2021–2022 academic year, 72 of 90 residents (80%) from four residency programs attended and evaluated the session. More than 90% of respondents reported the session met their educational needs and provided them with new ideas, information, and practical suggestions to use in their clinical endeavors. The results demonstrated significantly increased confidence on most of the metrics measured.

**Discussion:**

This session significantly improved residents' confidence in identifying and intervening with patients who have had trauma experiences using a TIC perspective, which may lead them to provide improved patient care to those who have experienced trauma.

## Educational Objectives

By the end of the session, learners will be able to:

1. Explain the link between childhood adversity and risk for poor health across the life span.
2. Utilize a trauma-informed approach when interacting with patients to reduce or mitigate the consequences of these adverse experiences.
3. Discuss the prevalence of abuse, neglect, and/or exploitation in terms of the public health impact on health care.
4. Employ best practices when evaluating patients who are victims of abuse, neglect, and/or exploitation.

## Introduction

Trauma exposures are a common experience for many people. The Substance Abuse and Mental Health Services Administration (SAMHSA) defines individual trauma as

an event, series of events, or set of circumstances that is experienced by an individual as physically or emotionally harmful or life threatening and that has lasting adverse effects on the individual's functioning and mental, physical, social, emotional, or spiritual well-being.^1^

The key to understanding a patient's trauma experiences and providing appropriate support and interventions is embedded in SAMHSA's definition of trauma in that the experience and effect of trauma are unique to each individual.

The cumulative effects of traumatic experiences throughout the life span can alter health, development, and well-being,^2,3^ yet medicine has been slow to embrace the concept of trauma as a medical issue. Over the past 6 decades,^4–10^ the medical community has pieced together an understanding of the nature and widespread occurrence of trauma experiences for patients. However, the different forms of family and interpersonal trauma have been treated independently in terms of research, education, and policy development.^11^ This narrow focus has overlooked the interconnectedness of different forms of trauma, and as a result, the medical community has failed to understand the cumulative and profound negative impacts of trauma on lifelong health and the increased risk for future revictimization for those who experience polyvictimization in childhood.^2,12^ The 1998 Adverse Childhood Experiences Study^2^ and subsequent studies^13^ have added to our understanding of the impact of adversity during childhood on lifelong health outcomes. Given the pervasive effects of trauma, the trauma-informed care (TIC)^1^ approach has become an essential component in trauma curricula for health care providers.

Since research has shown that (1) trauma is common, (2) individual trauma experience often involves multiple types of victimization, and (3) trauma can adversely impact an individual's health and well-being across their life span, we developed an educational session at the University of Utah to educate residents in four residency programs (internal medicine, medicine-pediatrics, pediatrics, and family medicine) on the identification and intervention of patient abuse, neglect, and exploitation using a trauma-informed perspective. These residency programs acknowledged a need to integrate this comprehensive education into the training of their residents. Of the most relevant *MedEdPORTAL* publications addressing interpersonal trauma, only three^14–16^ include multiple forms of trauma. However, these publications do not feature all the components that our educational session incorporates, specifically, a trauma-informed component. In addition, unlike this resource, none model their educational sessions on an institutional policy that has helped to provide an integrative component to the education.

Our educational model is unique in that it integrates a more comprehensive understanding of trauma; is modeled on the University of Utah Health—Policy on Abuse, Neglect, and Exploitation, which is actively used as guidance for physicians and staff within our health care system; and involves community partners who aid in educating the residents.

## Methods

We developed a 4-hour educational session, held during the 2021–2022 academic year, for residents in four residency programs (internal medicine, medicine-pediatrics, pediatrics, and family medicine). Due to COVID-19 pandemic restrictions, the sessions were held in person on campus, without a visit to the domestic violence shelter.

The residency programs scheduled residents to attend during an outpatient or advocacy rotation, with a group size of two to eight residents to facilitate role-playing and group discussion. Residents received no incentive to participate or to complete the evaluation. Once scheduled, residents received articles to review prior to meeting (Appendix A). The in-person session included the following elements:

• Didactic lecture (60 minutes)
• Break (15 minutes)
• Virtual tour of the local domestic violence shelter (15 minutes)
• Discussion with a survivor of trauma (45 minutes)
• Break (15 minutes)
• Role-playing session and closure (90 minutes)

### Didactic Lecture

The session started with a 60-minute PowerPoint didactic component (Appendix B). Its first 32 slides covered Educational Objectives 1–3 and set the learners up for success in role-playing activities covering all four objectives. Slides 3–11 defined and discussed trauma, adverse childhood experiences, and other forms of toxic stressors and associated poor health across the life span. This discussion included the science of how extrinsic and intrinsic factors playing a role in brain development during childhood were important in laying the foundations for a child's cognitive, physical, and socioemotional developmental responses to stress.^17^ To enhance understanding of these concepts, there was broad discussion regarding how advancements in the science of epigenetics, neurodevelopment, and developmental psychology provided plausible biological pathways between early experiences and future development and health.^18^ Slides 12–18 emphasized the universal approach of treating all patients with a trauma-informed perspective. Examples illustrated the use of a TIC perspective and emphasized that the goal was not to obtain a disclosure but to provide trauma-informed medical care and resources. Slides 19–32 highlighted the public health impact of trauma experiences. Slides 34-125 were used for the role-playing exercise.

### Use of Community Partners in the Education of Residents

We partnered with a local shelter and family justice center (FJC) to provide contact with service providers and advocates. The FJC director gave a 15-minute virtual tour of the local shelter and FJC services via Zoom. Next, residents had a 45-minute interactive discussion, via Zoom, with a survivor of trauma who spoke of their experience with trauma and their interaction with health care systems during and after their trauma experiences. This gave the residents an opportunity to utilize the survivor's expertise in discussing how health care could improve the response to patients who have experienced trauma. It also provided the residents with a patient perspective on health care, especially that of a patient who had experienced trauma.

### Faculty and Survivor Advocate Preparation

We recommend that faculty who wish to use this educational session be trained in trauma-informed practices, familiarize themselves with student/resident support systems in their institution, be trained in responding to a disclosure of trauma from a student/resident, and have access to faculty who can provide mental health services if needed by the learners. Author Kathleen Franchek-Roa was the chair of the University of Utah Health Domestic Violence Committee, which had written the policy for health care providers regarding interventions for persons experiencing abuse, neglect, and exploitation. The residents were made aware of the policy and available resources. Importantly, the survivor advocates had experience educating medical providers and others about their experiences and volunteered to participate in this resident training.

### Role-Playing Session

Role-playing has been shown to improve communication skills^19^ and increase self-efficacy^20^ of learners. Role-playing as an educational method is regarded as an effective form of simulation, due in part to the social^21^ and experiential^22^ context in which learning takes place. Additionally, when students role-play as patients, they may garner a more empathetic perspective towards patients' concerns.^21^ Therefore, we added a role-play component to the session to enhance learning and skill development.

The role-playing session adhered to Kolb and Fry's experiential learning model^22^ by (1) providing a concrete experience for the residents in practicing how to respond to a trauma disclosure, (2) involving the residents who were not role-playing as observers to enrich the discussion, and (3) creating generalizable concepts by dissecting the case into its component parts. The fourth component, testing implications in new situations, would occur subsequently as the residents applied the skills and knowledge gained in the session in direct patient care in the clinic or hospital setting.

We created seven role-playing scenarios: an intimate partner violence/abuse case, a child abuse case, pediatric and adult human trafficking cases, vulnerable adult abuse cases (both elder and nonelder), and a sexual assault case (Appendix C). Two to three role-playing scenarios were utilized during each 90-minute session. Scenarios were selected according to which residency programs were participating. The role-playing scripts had a definite beginning and end to simulate how these narratives could play out in a real case scenario and gave the residents a chance to use the SMART (screen vs. ask, message, assess symptoms and danger, resources and report, treat) tool (Appendix D) to help guide the role-play to its conclusion. The debriefing portion adhered to principles of debriefing,^23^ including establishing group norms, giving participants and observers an opportunity to reflect on the scenario, applying knowledge previously learned (Appendix B) to the role-playing scenarios, and allowing participants to discuss the value of this experience either verbally during the discussion or by providing written anonymous comments via the evaluation survey after the session.

The role-playing was guided by Appendix B's PowerPoint slides 34–125, which provided our state-specific reporting requirements, the SMART tool (Appendix D), and the discussion of the cases. Authors Kathleen Franchek-Roa and Aarti Vala developed the SMART tool to help learners navigate these difficult patient encounters. We designed the SMART tool more as a retrieval aid than as a core-learning strategy.^24^

Relevant principles adhered to in providing a psychologically safe learning environment^25^ and enhancing communication during the role-playing session^21^ included providing prework articles and objectives prior to the educational session; offering the didactic portion of the session before role-playing to provide background knowledge; utilizing the SMART tool (Appendix D) to aid residents in navigating difficult conversations; promoting empathy towards patients and each other, which could lead to enhancing the humanistic response (e.g., by emphasizing the understanding that high-risk health behaviors might be a coping strategy for patients who have experienced trauma,^2^ by having residents play the patient role,^21^ and by incorporating the understanding of the holistic approach to medicine); encouraging the residents to be comfortable in their uncertainty^26^ because many of these patient encounters might not lead to a satisfactory conclusion; and drawing on SAMHSA's trauma-informed principles^1^ in creating a safe space for learners. These concepts helped to enhance group norms among the learners as they shared these experiences. These educational concepts added an important dimension to what it meant to be a doctor.^25,27^ Faculty were available for residents if further discussion was needed. In addition, including a small-group role-playing session aided in providing peer support for the residents. Although most of the learners were interns, numerous sessions included second- and third-year residents, who provided the interns with constructive suggestions informed by their own interactions with patients who had experienced trauma.

### Postsession Materials

After the session, residents received a link to the electronic evaluation survey (Appendix E) and additional materials (Appendix F).

### Evaluation

The evaluation survey (Appendix E) measured whether the session met residents' educational needs and provided them with new ideas/information and practical suggestions to use in their clinical endeavors (choices were yes, somewhat, and no). The retrospective pretest/posttest evaluated residents' confidence in their ability to meet the objectives (choices were no confidence, low confidence, moderate confidence, and high confidence). The retrospective pretest/posttest had learners rate their level of baseline ability (e.g., knowledge, attitudes, behaviors) simultaneously with their posteducational intervention ability. Howard and colleagues^28^ undertook a rigorous evaluation of the traditional versus retrospective pretest/posttest design and found that the retrospective pretest/posttest design provided a measure of self-reported change more in line with objective changes observed. Our evaluation survey also included demographic questions and two free-text questions about behavioral change and ways to improve the session. The evaluation survey was implemented electronically and anonymously collected through REDCap, a self-service application subsidized for University of Utah research by the Clinical Translational Science Institute. Residents were free to choose whether to fill out the survey or not.

We summarized demographics and residency information using median and interquartile range for age as a continuous variable and counts and percentages for gender, residency type, and residency year. Likert plots were constructed for the survey questions both before and after the training using a retrospective pretest/posttest design. The pretest and posttest survey responses were summarized using percentages (in the Likert plots), means, and standard deviations and compared using Wilcoxon signed rank tests. The difference between the pretest and posttest survey responses was calculated for each survey question, and the Kruskal-Wallis test was used to compare the difference in responses by residency type and year. Year of residency was divided into two groups (first-year residents and second-/third-/fourth-year residents) to determine if year of residency, as a proxy for experience, impacted the change in resident confidence in the parameters measured. We also analyzed the free-text questions for thematic content. Statistical significance was assessed at the *p* < .05 level, and all tests were two-tailed. Statistical analyses were performed using R version 4.2.1 (R Foundation).

## Results

Ninety residents out of 106 eligible participated in the educational sessions during the 2021–2022 academic year, and 72 of the 90 (80%) completed the evaluation. The median age was 27 years, 48% of the respondents were female, 49% were internal medicine residents, and most (68%) were in their first year of residency (Table 1).

**Table 1.**
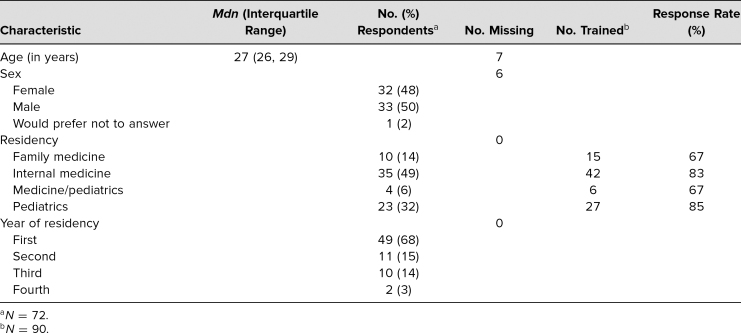
Survey Demographics

The overwhelming majority of respondents (≥90%) felt that the session met their educational needs, provided them with new ideas and information, and gave them practical suggestions that they could use in their clinical endeavors.

Table 2 shows that scores improved significantly for all items (*p* < .001). Importantly, there was no difference in score improvement (posttest minus pretest) between residency type and residency year except for one item. Score improvement for “Employ best practices when evaluating patients who are victims of abuse, neglect, and/or exploitation” was significantly higher for first-year residents relative to the combined group of second-/third-/fourth-year residents (*p* = .02).

**Table 2.**
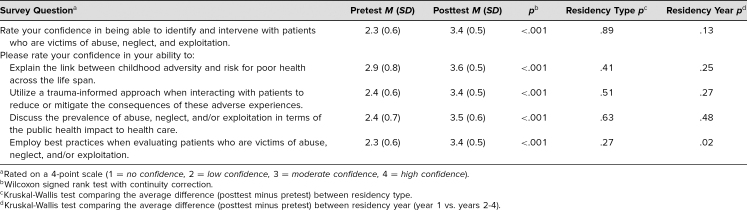
Retrospective Pretest/Posttest Survey Responses and Average Difference for Residency Type and Year (*N* = 72)

The Figure shows Likert plots for each question illustrating the differences between the retrospective pretest/posttest responses. For example, the statement “Rate your confidence in being able to identify and intervene with patients who are victims of abuse, neglect, and exploitation” shows that 31% rated their pretraining confidence as moderate or high as opposed to 100% rating their posttraining confidence as moderate or high. The remaining statements can be interpreted similarly.

**Figure.**
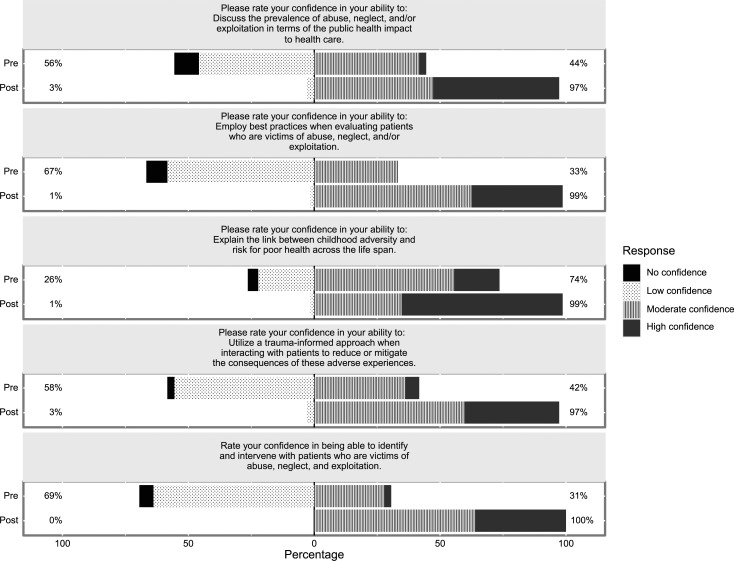
Likert plots for each survey question.

The thematic content for the question “What changes will you incorporate into your future clinical endeavors as a result of the knowledge acquired during this activity?” is represented in Table 3. The most common themes included recognizing the signs and symptoms of potential abuse (42%), screening for intimate partner violence as per the US Preventive Services Task Force recommendation^29^ (35%), and using the General Resource List (27%). The two most common themes in response to the question “How can we improve the session?” were “no improvement needed” (48%) and “more role-playing” (14%).

**Table 3.**
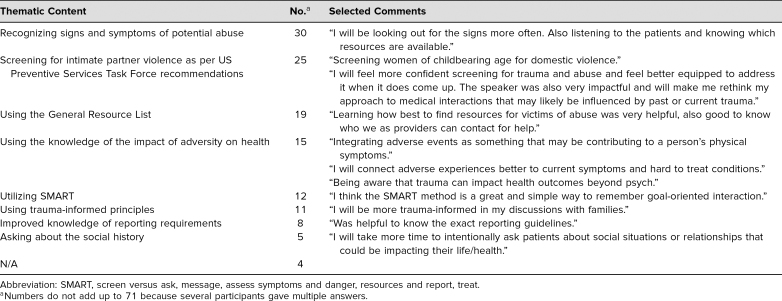
Thematic Content for the Reflective Question “What Changes Will You Incorporate Into Your Future Clinical Endeavors as a Result of the Knowledge Acquired During This Activity?”(*N* = 71; Single Responses Not Shown)

## Discussion

As the significance of the impact of trauma experiences on health has weaved its way into the health care arena, there is a need to develop medical education that improves future health care professionals' ability to address patients' trauma experiences and their impact. Many trauma-related medical education publications focus on only one form of interpersonal trauma. Since many people experience multiple types of trauma across their life span, offering this education as a holistic session, as our project does, is crucial in providing appropriate and effective care to patients.

The finding that participants' confidence was significantly improved after the educational session indicates that the session was effective in improving residents' confidence in their ability to care for patients who have experienced trauma. In addition, finding that only one-third of participants felt confident in their ability to intervene with patients who have experienced trauma before the session while all (100%) felt confident in this ability after the session suggests that residents would benefit from structured educational sessions on how to care for patients who have experienced trauma.

We undertook an analysis that included comparing residency type and year with confidence improvement to determine if different residency programs and year in residency (as a proxy for experience) influenced the results. We found that the improvement in confidence in caring for patients who have experienced trauma did not differ between residency types, suggesting that this session can be taught to multiple groups of residents. Regarding year in residency, we found that the improvement in confidence for four of the five survey questions did not differ between the first-year group and the combined second-/third-/fourth-year group. This may suggest that structured educational interventions, including skill acquisition through role-playing scenarios, are effective across all experience levels.

Our resource is unique because it provides a broadened approach to interpersonal trauma by incorporating trauma experiences across the life span, illustrating the interconnectedness of different trauma experiences for patients, including the TIC perspective, and being based on a policy at our institution. This comprehensive understanding of patients' life experiences aids in developing effective interventions. With intimate partner violence at least, developing an integrated systems-based approach has been shown to be crucial to providing the care and resources that patients exposed to this type of trauma need.^30^ It is reasonable to extrapolate that integrating a systemwide approach would also benefit patients experiencing other forms of trauma.

This session included educational methods derived from adult learning principles,^22,31^ such as making the content relevant to the work residents are engaged in, utilizing multiple educational techniques that support different learning styles, providing experiential opportunities to practice skills in a safe environment, offering faculty and peer feedback during the learning session, and providing content that learners can immediately use when they interact with patients (e.g., SMART tool). Skills taught can serve these residents in many inpatient and outpatient experiences. Although our session focused on interpersonal trauma, discussions after the role-playing scenarios expanded this concept of trauma to include other forms of stressors that patients and their families experience, such as social determinants of health, as well as trauma-informed principles that should be integrated into all patient encounters.

The weekly availability of sessions enabled us to engage with multiple residency programs. Allowing each of the residency programs to carve out a time that worked for its residents was key in being able to provide this education to a broad swath of primary care medical specialties within our medical school.

We have demonstrated the short-term effectiveness of this resource. The thematic content qualitatively showed that residents had begun to internalize what they had learned and were already envisioning ways to incorporate it into their practice. Over a quarter of the residents (27%) specifically mentioned the utility of a resource list when working with patients who have experienced trauma, and one-third (36%) indicated that they would be screening for intimate partner violence in women of reproductive age. This is a critical first step in providing care to patients.

Of the topics covered in this resource, the only screening recommendation from the US Preventive Services Task Force is for intimate partner violence in women of reproductive age.^29^ The lack of recommendations for screening for violence is emphasized in our session (and our institutional policy) by recommending that health care providers screen for intimate partner violence in women of reproductive age but also ask any patient with signs or symptoms suggestive of trauma. This is an important distinction because asking about trauma experiences in patients who have symptoms suggestive of trauma can yield a more accurate diagnosis, thereby avoiding misdiagnoses that can lead to mismanagement of symptoms.^32,33^ Recognizing trauma symptoms is critical in identifying patients who would benefit from evidence-based, trauma-specific treatments.^34,35^

Although our session was effective in increasing residents' confidence in their ability to identify and intervene with patients who have experienced trauma, there are several limitations. We implemented the session at only one institution, with four residency programs. Results may not be the same with other types of learners or at other institutions. We documented short-term improvement in the indices measured, but we do not yet have data to show that the improvements in knowledge and behavior were sustained. Our evaluation relied on self-reported, not observed, change, although using the retrospective pretest/posttest evaluation led to a more accurate assessment of change.^28^ In addition, it might be of value in the future to provide a multiple-choice survey of learners' understanding of these concepts, which could delineate areas of misunderstanding and lack of knowledge and thus enhance the educational sessions. Using trauma-informed principles is critical to providing a safe learning environment, and utilizing near-peer facilitators who have previously participated in the training might provide additional benefit to learners, especially those in their intern year. We learned valuable lessons by allowing the residents to provide anonymous comments via the evaluation survey.^23^ For example, resident comments included how to improve using the expert's lived experience to maximize the learning benefit; how having the session at the shelter, once COVID restrictions are eased, might be more impactful; and how, since the role-playing enhanced learning, more time should be allotted to this portion of the educational session.

Due to COVID-19 restrictions, we had a virtual visit with the domestic violence shelter. We surmise that holding sessions at a domestic violence shelter could enhance residents' experience. Engaging community partners in the education of residents strengthens relationships between health care and community resources, which is vital in providing support to patients. The discussion with the experts enriched the session by providing a relationship, albeit brief, between the residents and a survivor, which is difficult to achieve if not in real time or in person. It helped to put a face to these difficult issues.

Implementing this educational model is labor intensive, requiring dedicated faculty to provide numerous sessions so as to involve residents from different residency programs and rotations. The sessions are difficult to run with larger groups of participants because having fewer participants per session allows for more experiential opportunities, especially regarding the role-playing. Finally, a 4-hour session can be tiresome for participants and difficult to fit into a busy residency schedule. The session could be broken into smaller sessions, by trauma or activity, while still preserving the holistic approach to patient trauma experiences.

Our educational model was successful, in the short term, in educating residents to respond to patients who have experienced trauma. More research is needed to inform effective prevention and intervention efforts for patients with trauma experiences and to provide guidance on how to implement this into medical education. Training the next generation of health care providers in understanding how to identify and support patients suffering the lifelong effects of trauma, with a TIC perspective, is the first step towards ensuring a healthier future for everyone and is a pressing need in medical education.

## Appendices


Prework Articles.docxDidactic.pptxRole-Playing Facilitator Guide.docxSMART Tool.docxPretest-Posttest Survey.docxPostsession Materials.docx
*All appendices are peer reviewed as integral parts of the Original Publication.*
