# Supplementary material for: Patient Abuse, Neglect, and Exploitation: Why Physicians Need to Be Trauma-Informed
Source: MedEdPORTAL. 2024 Apr 23;20:11391. doi: 10.15766/mep_2374-8265.11391 (PMC11035495; doi:10.15766/mep_2374-8265.11391)
Supplement: Supplementary file 1 — Prework Articles.docxDidactic.pptxRole-Playing Facilitator Guide.docxSMART Tool.docxPretest-Posttest Survey.docxPostsession Materials.docx [file mep_2374-8265.11391-s001.zip › C. Role-Playing Facilitator Guide.docx]

Role-Playing Sessions Instructions for Presenter

***Note to*** *Facilitator****:*** Each of the interpersonal trauma types are linked to the role-play script. You can choose the role-playing scenarios that are appropriate for your learners.

The role-playing sessions are meant to enhance learning in a safe space. The residents are encouraged to use the SMART tool and to stop the role-playing to ask questions or get clarity on what they should do next.

Allow the residents to volunteer for the ‘physician’ and ‘patient’ role. If someone does not feel comfortable doing the role-play, then the facilitator can take that part. For each session, the ‘physician' role is shown to all the participants and the ‘patient’ role script is given to the volunteer who is playing the ‘patient’ role. The scripts are provided in the facilitator guide (Appendix C). Let the resident playing the ‘patient’ role know that they can *ad lib* as needed.

The role-playing scenarios are in a gender-neutral voice.

After the role-playing session, the following slides help to frame the discussion.

**Child Abuse Role-Play 1—Facilitator**

**Purpose**

The participant practices the skills learned to inquire about trauma exposure based on presenting symptoms in a pediatric patient.

**Physician Role**

- You are a resident in your Longitudinal Outpatient Experience Clinic.
- The parent of an 8-year-old boy named Elias is here for an appointment because the parent and the teacher are concerned that he has ADHD.
- **Role-play session begins when you walk into the room with the knowledge of the chief complaint.**
- Utilize **SMART** to interview the parent (**S**ymptoms, **M**essage, **A**ssess, **R**esources/**R**eport, **T**reat)

***Note to*** *Facilitator****:*** *The script for the ‘parent role’ is given to the resident who is playing the parent. Give the resident time to read their script and encourage the resident to ad lib as needed. Only the resident playing this role should see the script.*

**Parent Role**

- You are the parent of an 8-year-old boy named Elias.
- You are bringing him to the appointment today because you and his teacher are concerned that he has ADHD.
- **Role-play session begins when the physician enters the room to start the visit.**
- If the physician asks why you think he has ADHD or what are his symptoms you say,

***“He is doing poorly in school, does not finish his homework, seems to have difficulty concentrating, and always seems to be getting into trouble at school. He has always had problems in school, but things seem to be getting worse.”***

- If the physician asks the Traumatic Stress Questions, you hesitate and say,

***“No, nothing like that has ever happened to Elias.”***

- If the physician asks you why you think Elias’ behavior is getting worse or has anything changed recently say,

**“*My partner recently got released from prison and is back in our lives.”***

- If the physician asks you about your relationship with your partner say,

***“Oh, we have our ups and downs. We do argue a lot especially about drug use.***

***Lately, it seems our arguments are getting a little more violent, but Elias has***

***never seen this.”***

- If the physician probes more about the violence say**,**

***“Why are you asking me these questions? I came here to get medication for Elias’ ADHD.”***

- **Role-play session ends when physician talks to you about the adversity that Elias has experienced and explains how his behavior and symptoms are consistent with traumatic stress and/or talks to you about having to report this to CPS.**

**Post Role-Playing Discussion Questions**

- How did the scenario feel as the provider?
- How did the scenario feel as the patient’s parent?
- Provide time for the Observers to comment.
- Did the physician utilize the SMART mnemonic effectively?
- What tools did the participants find useful in this scenario (e.g., algorithms**, SMART)?**
- Did Elias have any adverse childhood experiences (ACEs)?

*Parent is a victim of domestic violence, caregiver incarcerated, caregiver substance use*

- Does this case have to be reported to CPS or law enforcement?

*(Refer to your state-specific mandatory reporting requirements for healthcare providers.)*

*In Utah, Commission of Domestic Violence in the Presence of a Child is considered child abuse. “In the presence of a child” means: in the physical presence of a child; or having knowledge that a child is present and may see or hear an act of domestic violence.”*

- Discuss the utility of providing Elias’ parent and teacher ADHD assessment scales in this case—specifically, what the assessment scales can and cannot help to diagnose.

**Notes on Attention Deficit Hyperactivity Disorder (ADHD)**

- ADHD is a disorder that begins in childhood with symptoms of hyperactivity, impulsivity and/or attention problems.^1^
- For a detailed description of the differential diagnosis for ADHD see UpToDate. ^2^
- National Center for Traumatic Stress^3^
  - “A number of researchers believe that symptoms of child traumatic stress could be mistaken for ADHD and that the risk of misdiagnosis is high. This is because there is an overlap between ADHD symptoms and the effects of experiencing trauma. Unless symptoms are examined closely, the profiles of child traumatic stress and ADHD can appear to be similar.
  - “For example: Young children who experience trauma may have symptoms of hyperactivity and disruptive behavior that resemble ADHD. Trauma can make children feel agitated, troubled, nervous, and on alert. These behaviors can be mistaken for hyperactivity. What might seem like inattention in children who experience trauma might actually be symptoms of dissociation (feelings of unreality or being outside of one’s body) or the result of avoidance of trauma reminders. Among children who experience trauma, intrusive thoughts or memories of trauma (e.g., feeling like it is happening all over again) may lead to confused or agitated behavior which can resemble the impulsivity of ADHD. Overlapping symptoms can make it difficult to obtain a correct diagnosis, which can complicate both assessment and treatment. This is especially true when little or nothing is known about the onset of symptoms.”

1 American Psychiatric Association. Attention-deficit/hyperactivity disorder. In: Diagnostic and Statistical Manual of Mental Disorders, Fifth Edition, American Psychiatric Association, Arlington, VA 2013. p.59.

2 Krull KR. Attention deficit hyperactivity disorder in children and adolescents: Clinical features and diagnosis. UpToDate. Literature review current through: May 2020. | This topic last updated: Nov 27, 2019.

3 Siegfried CB, Blackshear K, National Child Traumatic Stress Network, with assistance from the National Resource Center on ADHD: A Program of Children and Adults with Attention Deficit/Hyperactivity Disorder (CHADD). Is it ADHD or child traumatic stress? A guide for Clinicians. Los Angeles, CA & Durham, NC: National Center for Child Traumatic Stress. 2016.

**Intimate Partner Violence Role-Play 2—Facilitator**

**Purpose**

The participant practices the skills learned to screen/ask a patient about intimate partner violence.

**Physician Role**

- You are a resident in your Longitudinal Outpatient Experience Clinic.
- You are seeing a 30-year-old new patient who is here today to establish care.
- Social History includes that the patient is married and has a 2-year-old child. The patient works full-time as a CPA. Patient’s partner works full-time as an engineer.
- Past Medical History includes chronic headaches, poorly controlled asthma, and insomnia.
- **Role-play session begins now after you obtained the medical history as outlined above.**
- Utilize **SMART** to interview patient (**S**creen, **M**essage, **A**ssess, **R**esources/**R**eport, **T**reat)

***Note to*** *Facilitator****:*** *The script for the ‘patient role’ is given to the resident who is playing the patient. Give the resident time to read their script and encourage the resident to ad lib as needed. Only the resident playing this role should see the script.*

**Patient Role**

- You are a 30-year-old who is coming to see a new doctor today to establish care.
- You have been married for 5 years and have a 2-year-old child. You work full-time as a CPA and your partner works full-time as an engineer.
- Your main reasons for seeing a new doctor are that you have been suffering from chronic headache, poorly controlled asthma, and insomnia for years and all your previous doctors have not been able to help you feel better.
- You act a little irritated because you do not trust that any doctor can help you because none has; however, you are seeing this doctor because your headaches and asthma are getting worse, and it is making it difficult to function at work and home.
- **Role-play session begins after the physician has greeted you and obtains your chief complaints as documented above.**
- If the physician asks you about your relationship with your partner or screens you for Intimate Partner Violence you say,

***“Well, we do seem to argue a lot.”***

- If the physician follows up on your initial response of ‘arguing a lot’ you say,

***“The arguments seem to be getting more frequent. My partner has started yelling at me more, is constantly belittling me and making me feel bad about myself and has threatened to divorce me because I’m sick all the time.”***

- If the physician asks specifically about violence towards you, you say,

***“Oh, no. My partner has never hit me. I saw my dad hit my mom and our marriage is not that bad.”***

- **Role-play session ends when the physician offers you resources and discusses how unhealthy relationships can contribute to chronic health problems.**

**Post Role-Playing Discussion Questions**

- How did the scenario feel as the provider?
- How did the scenario feel as the patient?
- Provide time for the Observers to comment.
- Did the ‘physician’ utilize the SMART mnemonic effectively?
- What tools did the participants find useful in this scenario (e.g., algorithms, resource list, SMART)?
- What were some of the ‘red flags’ or symptoms in this case scenario?

*Chronic illness, difficulty managing chronic illness, history of exposure to ACEs*

- Why do you think patients are generally not forthcoming when initially asked about their relationships?

*They do not see themselves as a ‘victim,’ they are minimizing the abuse, they grew up seeing intimate partner violence between their parents and thought of it as the way relationships are, they are not making the connection between unhealthy relationships and poor health.*

- Does this case have to be reported to law enforcement?
- *(Refer to your state-specific mandatory reporting requirements for healthcare providers.) For Utah, It does not have to be reported. However, this is still an unhealthy relationship.*
- Utilize the Power and Control Wheel to explain the tactics used to keep victims in unhealthy relationships. (see <https://www.theduluthmodel.org/wheels/>)
- Discuss why victims stay in unhealthy relationships.

*Fear, financial stability, share children, built a life together, hope for change, limited resources, was not believed when disclosed to others, neurobiology of trauma, trauma-coerced attachment, previous poor response from systems designed to protect women and children*

- Explain the definition of Trauma-Coerced Attachment (in the past was referred to as Stockholm Syndrome or Trauma Bonding) and discuss how this might apply with some intimate partner violence relationships.

**Trauma-Coerced Attachment (TCA)^1,2^**

- Trauma-Coerced Attachment is a physiologic and psychological adaptation to situations of extreme danger and terror that help the victim cope with the violence to increase their safety and decrease their pain.
- It is marked by a powerful emotional attachment to the abusive partner.
- TCA is not a static condition; it remains dynamic and changing over the course of a relationship.
- A key feature of TCA is the immense dependency on an abuser whose behavior injures or destroys, rather than nurtures, the integrity of the victim.
- The seemingly contradictory, but positive, feelings of love, gratitude, or loyalty to the abuser may cause a victim to protect or defend the abuser when the relationship is threatened.
- The understanding of TCA is still incomplete, but it provides a framework to understand people’s behaviors in unhealthy, abusive relationships which may enhance their survival in their current situation. [See Slide 8: Neurobiology of Trauma: *“An abnormal reaction to an abnormal situation is normal behavior.”* (Victor Frankl, 1946^3^)]

^1^Cantor C, Price, J. (2007). Traumatic entrapment, appeasement and complex post-traumatic stress disorder: Evolutionary perspectives of hostage reactions, domestic abuse and the Stockholm syndrome. The Australian and New Zealand Journal of Psychiatry. 2007;41:377–384.

^2^Doychak K, Raghavan C. “No voice or vote:” trauma-coerced attachment in victims of sex trafficking. J Human Trafficking. 2018; DOI: 10.1080/23322705.2018.1518625

^3^Frankl VE. Man’s Search for Meaning. Beacon Press, Boston 1992. Originally published in 1946.

**Elder Abuse Role-Play 3—Facilitator**

**Purpose**

The participant practices the skills learned to inquire about abuse/neglect exposure based on presenting symptoms in a vulnerable adult (elder) patient.

**Physician Role**

- You are a resident in your Longitudinal Outpatient Experience Clinic.
- Mr. Grey, a 72-year-old male, comes in for a follow-up visit for his chronic conditions. His adult child brings him to the visit**.**
- Social History includes Mr. Grey lost his wife 2 years ago. They had been married for 45 years. He has been living alone since his wife passed away.
- Past Medical History includes renal failure, chronic hypertension, and coronary artery disease.
- Mr. Grey’s adult child tells you that their father is out of his blood pressure medication and has a sore on his heel making it difficult for him to walk.
- Mr. Grey has missed his last 2 appointments.
- Vital signs today include Temperature of 99, HR 112, RR 25, BP 168/103. He has lost 3.5 kg (8lb) since his last visit 6 months ago.
- In addition, you notice that Mr. Grey is wearing dirty clothes, his shirt is missing several buttons, his hair is greasy and unkempt.
- You start the visit with Mr. Grey alone; however, he is unable to answer most of your questions and tells you to ask his adult child.
- **Role-play session begins when you call Mr. Grey’s adult child back into the room to discuss Mr. Grey’s medical care.**
- Utilize **SMART** to interview Mr. Grey (**S**ymptoms, **M**essage, **A**ssess, **R**esources/**R**eport, **T**reat)

***Note to*** *Facilitator****:*** *The script for the ‘patient’s adult child role’ is given to the resident who is playing this part. Give the resident time to read their script and encourage the resident to ad lib as needed. Only the resident playing this role should see the script.*

**Patient’s Adult Child Role**

- You are the 50-year-old adult child of Mr. Grey.
- You have been trying to care for your father since your mother passed away 2 years ago.
- You have tried to get your father to move to an assisted-living center, but he refuses.
- The physician initially asked you to wait in the waiting room during the history-taking and exam; however, the physician calls you back into the room because your father is unable to answer most of the physician’s questions, so the physician needs you to help with the history.
- **Role-play session begins when you are called back into the exam room to help with the history.**
- If the physician asks you when Mr. Grey ran out of his blood pressure medication, you tell the physician you do not know.
- If the physician asks about Mr. Grey’s unkempt appearance and/or weight loss, you tell the physician that your father will not let you take care of him. You try to make sure he is eating enough, but it is difficult to stay on top of things because of your own obligations to your family and your job. You tell the physician you really do not know what else to do to make sure your father stays as healthy as can be.
- **Role-play session ends when the physician provides resources and informs your father and you that the physician must report this to Adult Protective Services.**

**Post Role-Playing Discussion Questions**

- How did the scenario feel as the provider?
- How did the scenario feel as the patient’s adult child?
- Provide time for the Observers to comment.
- Did the physician utilize the SMART mnemonic effectively?
- What tools did the participants find useful in this scenario (e.g., algorithms, SMART)?
- What were some of the ‘red flags’ in this case scenario?

*Under-medicated, malnourishment, unkempt appearance, failure to keep appointments*

Discuss why this needs to be reported to Adult Protective Services. *(Refer to your state-specific mandatory reporting requirements for healthcare providers.)*

*The important point to this scenario is that in Utah ‘self-neglect’ is reportable for elder abuse. Discuss the symptoms/signs for elder abuse as listed below.*

- Discuss the concerns that the caregiver and patient might have when you tell them you must report this to Adult Protective Services.

*In a trauma-informed manner, discuss with the family what this report might mean. It is difficult to say with certainty what APS will do but let the family know that you are making the report to provide services to Mr. Grey to improve his health and well-being.*

**Signs and Symptoms Concerning for Vulnerable Adult Abuse**

**Abuse**

•Unexplained bruises/welts •Multiple bruises

•Unexplained injuries •Multiple injuries

•Low self-esteem •Withdrawn/passive/fearful

•Reports/suspicious of sexual abuse •Frequent visits to ED

•Strangulation injury

**Neglect**

•Dehydration •Malnourishment

•Lack of glasses, hearing aids, etc. •Poor hygiene

•Soiled clothes •Over- or under-medicated

•Deserted or abandoned

**Self-Neglect is also reportable**

•Over- or under-medicated •Social isolation

•Malnourishment/dehydration •Unkempt appearance

•Lack of glasses, hearing aids, etc. •Failure to keep appts

**Exploitation**

•Disappearance of possessions •Forced to sell house

•Forced to change will •Overcharged for repairs

•Inadequate living environment •Cannot afford social activities •Unattended •No money for food/clothes

•Forced to sign over control of finances

**Adult Human Trafficking Role-Play 4a—Facilitator**

**Purpose**

The participant practices the skills learned to inquire about human trafficking and exploitation based on presenting symptoms in an adult patient.

**Physician Role**

- You are a resident doing a rotation in the Emergency Department.
- You are seeing an 18-year-old patient with the complaint of a toothache.
- The patient is accompanied by an older woman who identifies herself as the patient’s aunt.
- You notice that the patient seems anxious and has a tattoo of a barcode on their neck.
- Before you begin, you explain to the aunt that you see all your adult patients alone and ask the aunt to wait in the waiting room. The aunt seems annoyed about this and is in a hurry, but she does go to the waiting room.
- **Role-play session begins when you are alone with the patient.**
- Utilize **SMART** to interview this patient. (**S**ymptoms, **M**essage, **A**ssess, **R**esources/**R**eport, **T**reat)

***Note to*** *Facilitator****:*** *The script for the ‘patient role’ is given to the resident who is playing the patient. Give the resident time to read their script and encourage the resident to ad lib as needed. Only the resident playing this role should see the script.*

**Patient Role**

- You are an 18-year-old who comes to the Emergency Department with a very painful toothache which you have been suffering from for weeks now.
- You are working in a massage parlor which is a front for sex trafficking. The woman accompanying you is one of your abuser’s assistants (also called a “bottom girl”) and you were told to say that she is your aunt. You have been threatened with violence if you say anything to the doctor about your current situation. You can only tell the doctor about your medical complaint. You have a tattoo of a barcode on your neck that your trafficker made you get.
- **Role-play session begins once your ‘aunt’ leaves the room, and you are alone with the physician.**
- You are anxious and fearful; answer questions in short responses, act detached and distracted; and keep looking at your phone because you keep getting texts.
- If the physician asks how long you have been ill, you say ***“for weeks.”***
- If the physician asks why it took you so long to get medical care, you say that you didn’t have a way to get to a doctor.
- If the physician asks questions that hint at human trafficking, get irritated and say to the physician,

***“Why are you asking me all these questions? Everything is fine. I’m just working here until I can pay off a debt. How much longer is this going to take?”***

If the physician asks about the tattoo on your neck, say that your boyfriend wanted you to get it.

- **Role-play session ends when the physician gives you resources.**

**Post Role-Playing Discussion Questions**

- How did the scenario feel as the provider?
- How did the scenario feel as the patient?
- Provide time for the Observers to comment.
- Did the physician utilize the SMART mnemonic effectively?
- What tools did the participants find useful in this scenario (e.g. algorithms, SMART)?
- Discuss the venues in which Human Trafficking can occur. (see https://youtu.be/_C03ouE6e20)
- In this scenario, the aunt leaves the room as asked. How would you handle this situation if the aunt refused to leave you alone with the patient?

*If the companion is refusing to leave the room, assess your safety in the situation and discreetly notify security, if needed and if available. With any abuse, ALWAYS assess the risks/benefits of pushing the issue so as not to further endanger the patient when they leave your space (unless it is a mandatory reporting requirement). Sometimes it is in the best interest of the patient to leave the issue and not express your concerns so that you are able to provide medical care and convey to the victim that they have a safe place to return to and the perpetrator will be more likely to allow them to return.*

*There are a variety of ways to make sure that you get time alone with your patient and they include:*

- *Put signs up in your waiting room indicating that all teen and adult patients will be seen alone by the provider at some point during the visit.*
- *Make this a policy for your clinic/ED/hospital.*
- *If you feel that you need to see this patient alone (e.g., patient has an injury, you are concerned for the patient’s imminent safety) notify security who can escort the accompanying adult into the waiting room.* ***Keep in mind that this could increase the risk to the patient.***
- *If you do not feel that you must push the issue at this visit, you can call for a follow-up later and if patient is alone at that time, you can then talk about your concerns.*
- Discuss the reporting requirements in this scenario.
- *(Refer to your state-specific mandatory reporting requirements for healthcare providers.) If the adult patient (who is not a vulnerable adult), does not disclose assault or a life-threatening scenario, then this would not have to be reported to law enforcement. This is distinct from children and vulnerable adults, because if you* ***suspect*** *abuse, neglect, or exploitation then you are mandated to report, regardless of what the patient states. However, remember that* HIPAA 45 CFR 164.512(j)(1)(i), *allows disclosure to “a law enforcement official reasonably able to prevent or lessen a serious and imminent threat to the health or safety of an individual or the public.” This allows the healthcare provider some discretion.*
- Discuss the issues around disclosure for this patient.

**Important Dynamics to Consider**

The victim may not self-identify as a human trafficking victim.

Victims have been conditioned not to trust others.

Victims have been conditioned not to tell the truth.

The trafficker (or their assistant) may be present.

The patient, or the patient’s family, may have been threatened with assault or death if they disclose.

Remember: Prioritize the patient’s medical needs and safety as the primary reason for assessment.

**Questions to Consider**

Is the trafficker present?

Does the patient feel it is safe to talk to her/him alone?

What will happen if the patient does not return to the trafficker?

Does the patient believe he/she or a family member is in danger?

Is the patient a minor?

Consider calling HT Hotline for guidance 888-3737-888

- Discuss the risk factors of human trafficking.


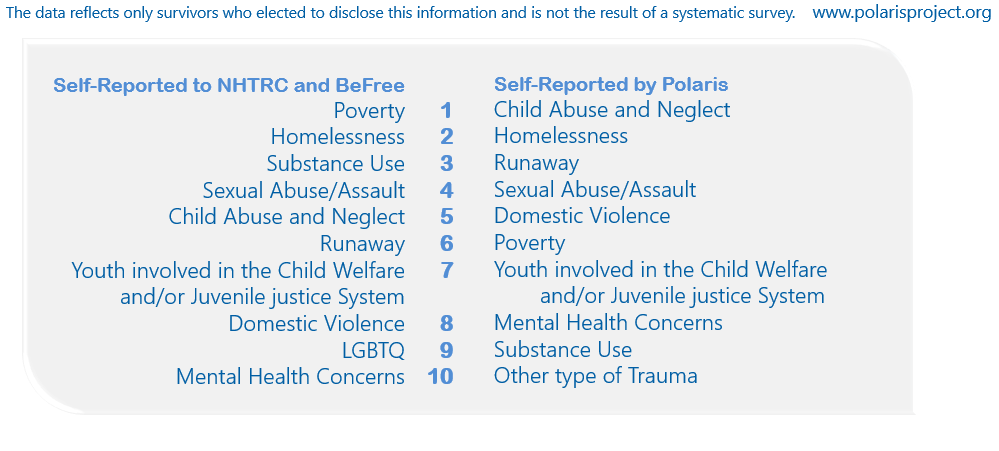


**Child Human Trafficking Role-Play 4b—Facilitator**

**Purpose**

The participant practices the skills learned to inquire about human trafficking and exploitation based on presenting symptoms in a pediatric patient.

**Physician Role**

- You are a resident in your Longitudinal Outpatient Experience Clinic.
- You are seeing a 16-year-old patient with the complaint of follow-up hospitalization for an asthma exacerbation. This is the fourth time the patient has been to the Emergency Department for an asthma exacerbation in the past 2 months and this last time they had to be admitted.
- Upon review of the medications, patient is supposed to be on a daily steroid inhaler and albuterol as needed.
- **Role-play session begins when you walk into the room with the knowledge of the chief complaint of follow-up hospitalization for an asthma exacerbation. The patient’s mother is in the room with the patient.**
- Utilize **SMART** to interview this patient. (**S**ymptoms, **M**essage, **A**ssess, **R**esources/**R**eport, **T**reat)

***Note to*** *Facilitator****:*** *The script for the ‘patient role’ is given to the resident who is playing this part. Give the resident time to read their script and encourage the resident to ad lib as needed. Only the resident playing this role should see the script.*

**Patient Role**

- You are a 16-year-old who comes to the resident clinic for a follow-up of a hospital admission for an asthma exacerbation.
- You have been having multiple asthma exacerbations over the past 2 months and have had to go the Emergency Department multiple times. This last time you had to be admitted.
- You rarely have your albuterol inhaler with you and sporadically take your steroid inhaler because you often stay over with your boyfriend and his friends. Your mother allows you to stay overnight.
- Your boyfriend has started to ask you to have sex with some of his friends. He gets drugs or money from his friends when you have sex with them. You really do not want to, but you do because you are afraid your boyfriend will leave you if you do not do as he asks.
- **Role-play session begins when the physician enters the room to start the visit.** **Your mother is in the room with you when the visit starts.**
- Answer the physician’s questions with short responses, acting distant and detached. Do not make eye contact with the physician and pick at your fingers during the visit.
- If the physician asks how often do you have to use your inhaler, say,

***“I don’t know. I don’t often have it with me.”***

- If the physician asks why you do not often have your inhaler with you, hesitate and say,

***“Well, I often spend the night with friends and I forget to take it with me.”***

- If the physician does not ask your mother to leave the room, then do not provide any more information about spending the night with friends. Do not reveal anything about your situation if your mother is still in the room.
- If the physician does ask your mother to leave the room and you are alone with the physician, then tell the physician what is happening to you.
- **Role-play session ends when the physician finds out you are being sexually exploited/trafficked and informs you that they must report this to law enforcement or if the doctor never gets this information, then the session ends when they give you an asthma action plan.**

**Post Role-Playing Discussion Questions**

- How did the scenario feel as the provider?
- How did the scenario feel as the patient?
- Provide time for the Observers to comment.
- Did the ‘physician’ utilize the SMART mnemonic effectively?
- What tools did the participants find useful in this scenario (e.g., algorithms, SMART)?
- Discuss the venues in which Human Trafficking can occur. (see https://youtu.be/_C03ouE6e20)
- In this scenario, the mother waits in the waiting room if she was asked. How would you handle this situation if the mother refused to leave you alone with her child?

*There are a variety of ways to make sure that you get time alone with your patient and they include:*

- *Put signs up in your waiting room indicating that all teen and adult patients will be seen alone by the provider at some point during the visit. Make this the expectation.*
- *Make this a policy for your clinic/ED/hospital.*
- *If the parent still refuses to leave their child alone with you, there is little you can do to force the issue. If you feel there is imminent danger, then call law enforcement/hospital security/campus police. If you have any suspicion or concern for abuse, neglect, or exploitation (because this is a minor) you should report to CPS or law enforcement as mandated.*

Discuss the reporting requirements in this scenario if the patient DOES disclose or DOES NOT disclose the abuse, depending on what information the physician obtained. *(Refer to your state-specific mandatory reporting requirements for healthcare providers.)*

- *Whether the patient discloses the abuse or not, there is still a cause for concern because her asthma is poorly controlled due to her mother not making sure that she has her proper medications, so you could consider this a medical neglect issue. If the patient tells you about the sex abuse, then this would trigger a mandatory report to CPS or law enforcement and would require an emergency intervention by CPS. This could also be considered supervisory neglect by the mother.*
- How would you inform the parent that due to what the patient has disclosed, you

are required to get law enforcement/CPS involved?

*When the parent returns to the room, you should inform them about your concerns and that you must report this to the authorities. This is not a breach of confidentiality as you are concerned about the patient’s safety (something you should mention to the patient before talking to the patient alone). You can tell the parent the concerns you have or say that the patient has informed you of some events that are going on that make you worried for her well-being and safety, and something that is most likely contributing to her asthma being poorly controlled (bringing it back to the chief complaint). State that given that they are a minor, you are required by law to report this to the authorities. ALWAYS assess the safety of yourself, your staff, and your patient. Get security involved prior to talking to the parent if you think it might turn into an unsafe situation.*

- Discuss the issues around disclosure for this patient and why it is crucial to interview the patient alone.

Important Dynamics to Consider

- - *The victim may not self-identify as a human trafficking victim.*
  - *Victims have been conditioned not to trust others.*
  - *Victims have been conditioned not to tell the truth.*
  - *The trafficker (or their assistant) may be present.*
  - *The patient, or the patient’s family, may have been threatened with assault or death if they disclose.*

Remember: Prioritize the patient’s medical needs and safety as the primary reason for assessment.

- Discuss risk factors of Human Trafficking.


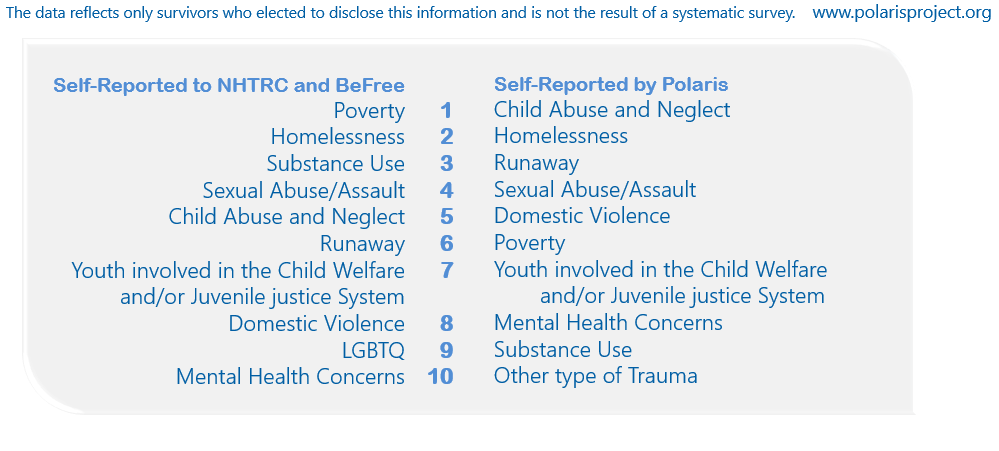


**Sexual Violence Role-Play 5—Facilitator**

**Purpose**

The participant practices the skills learned to inquire about sexual violence based on presenting symptoms in an adult patient.

**Physician Role**

- You are a resident in your Longitudinal Outpatient Experience Clinic.
- You are seeing an 18-year-old college student who presents with the complaint of perineal discomfort.
- **Role-play session begins when you walk into the room with the knowledge of the chief complaint of perineal discomfort.**
- Utilize **SMART** to interview the patient (**S**ymptoms, **M**essage, **A**ssess, **R**esources/**R**eport, **T**reat).

***Note to*** *Facilitator****:*** *The script for the ‘patient role’ is given to the resident who is playing this part. Give the resident time to read their script and encourage the resident to ad lib as needed. Consider using the facilitator as the patient in this scenario. Only the person playing this role should see the script.*

**Patient Role**

- You are an 18-year-old college student presenting with concerns of perineal discomfort.
- You were at a party last night and were drinking alcohol. You do not remember much of last night, but when you woke up in your dorm room this morning, your underwear was missing, and you feel sore in the perineal area. You are worried someone had sex with you last night, but you are not sure.
- **Role-play session begins when the physician enters the room to start the visit.**
- When the physician asks you about your concerns, you tell the physician that you are worried that someone might have had sex with you last night. You would like to be tested for STIs (and if female ‘actor’, would like medication to prevent a pregnancy). You are anxious and nervous and tell the physician that you do not want your parents to find out that you were drinking.
- If the physician tells you that this must be reported to law enforcement, you get upset and say,

***“I don’t want to get anyone in trouble.”***

- If the physician asks you if you want a forensic exam, you ask,

***“What does that mean?”***

When the physician explains this to you, you say,

***“No, I don’t want that exam. All I want is to be tested for STIs*** *(and if female you want medication to prevent a pregnancy).* ***You don’t have to tell my parents, do you?”***

- **Role-play session ends when physician either explains that they would like to do an exam to make sure you are medically stable, gives you treatment for STIs or both and informs you of their reporting requirement to law enforcement.**

**Post Role-Playing Discussion Questions**

- How did the scenario feel as the provider?
- How did the scenario feel as the patient?
- Provide time for the Observers to comment.
- Did the physician utilize the SMART mnemonic effectively?
- What tools did the participants find useful in this scenario (e.g., algorithms, SMART)?

Discuss why this had to be reported to law enforcement. Utilize your state’s laws to explain consent and mandatory reporting issues. *(Refer to your state-specific mandatory reporting requirements for healthcare providers.)*

*In Utah, this case would require a report to law enforcement, because healthcare providers are mandated to report any assaultive injury for which they are providing care. Even though, in this scenario, the patient does not know exactly what happened, the very fact that they do not know what exactly happened indicates that if someone had sex with them this was without their consent.* Discuss the state’s statutes on what constitutes consent. *(Refer to your state-specific mandatory reporting requirements for healthcare providers.)*

- Discuss the Campaign which is a public awareness campaign uniquely focused on the response to sexual assault—knowing how to respond is critical. For more information go to <https://www.startbybelieving.org/>.

**Vulnerable Adult Role-Play 6—Facilitator**

**Purpose**

The participant practices the skills learned to inquire about abuse/neglect exposure based on presenting symptoms in a vulnerable adult patient.

**Physician Role**

- You are a resident in your Longitudinal Outpatient Experience Clinic.
- Alex, who is brought in by his parent and guardian, is a non-verbal, non-ambulating 19-year-old with cerebral palsy, global developmental delay and is g-tube dependent who is being seen for a check-up.
- You noticed that they have missed his last two appointments, one of them for a weight check.
- His growth chart shows a decrease in weight and his weight percentile has now dipped below the 3^rd^ percentile (from the 20^th^% percentile in the past).
- Physical exam reveals a thin male with poor hygiene and body odor.
- **Role-play starts as you walk into the room to begin the clinic visit.**
- Utilize **SMART** to interview Alex’s parent. (**S**ymptoms, **M**essage, **A**ssess, **R**esources/**R**eport, **T**reat)

***Note to*** *Facilitator****:*** *The script for the ‘Alex’s Parent role’ is given to the resident who is playing this part. Give the resident time to read their script and encourage the resident to ad lib as needed. Only the resident playing this role should see the script.*

**Alex’s Parent Role**

- You are the parent and guardian of Alex, a 19-year-old, non-verbal, non-ambulating male with cerebral palsy and global developmental delay who is g-tube dependent.
- You are bringing him to the physician today for a check-up.
- You missed two of his previous appointments because of multiple stressors in your life. Alex has been losing weight because you cannot afford to buy enough of his formula.
- New stressors in your life include that you have separated from your partner 9 months ago, so you no longer have the partner’s help and income. Alex does not have medical insurance, so the loss of income has put more hardship on your family.
- **Role-play session begins when the physician comes into the room.**
- If the physician asks about Alex’s weight loss, tell the physician that you are having difficulty affording the formula, so you have had to decrease how much he is getting.
- If the physician asks what your biggest financial barriers are, let the physician know the above stressors.
- **Scenario ends when the physician provides resources and/or discusses involving APS to help the family get resources.**

**Post Role-Playing Discussion Questions**

- How did the scenario feel as the provider?
- How did the scenario feel as the parent?
- Provide time for the Observers to comment.
- Did the physician utilize the SMART mnemonic effectively?
- What tools did the participants find useful in this scenario (e.g., algorithms, SMART)?
- What were some of the ‘red flags’ in this case scenario?

*Malnourishment due to poverty, poor hygiene, soiled clothing, medical neglect*

- Discuss if this needs to be reported to Adult Protective Services.

*(Refer to your state-specific mandatory reporting requirements for healthcare providers.) In Utah, this would have to be reported to Adult Protective Services as an example of neglect. The purpose of involving Adult Protective Services is to provide resources to Alex and his parent to improve his health.*

- Discuss the social determinants of health and how this affects medical care.

Social Determinants of Health^1,2^

Social Determinants of Health (SDOH) are conditions in which we are born, grow, live, work and age. SDOH are shaped by money, power, and resources.

SDOH are influenced by

- Biology: Sex and Age
- Behavior: Smoking, Drinking, Drug Use, Physical Inactivity, Poor Diet

These are the leading causes of preventable death in the US

- Social: Discrimination, Income, Gender
- Physical: Home, Density, Green Space
- Health Services: Access, Insurance

^1^Heron M. Deaths: Leading causes for 2016. National Vital Statistics Reports; vol 67 no 6. Hyattsville, MD: National Center for Health Statistics. 2018

^2^Robert Wood Johnson Foundation and University of California, San Francisco, Center on Social Disparities in Health, Paula Braveman, Susan Egerter. Copyright 2008 Robert Wood Johnson Foundation/Overcoming Obstacles to Health

See the CDC link: <https://www.cdc.gov/socialdeterminants/> for more information.
