# Supplementary material for: Patient Abuse, Neglect, and Exploitation: Why Physicians Need to Be Trauma-Informed
Source: MedEdPORTAL. 2024 Apr 23;20:11391. doi: 10.15766/mep_2374-8265.11391 (PMC11035495; doi:10.15766/mep_2374-8265.11391)
Supplement: Supplementary file 1 — Prework Articles.docxDidactic.pptxRole-Playing Facilitator Guide.docxSMART Tool.docxPretest-Posttest Survey.docxPostsession Materials.docx [file mep_2374-8265.11391-s001.zip › D. SMART Tool.docx]

Appendix D

*Note to Facilitator: This is a summary of the SMART tool that the residents can use as a general guide when interacting with patients who disclose trauma.*

SMART tool developed by Kathleen Franchek-Roa MD and Aarti Vala MD

Abbreviations: IPV, intimate partner violence; SDOH, social determinants of health.

**S**

M

A

R

T

**Screen vs Ask**

**Screen** all women of reproductive age for IPV victimization

**Ask** anyone with symptoms of trauma

**Message**

I am sorry this happened to you.

I believe you and I can help.

**Assess Symptoms & Danger**

Linking the trauma exposure to patient’s symptomatology aids in the understanding of the connection between exposure and health. *“This may be contributing to why we are having such a hard time managing your symptoms.”*

**Resources**

2-1-1 List/include SDOH Resources

**Report**

Discuss any reporting requirements with a trauma-informed perspective

**Treat**

Discuss the management of symptoms with the knowledge about their trauma because now you can provide effective interventions.
