# Supplementary material for: Patient Abuse, Neglect, and Exploitation: Why Physicians Need to Be Trauma-Informed
Source: MedEdPORTAL. 2024 Apr 23;20:11391. doi: 10.15766/mep_2374-8265.11391 (PMC11035495; doi:10.15766/mep_2374-8265.11391)
Supplement: Supplementary file 1 — Prework Articles.docxDidactic.pptxRole-Playing Facilitator Guide.docxSMART Tool.docxPretest-Posttest Survey.docxPostsession Materials.docx [file mep_2374-8265.11391-s001.zip › E. Pretest-Posttest Survey.docx]

**Patient Abuse, Neglect, and Exploitation Education Session**

***Note to Facilitator:*** *This is the Retrospective Pretest-Posttest Evaluation form that is given to the residents AFTER the educational session is completed. A link to the evaluation form is sent to the residents via email.*

**Please complete the survey below. Thank you.**

| Please enter today’s date: | | | | |
| --- | --- | --- | --- | --- |
| Did the session meet your education needs? | | ○ Yes | ○ Somewhat | ○ No |
| Did the information presented provide new ideas/information? | | ○ Yes | ○ Somewhat | ○ No |
|  | | | | |
| **Rate your confidence in being able to identify and intervene with patients who are victims of abuse, neglect, and exploitation.** | | | | |
| Before the session  After the session | No Confidence  ○  ○ | Low Confidence  ○  ○ | Moderate Confidence  ○  ○ | High Confidence  ○  ○ |
| **Rate your confidence in your ability to: Explain the link between childhood adversity and risk for poor health across the lifespan. (Objective 1)** | | | | |
| Before the session  After the session | No Confidence  ○  ○ | Low Confidence  ○  ○ | Moderate Confidence  ○  ○ | High Confidence  ○  ○ |
| **Rate your confidence in your ability to: Utilize a trauma-informed approach when interacting with patients to reduce or mitigate the consequences of these adverse experiences. (Objective 2)** | | | | |
| Before the session  After the session | No Confidence  ○  ○ | Low Confidence  ○  ○ | Moderate Confidence  ○  ○ | High Confidence  ○  ○ |
| **Rate your confidence in your ability to: Discuss the prevalence of abuse, neglect, and/or exploitation in terms of the public health impact to healthcare. (Objective 3)** | | | | |
| Before the session  After the session | No Confidence  ○  ○ | Low Confidence  ○  ○ | Moderate Confidence  ○  ○ | High Confidence  ○  ○ |

| **Rate your confidence in your ability to: Employ best practices when evaluating patients who are victims of abuse, neglect, and/or exploitation. (Objective 4)** | | | | |
| --- | --- | --- | --- | --- |
| Before the session  After the session | No Confidence  ○  ○ | Low Confidence  ○  ○ | Moderate Confidence  ○  ○ | High Confidence  ○  ○ |
|  | | | |  |
| This educational session provided practical suggestions I can use in my clinical endeavors. | | ○ Yes | ○ Somewhat | ○ No |
|  | | | | |
| What changes will you incorporate into your future clinical endeavors as a result of the knowledge acquired during this activity? | | | | |
|  | | | | |
| How can we improve the session? | | | | |

| Please indicate your age. |
| --- |
|  |
| Please indicate your gender. ○ Female  ○ Male  ○ Other  ○ Would prefer not to answer |
|  |
| Please indicate your area of training ○ Pediatrics  ○ Medicine/Pediatrics  ○ Triple Board  ○ Internal Medicine ○ Family Medicine  ○ Medical Student  ○ Fellow  ○ Other |
|  |
| Please indicate your year in residency. ○ First  ○ Second  ○ Third  ○ Fourth ○ Fifth  ○ Fellow  ○ Other |
