# Supplementary material for: Patient Abuse, Neglect, and Exploitation: Why Physicians Need to Be Trauma-Informed
Source: MedEdPORTAL. 2024 Apr 23;20:11391. doi: 10.15766/mep_2374-8265.11391 (PMC11035495; doi:10.15766/mep_2374-8265.11391)
Supplement: Supplementary file 1 — Prework Articles.docxDidactic.pptxRole-Playing Facilitator Guide.docxSMART Tool.docxPretest-Posttest Survey.docxPostsession Materials.docx [file mep_2374-8265.11391-s001.zip › A. Prework Articles.docx]

**PATIENT ABUSE, NEGLECT AND EXPLOITATION—**

**Why We Need to be Trauma-Informed**

**(Prework)**

**Developed by Kathy Franchek-Roa, MD and Aarti Vala, MD**

**(Updated July 2021)**

*Note to Facilitator: This information sheet and the articles are sent to residents about 1 week before the session.*

Goal:

The goal is to educate physicians and other healthcare professionals on becoming knowledgeable in identifying and intervening with patients who are victims of abuse, neglect, and/or exploitation in order to provide effective, appropriate medical care.

**Objectives:**

At the conclusion of this educational session, participants will be able to

1. Explain the link between childhood adversity and risk for poor health across the lifespan;
2. Utilize a trauma-informed approach when interacting with patients to reduce or mitigate the consequences of these adverse experiences;
3. Discuss the prevalence of abuse, neglect, and/or exploitation in terms of the public health impact to healthcare; and
4. Employ best practices when evaluating patients who are victims of abuse, neglect, and/or exploitation.

**Educational Session—Modified to accommodate COVID-19 restrictions**

A 4 hour session with Dr. Franchek and/or Dr. Vala that will include: (1) a discussion to define the role of the physician and other healthcare providers in identifying and assisting victims of abuse, neglect, and exploitation; (2) a discussion with a survivor of intimate partner violence or adverse childhood experiences to obtain insight into how the medical community can help families living with violence; (3) ‘virtual tour’ of the local domestic violence shelter; and (4) role-playing sessions so that the participants can improve their skills in providing care to patients who are experiencing abuse, neglect, and exploitation.

**Recommended Reading:**

Reflective Articles:

- Peabody FW. The care of the patient. JAMA. 1927;88(12):877-882.
- Sweetnam S. Where do you think domestic abuse hurts most? Violence Against Women. 2013;19(1):133-138.

Health Care Providers’ Response:

- Deshpande NA, Lewis-O’Connor A. Screening for intimate partner violence during pregnancy. Rev Obstet Gynecol 2013;6(3/4):141-148.
- Miller E, McCaw B. Intimate partner violence. NEJM. 2019; 380:850-857
- Garner A, Yogman M, Committee on Psychosocial Aspects of Child and Family Health, Section on Developmental and Behavioral Pediatrics, Council on Early Childhood. Preventing Childhood Toxic Stress: Partnering With Families and Communities to Promote Relational Health. Pediatrics. 2021;148(2):e2021052582

Faculty: Kathy Franchek, M.D. Aarti Vala, MD
