# Supplementary material for: Patient Abuse, Neglect, and Exploitation: Why Physicians Need to Be Trauma-Informed
Source: MedEdPORTAL. 2024 Apr 23;20:11391. doi: 10.15766/mep_2374-8265.11391 (PMC11035495; doi:10.15766/mep_2374-8265.11391)
Supplement: Supplementary file 1 — Prework Articles.docxDidactic.pptxRole-Playing Facilitator Guide.docxSMART Tool.docxPretest-Posttest Survey.docxPostsession Materials.docx [file mep_2374-8265.11391-s001.zip › B. Didactic.pptx]

## Slide 1
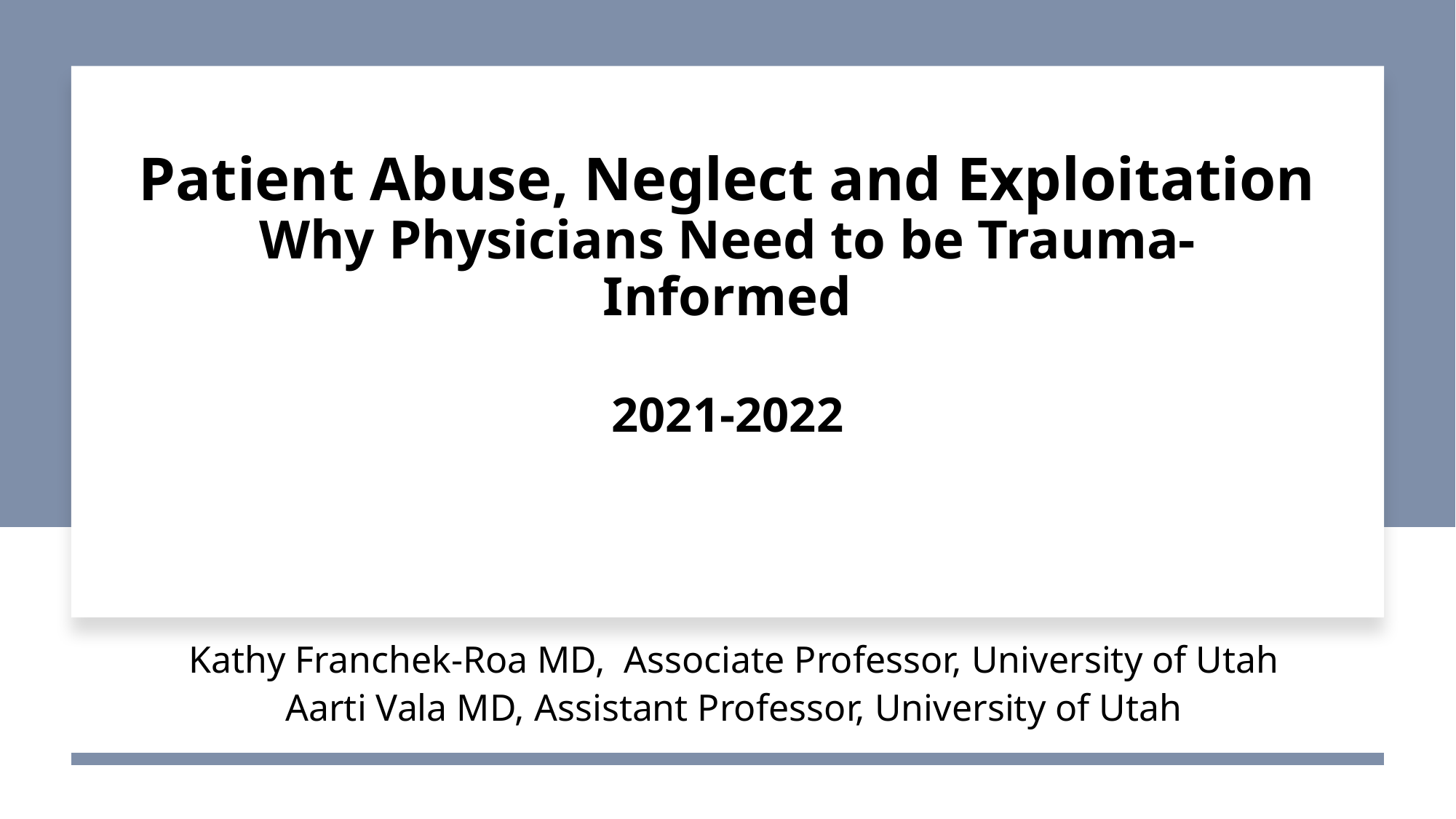

# Patient Abuse, Neglect and Exploitation Why Physicians Need to be Trauma-Informed 2021-2022
Kathy Franchek-Roa MD, Associate Professor, University of Utah
Aarti Vala MD, Assistant Professor, University of Utah

## Slide 2
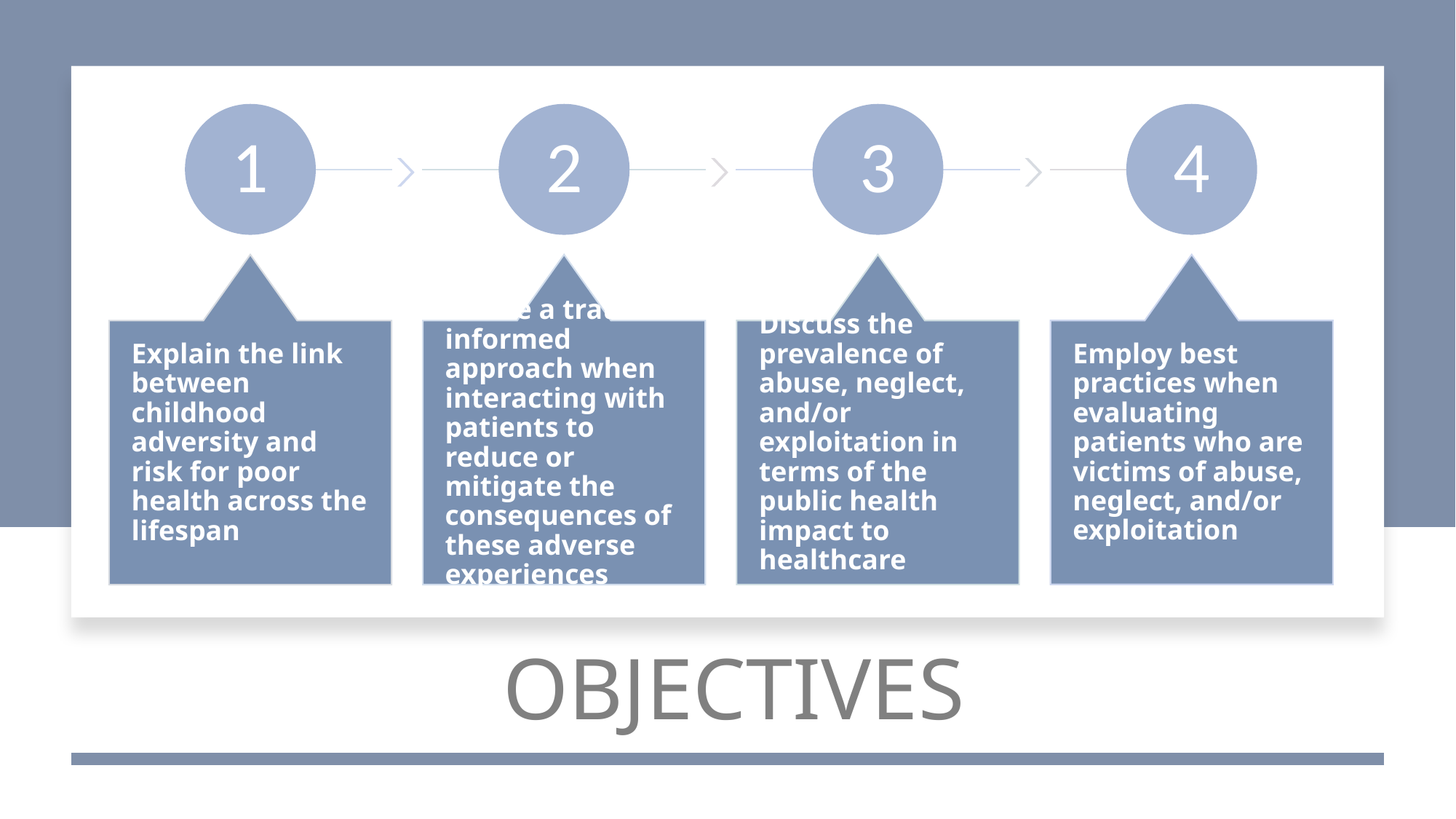

2
Utilize a trauma-informed approach when interacting with patients to reduce or mitigate the consequences of these adverse experiences
3
Discuss the prevalence of abuse, neglect, and/or exploitation in terms of the public health impact to healthcare
4
Employ best practices when evaluating patients who are victims of abuse, neglect, and/or exploitation
1
Explain the link between childhood adversity and risk for poor health across the lifespan
OBJECTIVES

## Slide 3
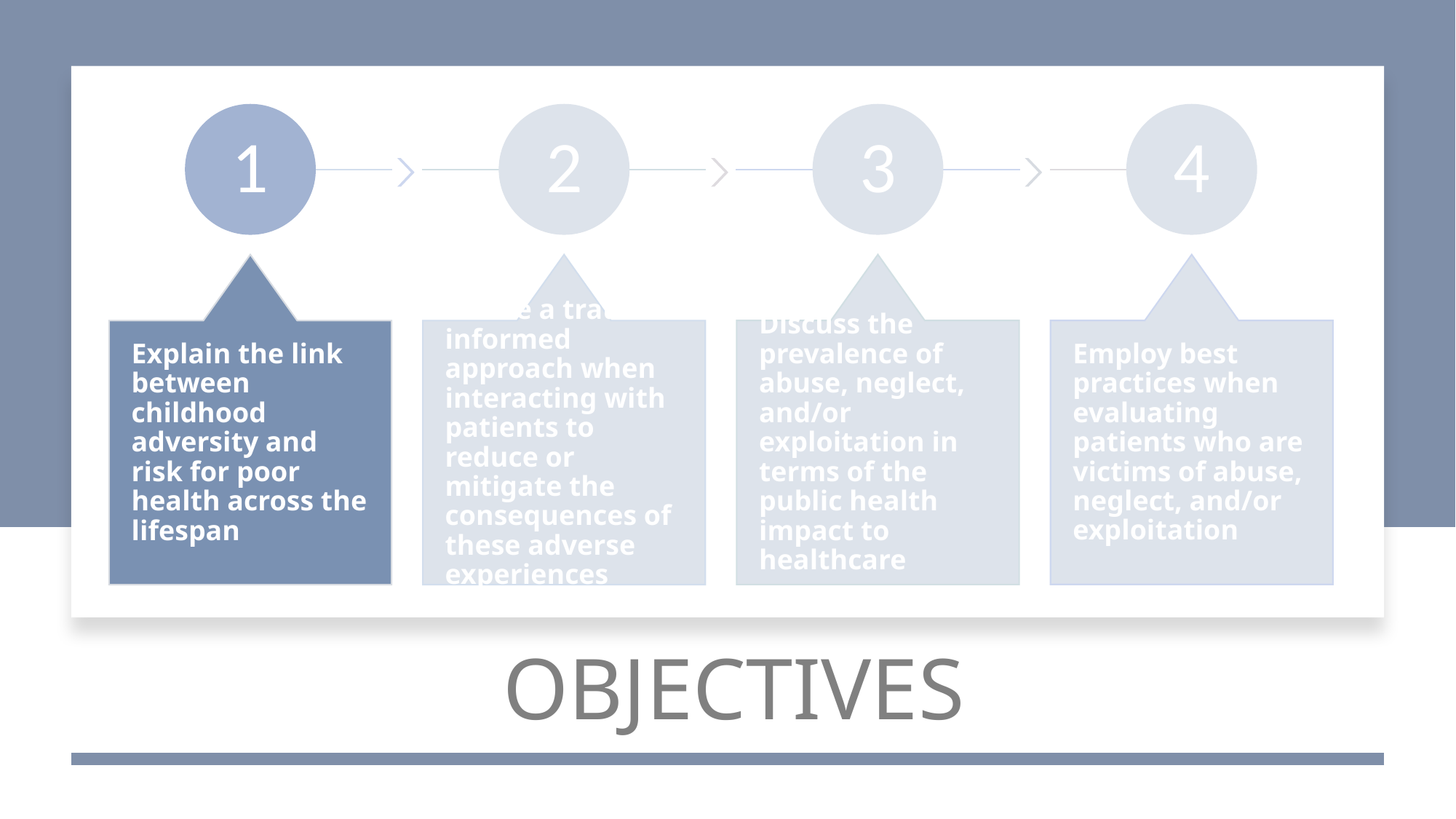

2
Utilize a trauma-informed approach when interacting with patients to reduce or mitigate the consequences of these adverse experiences
3
Discuss the prevalence of abuse, neglect, and/or exploitation in terms of the public health impact to healthcare
4
Employ best practices when evaluating patients who are victims of abuse, neglect, and/or exploitation
1
Explain the link between childhood adversity and risk for poor health across the lifespan
OBJECTIVES

## Slide 4
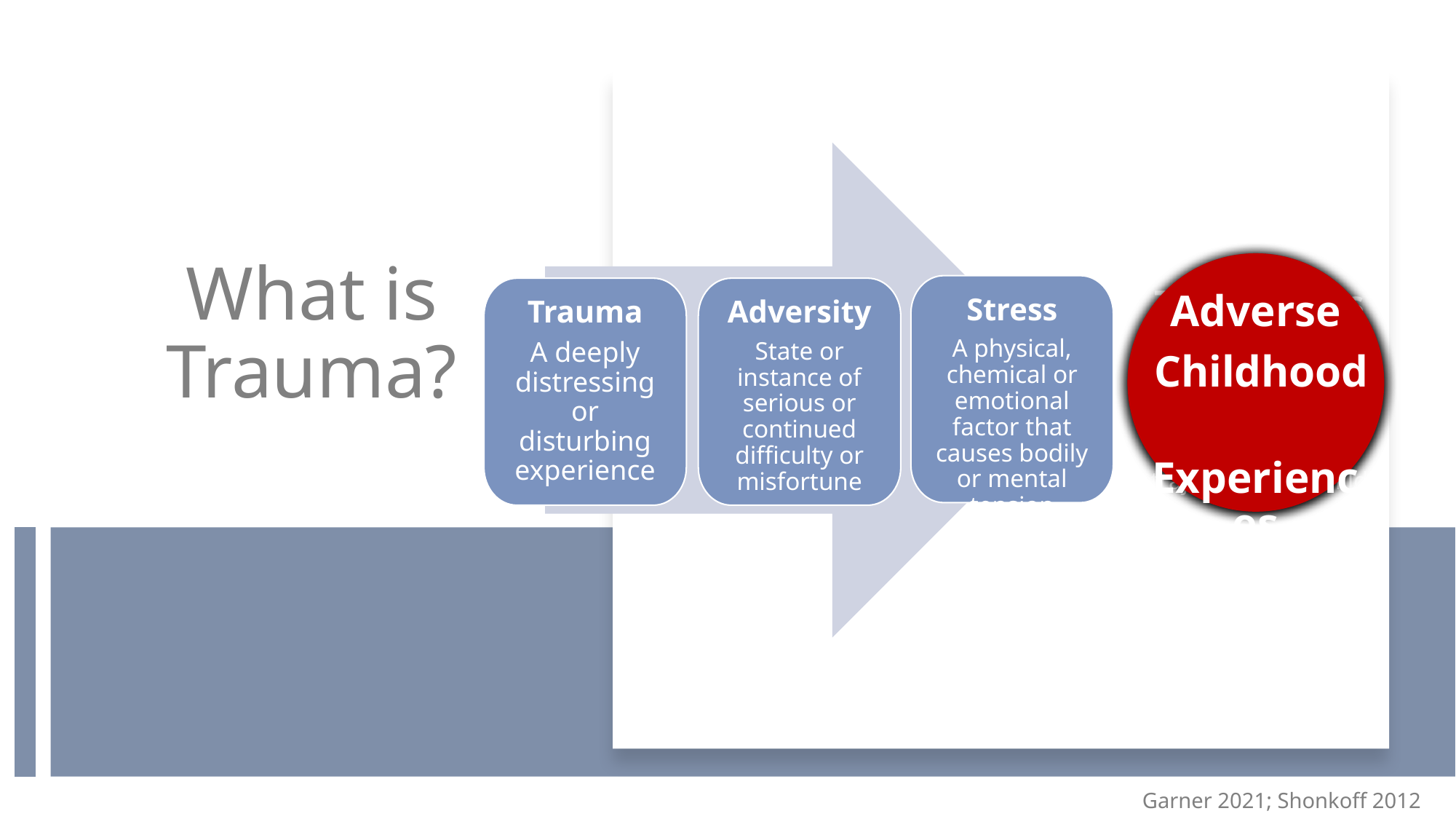

# What is Trauma?
Adverse
 Childhood
 Experiences
Toxic Stress
A trauma, adversity or stress that interrupts the normal developmental trajectory of a child
Stress
A physical, chemical or emotional factor that causes bodily or mental tension
Trauma
A deeply distressing or disturbing experience
Adversity
State or instance of serious or continued difficulty or misfortune
Garner 2021; Shonkoff 2012

## Slide 5
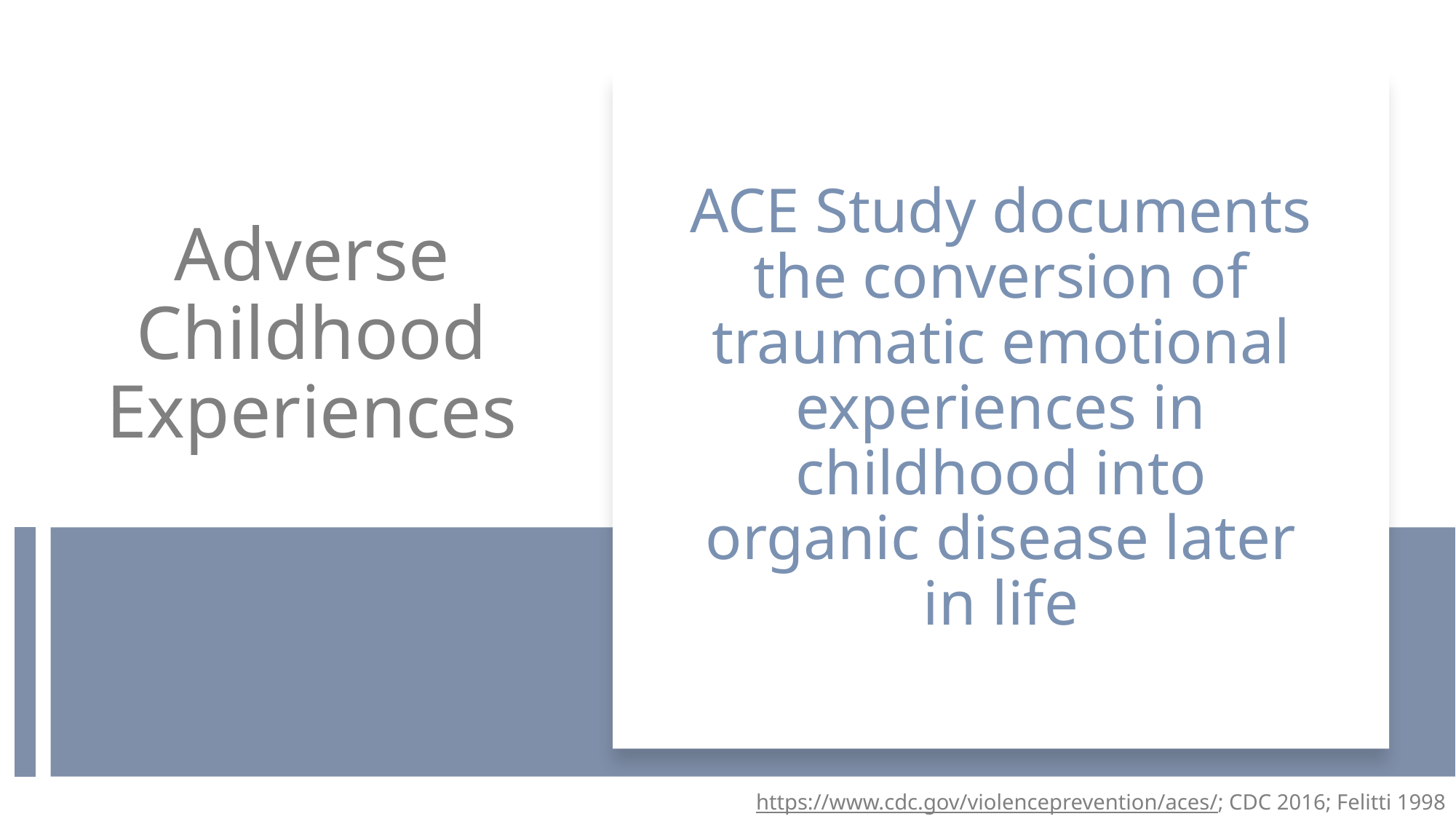

# Adverse Childhood Experiences
ACE Study documents the conversion of traumatic emotional experiences in childhood into organic disease later in life
https://www.cdc.gov/violenceprevention/aces/; CDC 2016; Felitti 1998

## Slide 6
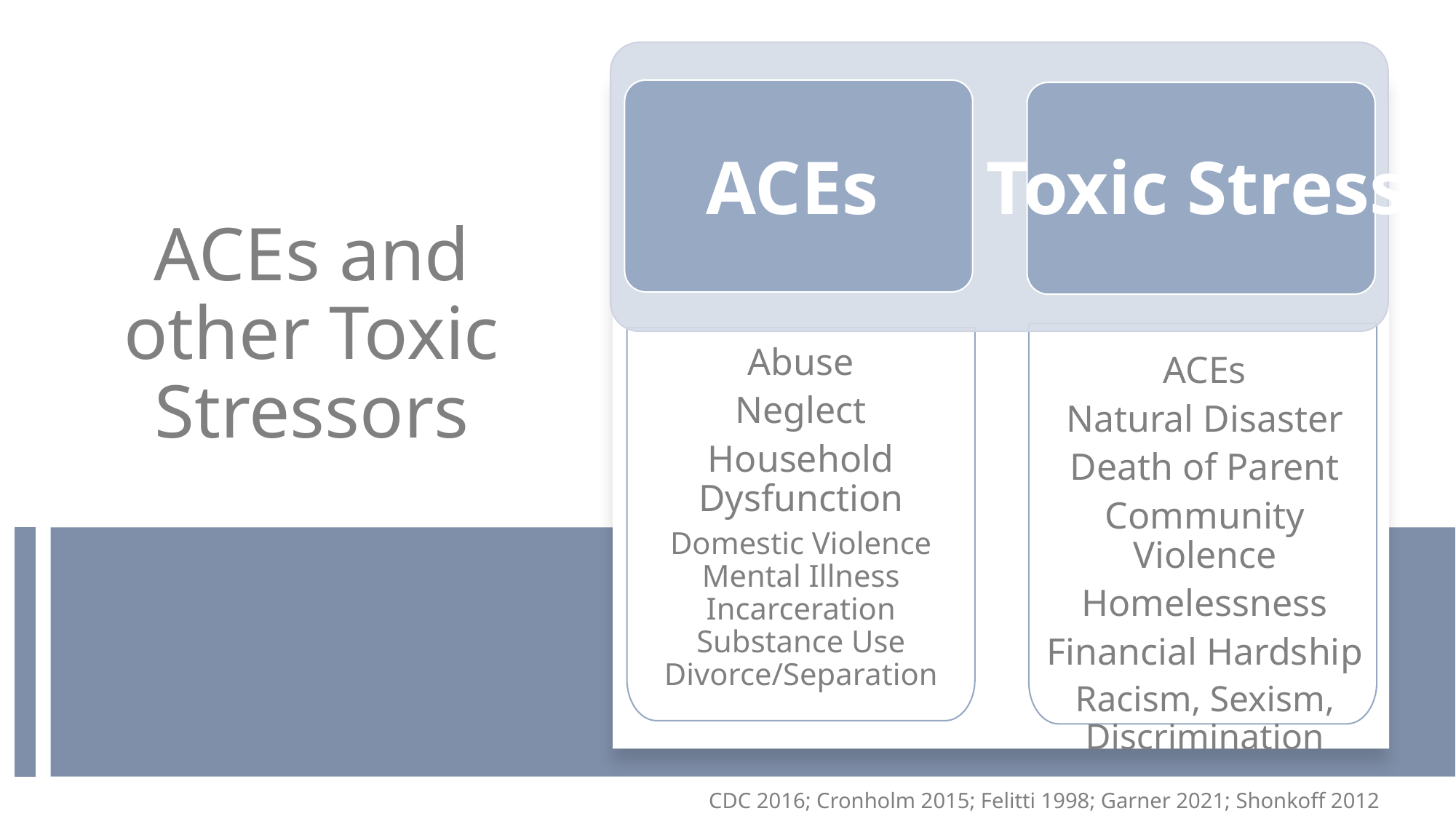

ACEs
Toxic Stress
# ACEs and other Toxic Stressors
Abuse
Neglect
Household Dysfunction
Domestic Violence
Mental Illness
Incarceration
Substance Use
Divorce/Separation
ACEs
Natural Disaster
Death of Parent
Community Violence
Homelessness
Financial Hardship
Racism, Sexism, Discrimination
CDC 2016; Cronholm 2015; Felitti 1998; Garner 2021; Shonkoff 2012

## Slide 7
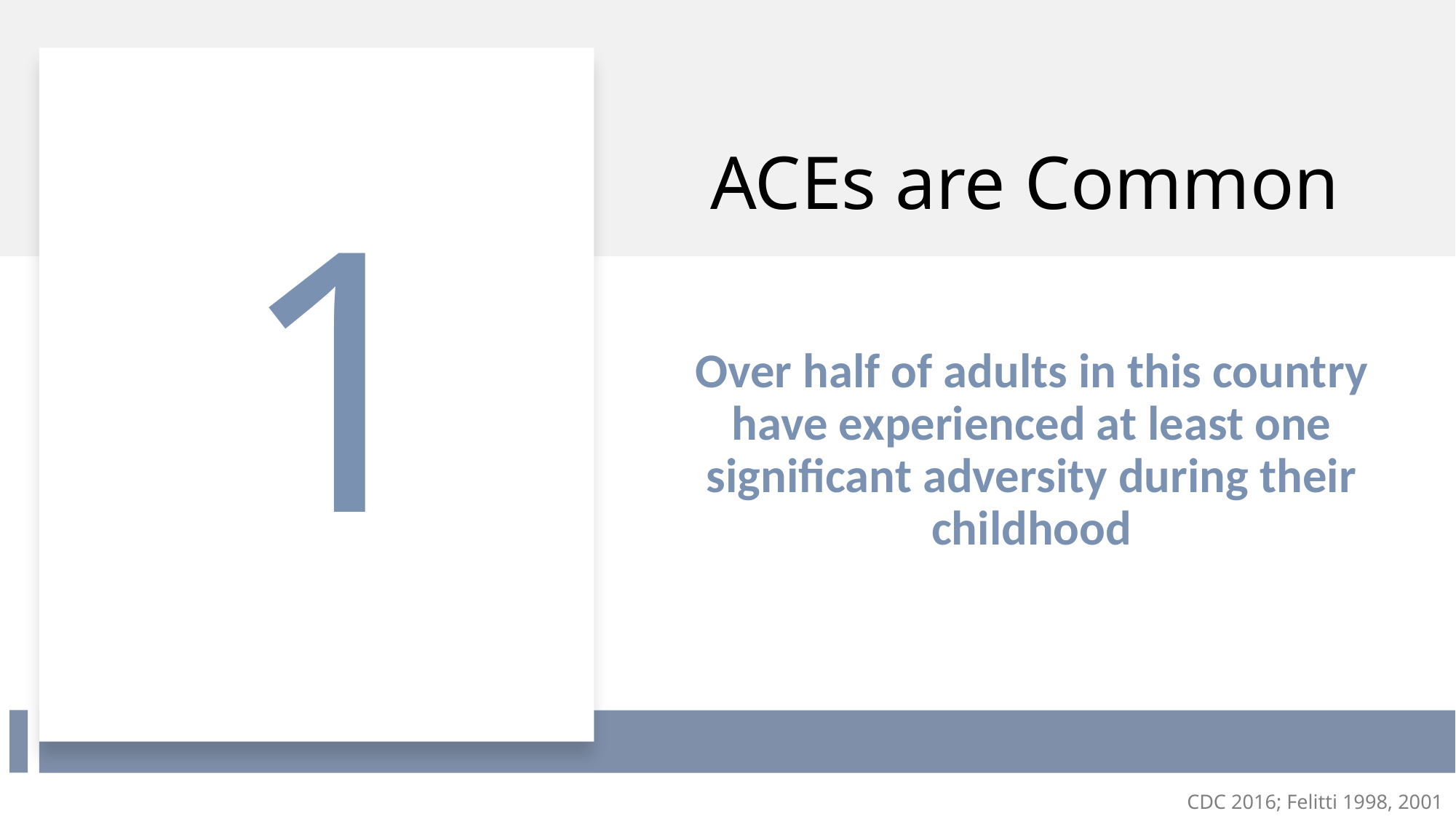

ACEs are Common
1
Over half of adults in this country have experienced at least one significant adversity during their childhood
CDC 2016; Felitti 1998, 2001

## Slide 8
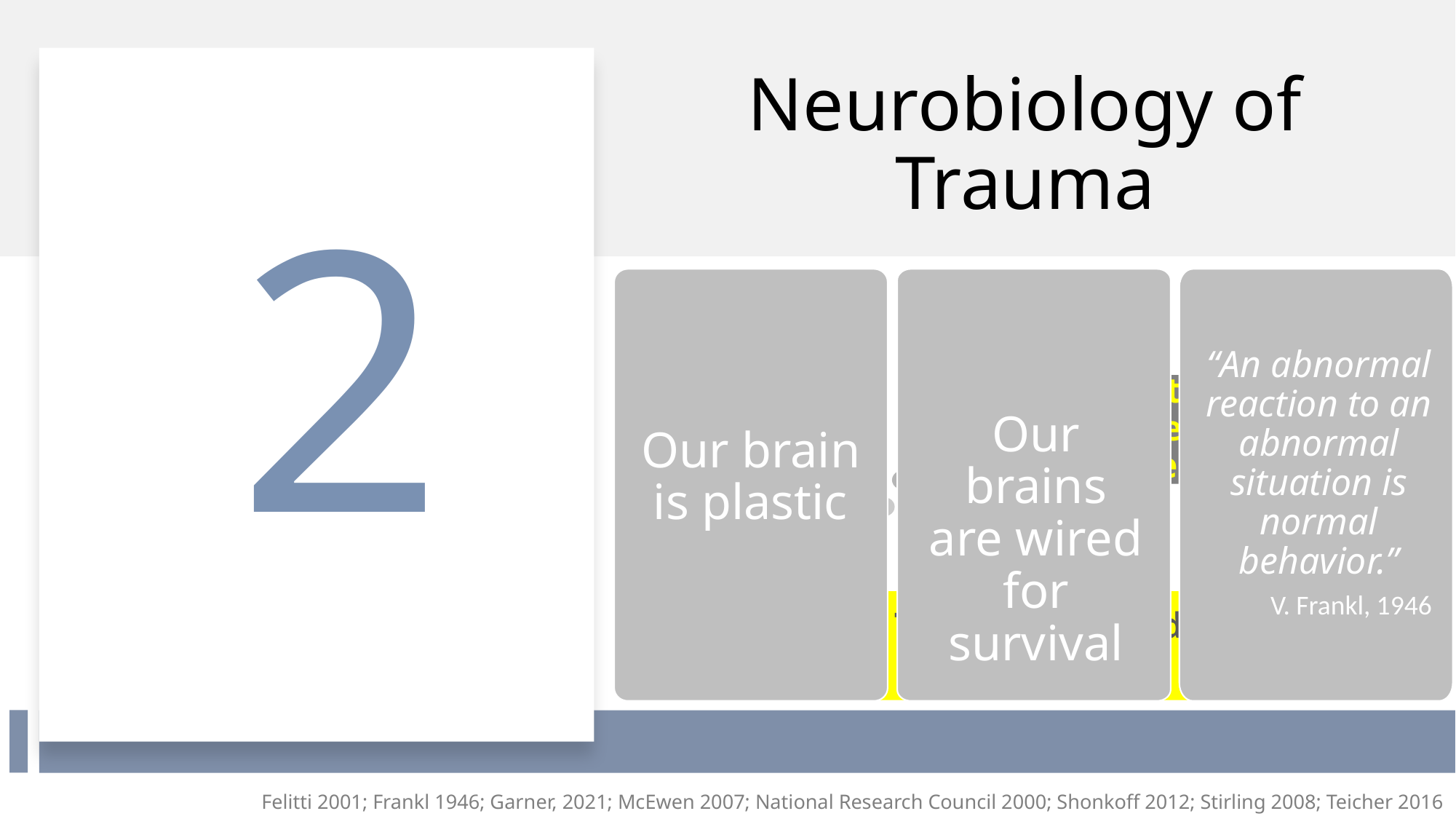

Neurobiology of Trauma
2
Our brains are wired for survival
Our brain is plastic
“An abnormal reaction to an abnormal situation is normal behavior.”
V. Frankl, 1946
Into the lead of a depressed, diseased adult
Turning the gold of a newborn
Felitti 2001; Frankl 1946; Garner, 2021; McEwen 2007; National Research Council 2000; Shonkoff 2012; Stirling 2008; Teicher 2016

## Slide 9
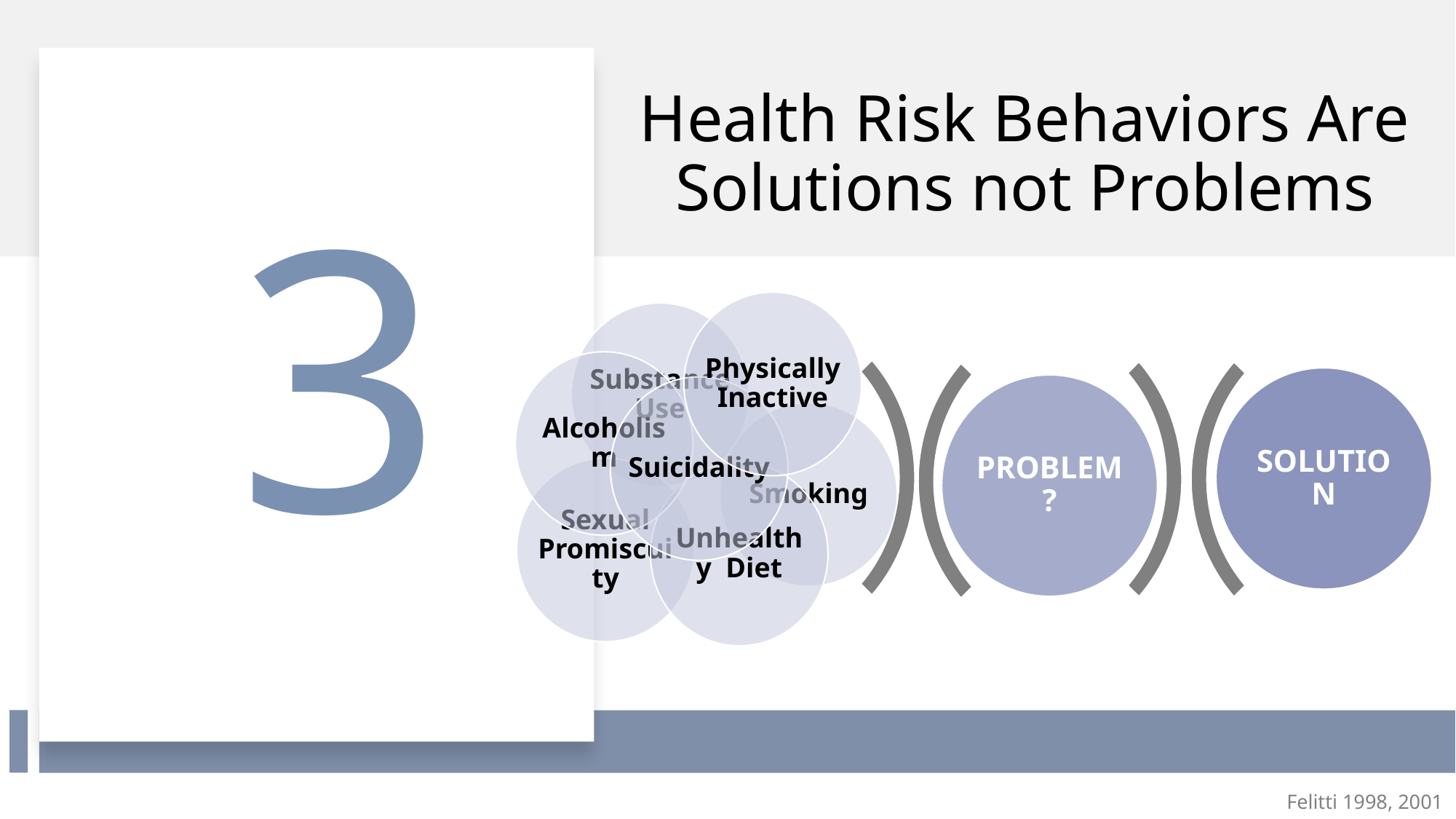

# Health Risk Behaviors Are Solutions not Problems
3
Physically Inactive
Substance Use
Alcoholism
SOLUTION
PROBLEM?
Suicidality
Smoking
Sexual Promiscuity
Unhealthy Diet
Felitti 1998, 2001

## Slide 10
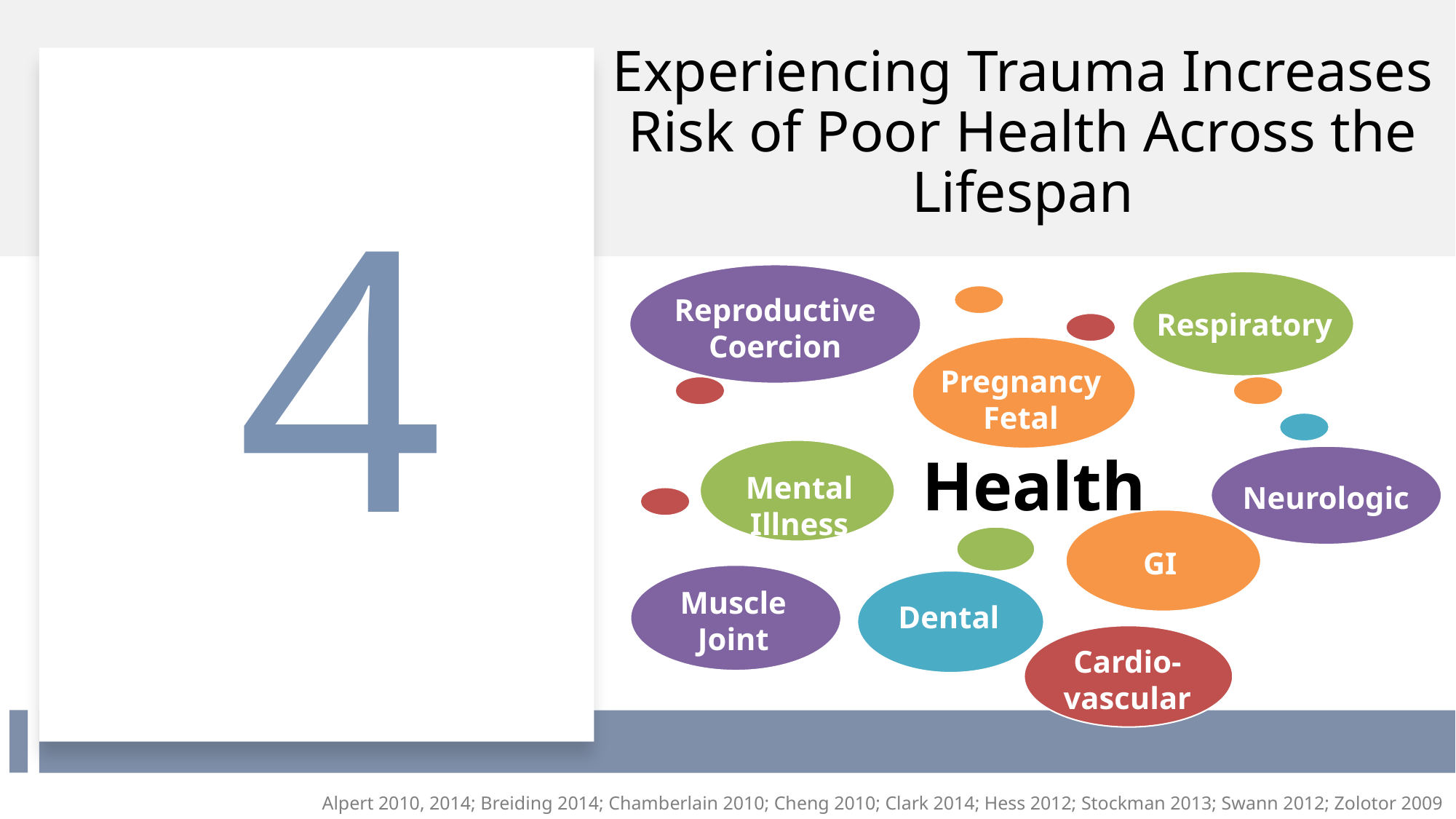

# Experiencing Trauma Increases Risk of Poor Health Across the Lifespan
4
Reproductive Coercion
Respiratory
Pregnancy
Fetal
Health
Mental Illness
Neurologic
GI
Muscle
Joint
Dental
Cardio-vascular
Alpert 2010, 2014; Breiding 2014; Chamberlain 2010; Cheng 2010; Clark 2014; Hess 2012; Stockman 2013; Swann 2012; Zolotor 2009

## Slide 11
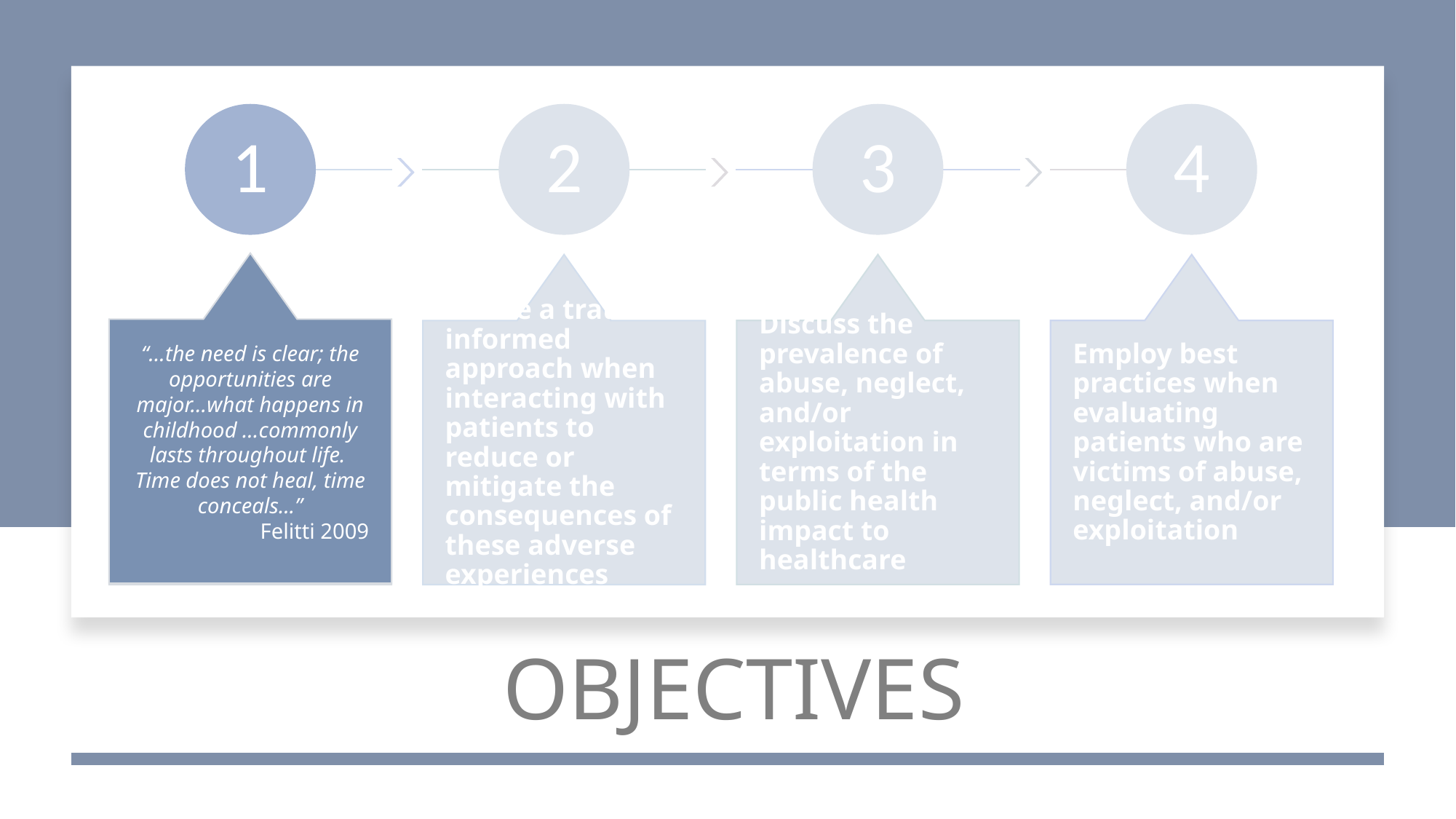

2
Utilize a trauma-informed approach when interacting with patients to reduce or mitigate the consequences of these adverse experiences
3
Discuss the prevalence of abuse, neglect, and/or exploitation in terms of the public health impact to healthcare
4
Employ best practices when evaluating patients who are victims of abuse, neglect, and/or exploitation
1
Explain the link between childhood adversity and risk for poor health across the lifespan
“…the need is clear; the opportunities are major…what happens in childhood …commonly lasts throughout life. Time does not heal, time conceals...”
Felitti 2009
OBJECTIVES

## Slide 12
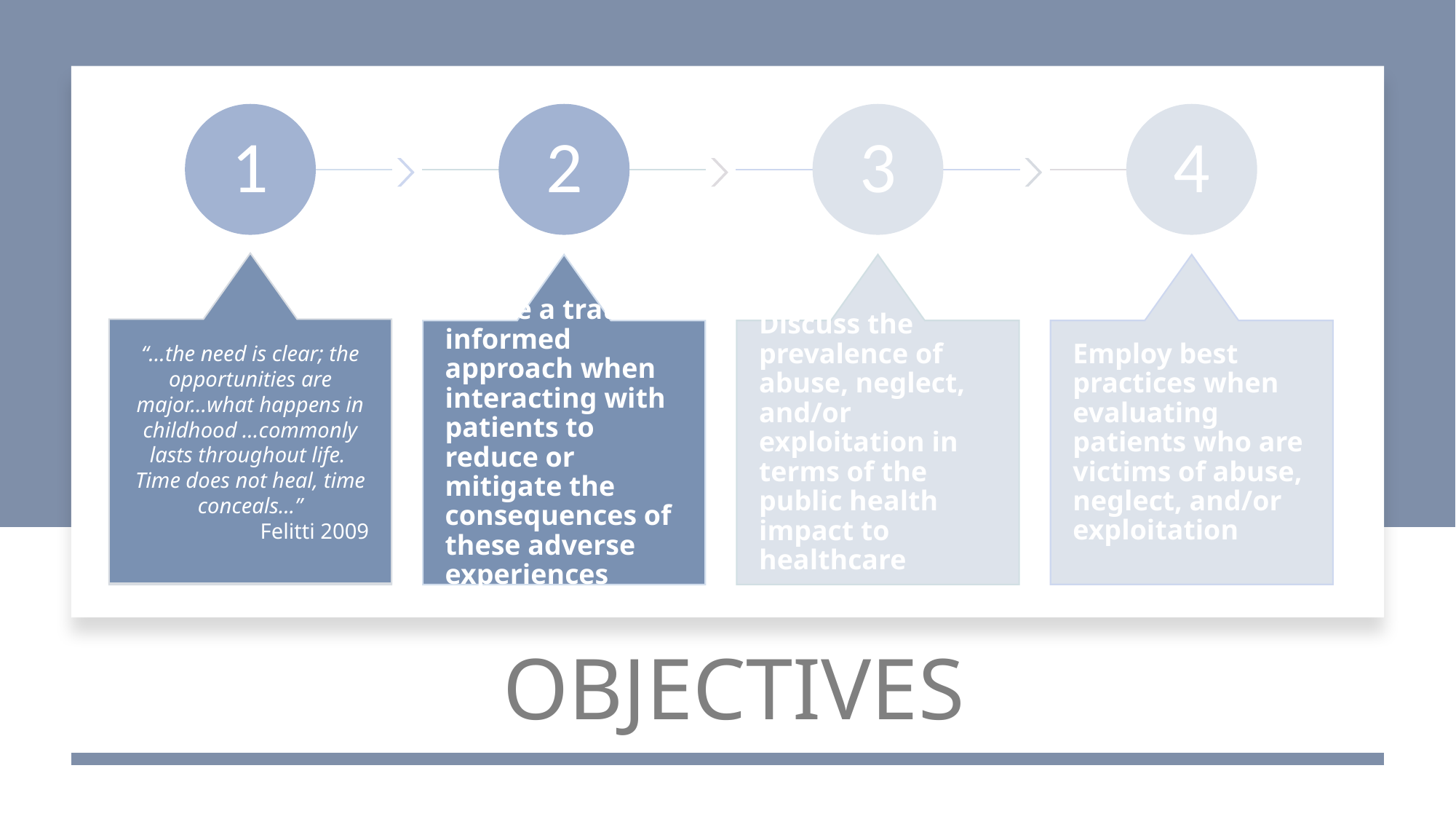

2
Utilize a trauma-informed approach when interacting with patients to reduce or mitigate the consequences of these adverse experiences
3
Discuss the prevalence of abuse, neglect, and/or exploitation in terms of the public health impact to healthcare
4
Employ best practices when evaluating patients who are victims of abuse, neglect, and/or exploitation
1
Explain the link between childhood adversity and risk for poor health across the lifespan
“…the need is clear; the opportunities are major…what happens in childhood …commonly lasts throughout life. Time does not heal, time conceals...”
Felitti 2009
OBJECTIVES

## Slide 13
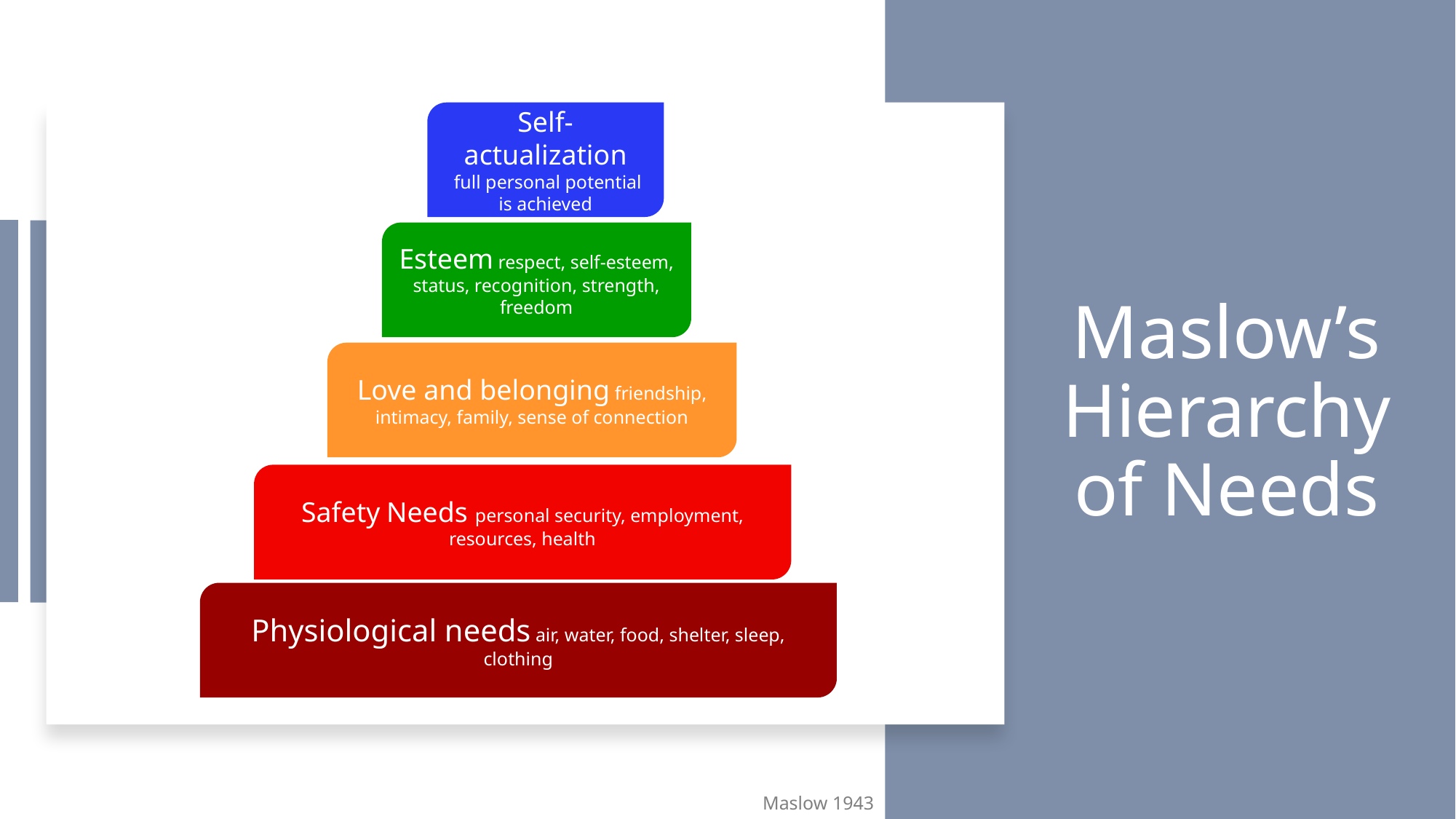

Self-actualization
 full personal potential is achieved
Esteem respect, self-esteem, status, recognition, strength, freedom
Love and belonging friendship, intimacy, family, sense of connection
Safety Needs personal security, employment, resources, health
Physiological needs air, water, food, shelter, sleep, clothing
# Maslow’s Hierarchy of Needs
Maslow 1943

## Slide 14
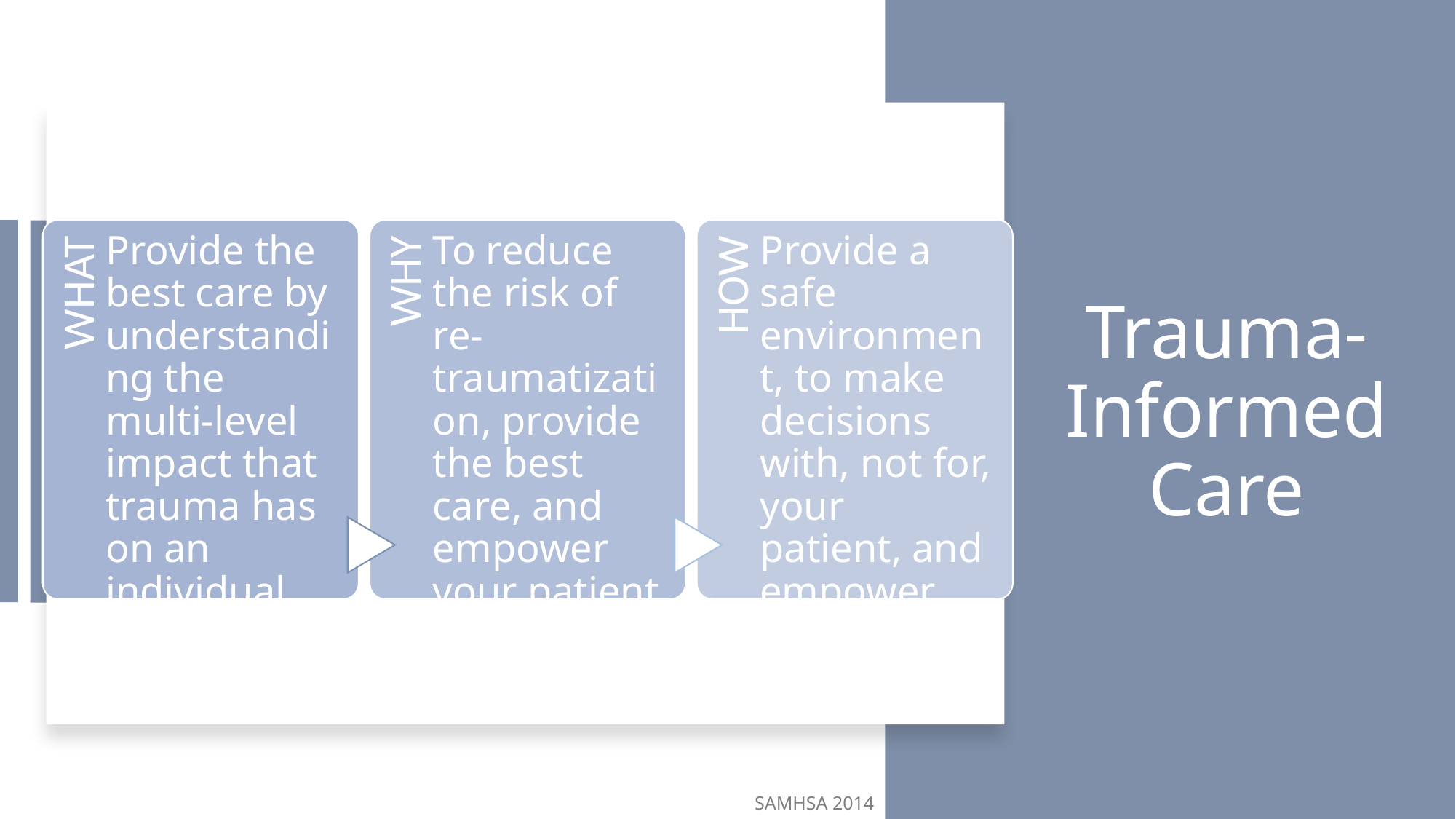

Provide the best care by understanding the multi-level impact that trauma has on an individual
WHAT
To reduce the risk of re-traumatization, provide the best care, and empower your patient
WHY
Provide a safe environment, to make decisions with, not for, your patient, and empower your patient
HOW
Trauma-Informed Care
SAMHSA 2014

## Slide 15
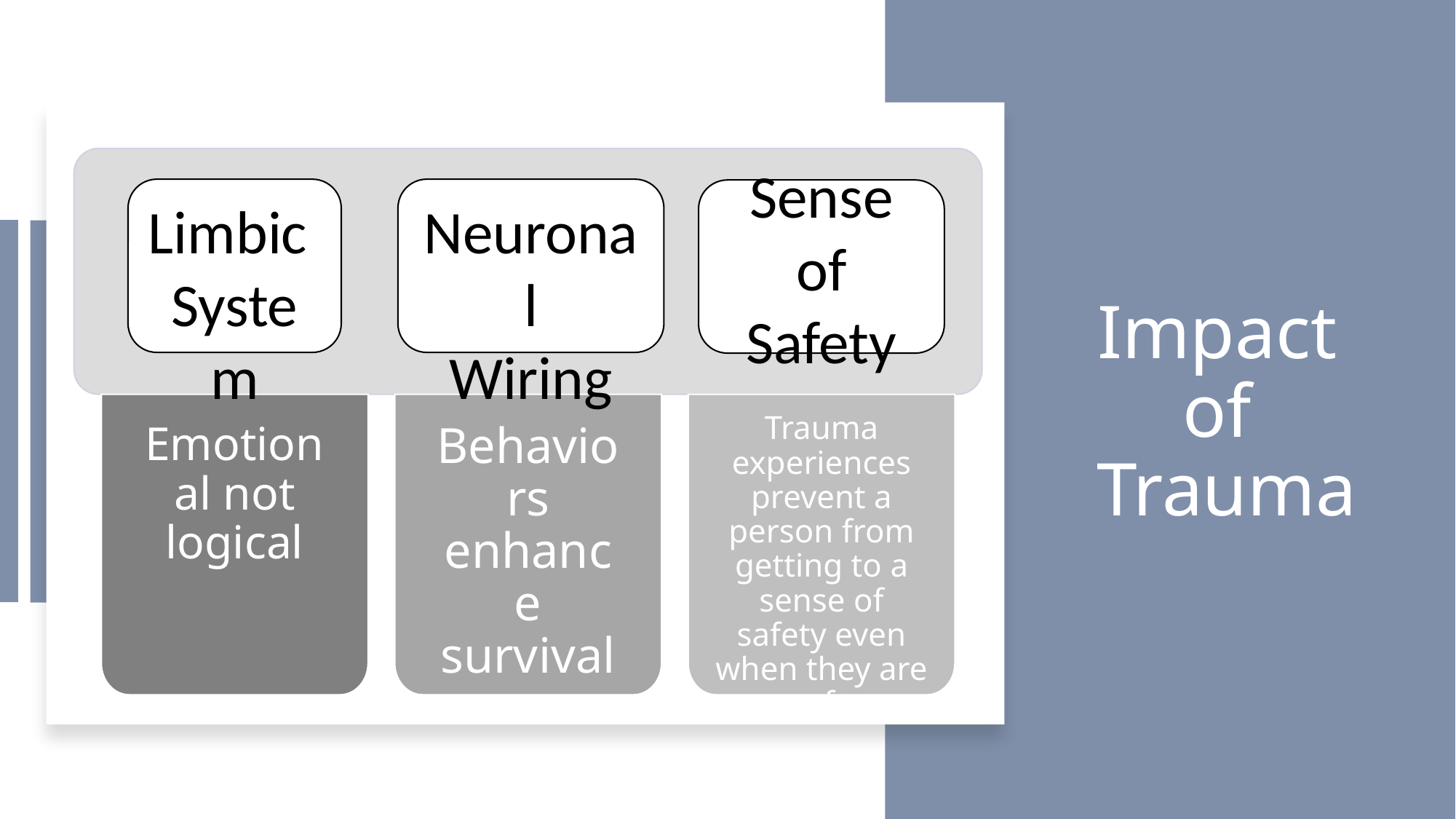

Behaviors enhance survival
Emotional not logical
Trauma experiences prevent a person from getting to a sense of safety even when they are safe
Neuronal
Wiring
Sense of
Safety
Limbic
System
# Impact of Trauma

## Slide 16
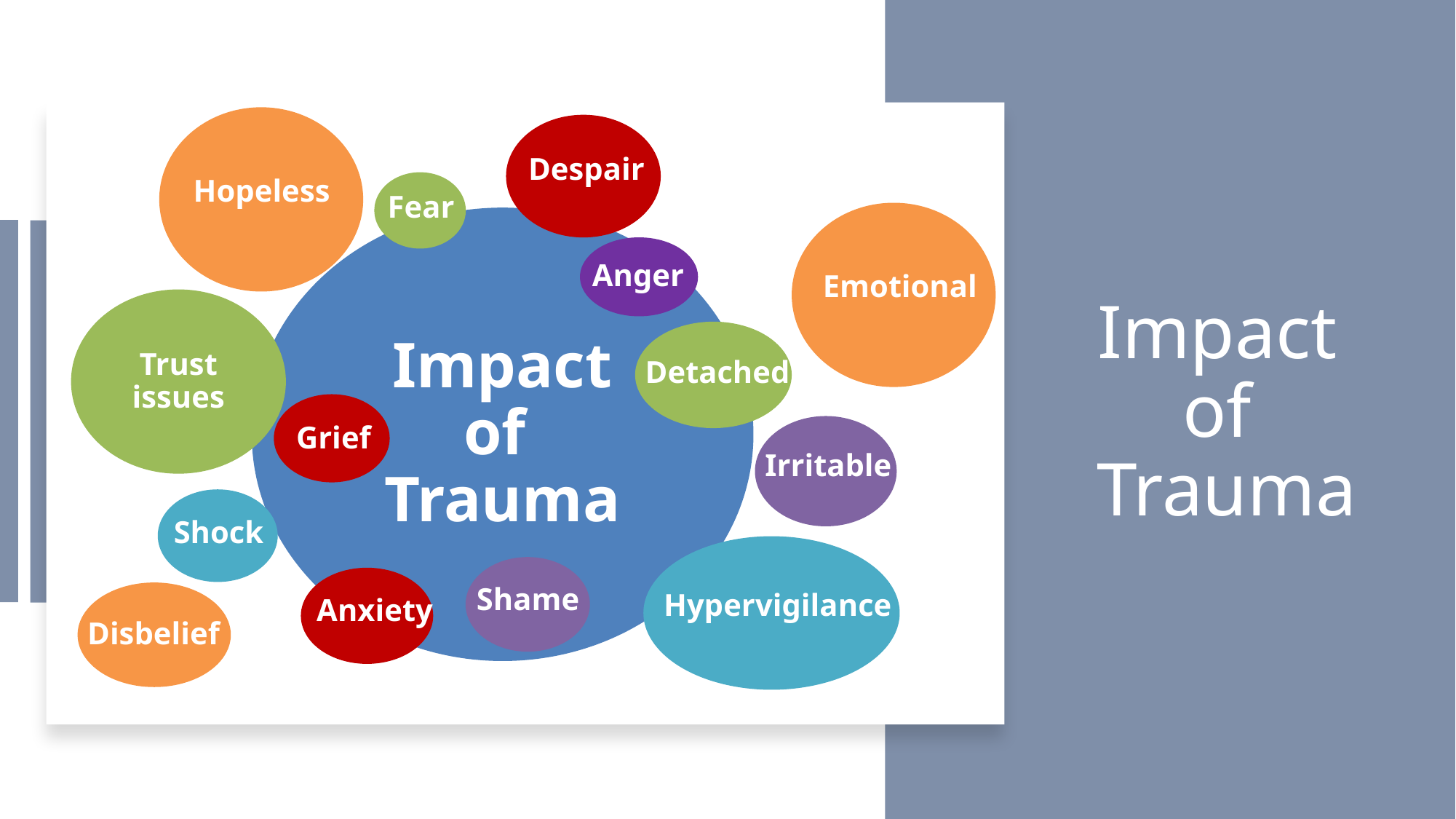

Hopeless
Despair
Fear
Emotional
Impact of Trauma
Anger
Trust issues
Detached
Grief
Irritable
Shock
Hypervigilance
Shame
Anxiety
Disbelief
# Impact of Trauma

## Slide 17
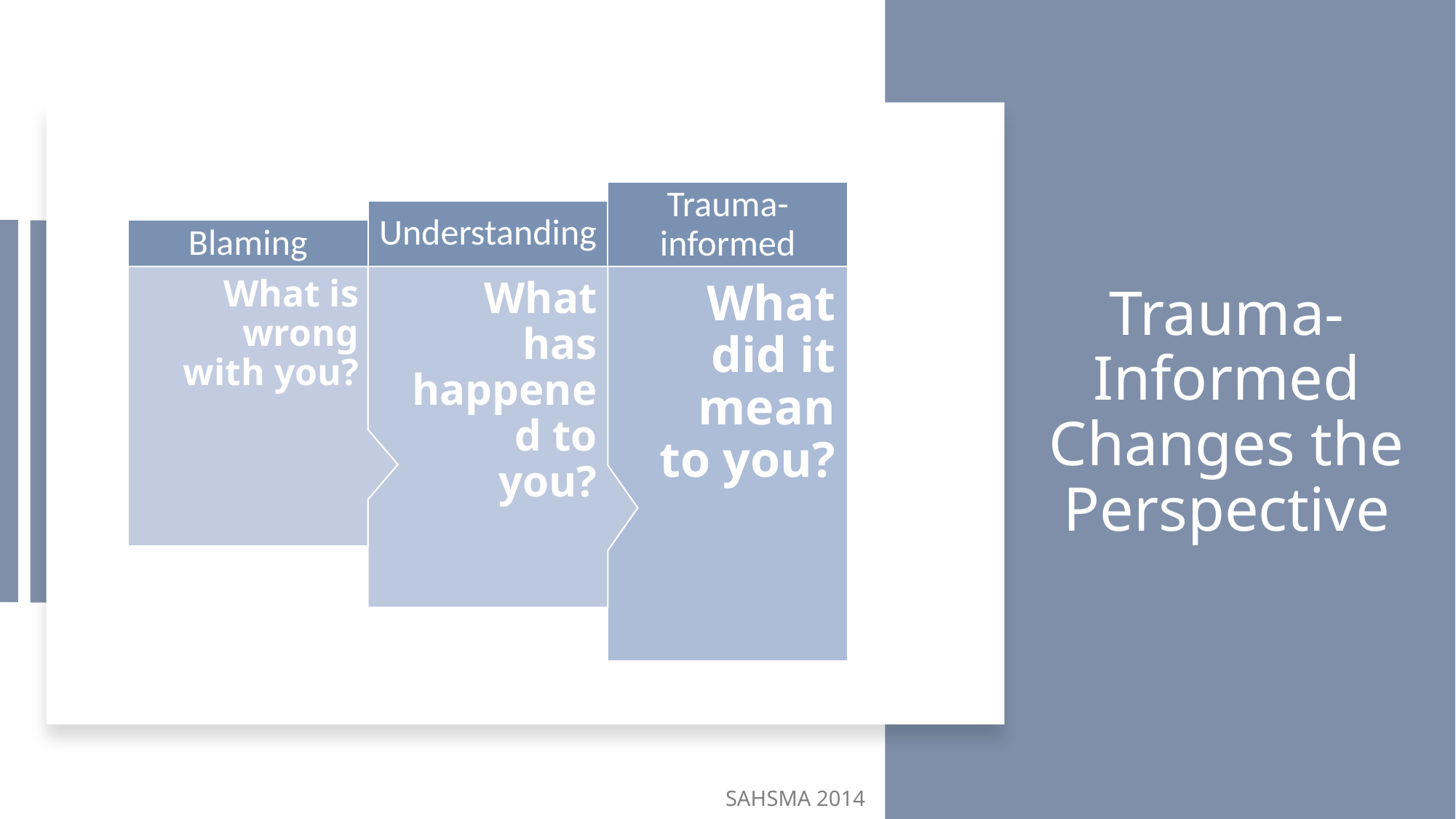

Trauma-informed
Understanding
Blaming
# Trauma-Informed Changes the Perspective
What has happened to you?
What is wrong with you?
What did it mean to you?
SAHSMA 2014

## Slide 18
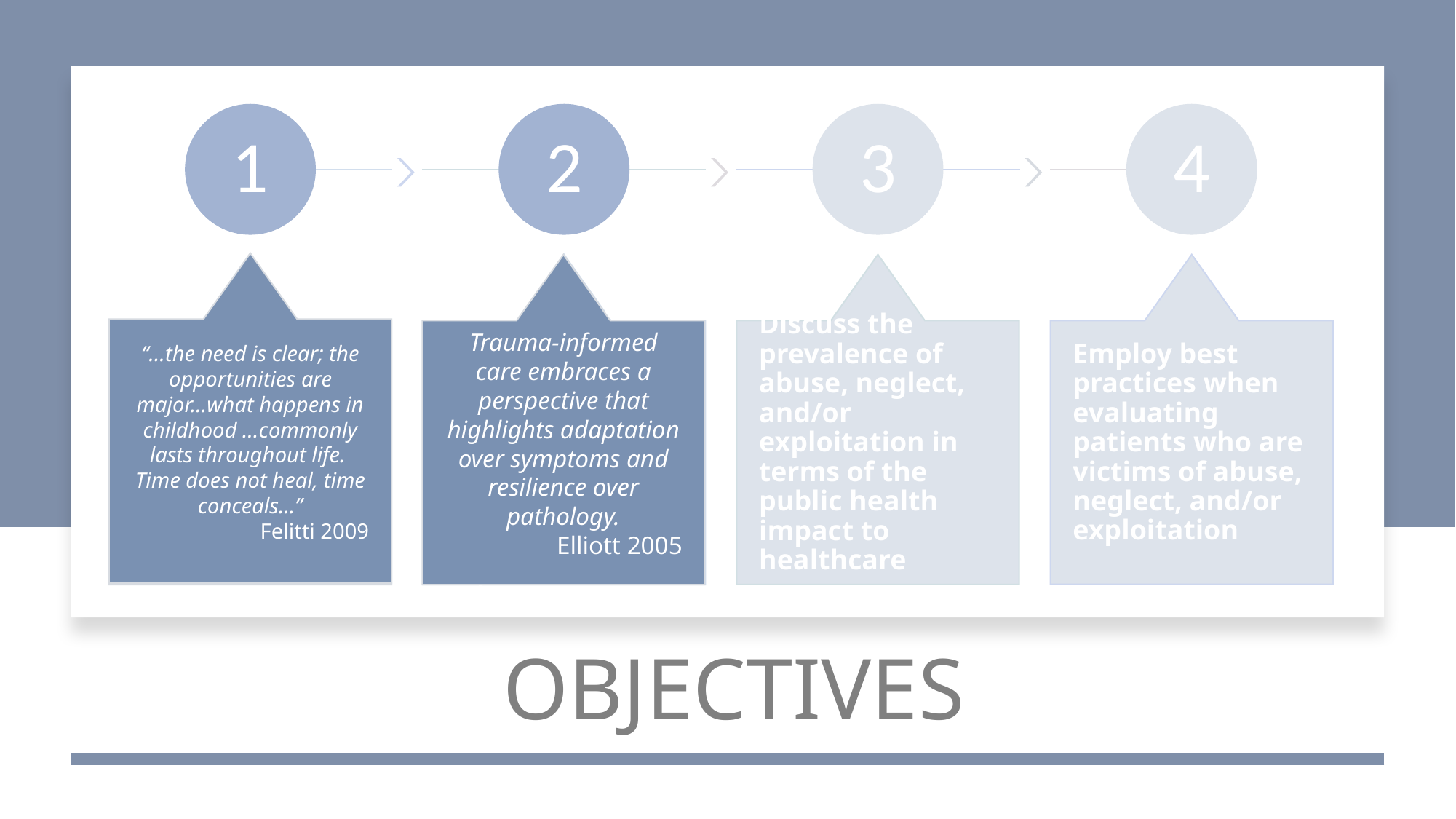

2
Utilize a trauma-informed approach when interacting with patients to reduce or mitigate the consequences of these adverse experiences
3
Discuss the prevalence of abuse, neglect, and/or exploitation in terms of the public health impact to healthcare
4
Employ best practices when evaluating patients who are victims of abuse, neglect, and/or exploitation
1
Explain the link between childhood adversity and risk for poor health across the lifespan
“…the need is clear; the opportunities are major…what happens in childhood …commonly lasts throughout life. Time does not heal, time conceals...”
Felitti 2009
Trauma-informed care embraces a perspective that highlights adaptation over symptoms and resilience over pathology.
Elliott 2005
OBJECTIVES

## Slide 19
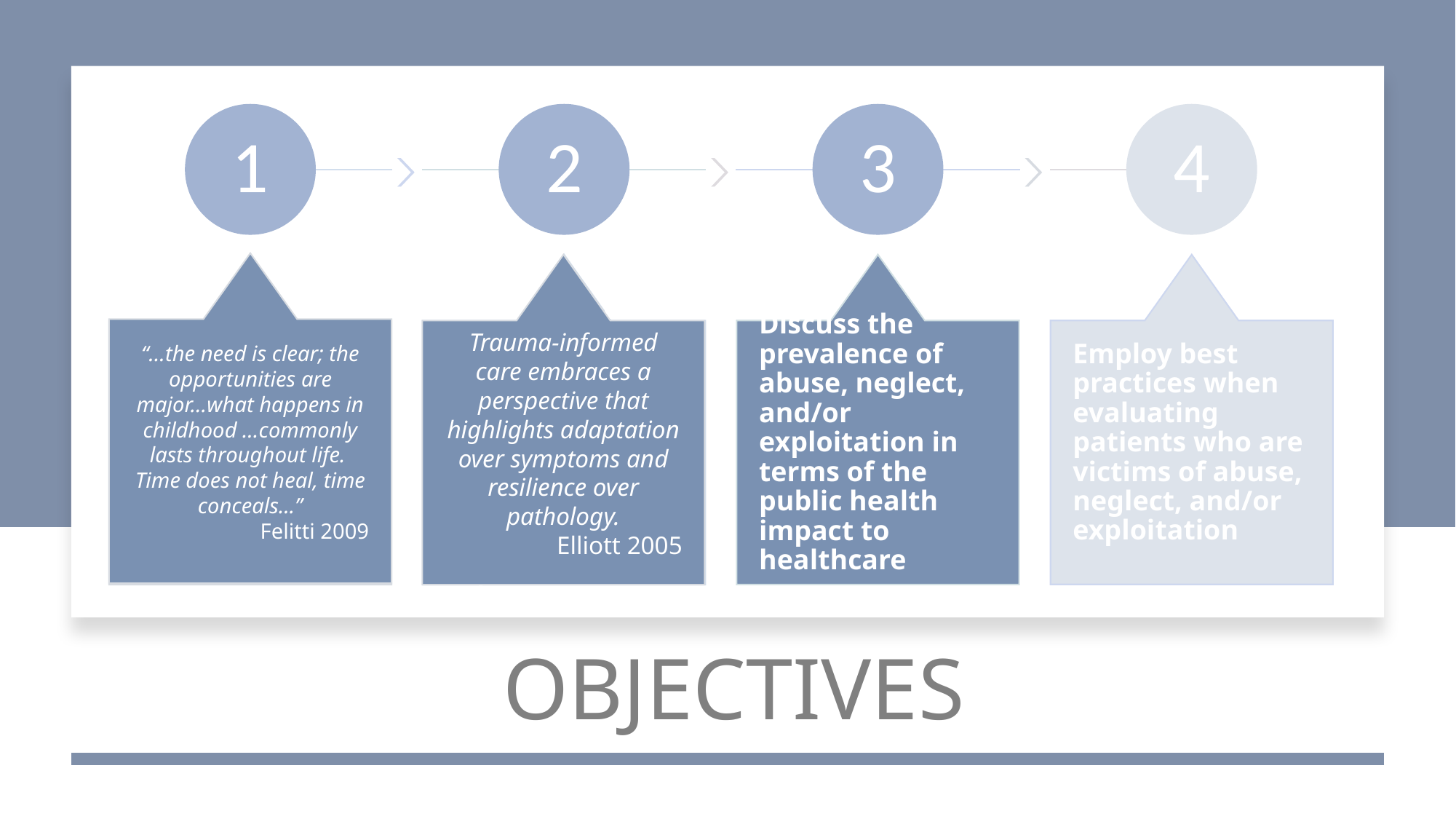

2
Utilize a trauma-informed approach when interacting with patients to reduce or mitigate the consequences of these adverse experiences
3
Discuss the prevalence of abuse, neglect, and/or exploitation in terms of the public health impact to healthcare
4
Employ best practices when evaluating patients who are victims of abuse, neglect, and/or exploitation
1
Explain the link between childhood adversity and risk for poor health across the lifespan
“…the need is clear; the opportunities are major…what happens in childhood …commonly lasts throughout life. Time does not heal, time conceals...”
Felitti 2009
Trauma-informed care embraces a perspective that highlights adaptation over symptoms and resilience over pathology.
Elliott 2005
OBJECTIVES

## Slide 20
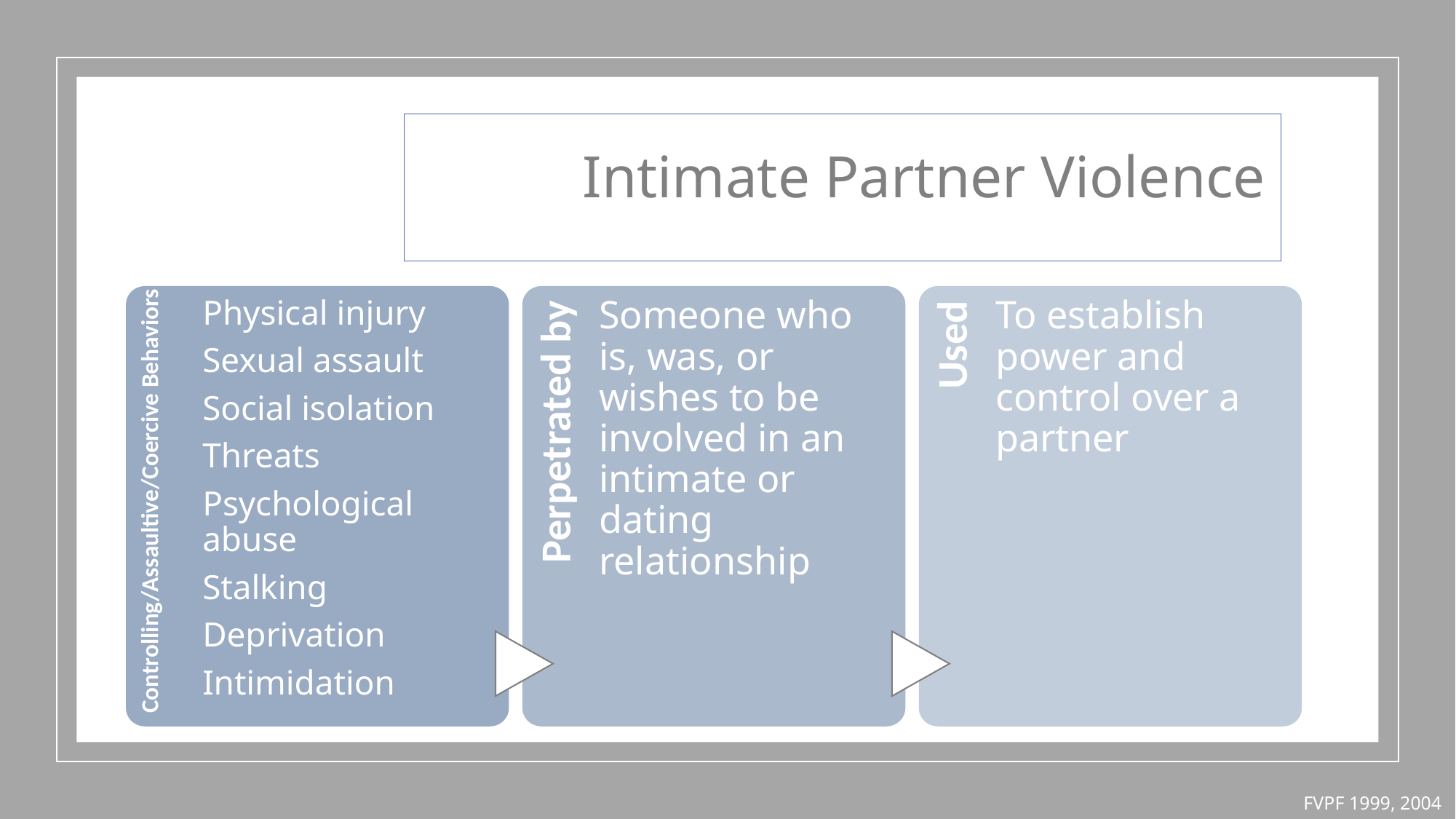

Intimate Partner Violence
Physical injury
Sexual assault
Social isolation
Threats
Psychological abuse
Stalking
Deprivation
Intimidation
Controlling/Assaultive/Coercive Behaviors
Someone who is, was, or wishes to be involved in an intimate or dating relationship
Perpetrated by
To establish power and control over a partner
Used
FVPF 1999, 2004

## Slide 21
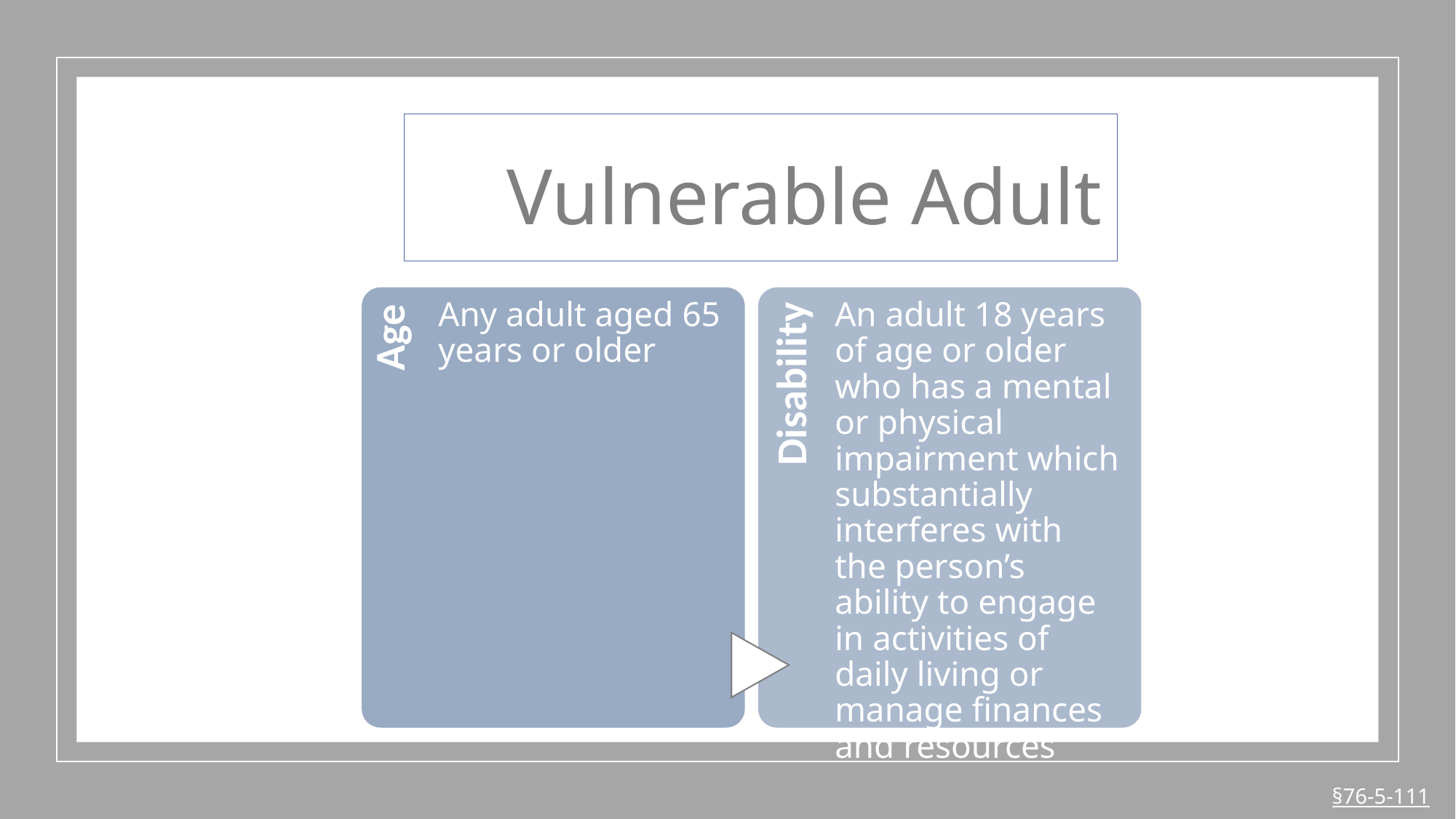

Vulnerable Adult
Any adult aged 65 years or older
Age
An adult 18 years of age or older who has a mental or physical impairment which substantially interferes with the person’s ability to engage in activities of daily living or manage finances and resources
Disability
§76-5-111

## Slide 22
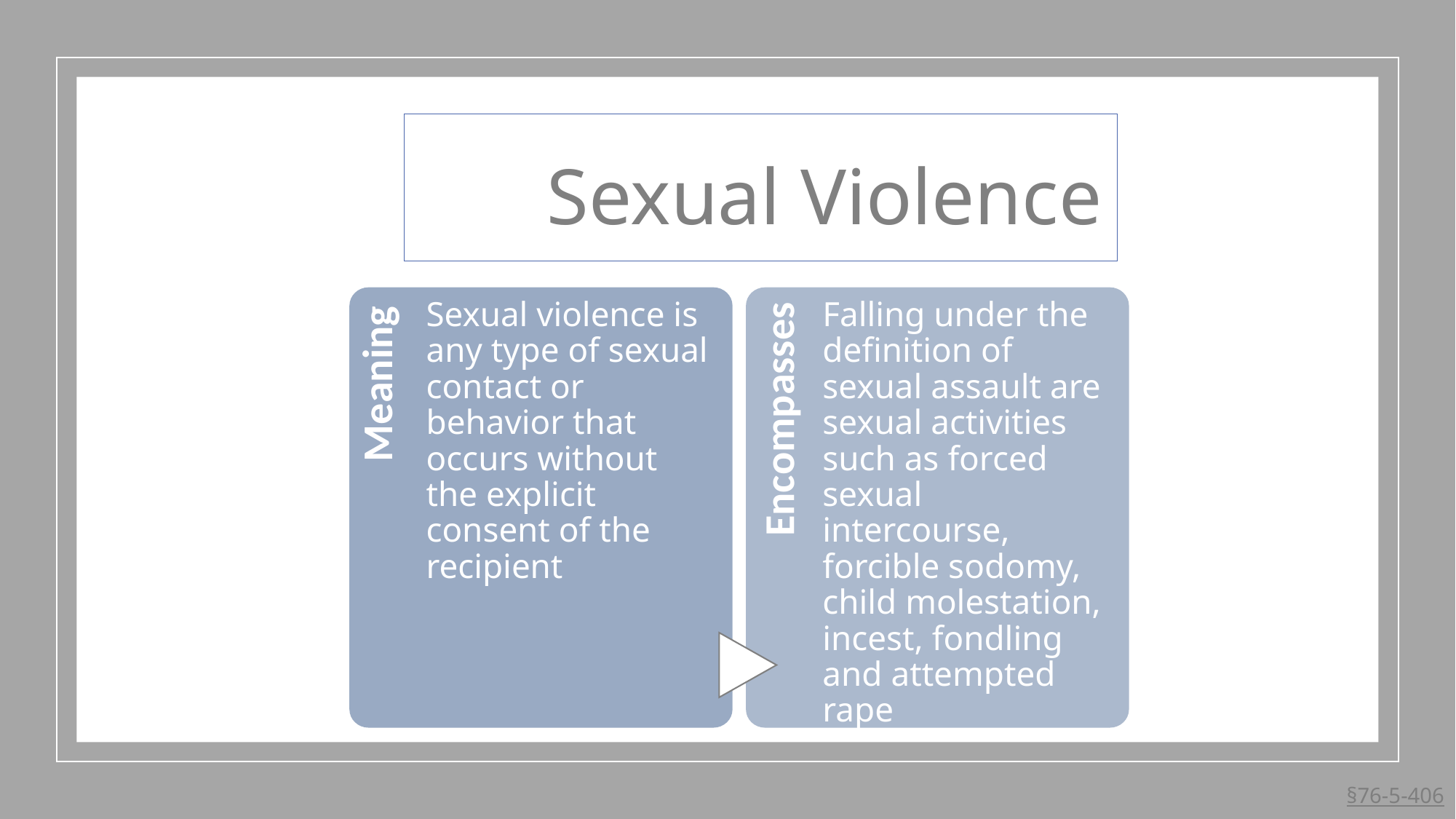

Sexual Violence
Sexual violence is any type of sexual contact or behavior that occurs without the explicit consent of the recipient
Meaning
Falling under the definition of sexual assault are sexual activities such as forced sexual intercourse, forcible sodomy, child molestation, incest, fondling and attempted rape
Encompasses
§76-5-406

## Slide 23
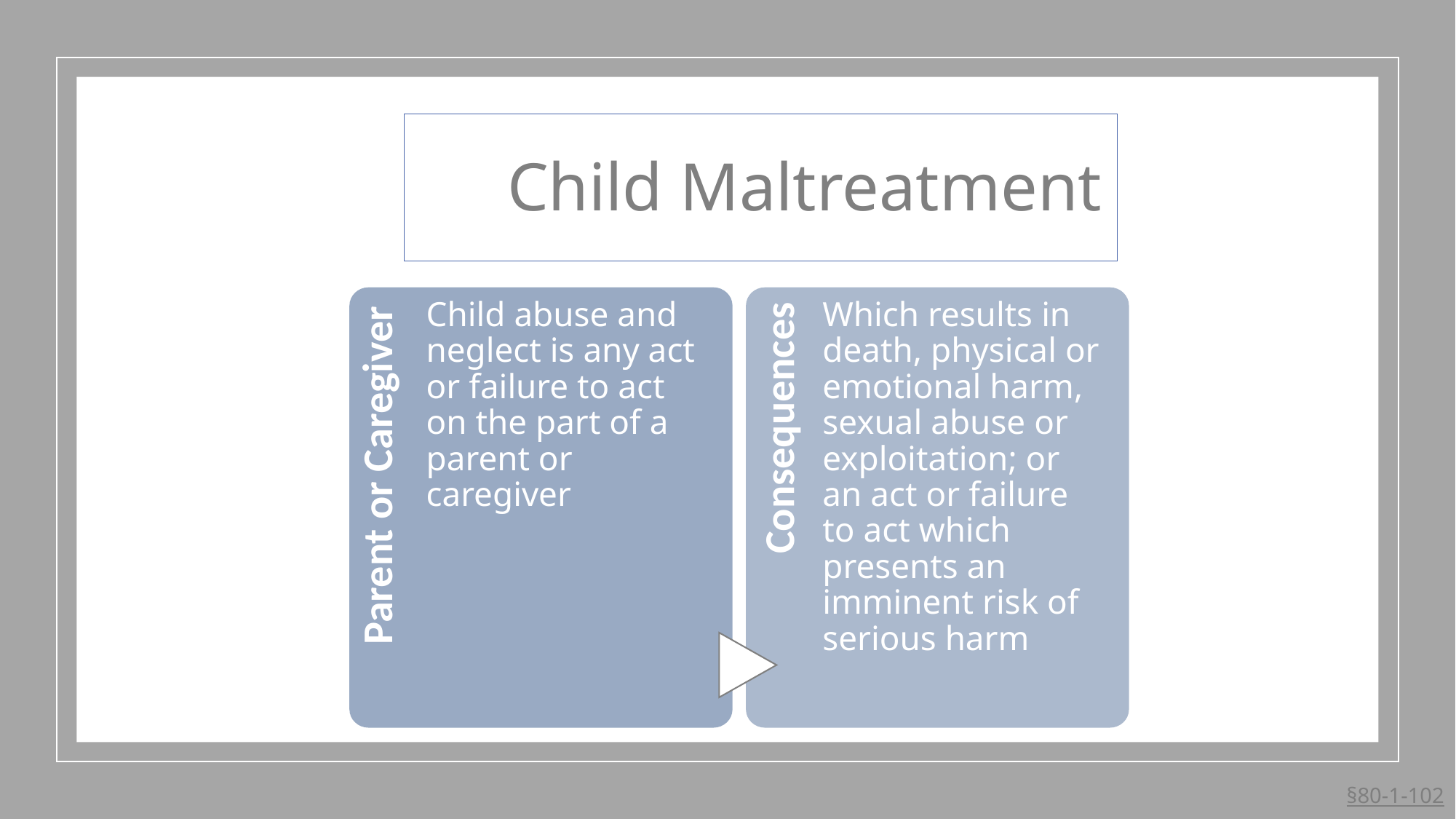

Child Maltreatment
Child abuse and neglect is any act or failure to act on the part of a parent or caregiver
Parent or Caregiver
Which results in death, physical or emotional harm, sexual abuse or exploitation; or an act or failure to act which presents an imminent risk of serious harm
Consequences
§80-1-102

## Slide 24
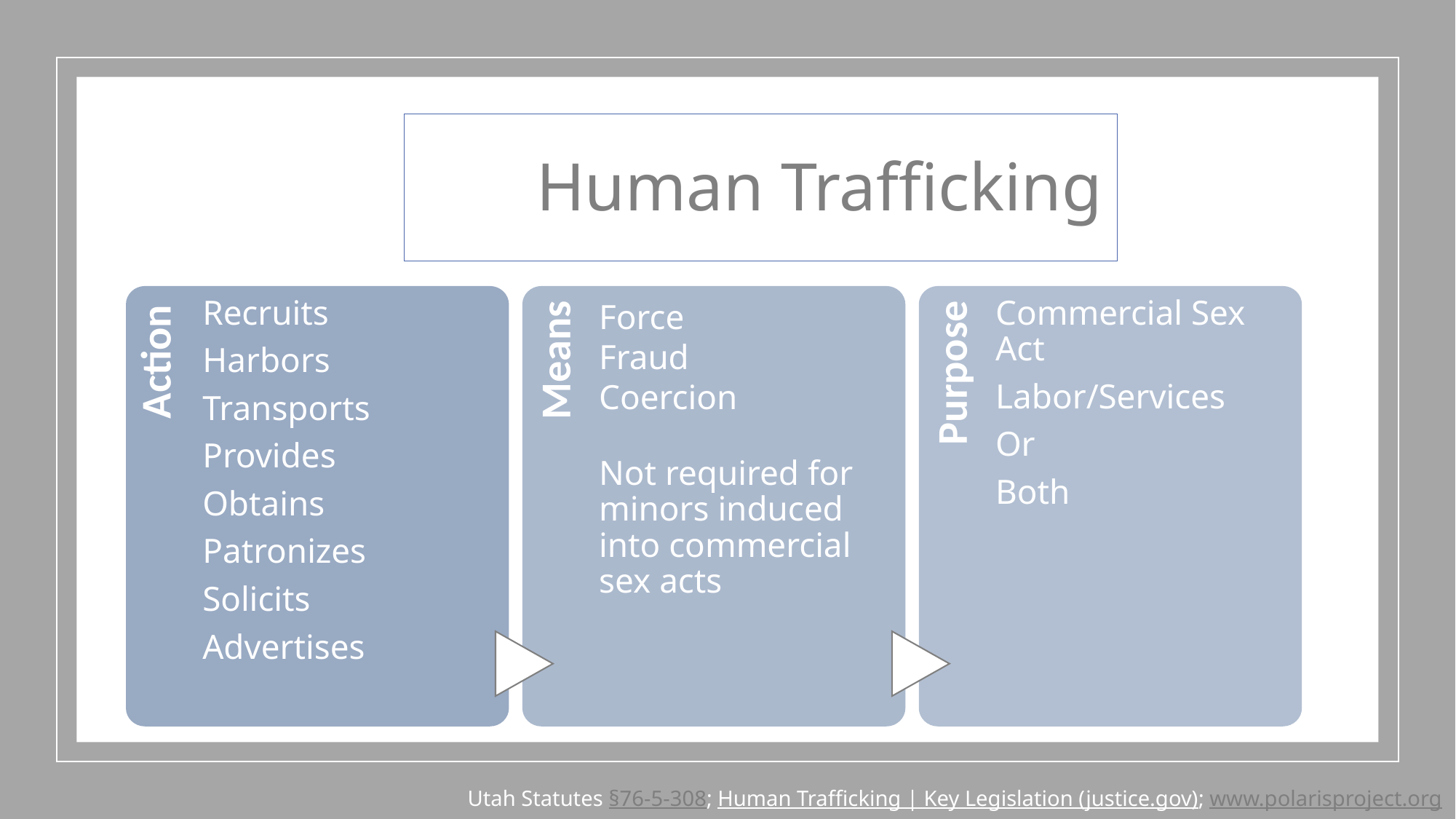

Human Trafficking
Force
Fraud
Coercion
Not required for minors induced into commercial sex acts
Means
Commercial Sex Act
Labor/Services
Or
Both
Purpose
Recruits
Harbors
Transports
Provides
Obtains
Patronizes
Solicits
Advertises
Action
Utah Statutes §76-5-308; Human Trafficking | Key Legislation (justice.gov); www.polarisproject.org

## Slide 25
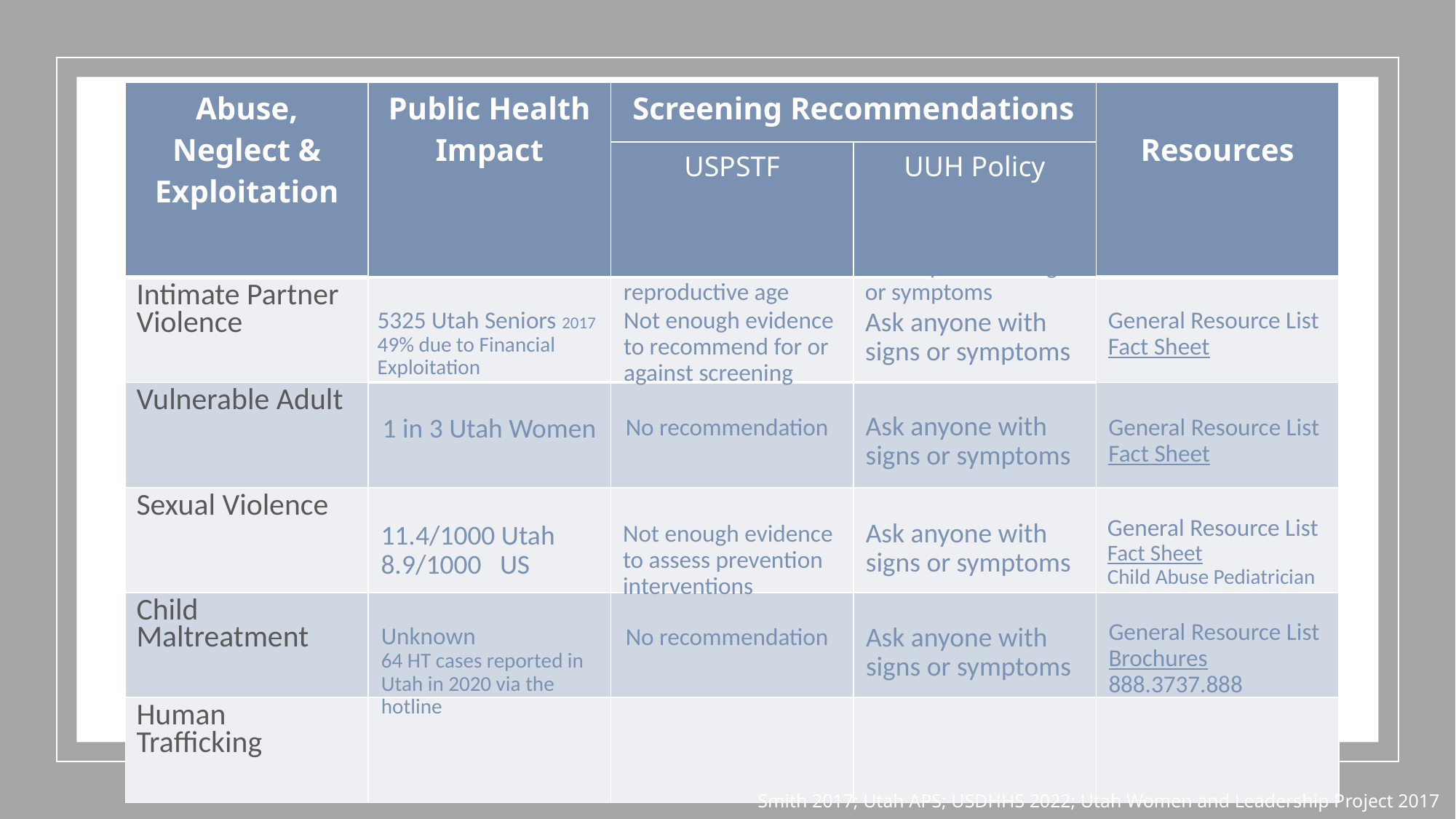

| Abuse, Neglect & Exploitation | Public Health Impact | Screening Recommendations | | Resources |
| --- | --- | --- | --- | --- |
| | | USPSTF | UUH Policy | |
| Intimate Partner Violence | | | | |
| Vulnerable Adult | | | | |
| Sexual Violence | | | | |
| Child Maltreatment | | | | |
| Human Trafficking | | | | |
General Resource List
800-897-5465
Fact Sheet
1 in 3 Utah Women
1 in 5 Utah Men
Screen for IPV victimization in women of reproductive age
Screen as per USPSTF AND
Ask anyone with signs or symptoms
5325 Utah Seniors 2017
49% due to Financial Exploitation
Not enough evidence to recommend for or against screening
Ask anyone with signs or symptoms
General Resource List
Fact Sheet
Ask anyone with signs or symptoms
1 in 3 Utah Women
No recommendation
General Resource List
Fact Sheet
General Resource List
Fact Sheet
Child Abuse Pediatrician
Ask anyone with signs or symptoms
11.4/1000 Utah
8.9/1000 US
Not enough evidence to assess prevention interventions
General Resource List
Brochures
888.3737.888
Ask anyone with signs or symptoms
Unknown
64 HT cases reported in Utah in 2020 via the hotline
No recommendation
Smith 2017; Utah APS; USDHHS 2022; Utah Women and Leadership Project 2017

## Slide 26
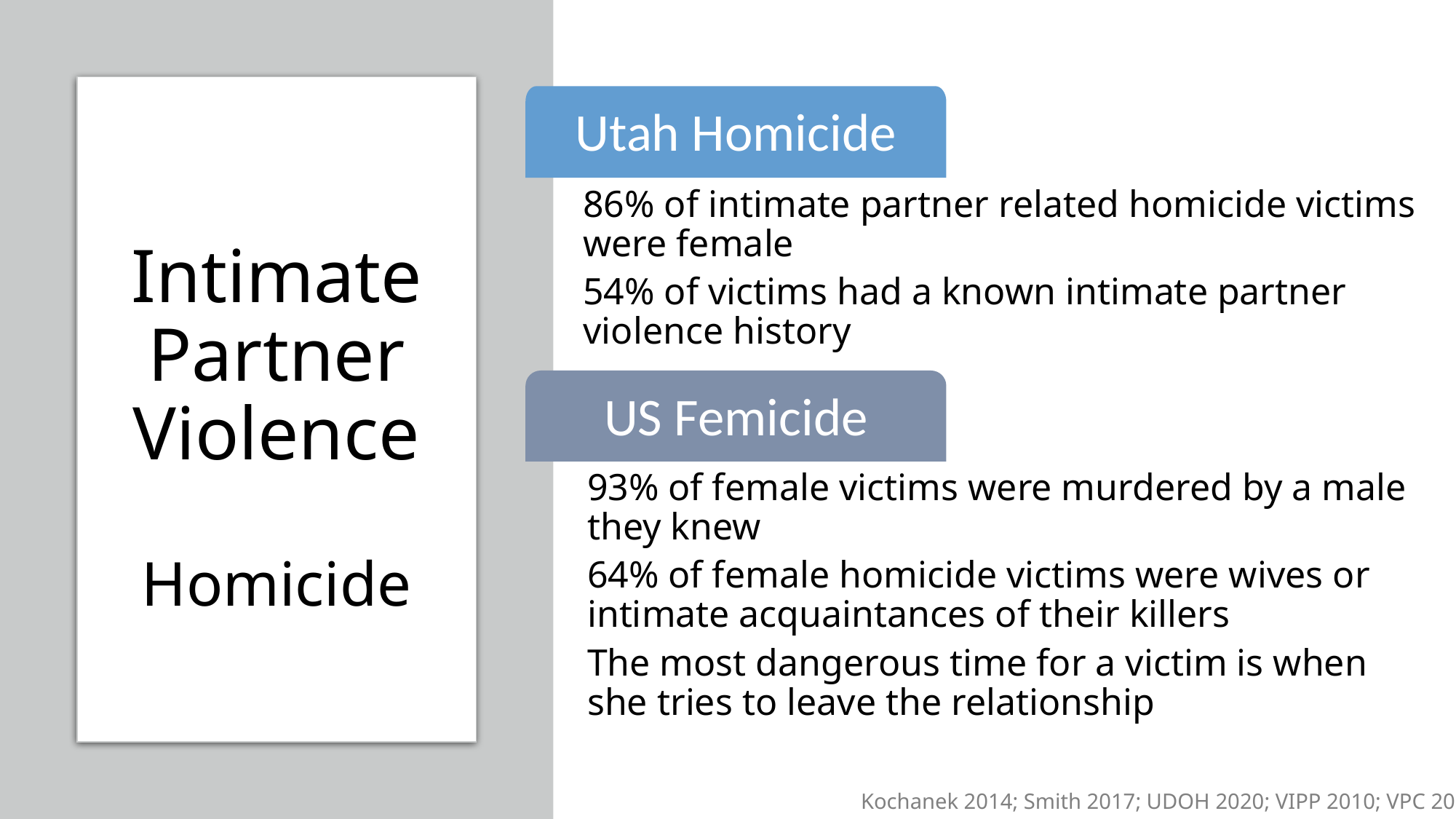

# Intimate Partner Violence Homicide
Utah Homicide
86% of intimate partner related homicide victims were female
54% of victims had a known intimate partner violence history
US Femicide
93% of female victims were murdered by a male they knew
64% of female homicide victims were wives or intimate acquaintances of their killers
The most dangerous time for a victim is when she tries to leave the relationship
Kochanek 2014; Smith 2017; UDOH 2020; VIPP 2010; VPC 2017

## Slide 27
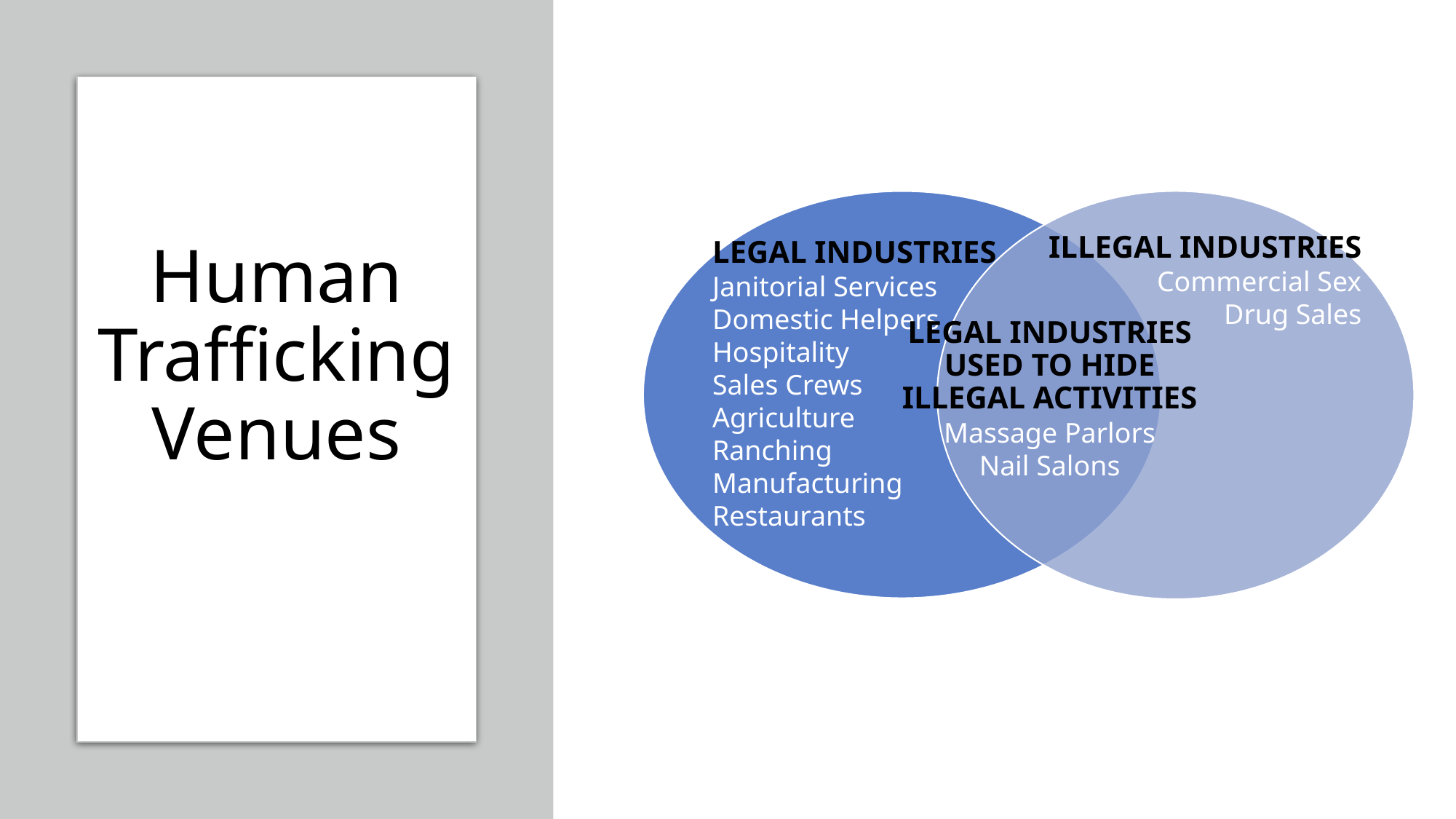

# Human Trafficking Venues
Legal Industries
Janitorial Services
Domestic Helpers
Hospitality
Sales Crews
Agriculture
Ranching
Manufacturing
Restaurants
Illegal Industries
Commercial Sex
Drug Sales
Legal Industries
Used to Hide
Illegal Activities
Massage Parlors
Nail Salons

## Slide 28
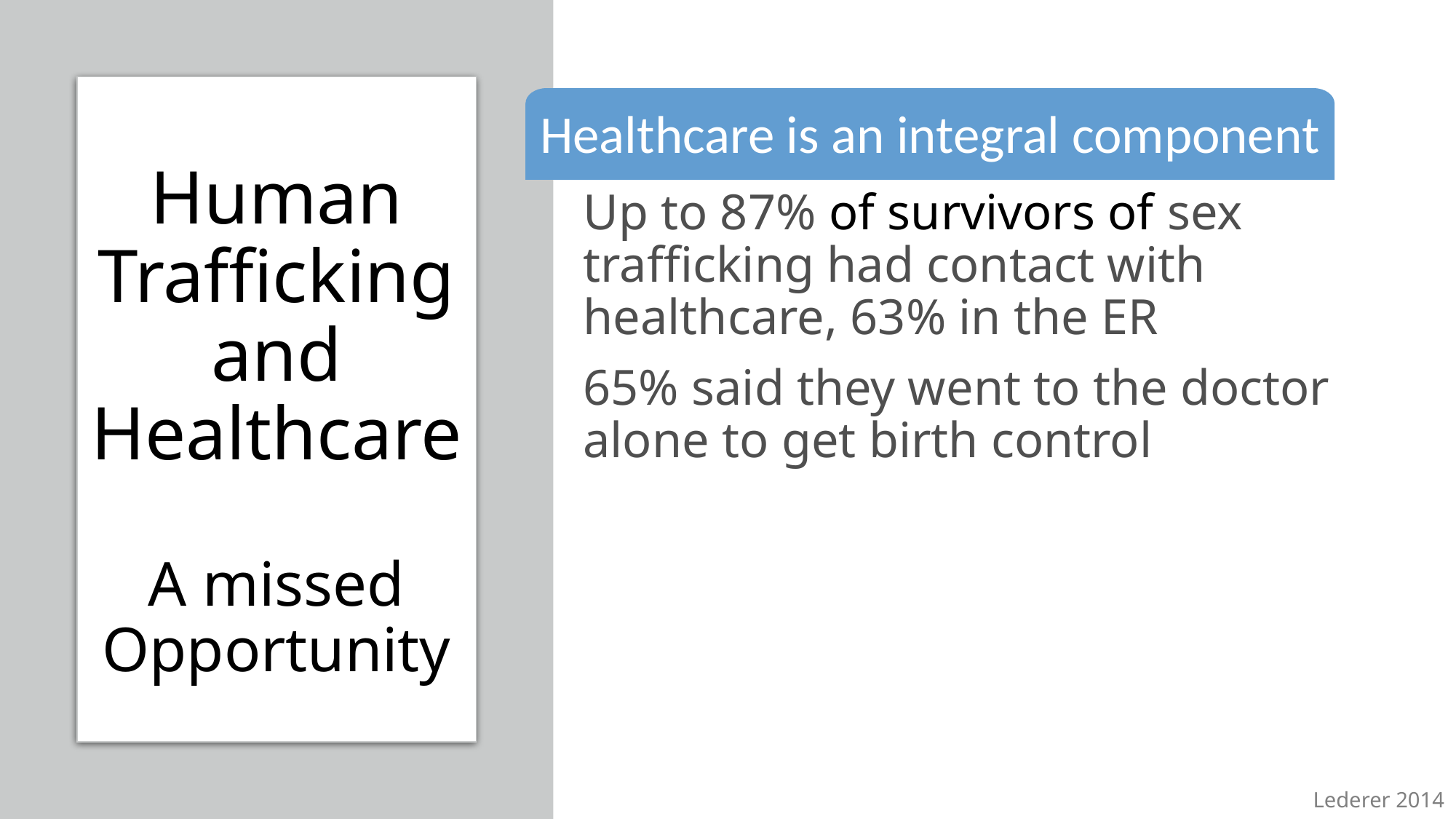

# Human Trafficking and Healthcare A missed Opportunity
Healthcare is an integral component
Up to 87% of survivors of sex trafficking had contact with healthcare, 63% in the ER
65% said they went to the doctor alone to get birth control
Lederer 2014

## Slide 29
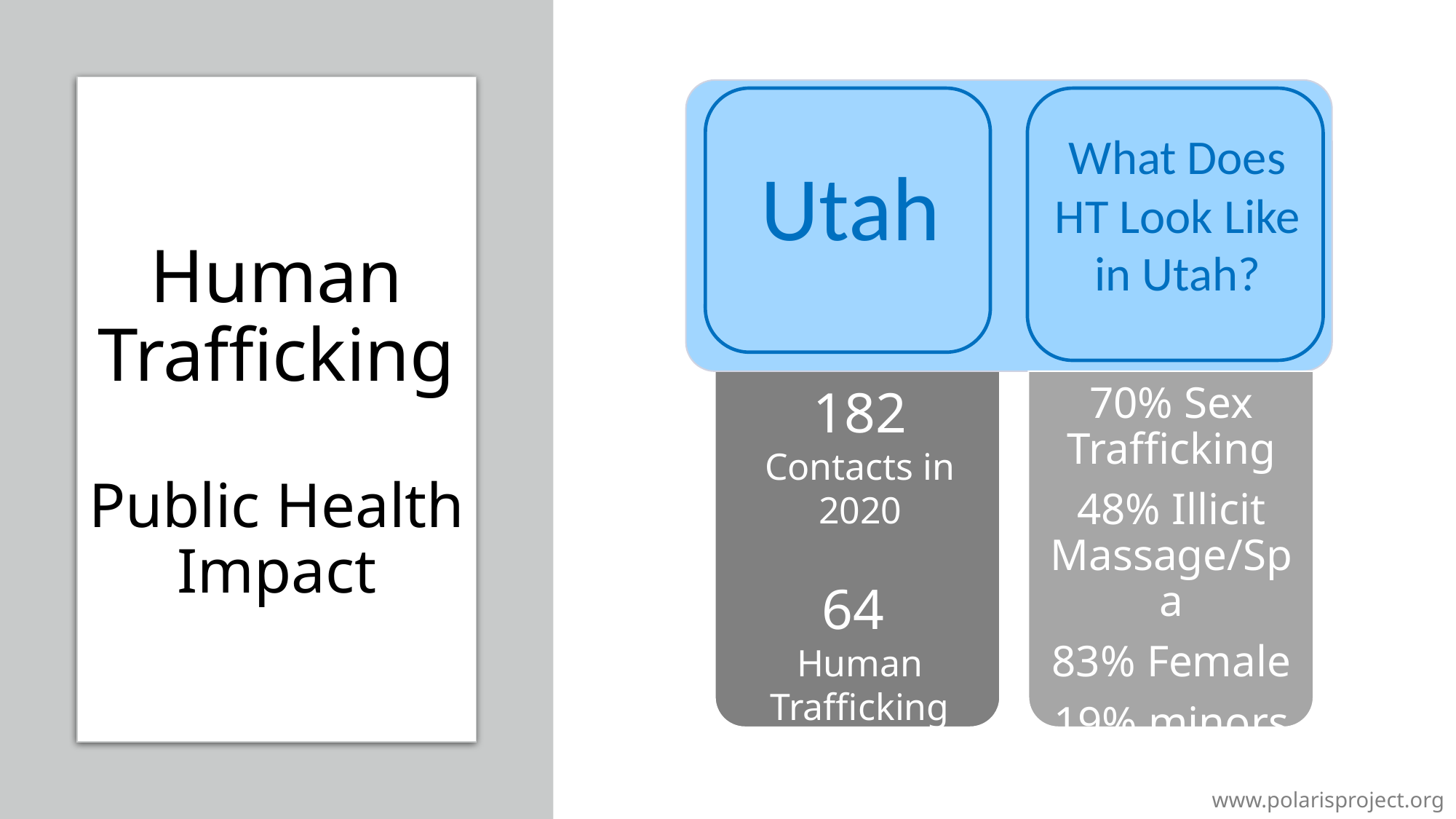

# Human Trafficking Public Health Impact
What Does HT Look Like in Utah?
Utah
70% Sex Trafficking
48% Illicit Massage/Spa
83% Female
19% minors
182
Contacts in 2020
64
Human Trafficking Cases Reported
www.polarisproject.org

## Slide 30
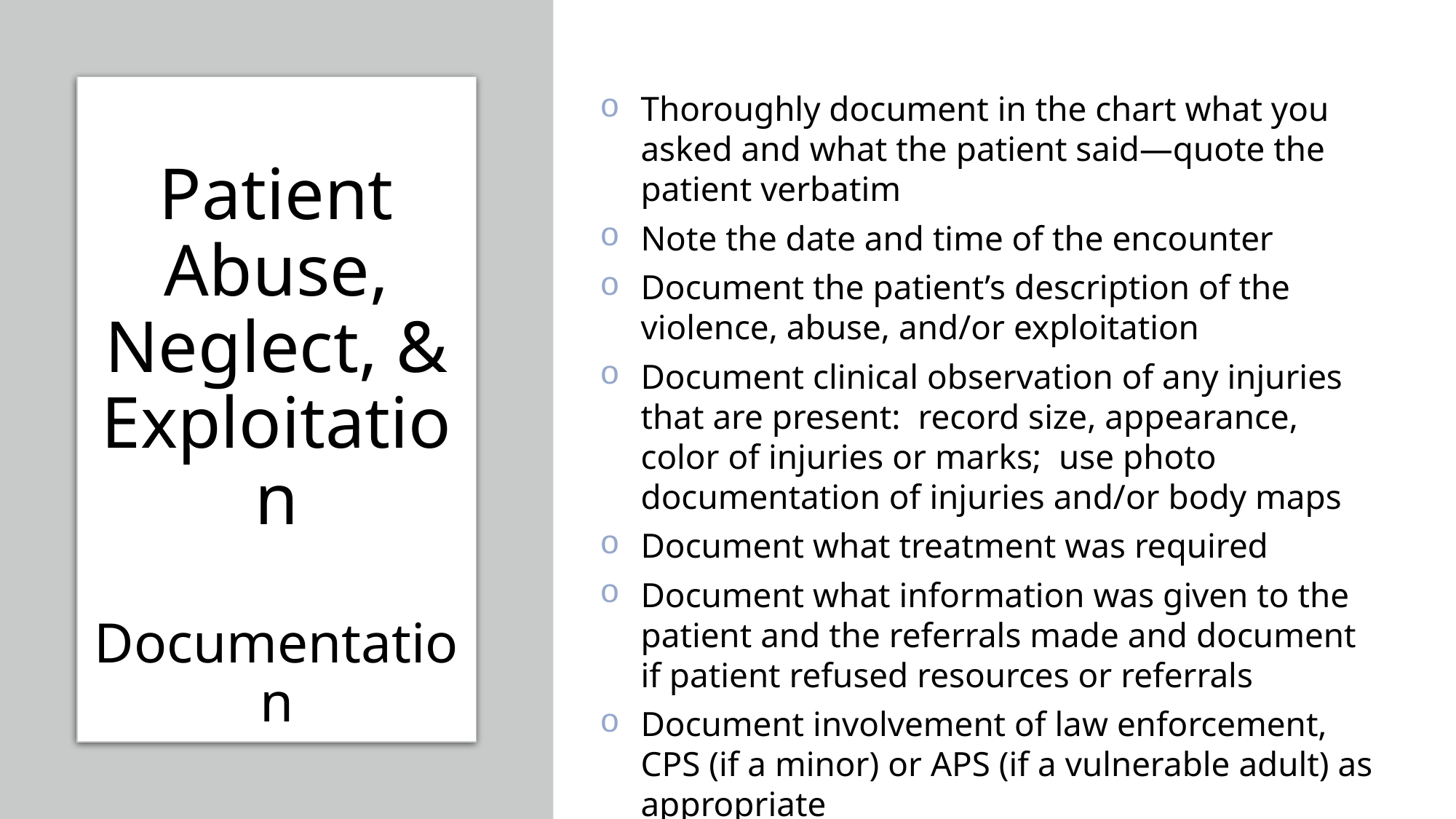

# Patient Abuse, Neglect, & Exploitation Documentation
Thoroughly document in the chart what you asked and what the patient said—quote the patient verbatim
Note the date and time of the encounter
Document the patient’s description of the violence, abuse, and/or exploitation
Document clinical observation of any injuries that are present: record size, appearance, color of injuries or marks; use photo documentation of injuries and/or body maps
Document what treatment was required
Document what information was given to the patient and the referrals made and document if patient refused resources or referrals
Document involvement of law enforcement, CPS (if a minor) or APS (if a vulnerable adult) as appropriate

## Slide 31
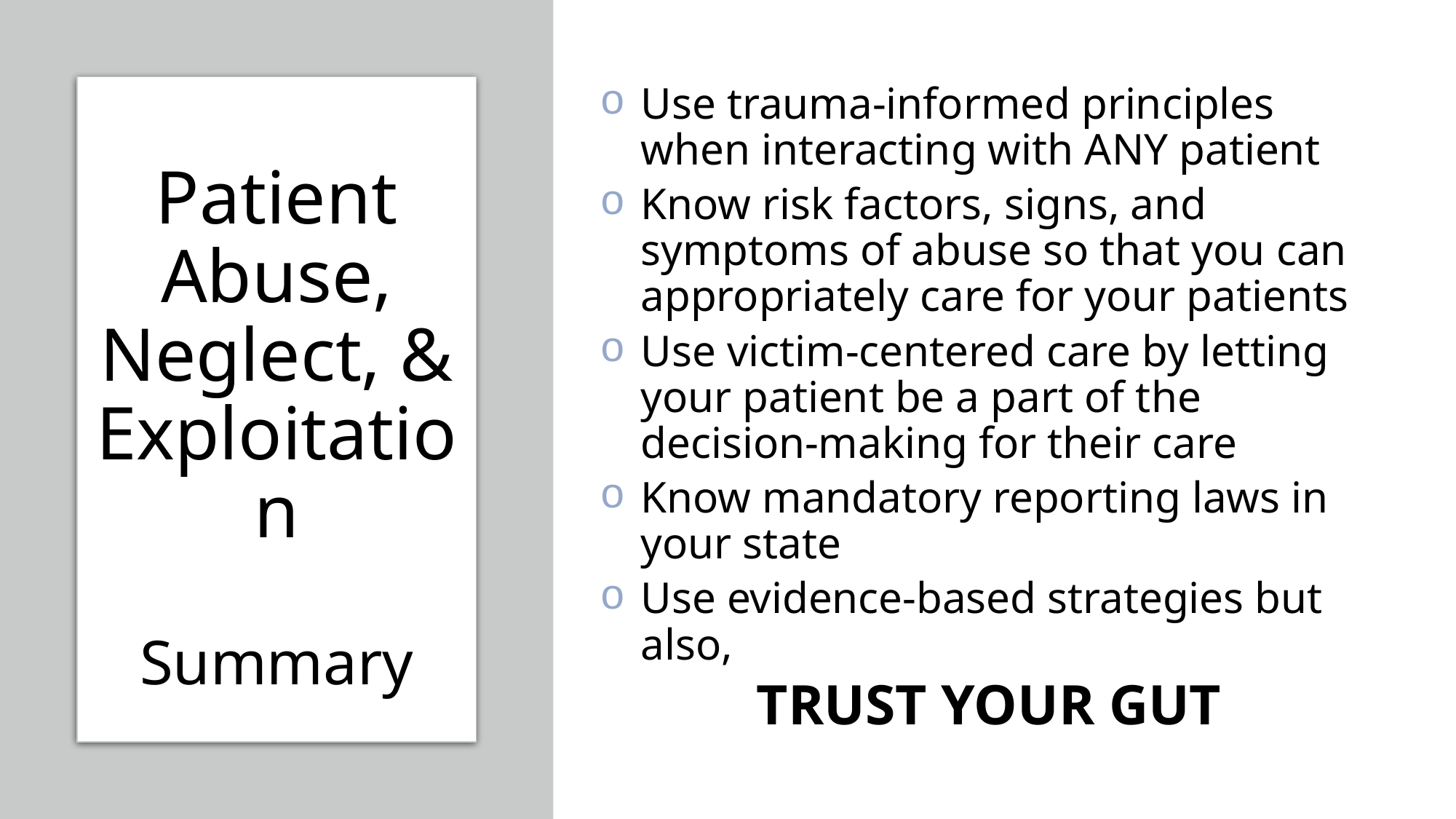

# Patient Abuse, Neglect, & Exploitation Summary
Use trauma-informed principles when interacting with ANY patient
Know risk factors, signs, and symptoms of abuse so that you can appropriately care for your patients
Use victim-centered care by letting your patient be a part of the decision-making for their care
Know mandatory reporting laws in your state
Use evidence-based strategies but also,
Trust Your Gut

## Slide 32
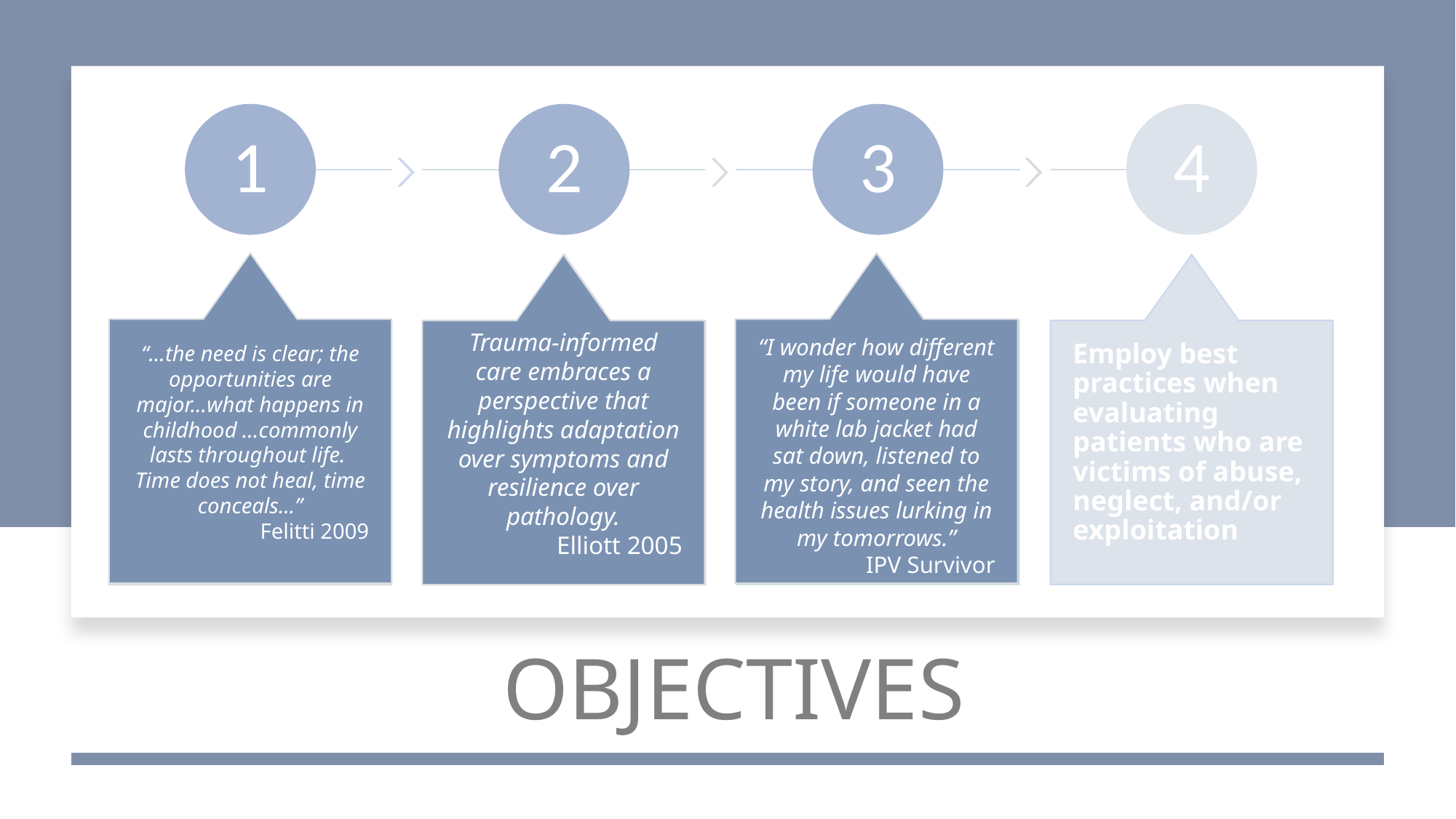

2
Utilize a trauma-informed approach when interacting with patients to reduce or mitigate the consequences of these adverse experiences
3
Discuss the prevalence of abuse, neglect, and/or exploitation in terms of the public health impact to healthcare
4
Employ best practices when evaluating patients who are victims of abuse, neglect, and/or exploitation
1
Explain the link between childhood adversity and risk for poor health across the lifespan
“I wonder how different my life would have been if someone in a white lab jacket had sat down, listened to my story, and seen the health issues lurking in my tomorrows.”
 IPV Survivor
“…the need is clear; the opportunities are major…what happens in childhood …commonly lasts throughout life. Time does not heal, time conceals...”
Felitti 2009
Trauma-informed care embraces a perspective that highlights adaptation over symptoms and resilience over pathology.
Elliott 2005
OBJECTIVES

## Slide 33
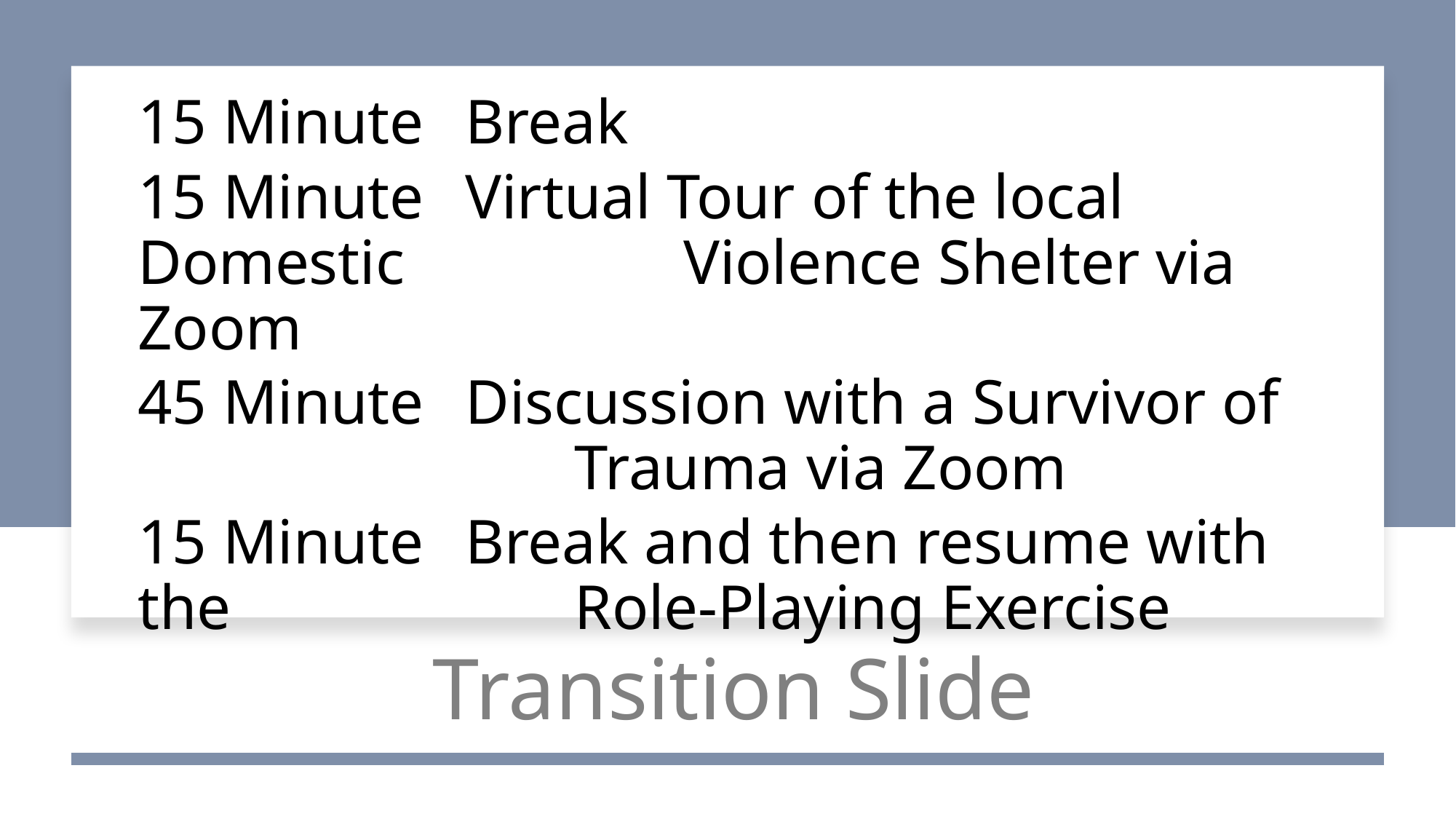

15 Minute 	Break
15 Minute 	Virtual Tour of the local Domestic 			Violence Shelter via Zoom
45 Minute 	Discussion with a Survivor of 				Trauma via Zoom
15 Minute 	Break and then resume with the 			Role-Playing Exercise
Transition Slide

## Slide 34
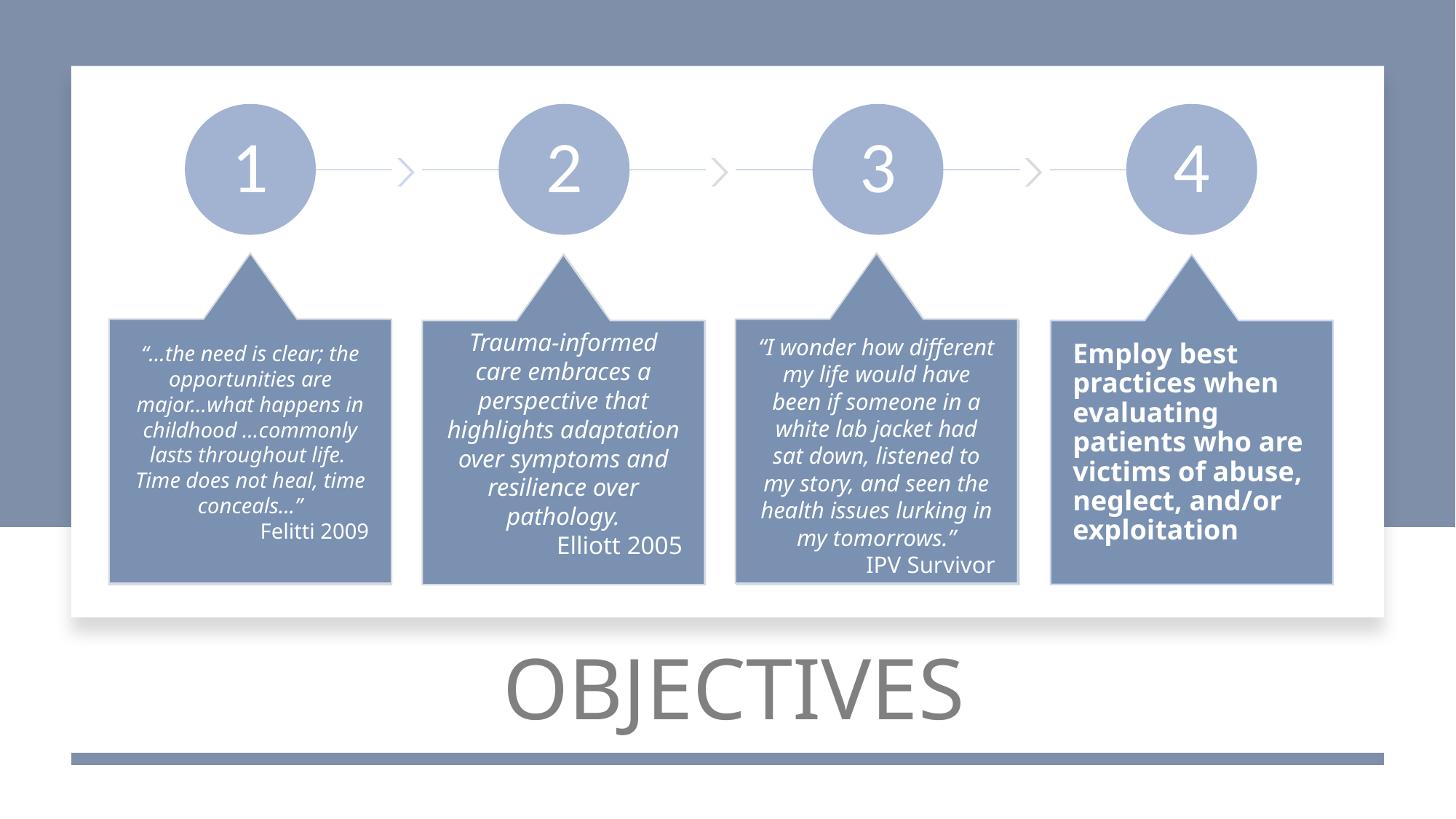

2
Utilize a trauma-informed approach when interacting with patients to reduce or mitigate the consequences of these adverse experiences
3
Discuss the prevalence of abuse, neglect, and/or exploitation in terms of the public health impact to healthcare
4
Employ best practices when evaluating patients who are victims of abuse, neglect, and/or exploitation
1
Explain the link between childhood adversity and risk for poor health across the lifespan
“I wonder how different my life would have been if someone in a white lab jacket had sat down, listened to my story, and seen the health issues lurking in my tomorrows.”
 IPV Survivor
“…the need is clear; the opportunities are major…what happens in childhood …commonly lasts throughout life. Time does not heal, time conceals...”
Felitti 2009
Trauma-informed care embraces a perspective that highlights adaptation over symptoms and resilience over pathology.
Elliott 2005
OBJECTIVES

## Slide 35
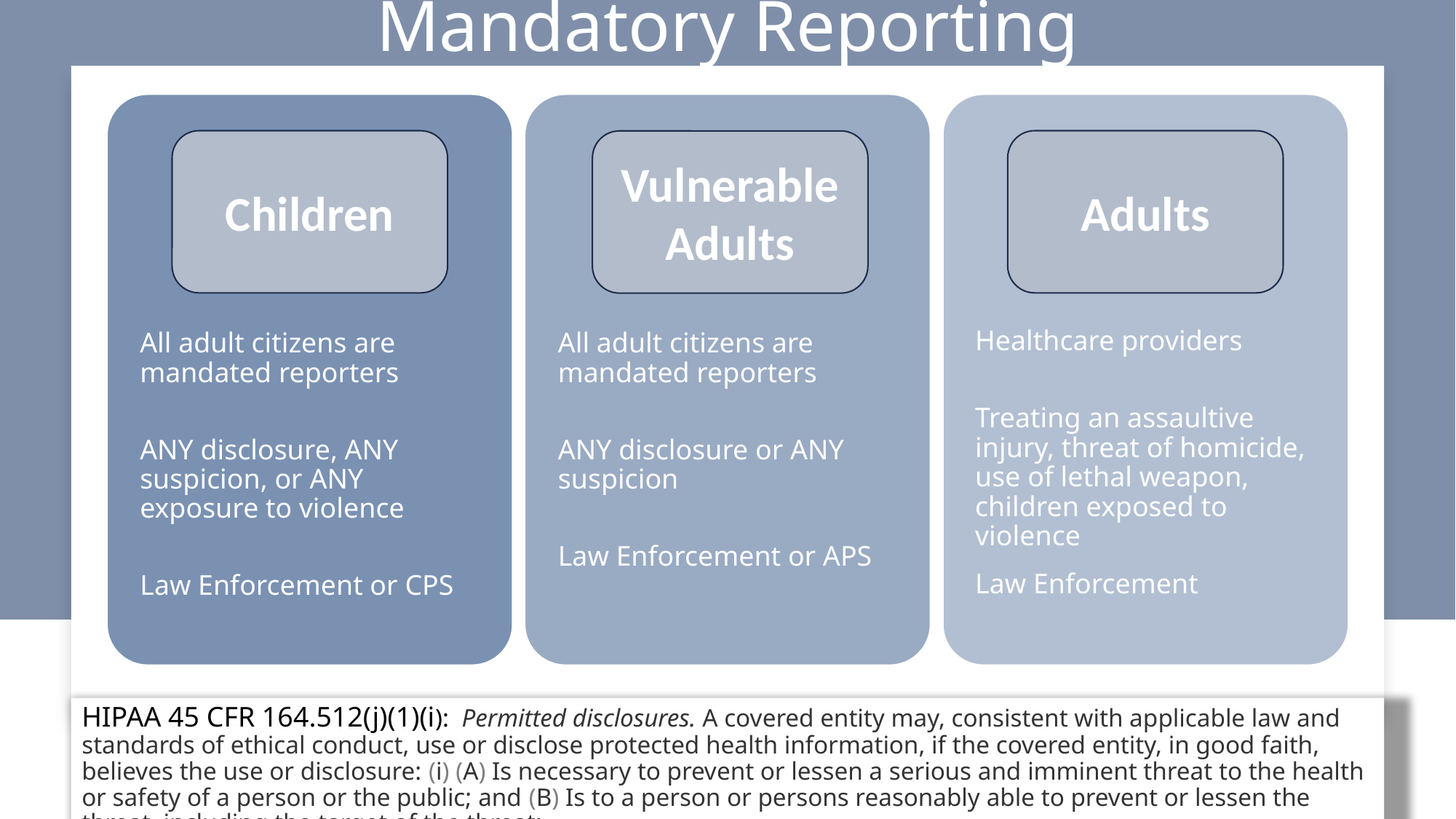

Mandatory Reporting
Adults
Children
Vulnerable Adults
Healthcare providers
Treating an assaultive injury, threat of homicide, use of lethal weapon, children exposed to violence
Law Enforcement
All adult citizens are mandated reporters
ANY disclosure or ANY suspicion
Law Enforcement or APS
All adult citizens are mandated reporters
ANY disclosure, ANY suspicion, or ANY exposure to violence
Law Enforcement or CPS
HIPAA 45 CFR 164.512(j)(1)(i): Permitted disclosures. A covered entity may, consistent with applicable law and standards of ethical conduct, use or disclose protected health information, if the covered entity, in good faith, believes the use or disclosure: (i) (A) Is necessary to prevent or lessen a serious and imminent threat to the health or safety of a person or the public; and (B) Is to a person or persons reasonably able to prevent or lessen the threat, including the target of the threat;…

## Slide 36
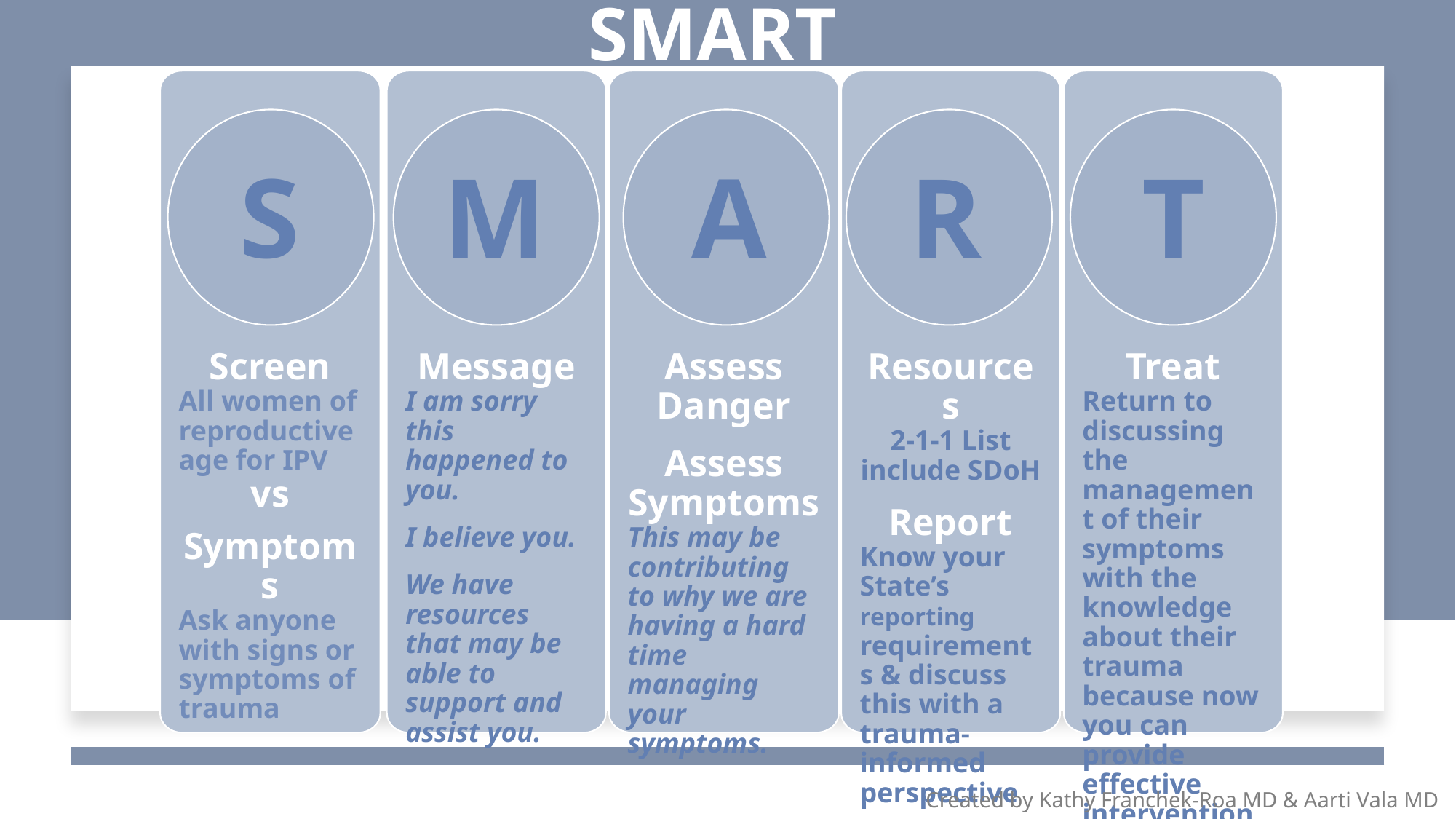

SMART
Screen
All women of reproductive age for IPV
vs
Symptoms
Ask anyone with signs or symptoms of trauma
Message
I am sorry this happened to you.
I believe you.
We have resources that may be able to support and assist you.
Assess Danger
Assess Symptoms
This may be contributing to why we are having a hard time managing your symptoms.
Resources
2-1-1 List include SDoH
Report
Know your State’s reporting requirements & discuss this with a trauma-informed perspective
Treat
Return to discussing the management of their symptoms with the knowledge about their trauma because now you can provide effective interventions
M
A
T
S
R
Created by Kathy Franchek-Roa MD & Aarti Vala MD

## Slide 37
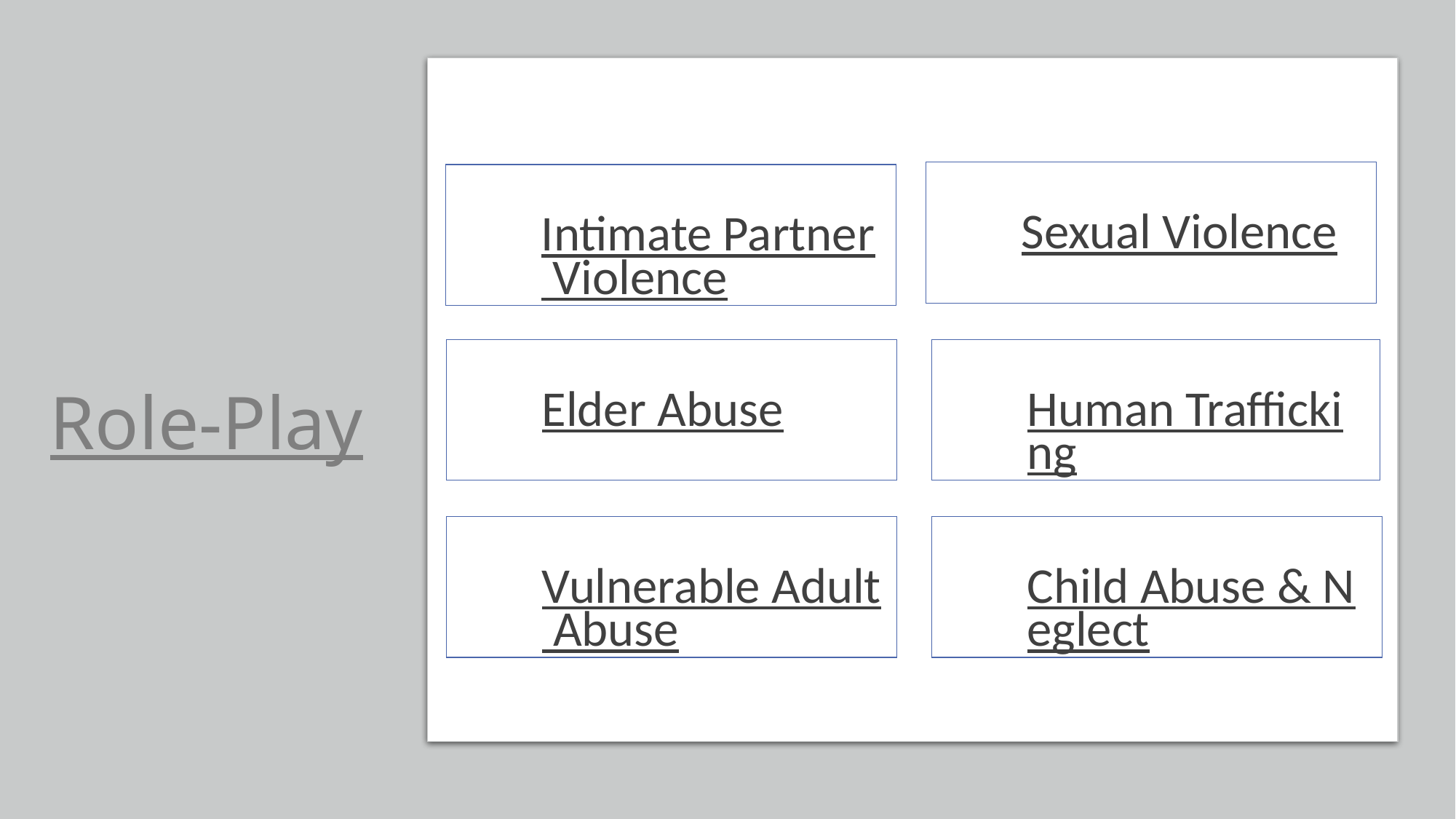

Sexual Violence
Intimate Partner Violence
# Role-Play
Elder Abuse
Human Trafficking
Vulnerable Adult Abuse
Child Abuse & Neglect

## Slide 38
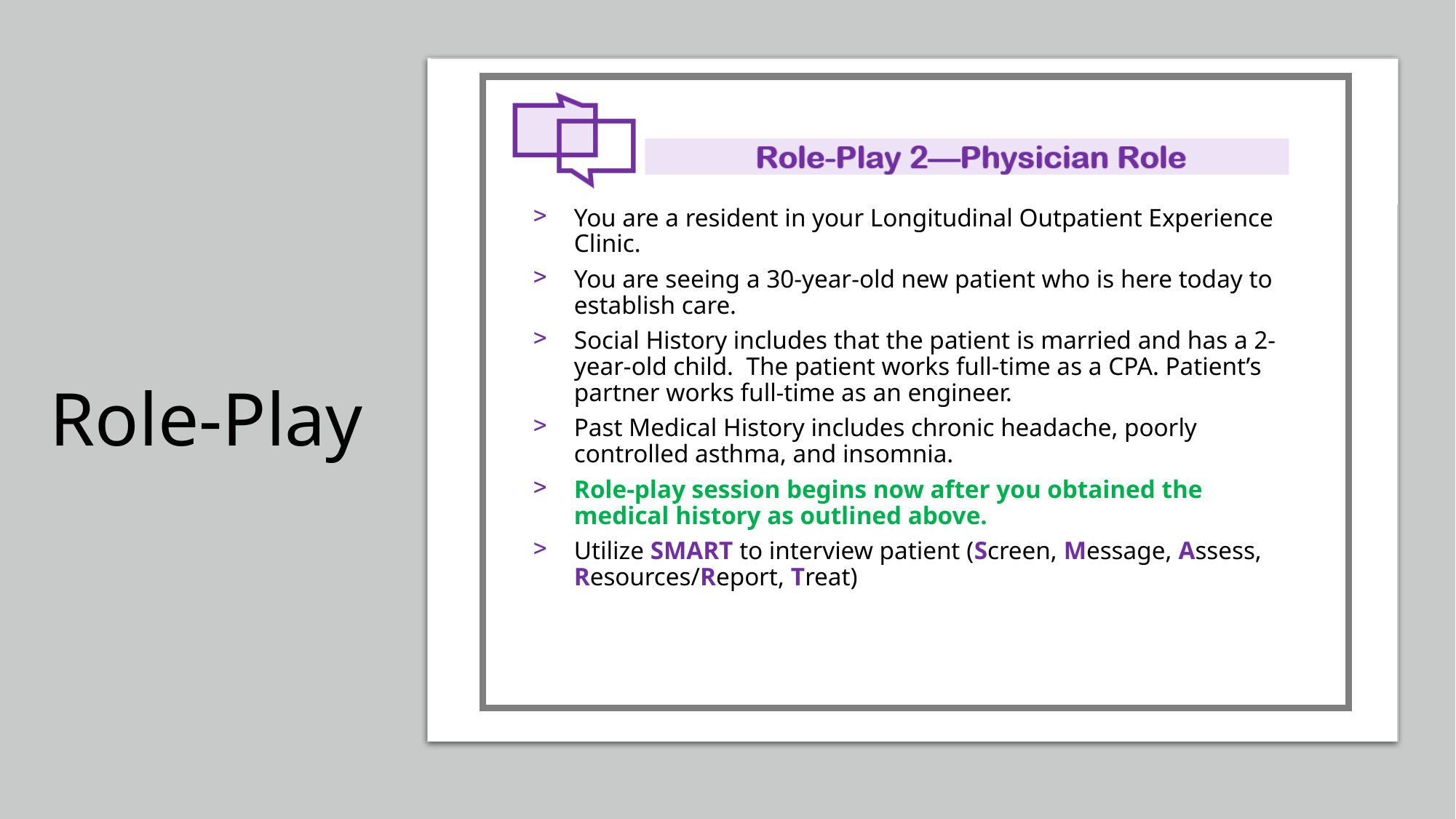

You are a resident in your Longitudinal Outpatient Experience Clinic.
You are seeing a 30-year-old new patient who is here today to establish care.
Social History includes that the patient is married and has a 2-year-old child. The patient works full-time as a CPA. Patient’s partner works full-time as an engineer.
Past Medical History includes chronic headache, poorly controlled asthma, and insomnia.
Role-play session begins now after you obtained the medical history as outlined above.
Utilize SMART to interview patient (Screen, Message, Assess, Resources/Report, Treat)
# Role-Play

## Slide 39
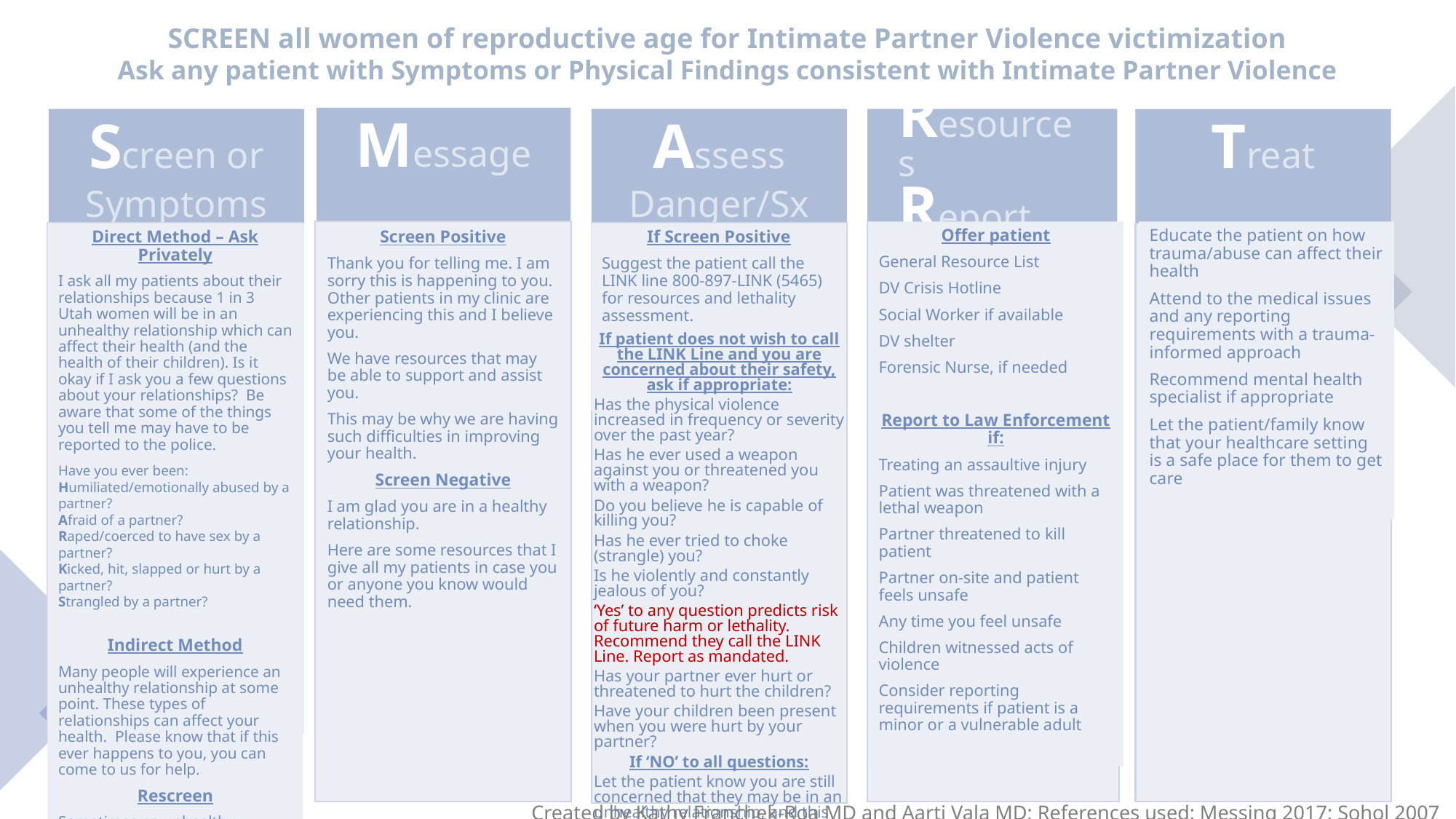

SCREEN all women of reproductive age for Intimate Partner Violence victimization
Ask any patient with Symptoms or Physical Findings consistent with Intimate Partner Violence
Message
Danger/Sx
Screen or
Symptoms
Assess
Danger/Sx
Resources
Report
Treat
Danger/Sx
Educate the patient on how trauma/abuse can affect their health
Attend to the medical issues and any reporting requirements with a trauma-informed approach
Recommend mental health specialist if appropriate
Let the patient/family know that your healthcare setting is a safe place for them to get care
Offer patient
General Resource List
DV Crisis Hotline
Social Worker if available
DV shelter
Forensic Nurse, if needed
Report to Law Enforcement if:
Treating an assaultive injury
Patient was threatened with a lethal weapon
Partner threatened to kill patient
Partner on-site and patient feels unsafe
Any time you feel unsafe
Children witnessed acts of violence
Consider reporting requirements if patient is a minor or a vulnerable adult
Screen Positive
Thank you for telling me. I am sorry this is happening to you. Other patients in my clinic are experiencing this and I believe you.
We have resources that may be able to support and assist you.
This may be why we are having such difficulties in improving your health.
Screen Negative
I am glad you are in a healthy relationship.
Here are some resources that I give all my patients in case you or anyone you know would need them.
Direct Method – Ask Privately
I ask all my patients about their relationships because 1 in 3 Utah women will be in an unhealthy relationship which can affect their health (and the health of their children). Is it okay if I ask you a few questions about your relationships? Be aware that some of the things you tell me may have to be reported to the police.
Have you ever been:
Humiliated/emotionally abused by a partner?
Afraid of a partner?
Raped/coerced to have sex by a partner?
Kicked, hit, slapped or hurt by a partner?
Strangled by a partner?
Indirect Method
Many people will experience an unhealthy relationship at some point. These types of relationships can affect your health. Please know that if this ever happens to you, you can come to us for help.
Rescreen
Sometimes an unhealthy relationship can interfere with your ability to improve your health. Do you think this might be happening to you?
If Screen Positive
Suggest the patient call the LINK line 800-897-LINK (5465) for resources and lethality assessment.
If patient does not wish to call the LINK Line and you are concerned about their safety, ask if appropriate:
Has the physical violence increased in frequency or severity over the past year?
Has he ever used a weapon against you or threatened you with a weapon?
Do you believe he is capable of killing you?
Has he ever tried to choke (strangle) you?
Is he violently and constantly jealous of you?
‘Yes’ to any question predicts risk of future harm or lethality. Recommend they call the LINK Line. Report as mandated.
Has your partner ever hurt or threatened to hurt the children?
Have your children been present when you were hurt by your partner?
If ‘NO’ to all questions:
Let the patient know you are still concerned that they may be in an unhealthy relationship, and this might be affecting their health.
Created by Kathy Franchek-Roa MD and Aarti Vala MD; References used: Messing 2017; Sohol 2007

## Slide 40
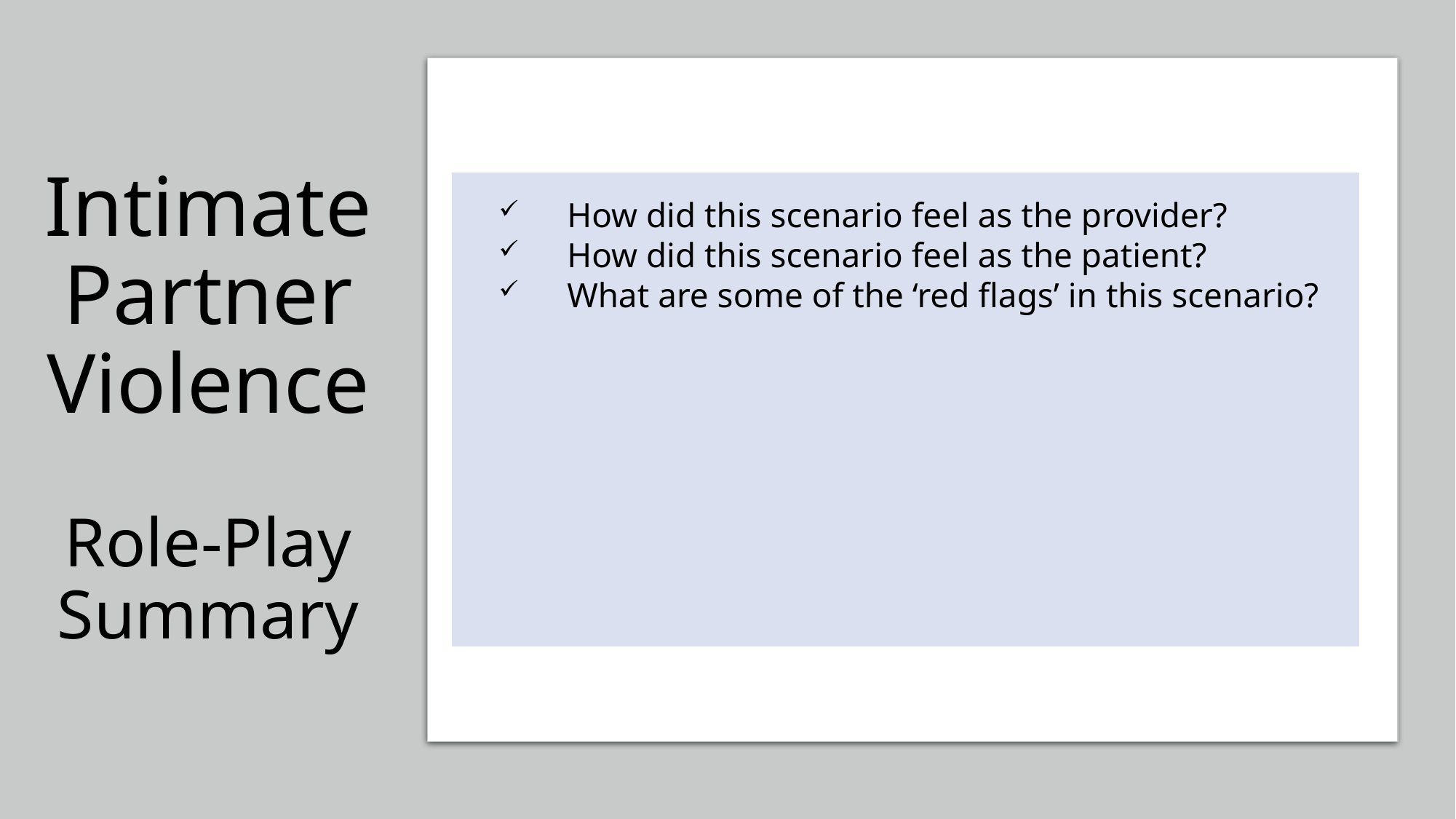

# Intimate Partner Violence Role-Play Summary
How did this scenario feel as the provider?
How did this scenario feel as the patient?
What are some of the ‘red flags’ in this scenario?

## Slide 41
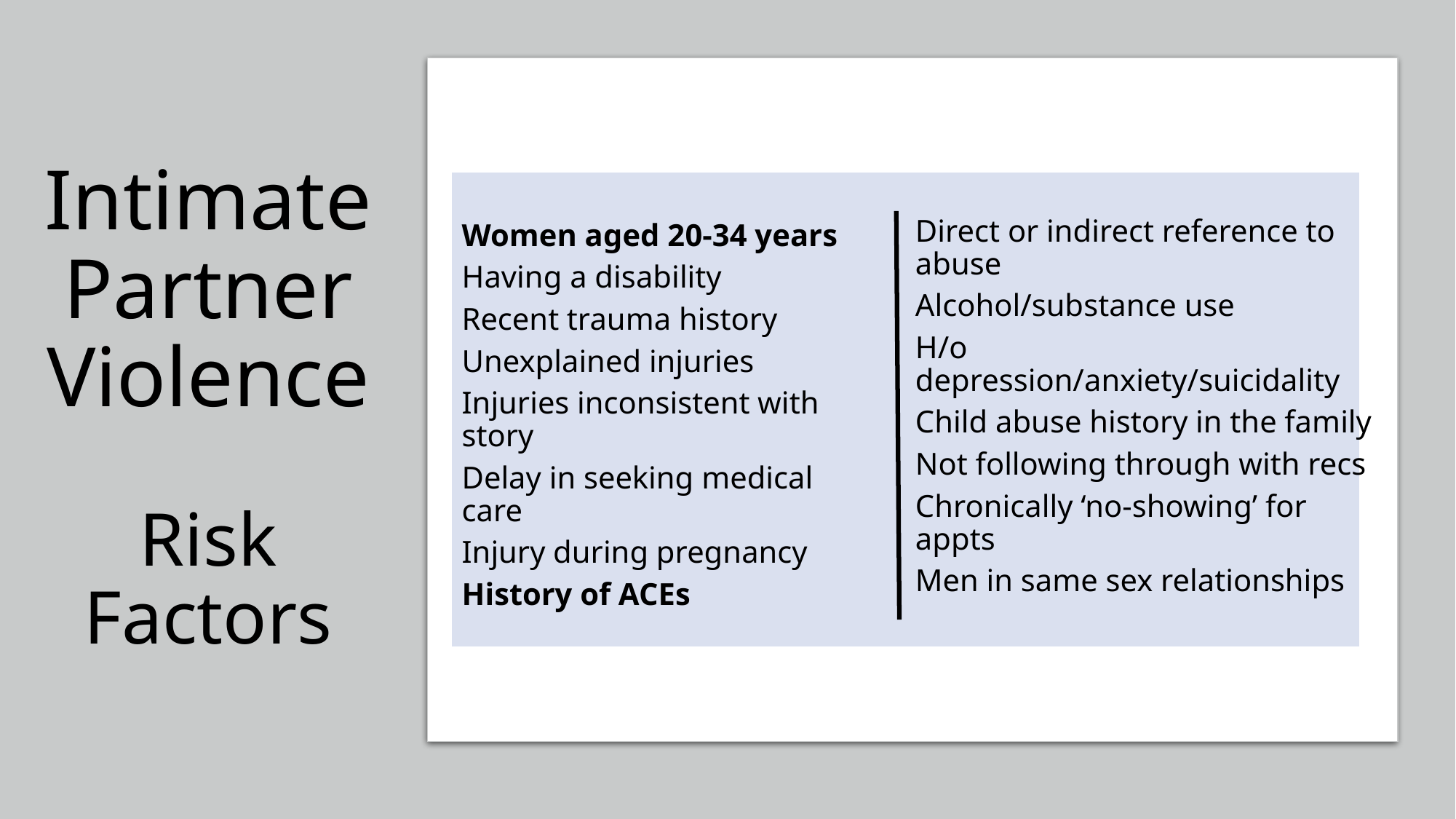

Intimate Partner Violence
Risk Factors
Direct or indirect reference to abuse
Alcohol/substance use
H/o depression/anxiety/suicidality
Child abuse history in the family
Not following through with recs
Chronically ‘no-showing’ for appts
Men in same sex relationships
Women aged 20-34 years
Having a disability
Recent trauma history
Unexplained injuries
Injuries inconsistent with story
Delay in seeking medical care
Injury during pregnancy
History of ACEs

## Slide 42
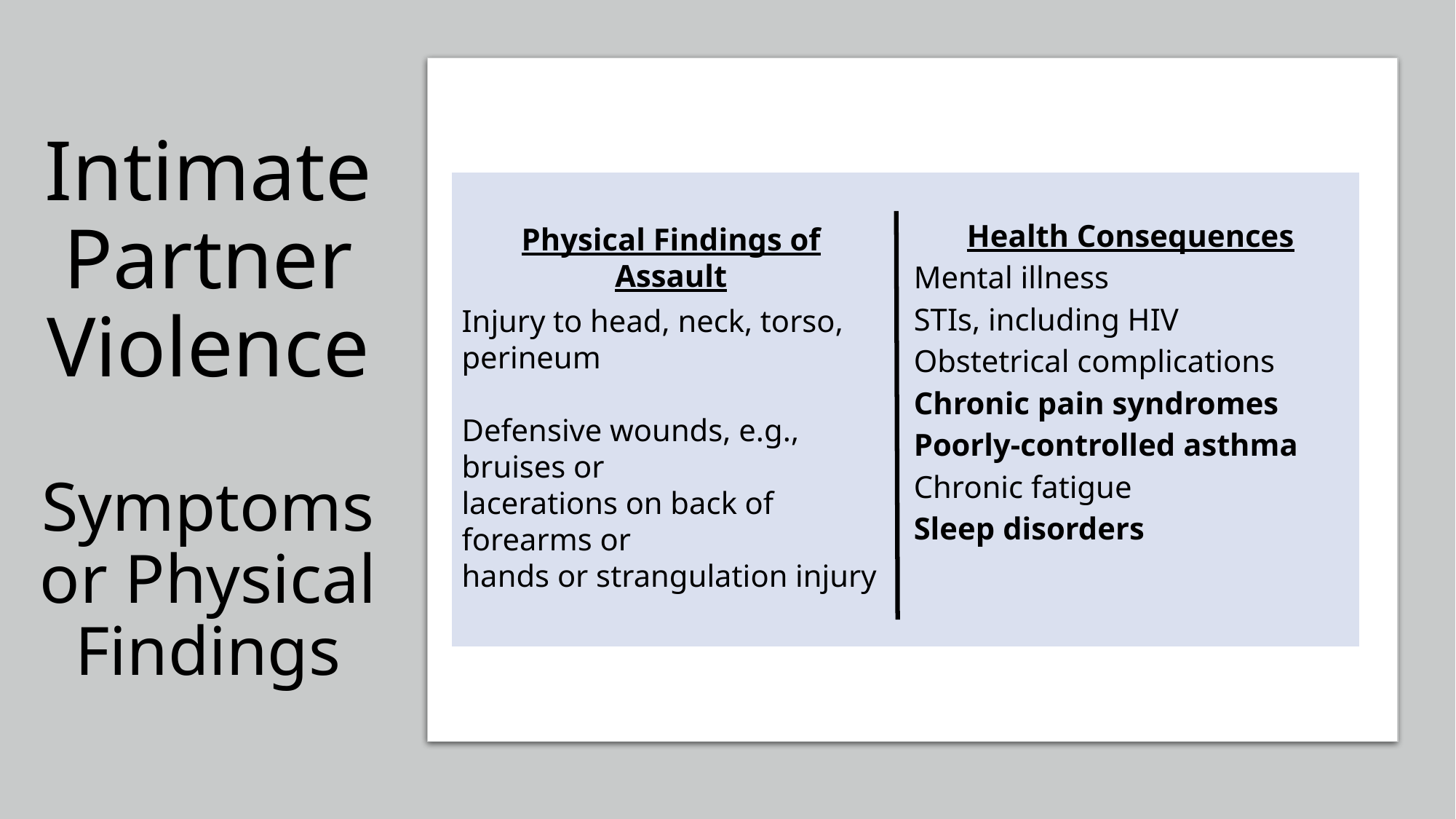

# Intimate Partner Violence Symptoms or Physical Findings
Physical Findings of Assault
Injury to head, neck, torso,
perineum
Defensive wounds, e.g., bruises or
lacerations on back of forearms or
hands or strangulation injury
Health Consequences
Mental illness
STIs, including HIV
Obstetrical complications
Chronic pain syndromes
Poorly-controlled asthma
Chronic fatigue
Sleep disorders

## Slide 43
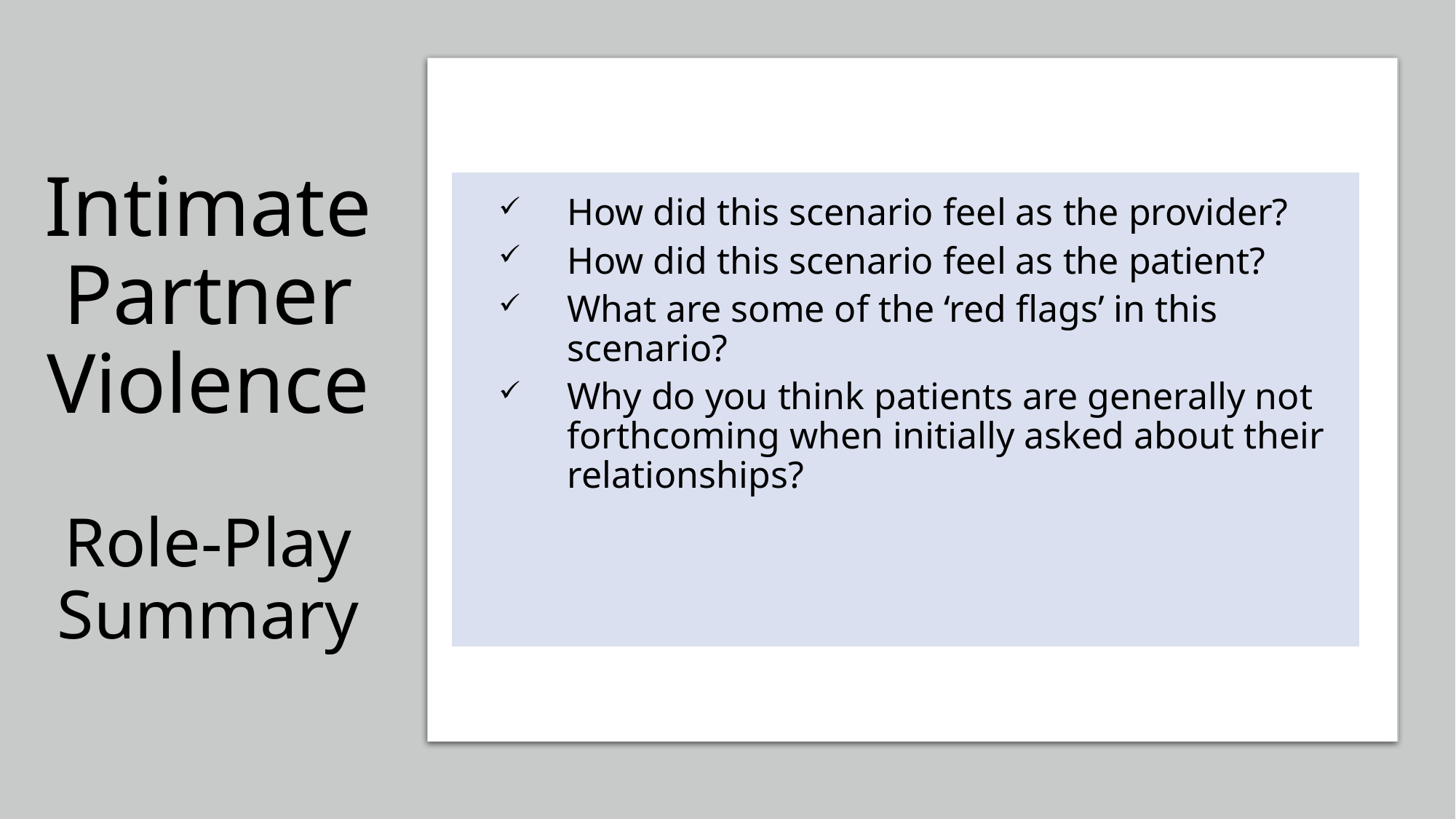

Intimate Partner Violence
Role-Play Summary
How did this scenario feel as the provider?
How did this scenario feel as the patient?
What are some of the ‘red flags’ in this scenario?
Why do you think patients are generally not forthcoming when initially asked about their relationships?

## Slide 44
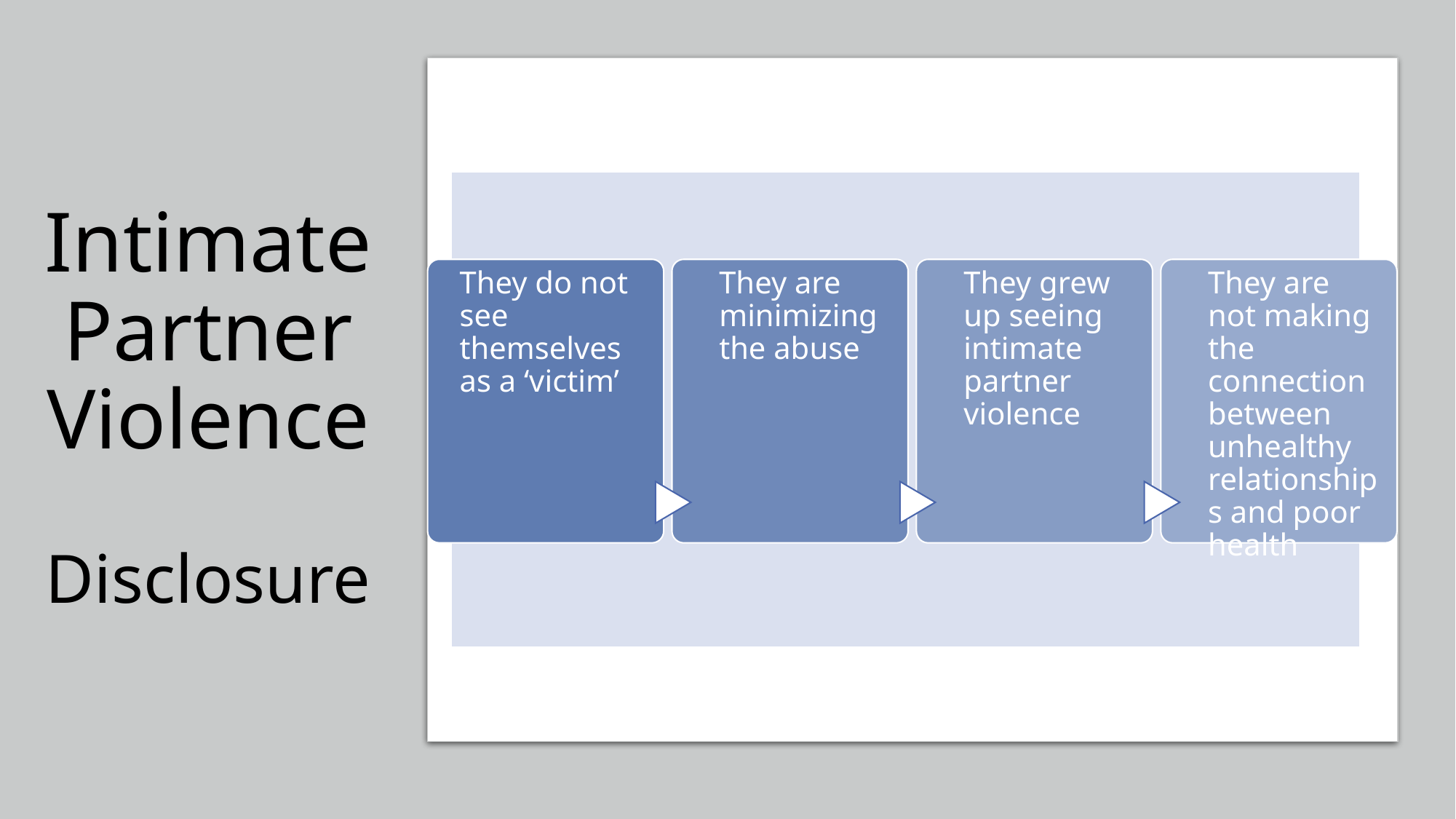

# Intimate Partner Violence Disclosure
They do not see themselves as a ‘victim’
They are minimizing the abuse
They grew up seeing intimate partner violence
They are not making the connection between unhealthy relationships and poor health

## Slide 45
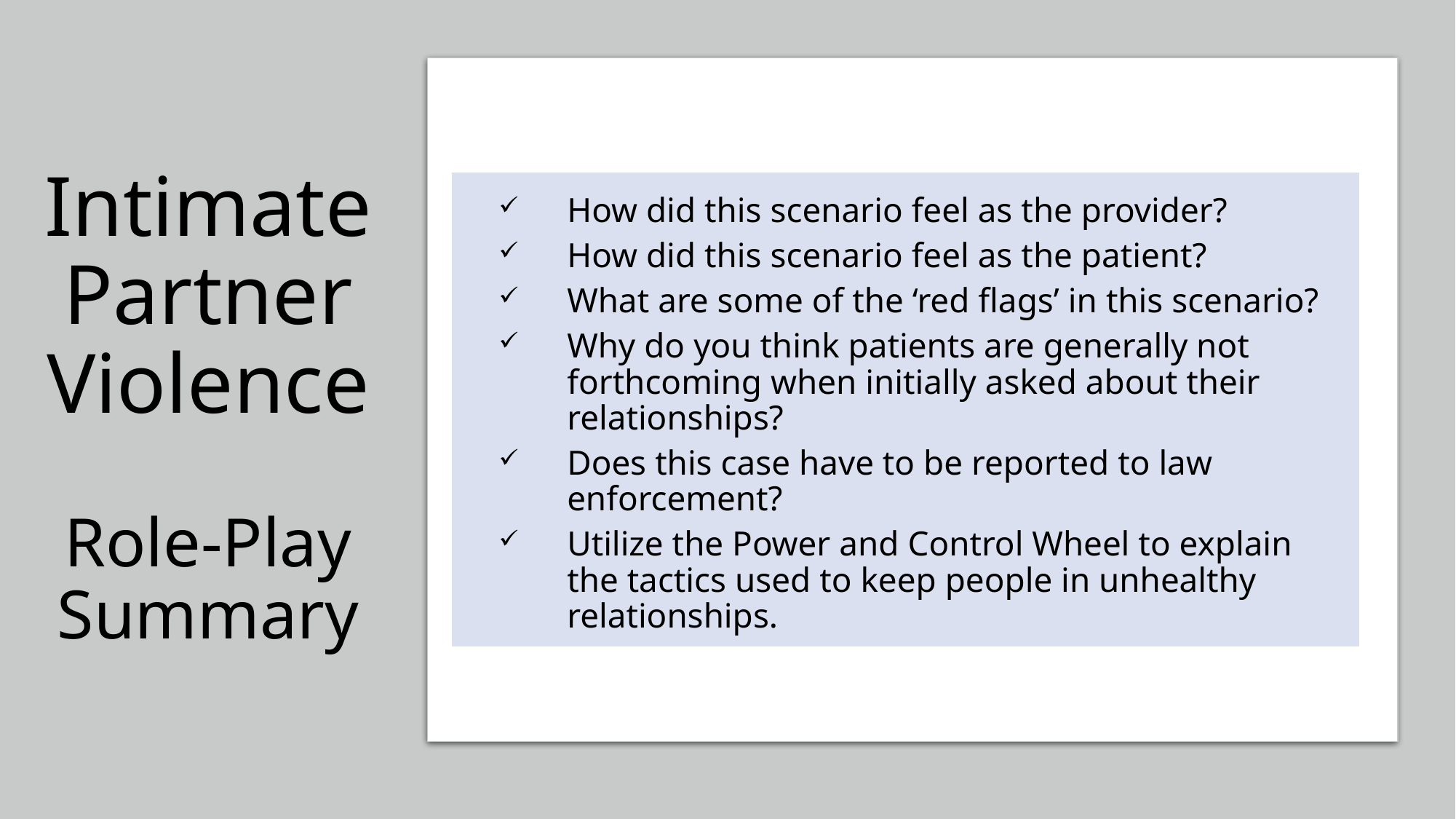

Intimate Partner Violence
Role-Play Summary
How did this scenario feel as the provider?
How did this scenario feel as the patient?
What are some of the ‘red flags’ in this scenario?
Why do you think patients are generally not forthcoming when initially asked about their relationships?
Does this case have to be reported to law enforcement?
Utilize the Power and Control Wheel to explain the tactics used to keep people in unhealthy relationships.

## Slide 46
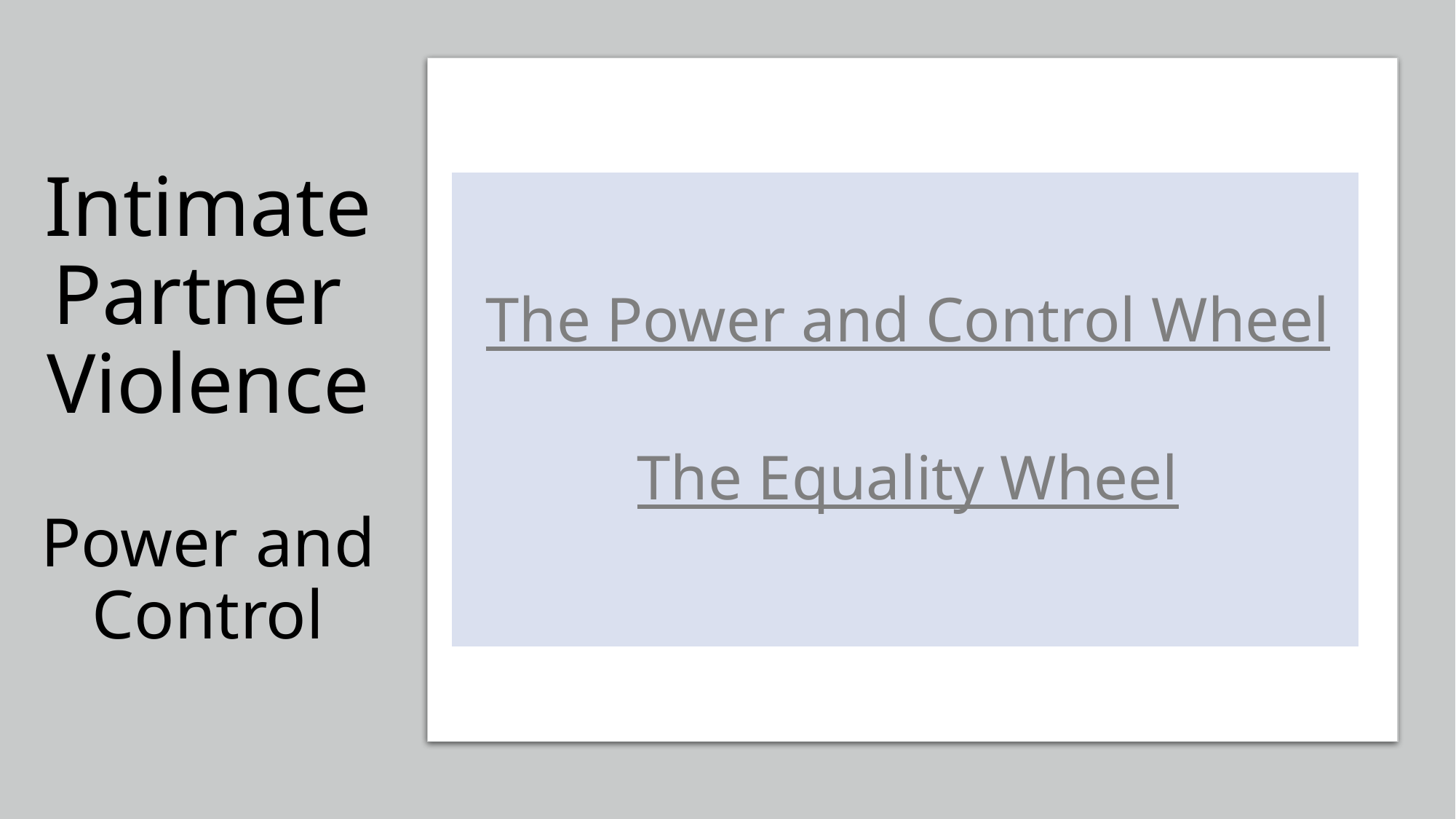

# Intimate Partner Violence Power and Control
The Power and Control Wheel
The Equality Wheel

## Slide 47
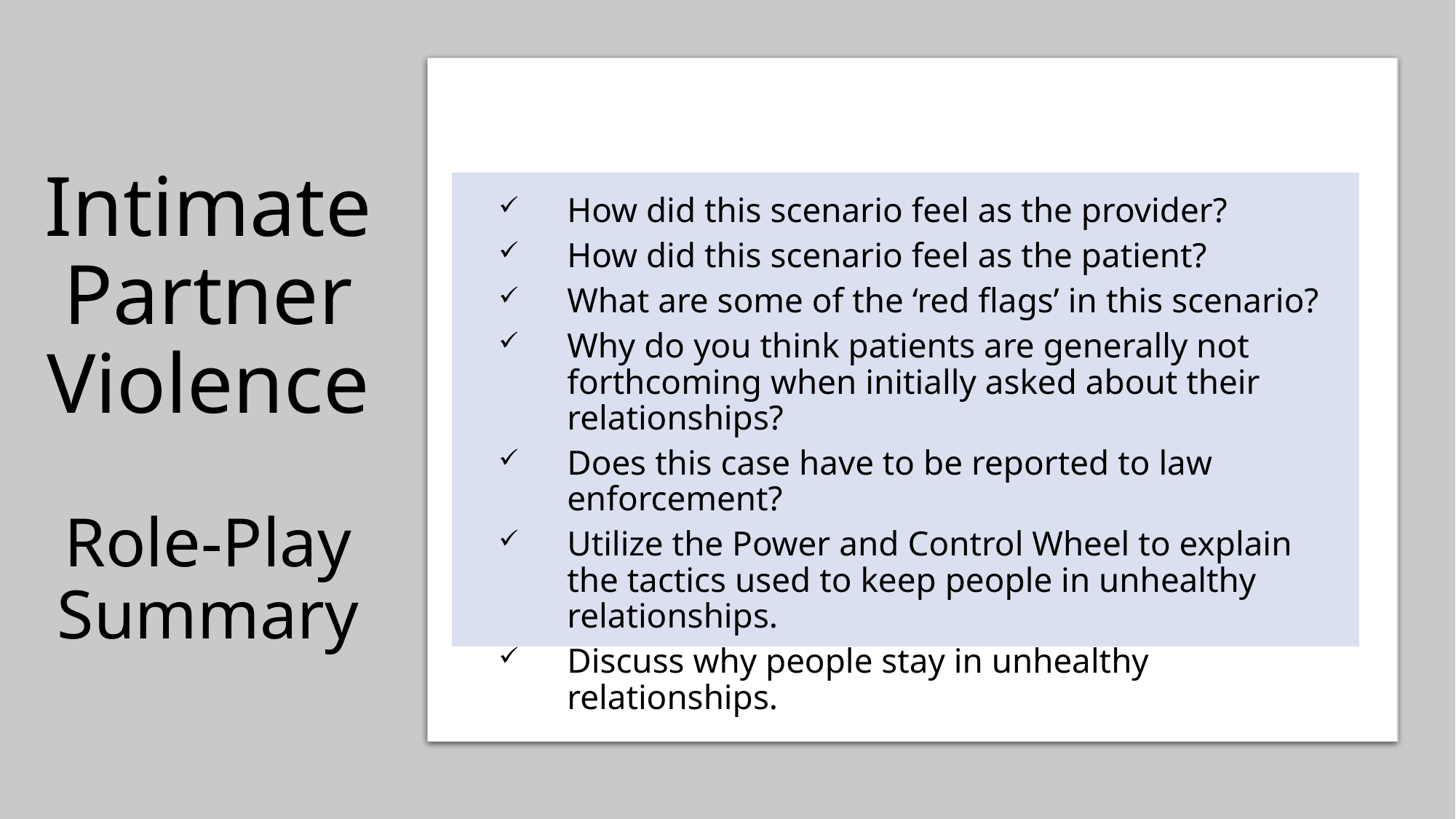

# Intimate Partner Violence Role-Play Summary
How did this scenario feel as the provider?
How did this scenario feel as the patient?
What are some of the ‘red flags’ in this scenario?
Why do you think patients are generally not forthcoming when initially asked about their relationships?
Does this case have to be reported to law enforcement?
Utilize the Power and Control Wheel to explain the tactics used to keep people in unhealthy relationships.
Discuss why people stay in unhealthy relationships.

## Slide 48
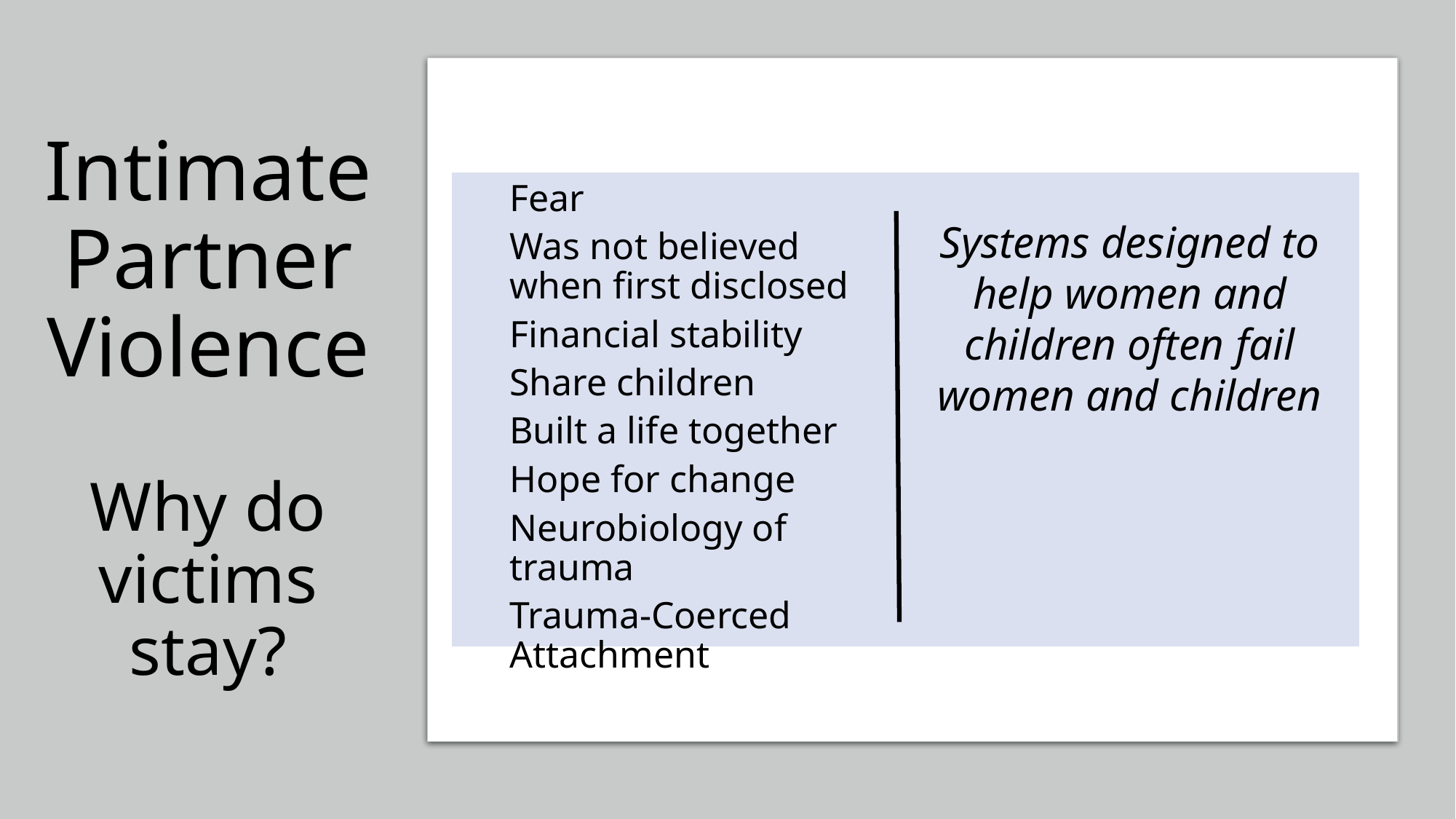

# Intimate Partner Violence Why do victims stay?
Fear
Was not believed when first disclosed
Financial stability
Share children
Built a life together
Hope for change
Neurobiology of trauma
Trauma-Coerced Attachment
Systems designed to help women and children often fail women and children

## Slide 49
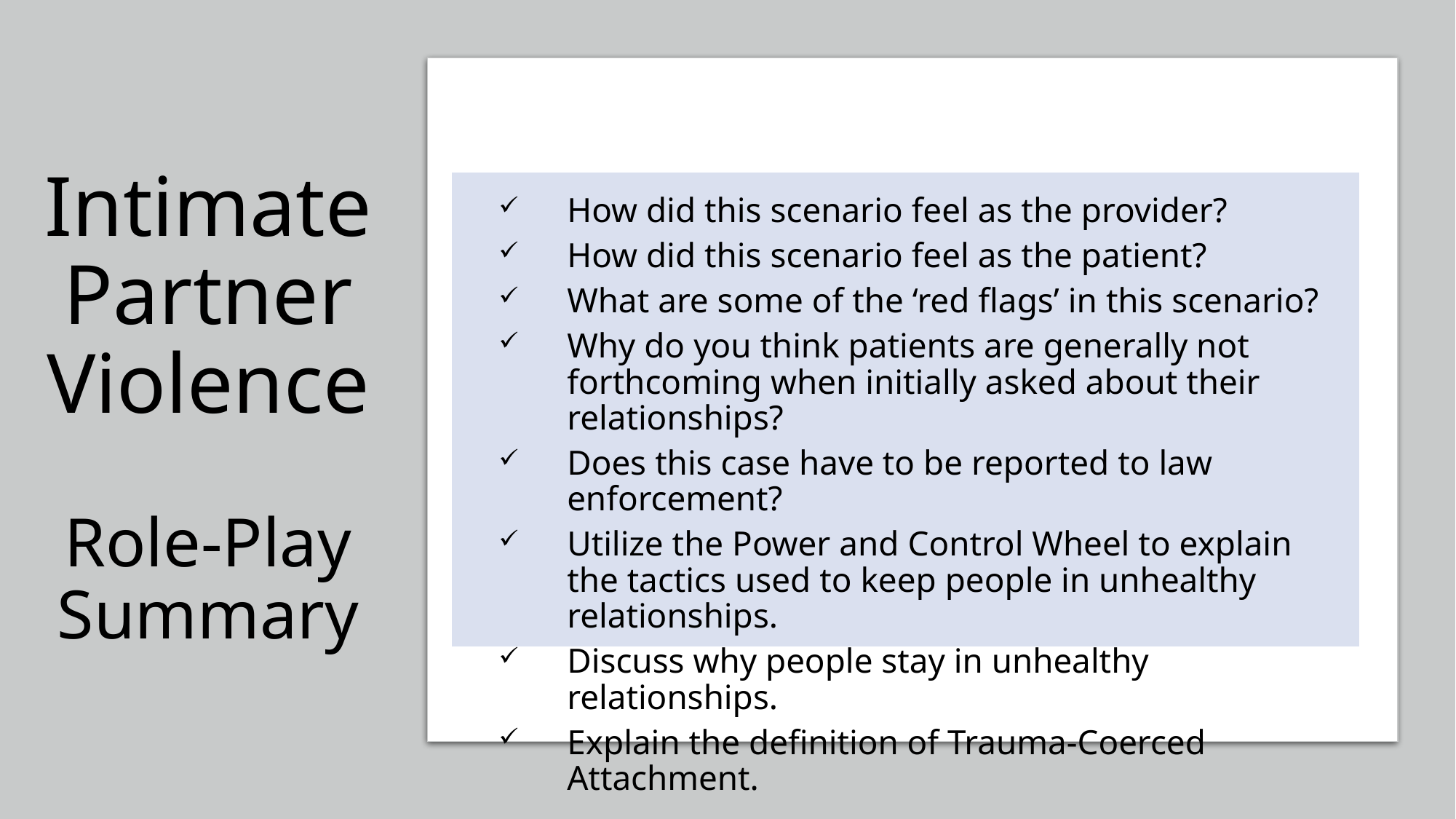

# Intimate Partner Violence Role-Play Summary
How did this scenario feel as the provider?
How did this scenario feel as the patient?
What are some of the ‘red flags’ in this scenario?
Why do you think patients are generally not forthcoming when initially asked about their relationships?
Does this case have to be reported to law enforcement?
Utilize the Power and Control Wheel to explain the tactics used to keep people in unhealthy relationships.
Discuss why people stay in unhealthy relationships.
Explain the definition of Trauma-Coerced Attachment.

## Slide 50
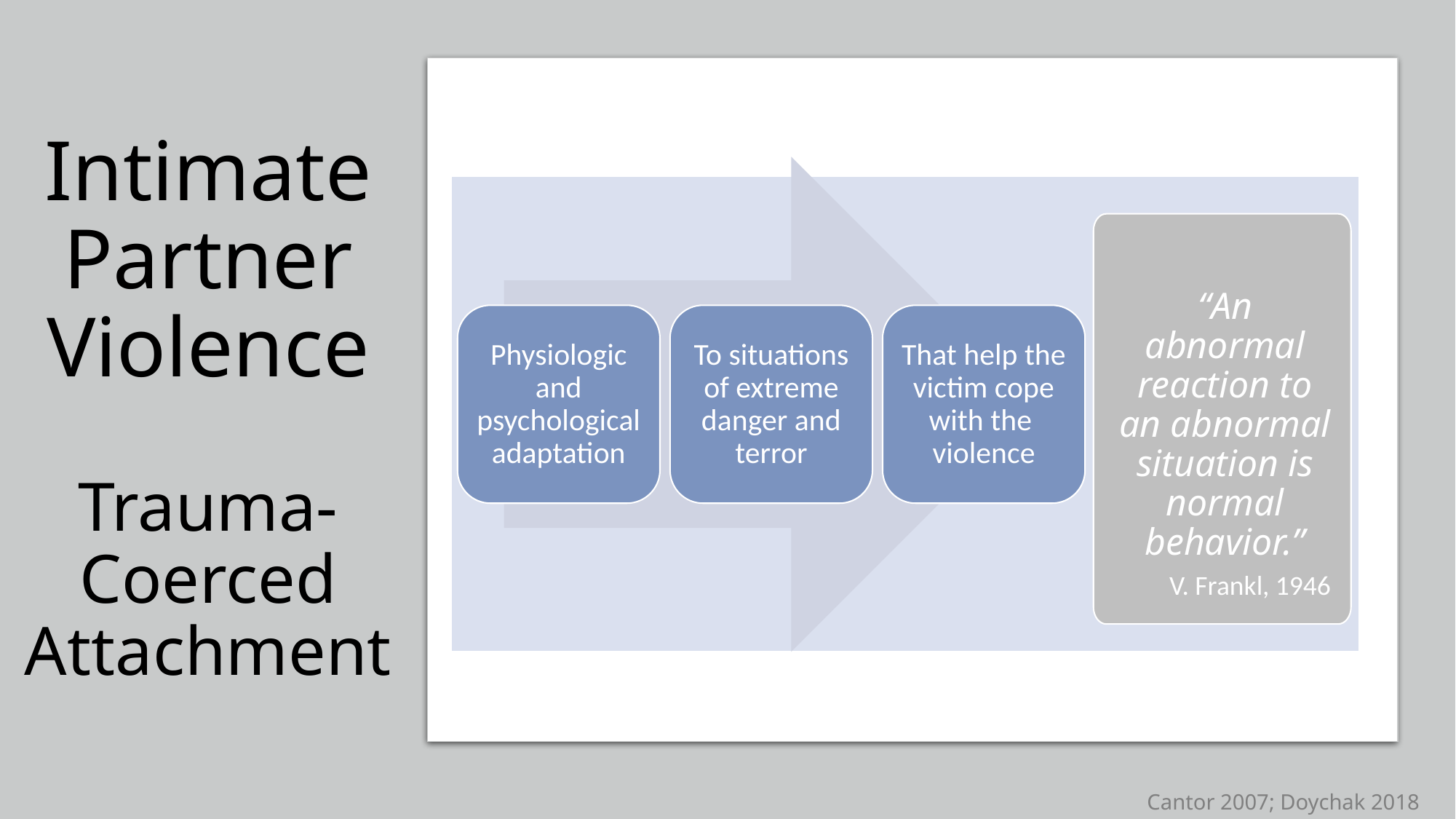

# Intimate Partner Violence Trauma- Coerced Attachment
Physiologic and psychological adaptation
To situations of extreme danger and terror
That help the victim cope with the violence
“An abnormal reaction to an abnormal situation is normal behavior.”
V. Frankl, 1946
Cantor 2007; Doychak 2018

## Slide 51
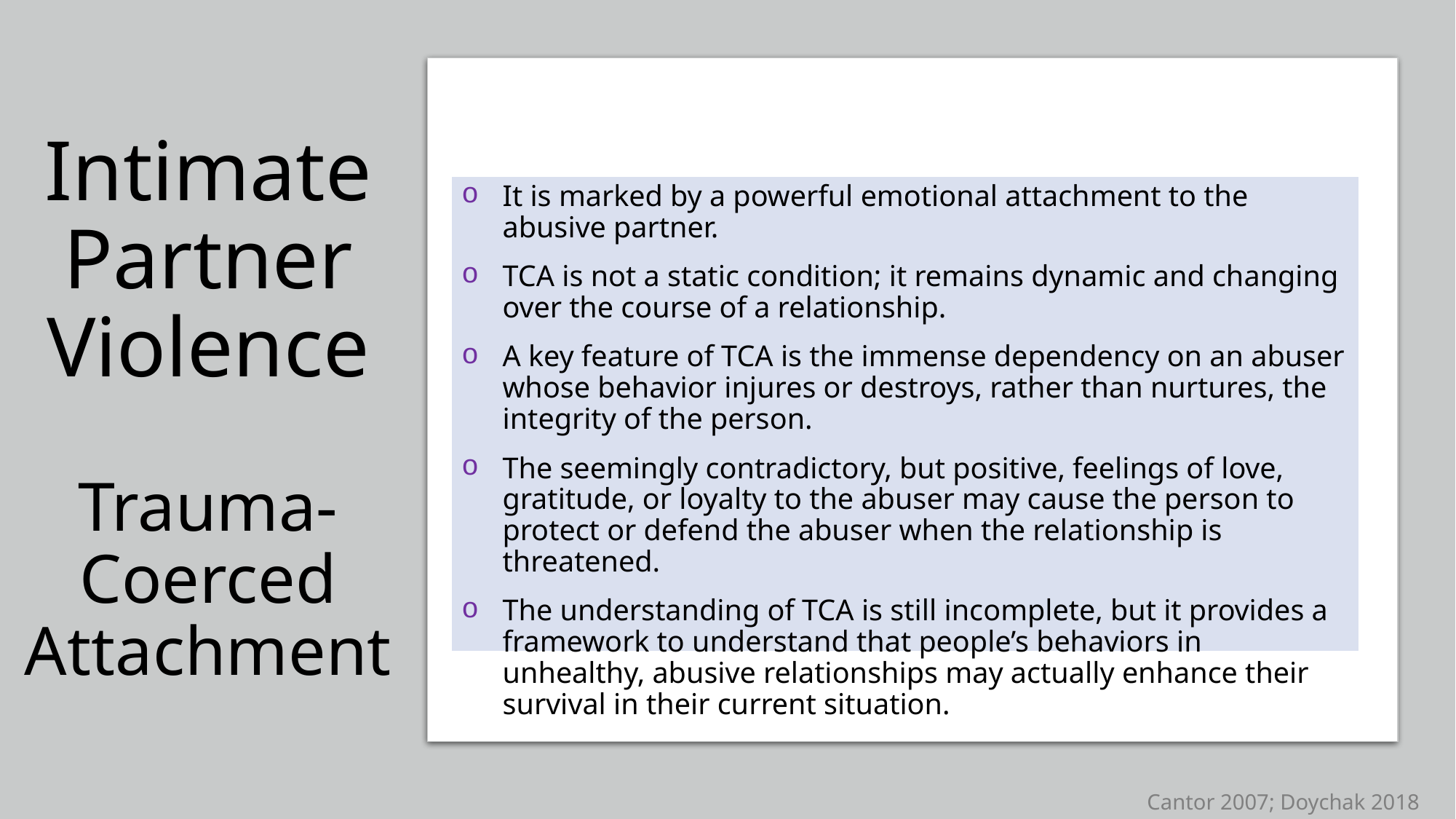

# Intimate Partner Violence Trauma- Coerced Attachment
It is marked by a powerful emotional attachment to the abusive partner.
TCA is not a static condition; it remains dynamic and changing over the course of a relationship.
A key feature of TCA is the immense dependency on an abuser whose behavior injures or destroys, rather than nurtures, the integrity of the person.
The seemingly contradictory, but positive, feelings of love, gratitude, or loyalty to the abuser may cause the person to protect or defend the abuser when the relationship is threatened.
The understanding of TCA is still incomplete, but it provides a framework to understand that people’s behaviors in unhealthy, abusive relationships may actually enhance their survival in their current situation.
Cantor 2007; Doychak 2018

## Slide 52
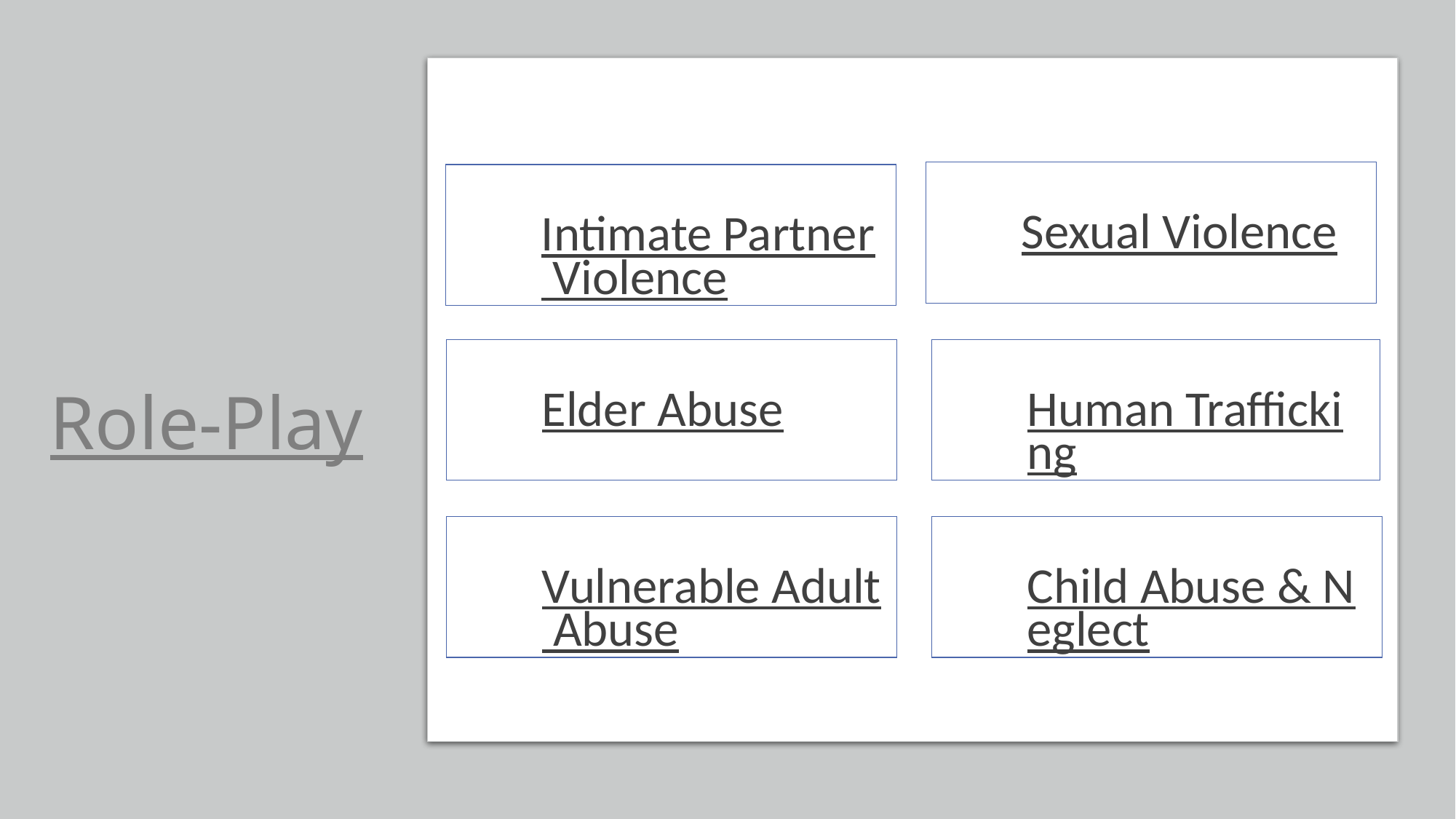

Sexual Violence
Intimate Partner Violence
# Role-Play
Elder Abuse
Human Trafficking
Vulnerable Adult Abuse
Child Abuse & Neglect

## Slide 53
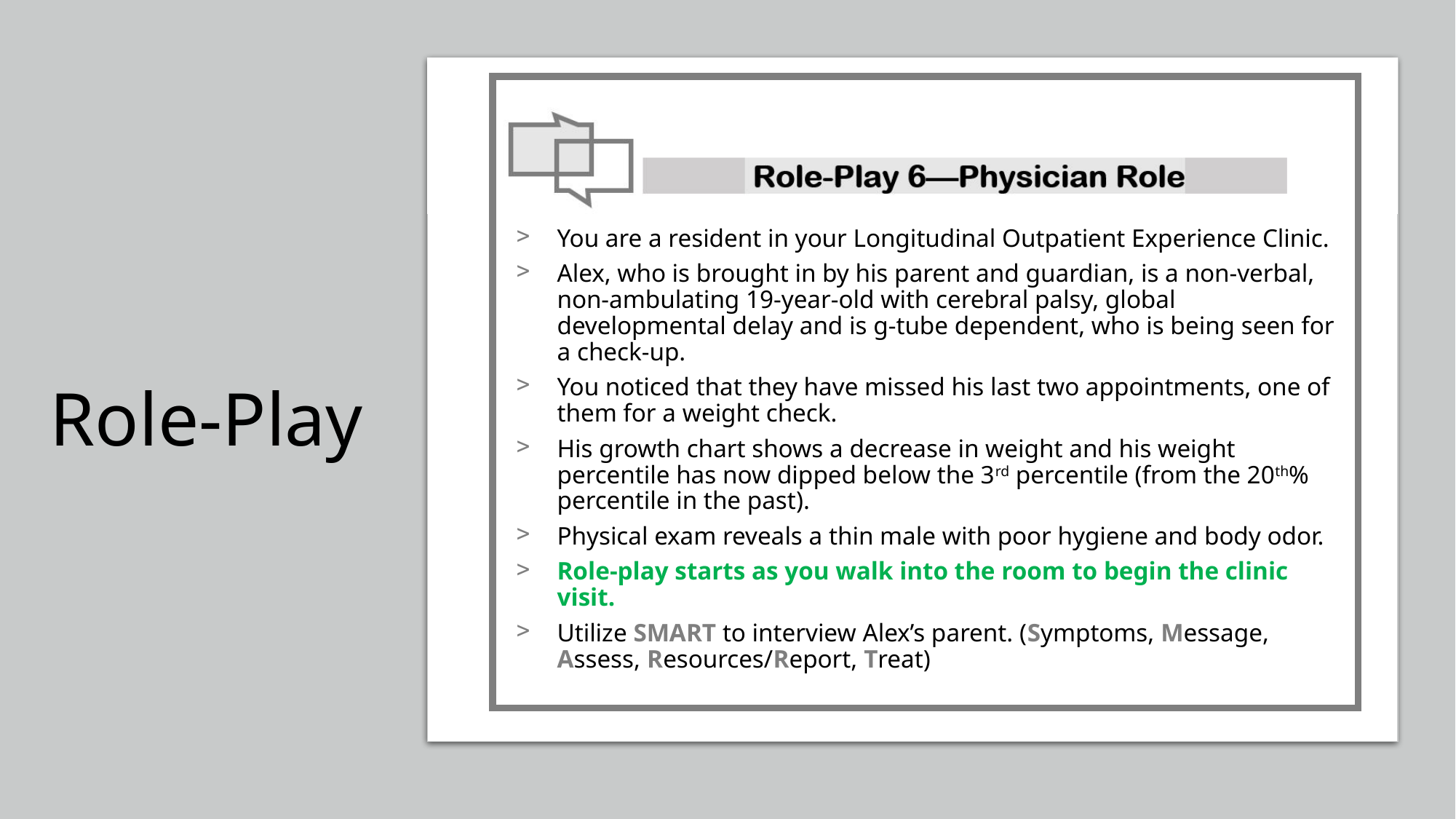

You are a resident in your Longitudinal Outpatient Experience Clinic.
Alex, who is brought in by his parent and guardian, is a non-verbal, non-ambulating 19-year-old with cerebral palsy, global developmental delay and is g-tube dependent, who is being seen for a check-up.
You noticed that they have missed his last two appointments, one of them for a weight check.
His growth chart shows a decrease in weight and his weight percentile has now dipped below the 3rd percentile (from the 20th% percentile in the past).
Physical exam reveals a thin male with poor hygiene and body odor.
Role-play starts as you walk into the room to begin the clinic visit.
Utilize SMART to interview Alex’s parent. (Symptoms, Message, Assess, Resources/Report, Treat)
# Role-Play

## Slide 54
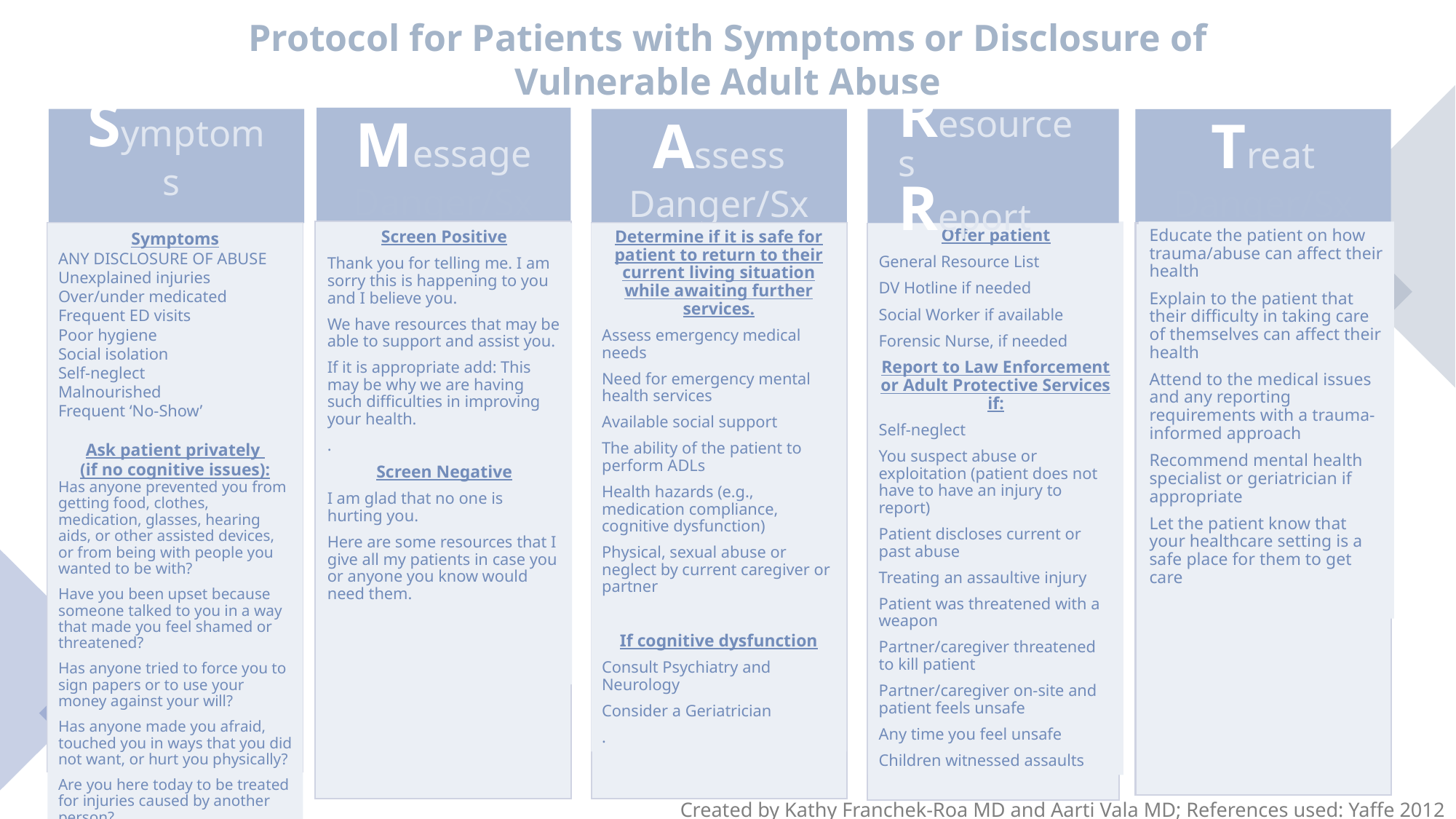

Protocol for Patients with Symptoms or Disclosure of
Vulnerable Adult Abuse
Message
Danger/Sx
Symptoms
Assess
Danger/Sx
Resources
Report
Treat
Danger/Sx
Educate the patient on how trauma/abuse can affect their health
Explain to the patient that their difficulty in taking care of themselves can affect their health
Attend to the medical issues and any reporting requirements with a trauma-informed approach
Recommend mental health specialist or geriatrician if appropriate
Let the patient know that your healthcare setting is a safe place for them to get care
Screen Positive
Thank you for telling me. I am sorry this is happening to you and I believe you.
We have resources that may be able to support and assist you.
If it is appropriate add: This may be why we are having such difficulties in improving your health.
.
Screen Negative
I am glad that no one is hurting you.
Here are some resources that I give all my patients in case you or anyone you know would need them.
Offer patient
General Resource List
DV Hotline if needed
Social Worker if available
Forensic Nurse, if needed
Report to Law Enforcement or Adult Protective Services if:
Self-neglect
You suspect abuse or exploitation (patient does not have to have an injury to report)
Patient discloses current or past abuse
Treating an assaultive injury
Patient was threatened with a weapon
Partner/caregiver threatened to kill patient
Partner/caregiver on-site and patient feels unsafe
Any time you feel unsafe
Children witnessed assaults
Symptoms
ANY DISCLOSURE OF ABUSE Unexplained injuries
Over/under medicated
Frequent ED visits
Poor hygiene
Social isolation
Self-neglect
Malnourished
Frequent ‘No-Show’
Ask patient privately
(if no cognitive issues):
Has anyone prevented you from getting food, clothes, medication, glasses, hearing aids, or other assisted devices, or from being with people you wanted to be with?
Have you been upset because someone talked to you in a way that made you feel shamed or threatened?
Has anyone tried to force you to sign papers or to use your money against your will?
Has anyone made you afraid, touched you in ways that you did not want, or hurt you physically?
Are you here today to be treated for injuries caused by another person?
Determine if it is safe for patient to return to their current living situation while awaiting further services.
Assess emergency medical needs
Need for emergency mental health services
Available social support
The ability of the patient to perform ADLs
Health hazards (e.g., medication compliance, cognitive dysfunction)
Physical, sexual abuse or neglect by current caregiver or partner
If cognitive dysfunction
Consult Psychiatry and Neurology
Consider a Geriatrician
.
Created by Kathy Franchek-Roa MD and Aarti Vala MD; References used: Yaffe 2012

## Slide 55
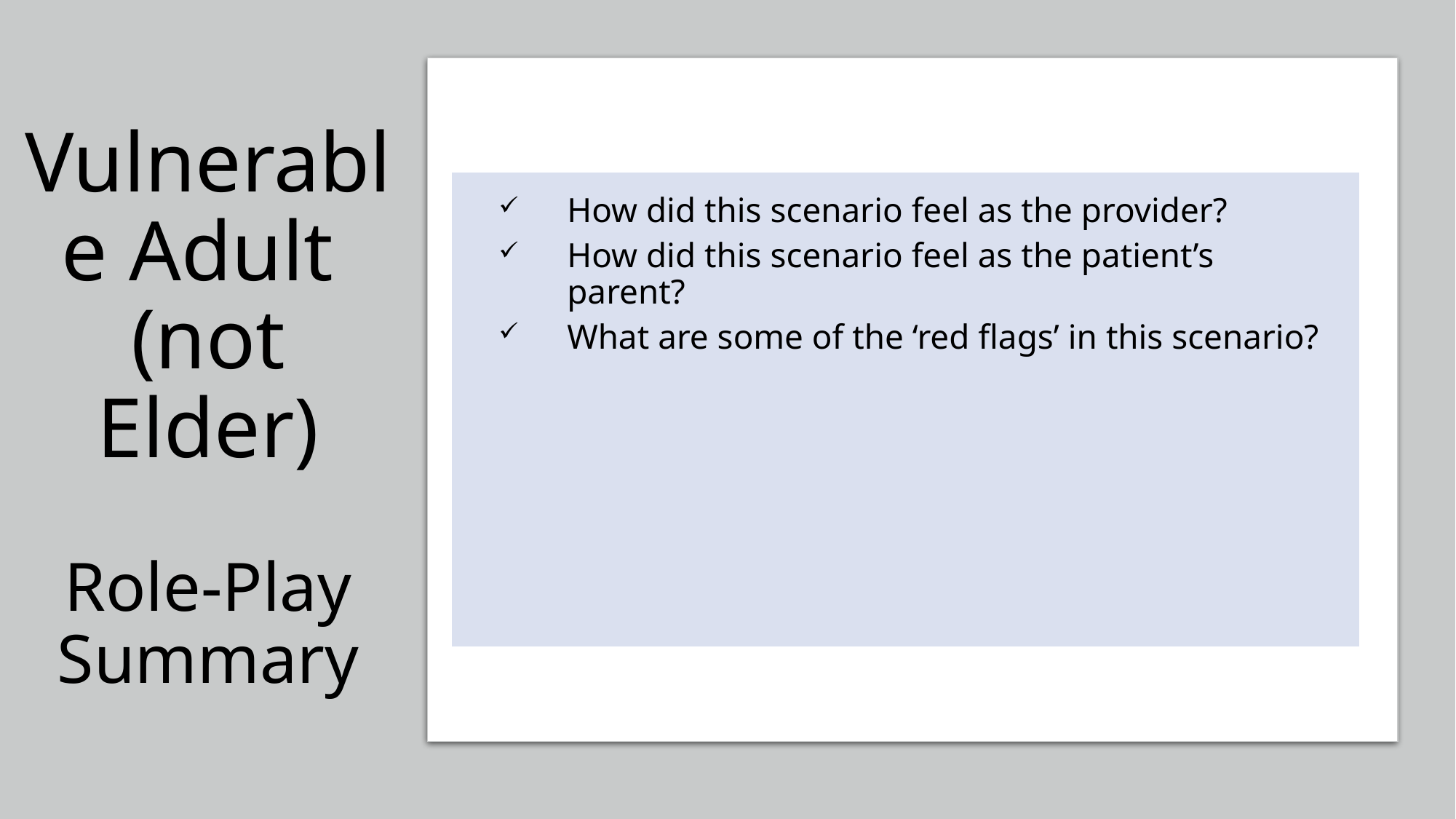

# Vulnerable Adult (not Elder) Role-Play Summary
How did this scenario feel as the provider?
How did this scenario feel as the patient’s parent?
What are some of the ‘red flags’ in this scenario?

## Slide 56
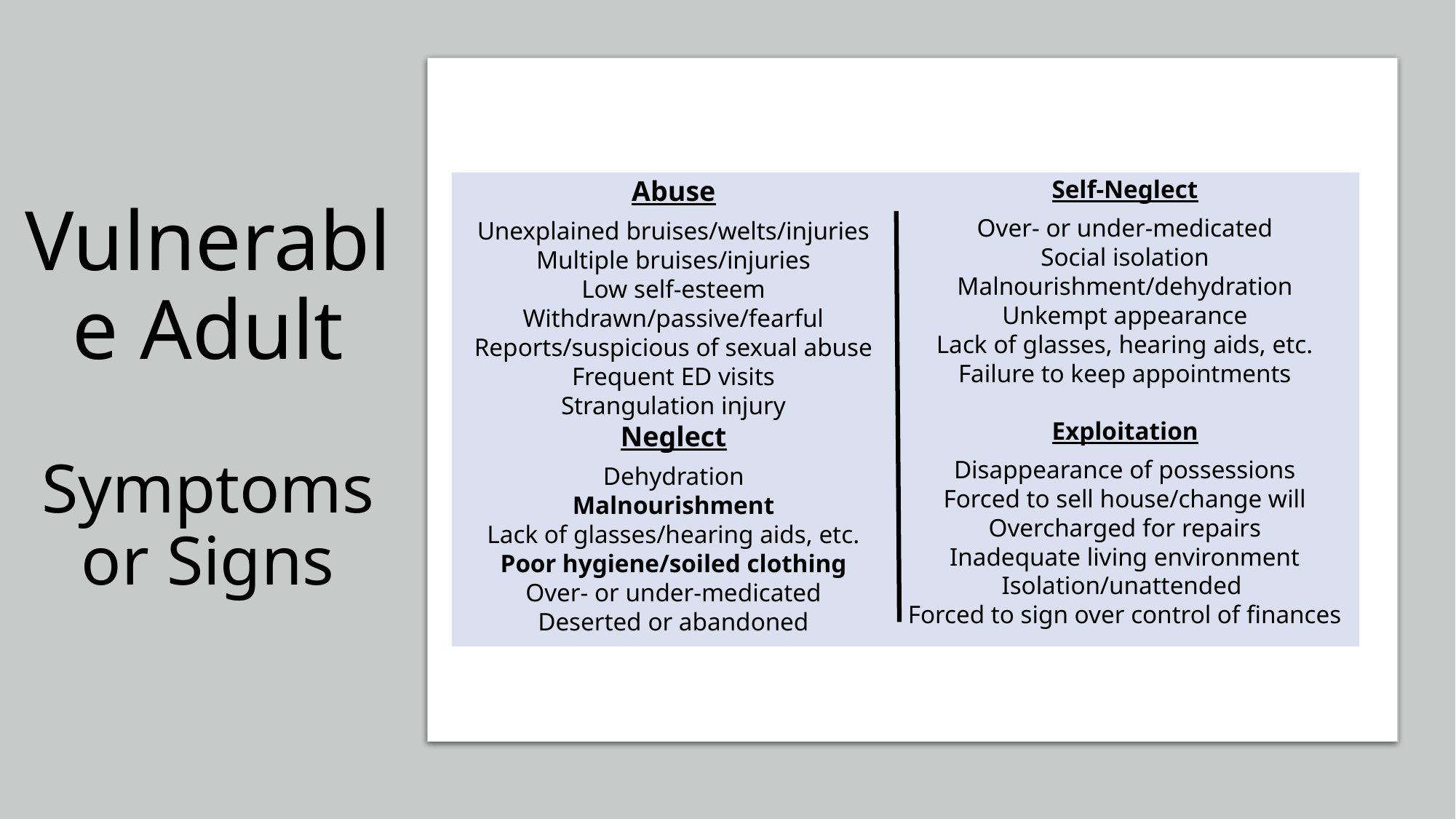

Abuse
Unexplained bruises/welts/injuries
Multiple bruises/injuries
Low self-esteem
Withdrawn/passive/fearful
Reports/suspicious of sexual abuse
Frequent ED visits
Strangulation injury
Neglect
Dehydration
Malnourishment
Lack of glasses/hearing aids, etc.
Poor hygiene/soiled clothing
Over- or under-medicated
Deserted or abandoned
Self-Neglect
Over- or under-medicated
Social isolation
Malnourishment/dehydration
Unkempt appearance
Lack of glasses, hearing aids, etc.
Failure to keep appointments
Exploitation
Disappearance of possessions
Forced to sell house/change will
Overcharged for repairs
Inadequate living environment
Isolation/unattended
Forced to sign over control of finances
# Vulnerable Adult Symptoms or Signs

## Slide 57
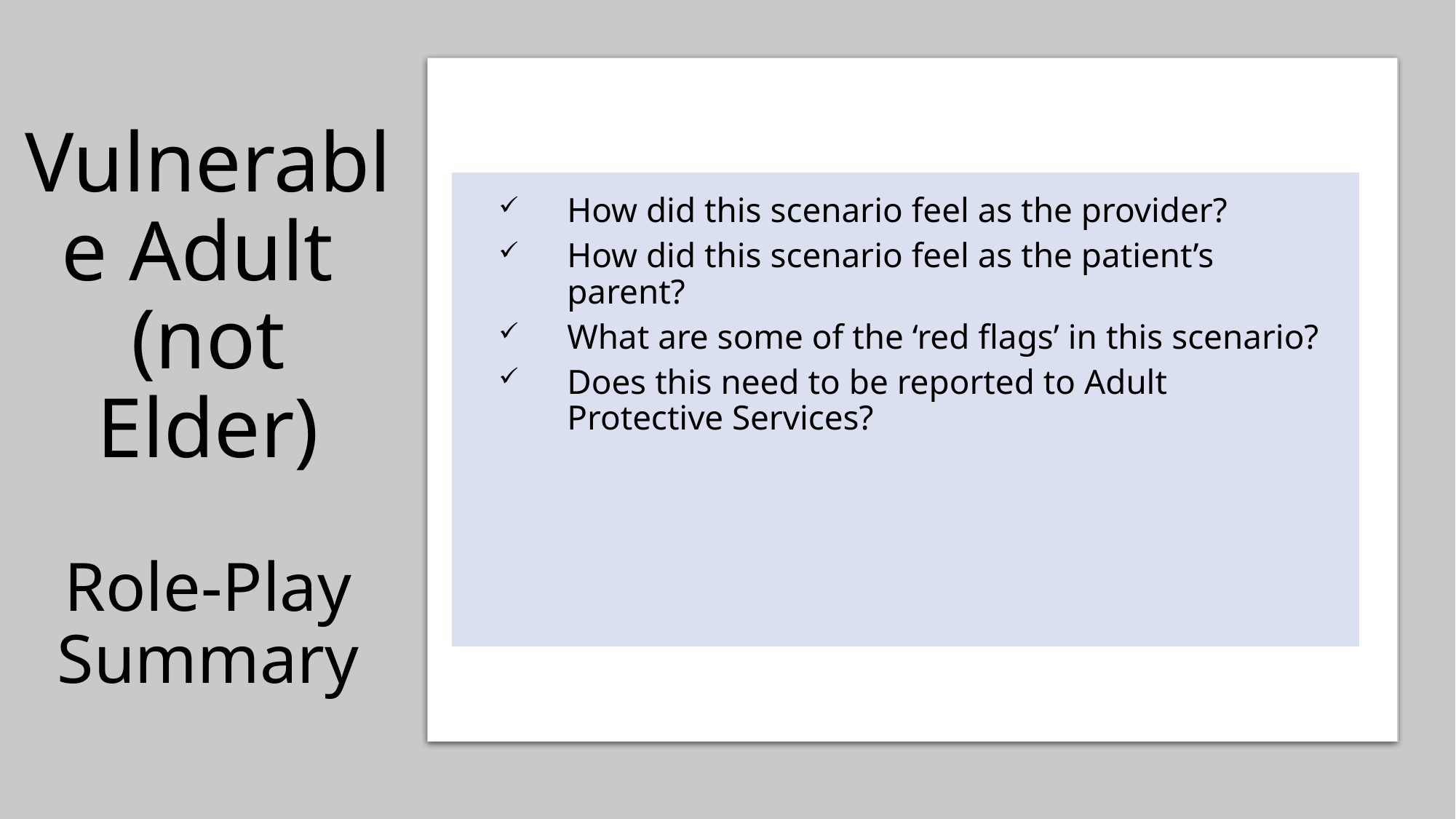

# Vulnerable Adult (not Elder) Role-Play Summary
How did this scenario feel as the provider?
How did this scenario feel as the patient’s parent?
What are some of the ‘red flags’ in this scenario?
Does this need to be reported to Adult Protective Services?

## Slide 58
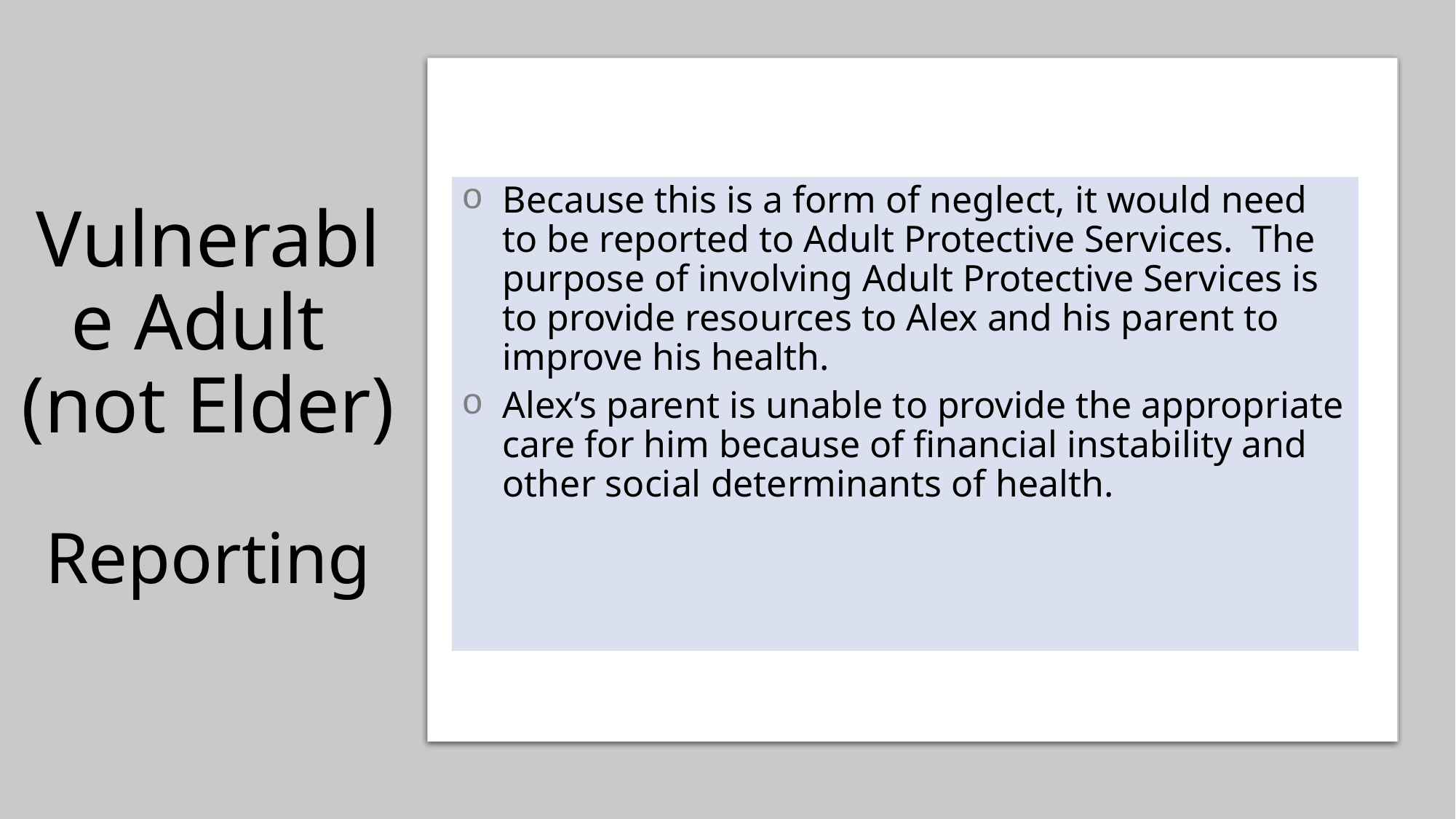

Because this is a form of neglect, it would need to be reported to Adult Protective Services. The purpose of involving Adult Protective Services is to provide resources to Alex and his parent to improve his health.
Alex’s parent is unable to provide the appropriate care for him because of financial instability and other social determinants of health.
# Vulnerable Adult (not Elder) Reporting

## Slide 59
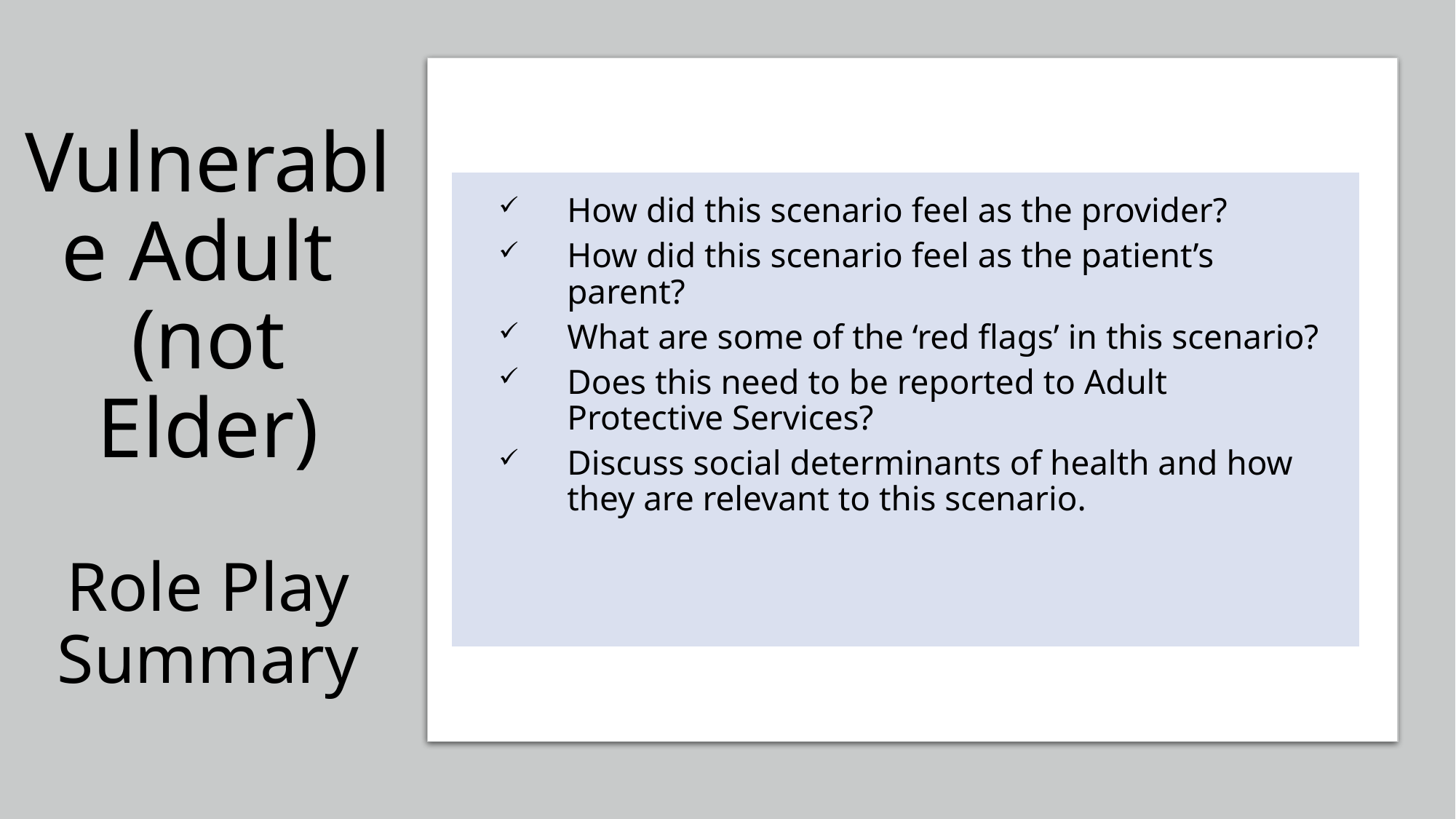

# Vulnerable Adult (not Elder) Role Play Summary
How did this scenario feel as the provider?
How did this scenario feel as the patient’s parent?
What are some of the ‘red flags’ in this scenario?
Does this need to be reported to Adult Protective Services?
Discuss social determinants of health and how they are relevant to this scenario.

## Slide 60
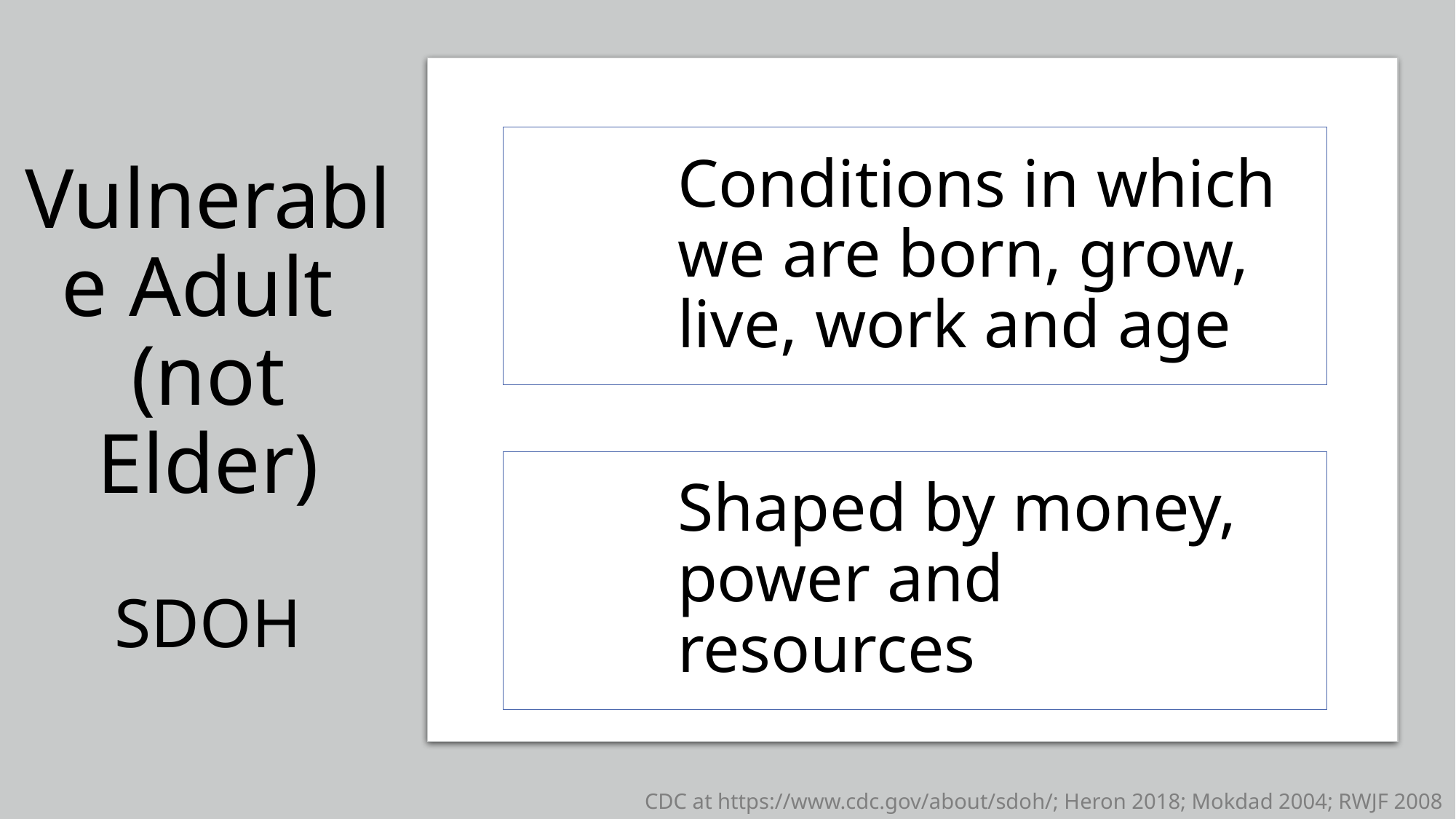

# Vulnerable Adult (not Elder) SDOH
Conditions in which we are born, grow, live, work and age
Shaped by money, power and resources
CDC at https://www.cdc.gov/about/sdoh/; Heron 2018; Mokdad 2004; RWJF 2008

## Slide 61
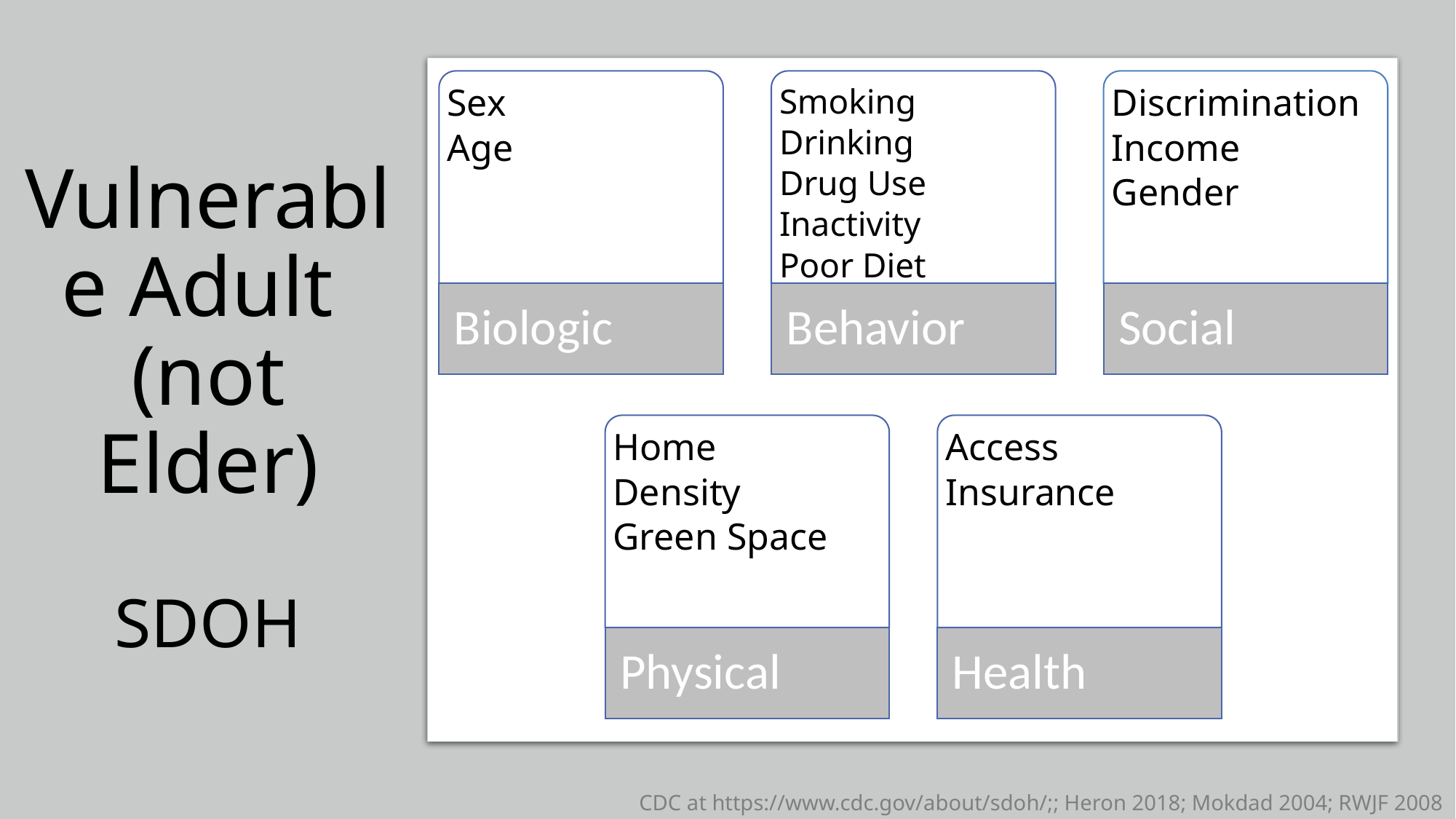

# Vulnerable Adult (not Elder) SDOH
Sex
Age
Smoking
Drinking
Drug Use
Inactivity
Poor Diet
Discrimination
Income
Gender
Biologic
Behavior
Social
Home
Density
Green Space
Access
Insurance
Physical
Health
CDC at https://www.cdc.gov/about/sdoh/;; Heron 2018; Mokdad 2004; RWJF 2008

## Slide 62
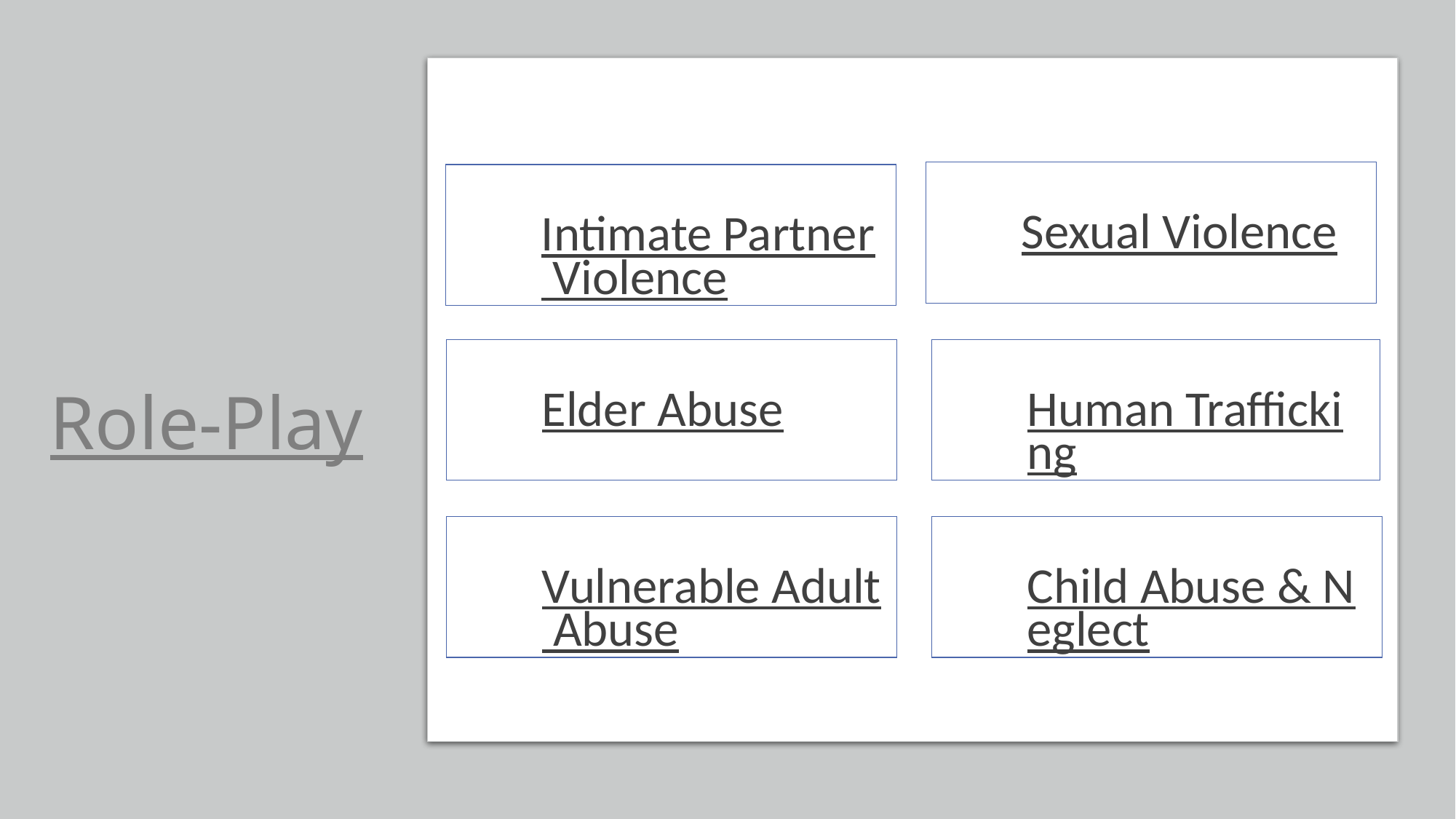

Sexual Violence
Intimate Partner Violence
# Role-Play
Elder Abuse
Human Trafficking
Vulnerable Adult Abuse
Child Abuse & Neglect

## Slide 63
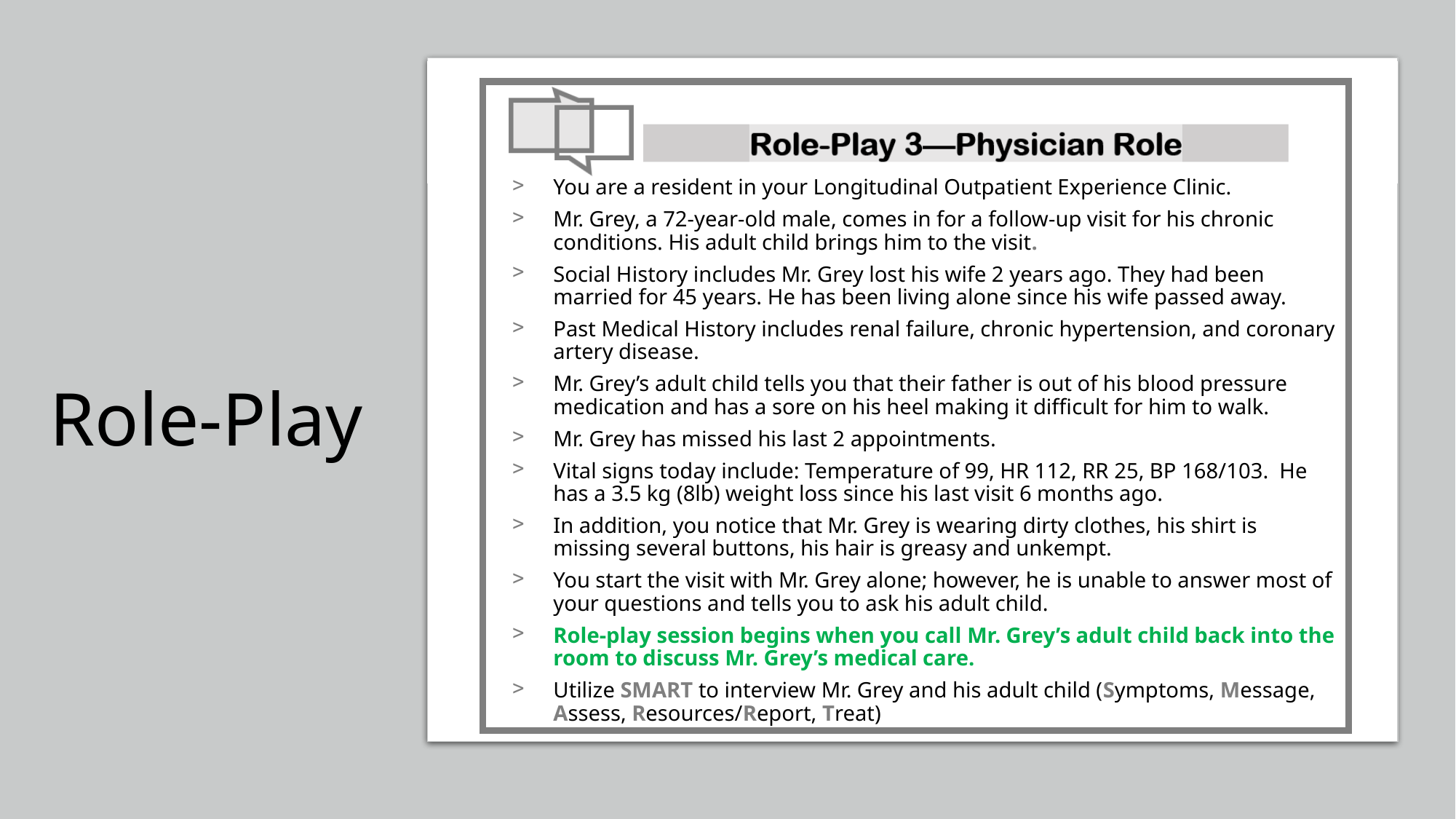

You are a resident in your Longitudinal Outpatient Experience Clinic.
Mr. Grey, a 72-year-old male, comes in for a follow-up visit for his chronic conditions. His adult child brings him to the visit.
Social History includes Mr. Grey lost his wife 2 years ago. They had been married for 45 years. He has been living alone since his wife passed away.
Past Medical History includes renal failure, chronic hypertension, and coronary artery disease.
Mr. Grey’s adult child tells you that their father is out of his blood pressure medication and has a sore on his heel making it difficult for him to walk.
Mr. Grey has missed his last 2 appointments.
Vital signs today include: Temperature of 99, HR 112, RR 25, BP 168/103. He has a 3.5 kg (8lb) weight loss since his last visit 6 months ago.
In addition, you notice that Mr. Grey is wearing dirty clothes, his shirt is missing several buttons, his hair is greasy and unkempt.
You start the visit with Mr. Grey alone; however, he is unable to answer most of your questions and tells you to ask his adult child.
Role-play session begins when you call Mr. Grey’s adult child back into the room to discuss Mr. Grey’s medical care.
Utilize SMART to interview Mr. Grey and his adult child (Symptoms, Message, Assess, Resources/Report, Treat)
# Role-Play

## Slide 64
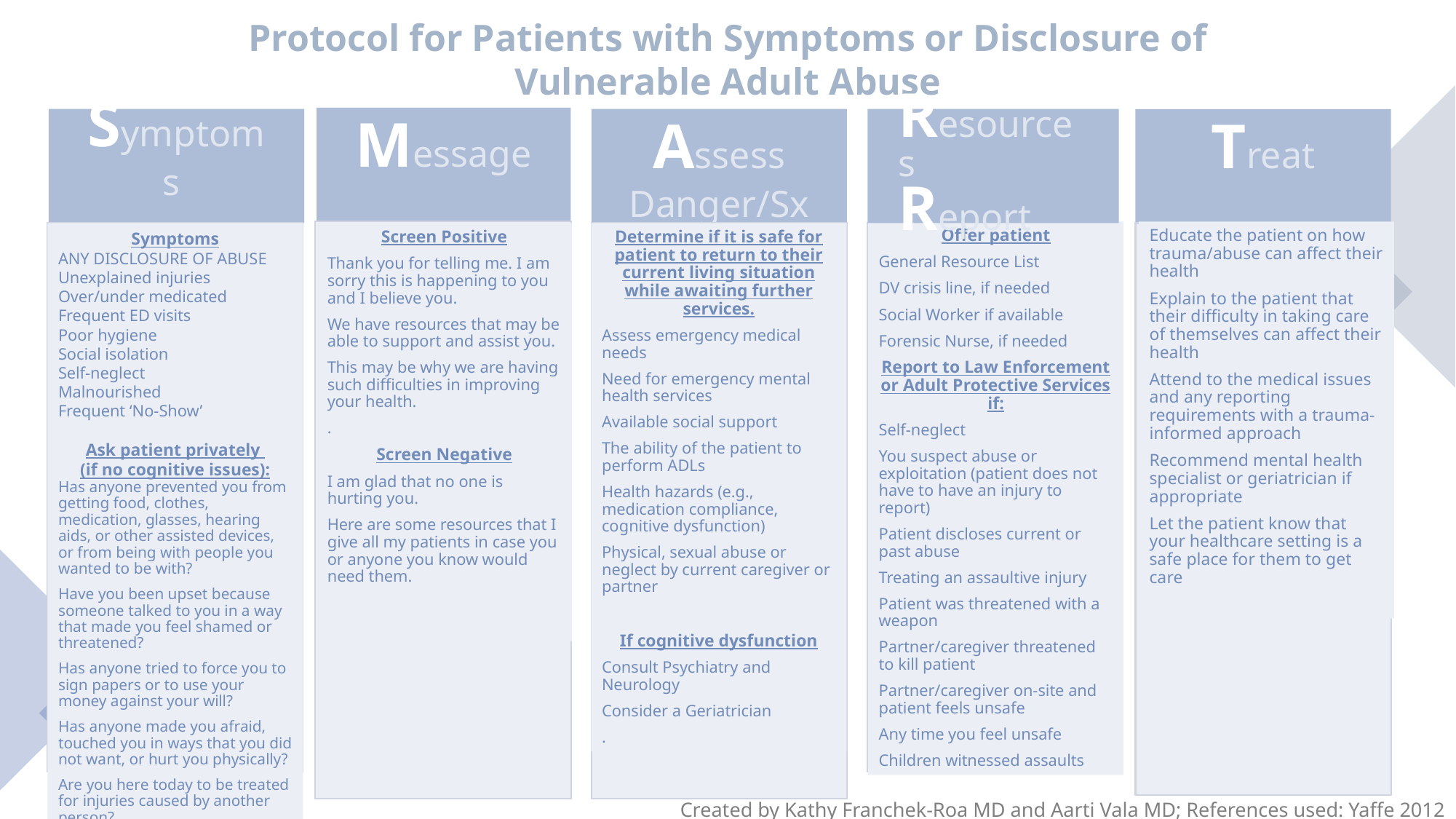

Protocol for Patients with Symptoms or Disclosure of
Vulnerable Adult Abuse
Message
Danger/Sx
Symptoms
Assess
Danger/Sx
Resources
Report
Treat
Danger/Sx
Educate the patient on how trauma/abuse can affect their health
Explain to the patient that their difficulty in taking care of themselves can affect their health
Attend to the medical issues and any reporting requirements with a trauma-informed approach
Recommend mental health specialist or geriatrician if appropriate
Let the patient know that your healthcare setting is a safe place for them to get care
Screen Positive
Thank you for telling me. I am sorry this is happening to you and I believe you.
We have resources that may be able to support and assist you.
This may be why we are having such difficulties in improving your health.
.
Screen Negative
I am glad that no one is hurting you.
Here are some resources that I give all my patients in case you or anyone you know would need them.
Offer patient
General Resource List
DV crisis line, if needed
Social Worker if available
Forensic Nurse, if needed
Report to Law Enforcement or Adult Protective Services if:
Self-neglect
You suspect abuse or exploitation (patient does not have to have an injury to report)
Patient discloses current or past abuse
Treating an assaultive injury
Patient was threatened with a weapon
Partner/caregiver threatened to kill patient
Partner/caregiver on-site and patient feels unsafe
Any time you feel unsafe
Children witnessed assaults
Symptoms
ANY DISCLOSURE OF ABUSE Unexplained injuries
Over/under medicated
Frequent ED visits
Poor hygiene
Social isolation
Self-neglect
Malnourished
Frequent ‘No-Show’
Ask patient privately
(if no cognitive issues):
Has anyone prevented you from getting food, clothes, medication, glasses, hearing aids, or other assisted devices, or from being with people you wanted to be with?
Have you been upset because someone talked to you in a way that made you feel shamed or threatened?
Has anyone tried to force you to sign papers or to use your money against your will?
Has anyone made you afraid, touched you in ways that you did not want, or hurt you physically?
Are you here today to be treated for injuries caused by another person?
Determine if it is safe for patient to return to their current living situation while awaiting further services.
Assess emergency medical needs
Need for emergency mental health services
Available social support
The ability of the patient to perform ADLs
Health hazards (e.g., medication compliance, cognitive dysfunction)
Physical, sexual abuse or neglect by current caregiver or partner
If cognitive dysfunction
Consult Psychiatry and Neurology
Consider a Geriatrician
.
Created by Kathy Franchek-Roa MD and Aarti Vala MD; References used: Yaffe 2012

## Slide 65
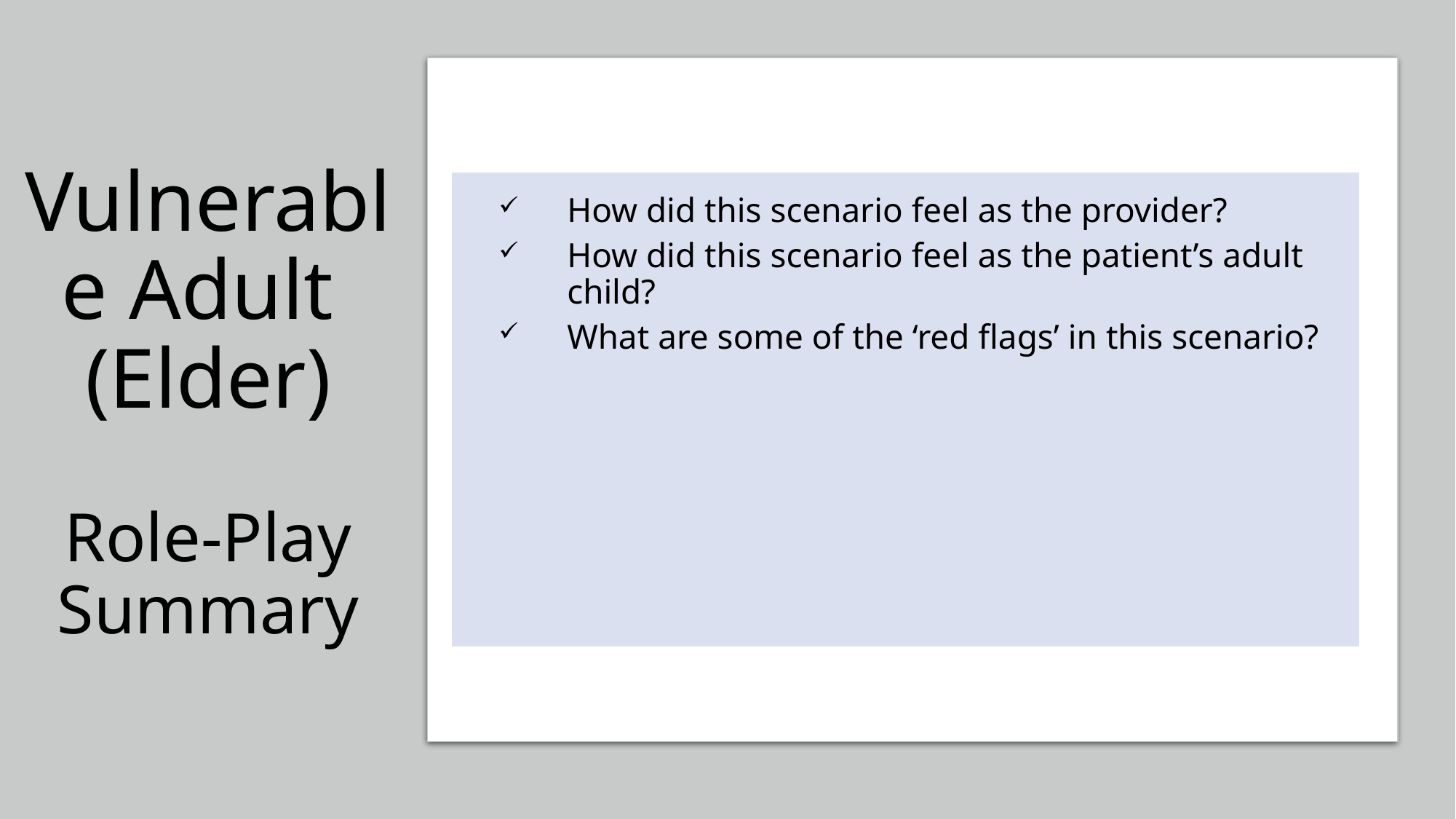

# Vulnerable Adult (Elder) Role-Play Summary
How did this scenario feel as the provider?
How did this scenario feel as the patient’s adult child?
What are some of the ‘red flags’ in this scenario?

## Slide 66
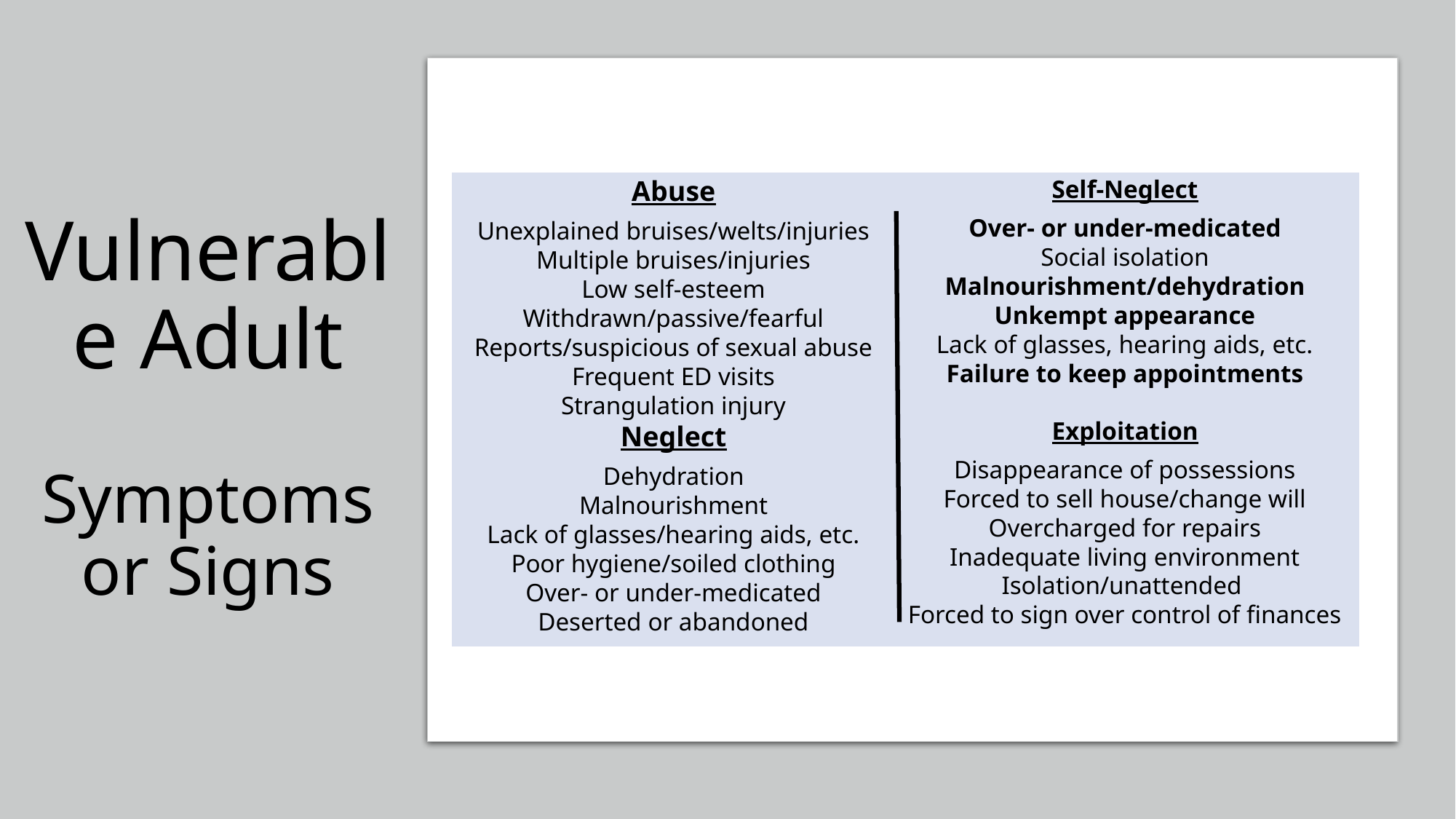

# Vulnerable Adult Symptoms or Signs
Abuse
Unexplained bruises/welts/injuries
Multiple bruises/injuries
Low self-esteem
Withdrawn/passive/fearful
Reports/suspicious of sexual abuse
Frequent ED visits
Strangulation injury
Neglect
Dehydration
Malnourishment
Lack of glasses/hearing aids, etc.
Poor hygiene/soiled clothing
Over- or under-medicated
Deserted or abandoned
Self-Neglect
Over- or under-medicated
Social isolation
Malnourishment/dehydration
Unkempt appearance
Lack of glasses, hearing aids, etc.
Failure to keep appointments
Exploitation
Disappearance of possessions
Forced to sell house/change will
Overcharged for repairs
Inadequate living environment
Isolation/unattended
Forced to sign over control of finances

## Slide 67
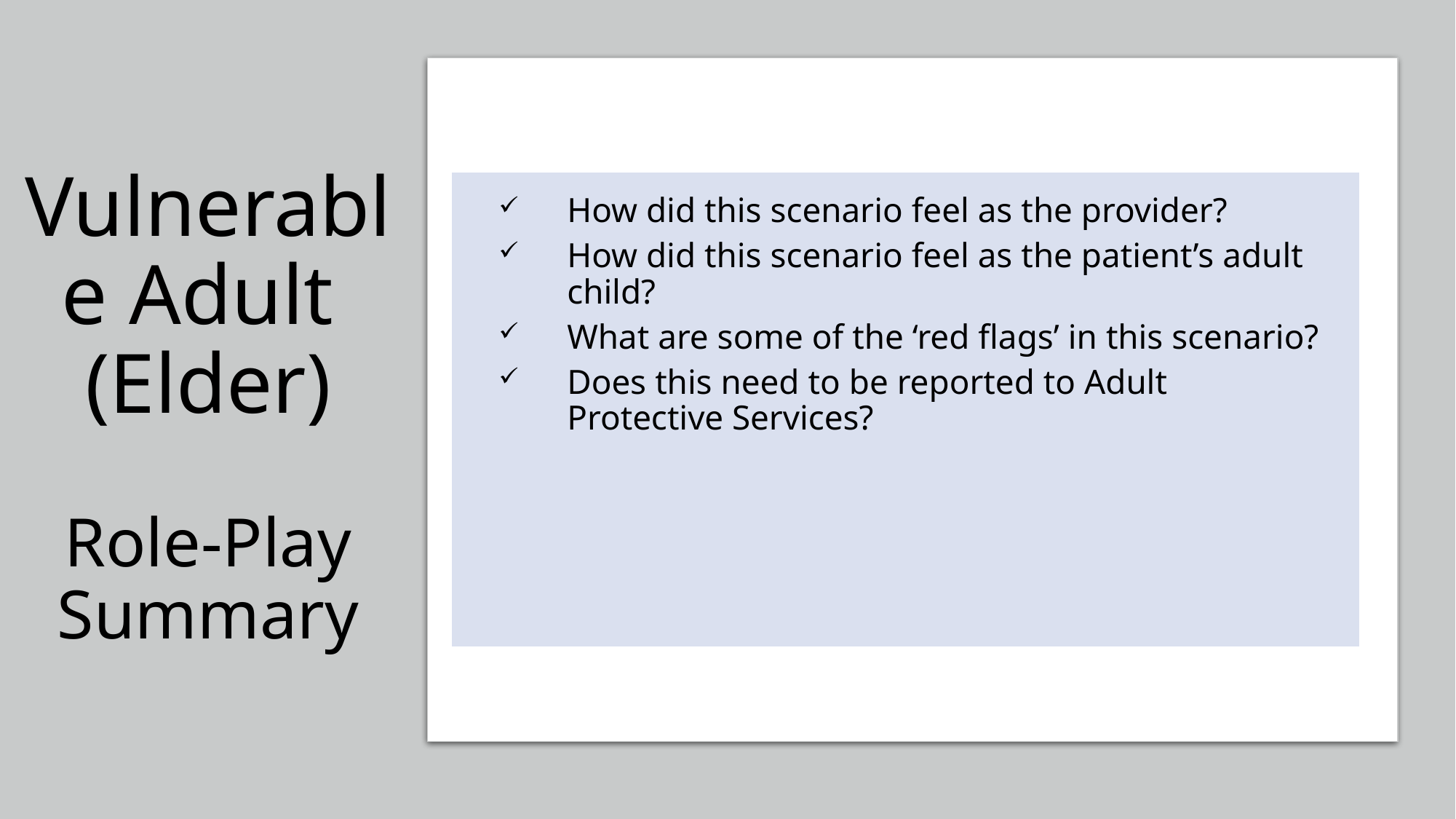

# Vulnerable Adult (Elder) Role-Play Summary
How did this scenario feel as the provider?
How did this scenario feel as the patient’s adult child?
What are some of the ‘red flags’ in this scenario?
Does this need to be reported to Adult Protective Services?

## Slide 68
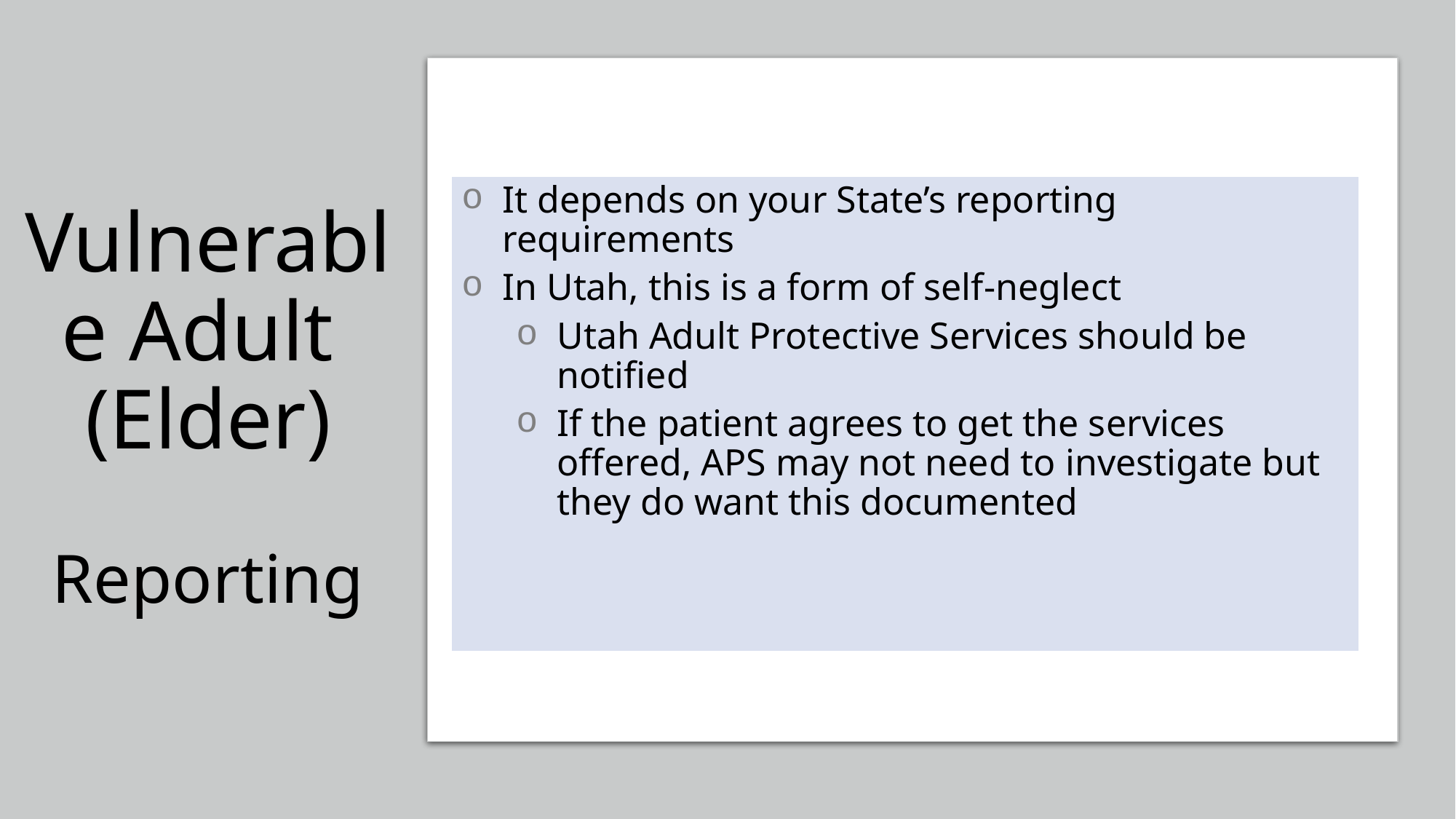

# Vulnerable Adult (Elder) Reporting
It depends on your State’s reporting requirements
In Utah, this is a form of self-neglect
Utah Adult Protective Services should be notified
If the patient agrees to get the services offered, APS may not need to investigate but they do want this documented

## Slide 69
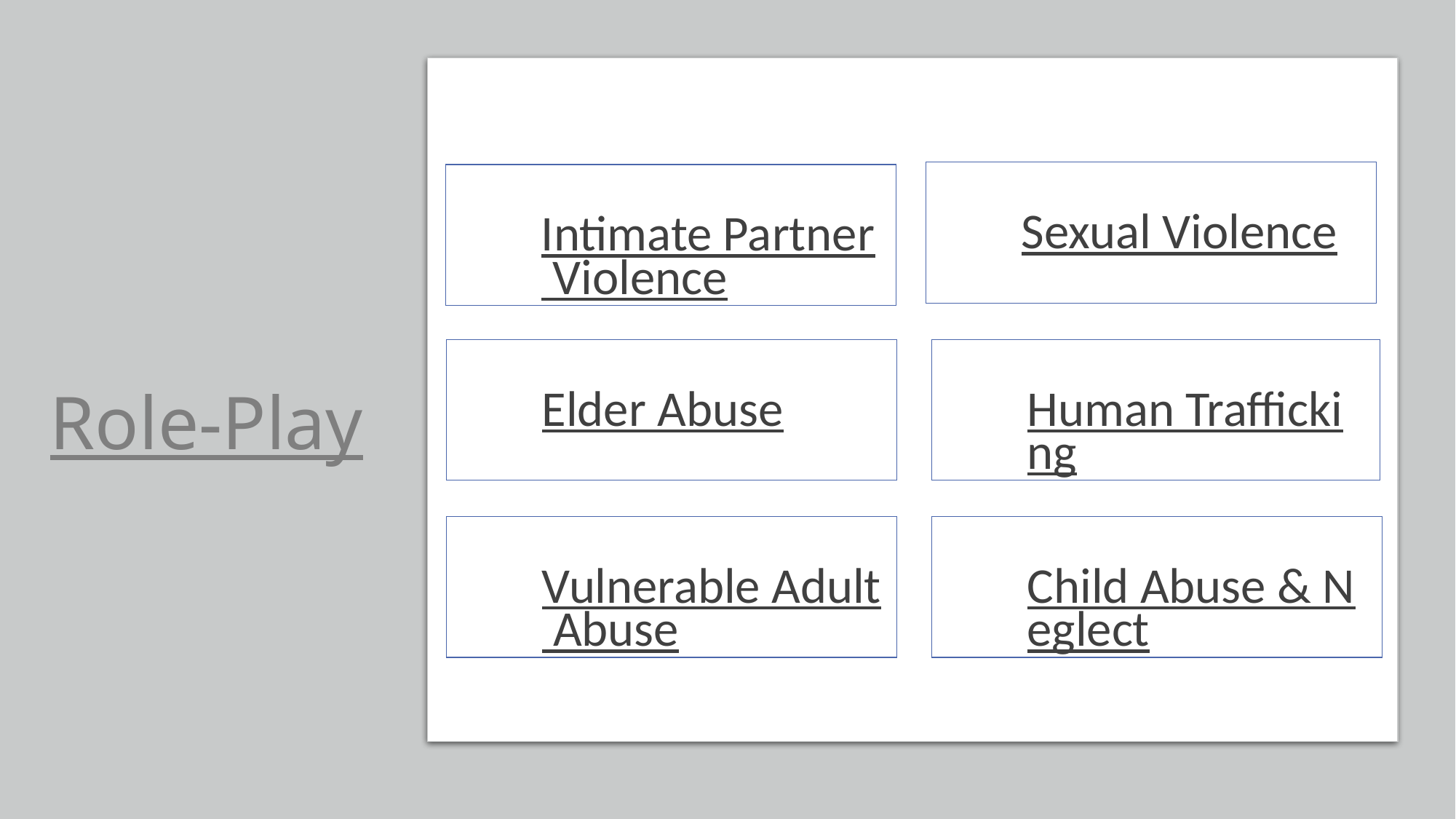

Sexual Violence
Intimate Partner Violence
# Role-Play
Elder Abuse
Human Trafficking
Vulnerable Adult Abuse
Child Abuse & Neglect

## Slide 70
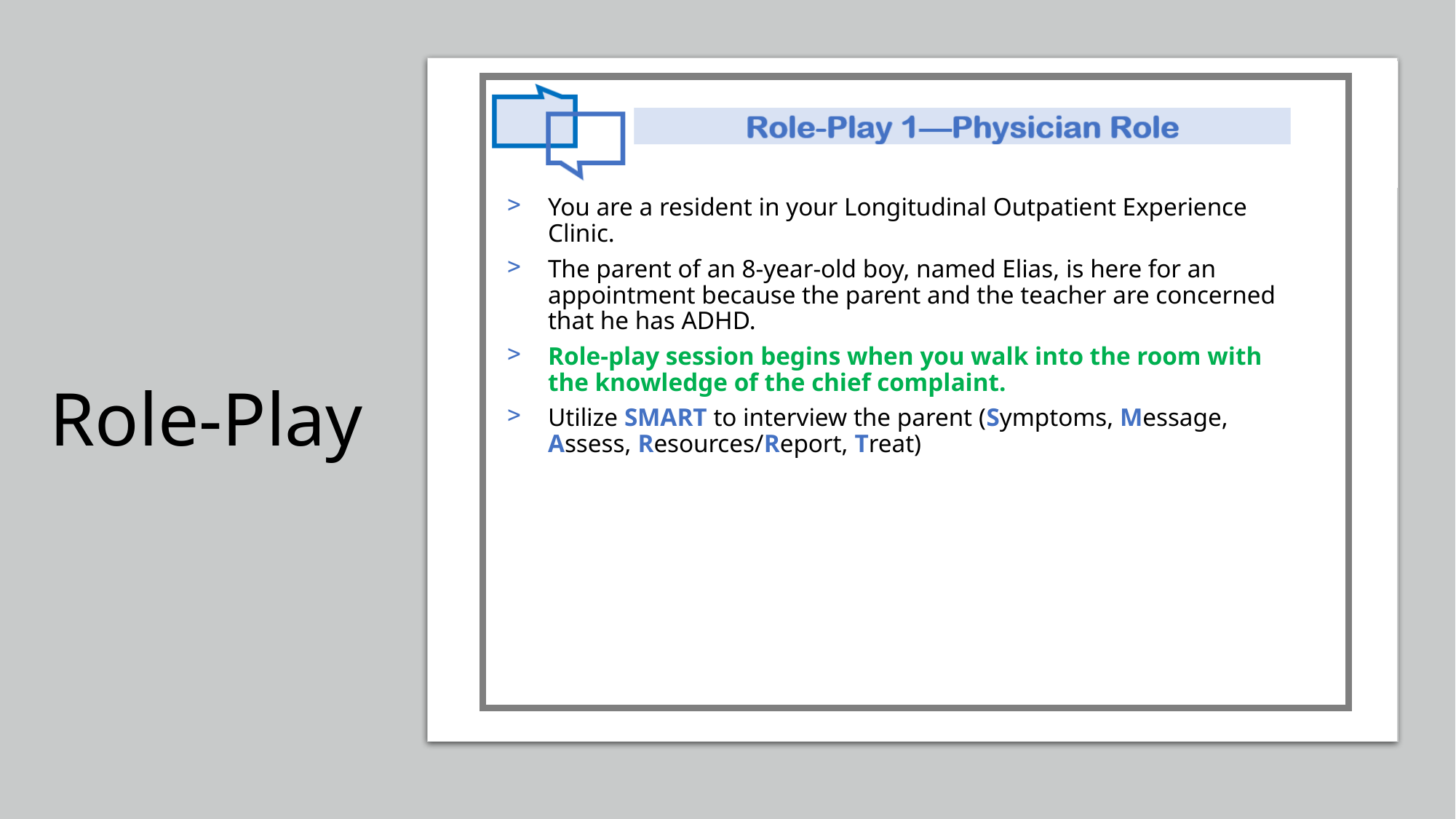

You are a resident in your Longitudinal Outpatient Experience Clinic.
The parent of an 8-year-old boy, named Elias, is here for an appointment because the parent and the teacher are concerned that he has ADHD.
Role-play session begins when you walk into the room with the knowledge of the chief complaint.
Utilize SMART to interview the parent (Symptoms, Message, Assess, Resources/Report, Treat)
# Role-Play

## Slide 71
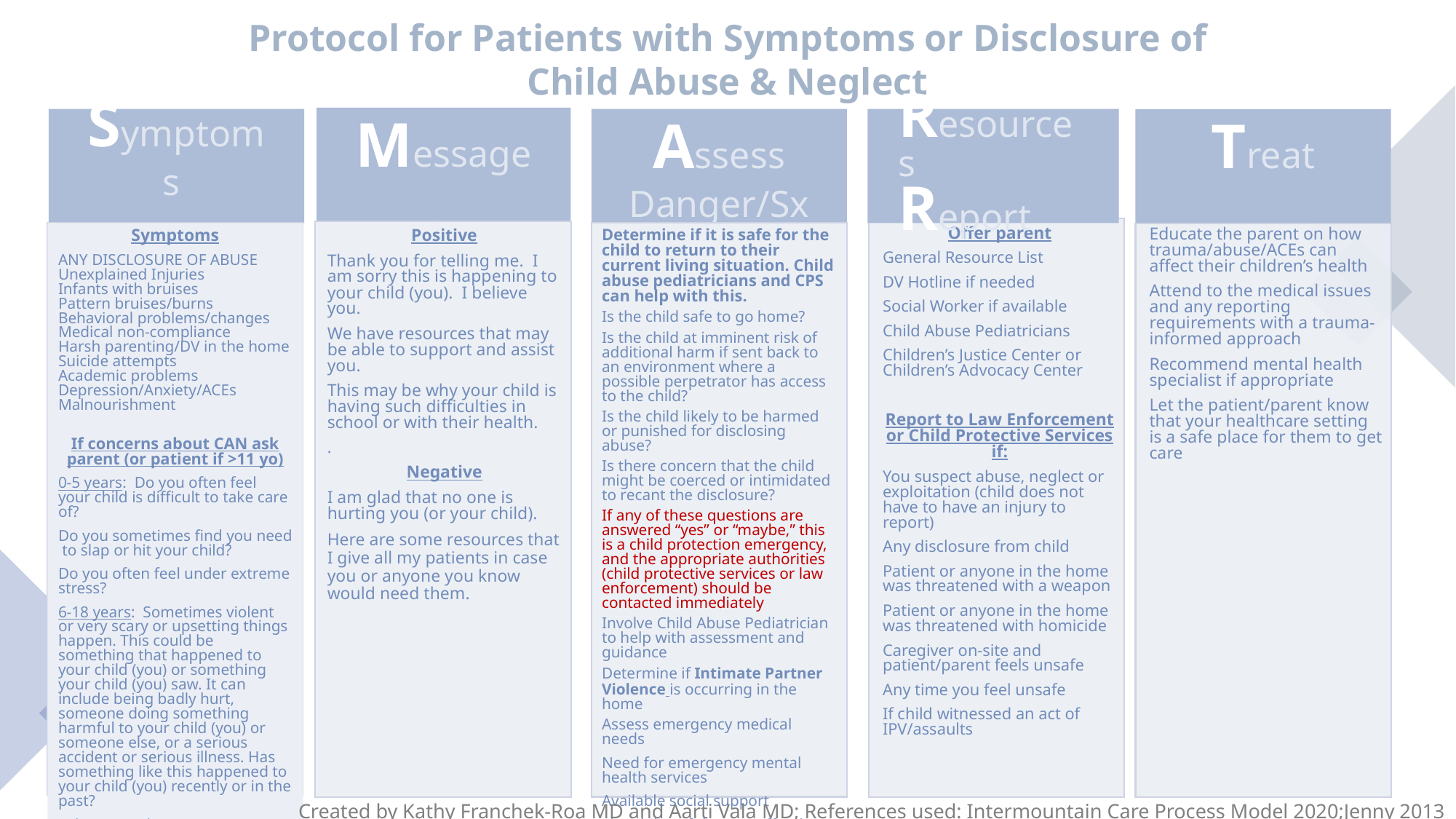

Protocol for Patients with Symptoms or Disclosure of
Child Abuse & Neglect
Message
Danger/Sx
Symptoms
Assess
Danger/Sx
Resources
Report
Treat
Danger/Sx
Offer parent
General Resource List
DV Hotline if needed
Social Worker if available
Child Abuse Pediatricians
Children’s Justice Center or Children’s Advocacy Center
Report to Law Enforcement or Child Protective Services if:
You suspect abuse, neglect or exploitation (child does not have to have an injury to report)
Any disclosure from child
Patient or anyone in the home was threatened with a weapon
Patient or anyone in the home was threatened with homicide
Caregiver on-site and patient/parent feels unsafe
Any time you feel unsafe
If child witnessed an act of IPV/assaults
Educate the parent on how trauma/abuse/ACEs can affect their children’s health
Attend to the medical issues and any reporting requirements with a trauma-informed approach
Recommend mental health specialist if appropriate
Let the patient/parent know that your healthcare setting is a safe place for them to get care
Positive
Thank you for telling me. I am sorry this is happening to your child (you). I believe you.
We have resources that may be able to support and assist you.
This may be why your child is having such difficulties in school or with their health.
.
Negative
I am glad that no one is hurting you (or your child).
Here are some resources that I give all my patients in case you or anyone you know would need them.
Symptoms
ANY DISCLOSURE OF ABUSE
Unexplained Injuries
Infants with bruises
Pattern bruises/burns
Behavioral problems/changes
Medical non-compliance
Harsh parenting/DV in the home
Suicide attempts
Academic problems
Depression/Anxiety/ACEs
Malnourishment
If concerns about CAN ask parent (or patient if >11 yo)
0-5 years: Do you often feel your child is difficult to take care of?
Do you sometimes find you need to slap or hit your child?
Do you often feel under extreme stress?
6-18 years: Sometimes violent or very scary or upsetting things happen. This could be something that happened to your child (you) or something your child (you) saw. It can include being badly hurt, someone doing something harmful to your child (you) or someone else, or a serious accident or serious illness. Has something like this happened to your child (you) recently or in the past?
Ask parent about IPV
Determine if it is safe for the child to return to their current living situation. Child abuse pediatricians and CPS can help with this.
Is the child safe to go home?
Is the child at imminent risk of additional harm if sent back to an environment where a possible perpetrator has access to the child?
Is the child likely to be harmed or punished for disclosing abuse?
Is there concern that the child might be coerced or intimidated to recant the disclosure?
If any of these questions are answered “yes” or “maybe,” this is a child protection emergency, and the appropriate authorities (child protective services or law enforcement) should be contacted immediately
Involve Child Abuse Pediatrician to help with assessment and guidance
Determine if Intimate Partner Violence is occurring in the home
Assess emergency medical needs
Need for emergency mental health services
Available social support
The ability of the non-offending parent to care for the child
Created by Kathy Franchek-Roa MD and Aarti Vala MD; References used: Intermountain Care Process Model 2020;Jenny 2013

## Slide 72
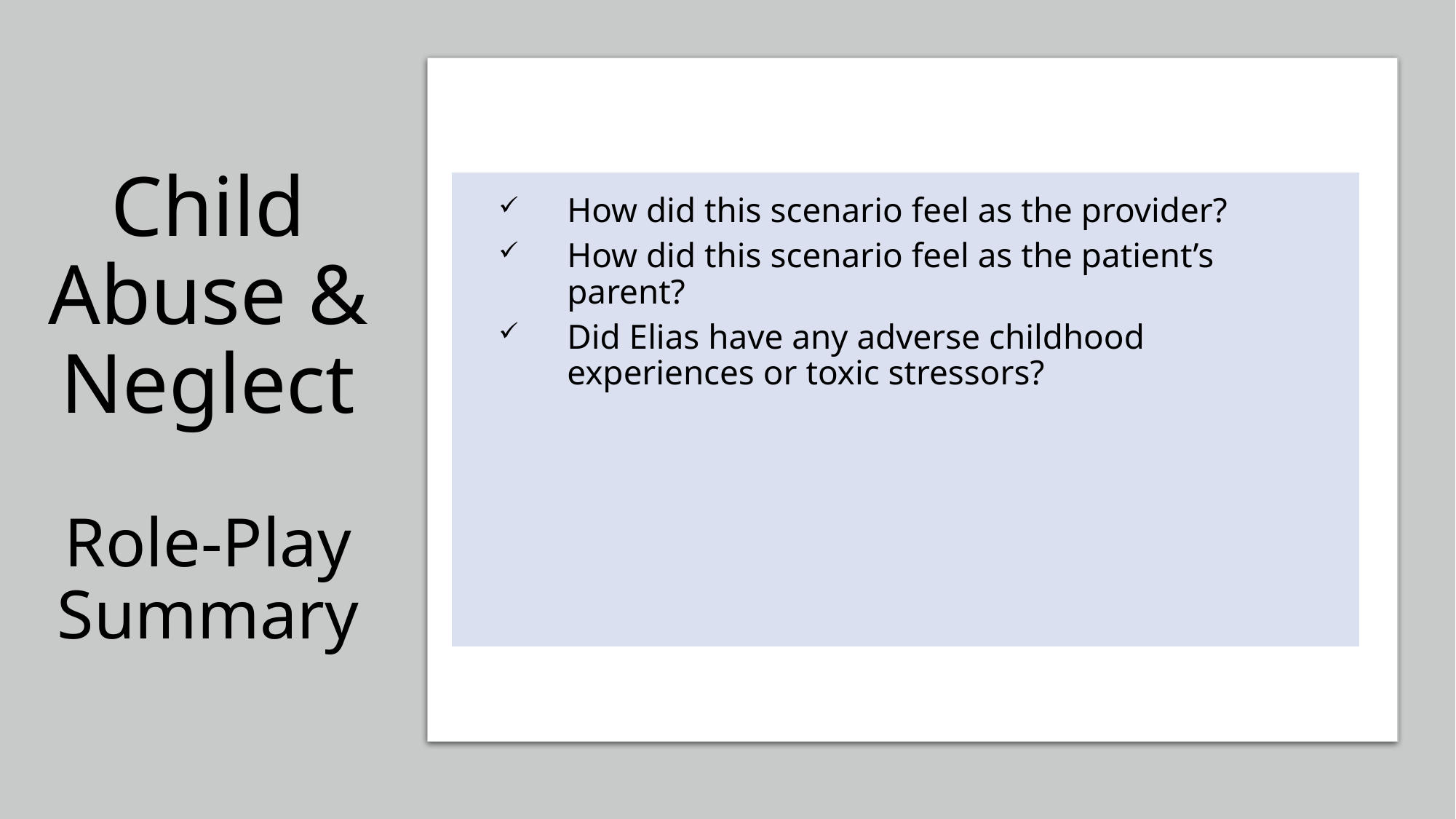

# Child Abuse & Neglect Role-Play Summary
How did this scenario feel as the provider?
How did this scenario feel as the patient’s parent?
Did Elias have any adverse childhood experiences or toxic stressors?

## Slide 73
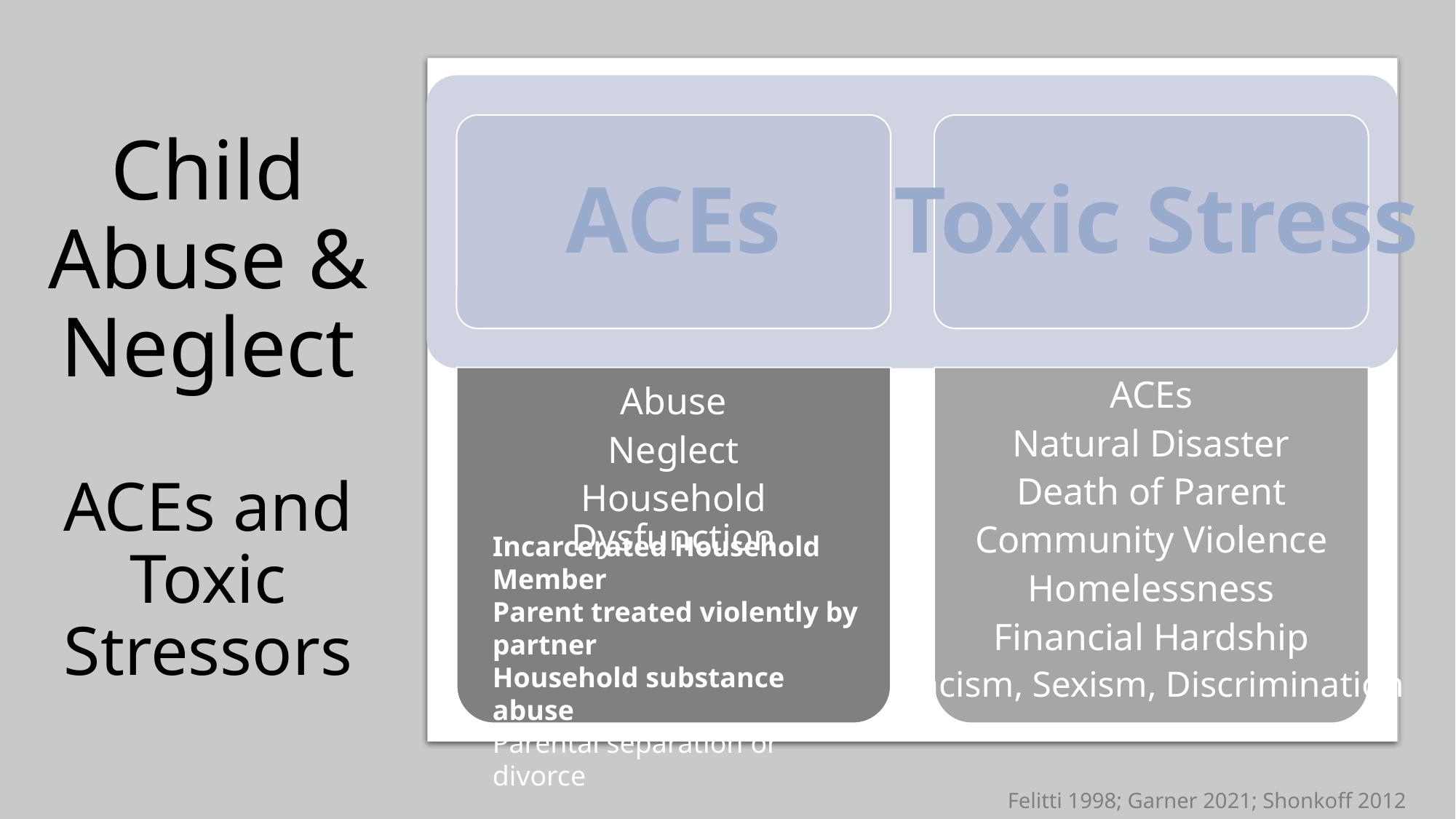

# Child Abuse & Neglect ACEs and Toxic Stressors
ACEs
Toxic Stress
Abuse
Neglect
Household Dysfunction
ACEs
Natural Disaster
Death of Parent
Community Violence
Homelessness
Financial Hardship
Racism, Sexism, Discrimination
Incarcerated Household Member
Parent treated violently by partner
Household substance abuse
Parental separation or divorce
Felitti 1998; Garner 2021; Shonkoff 2012

## Slide 74
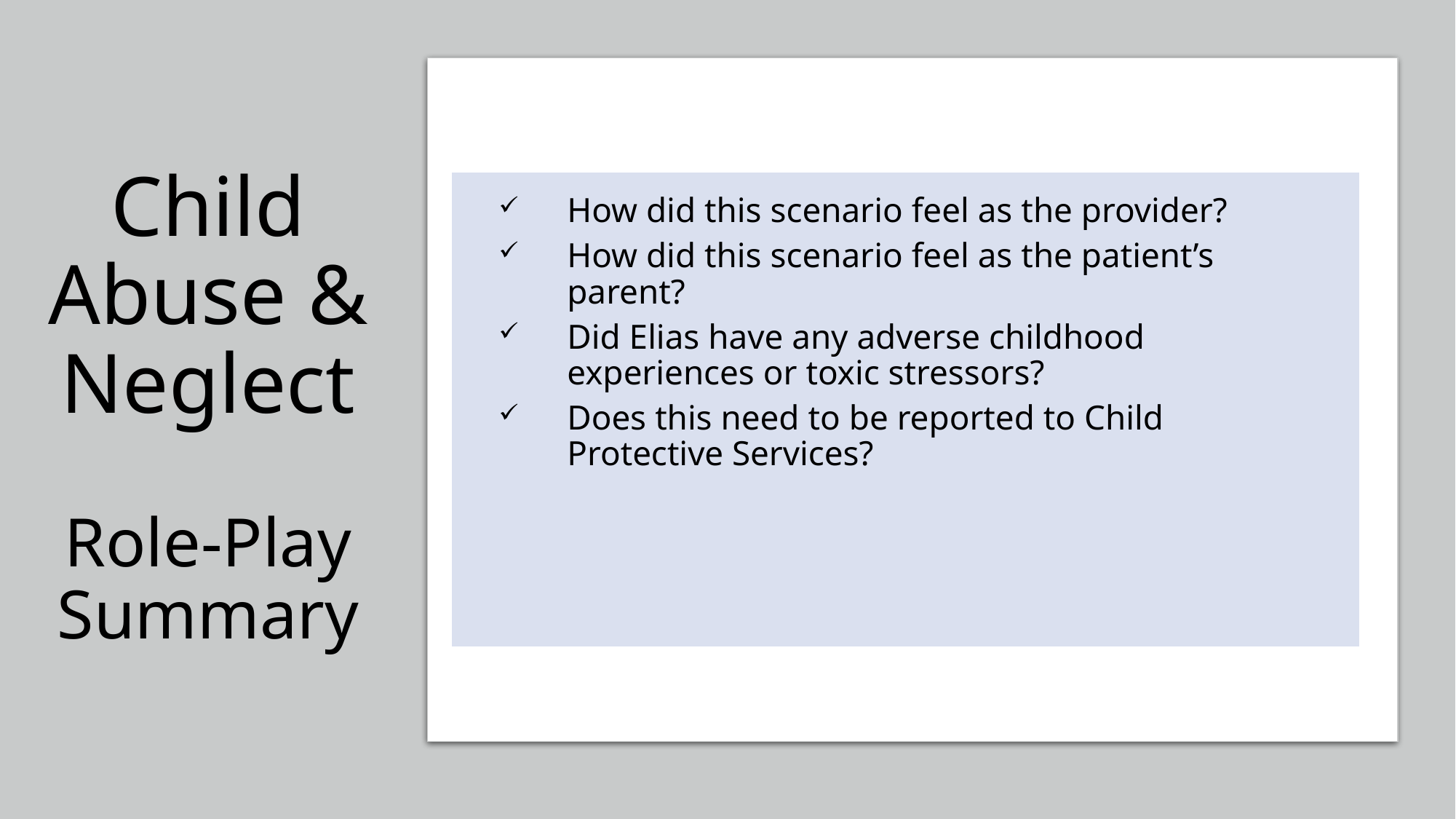

# Child Abuse & Neglect Role-Play Summary
How did this scenario feel as the provider?
How did this scenario feel as the patient’s parent?
Did Elias have any adverse childhood experiences or toxic stressors?
Does this need to be reported to Child Protective Services?

## Slide 75
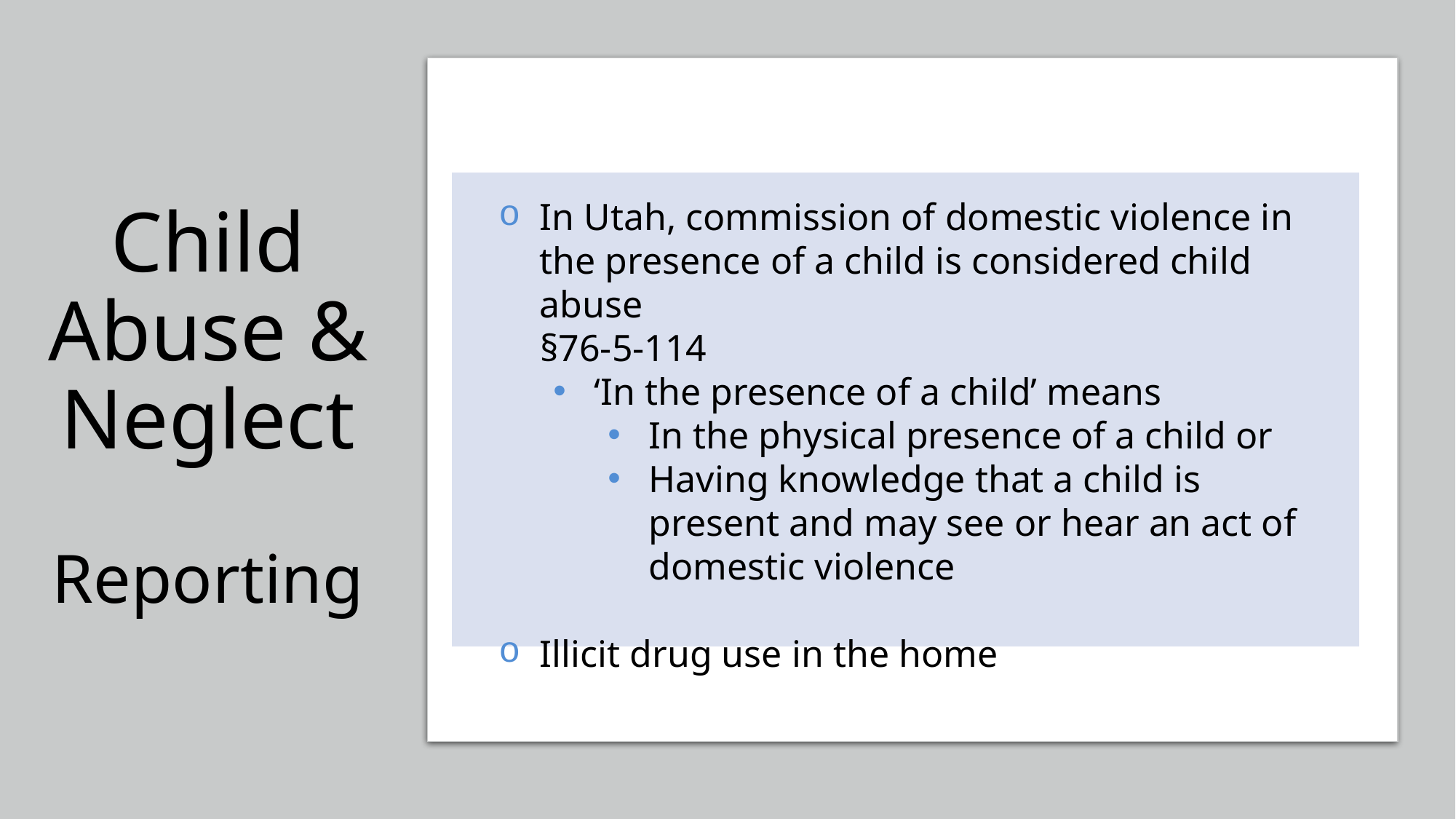

# Child Abuse & Neglect Reporting
In Utah, commission of domestic violence in the presence of a child is considered child abuse
	§76-5-114
‘In the presence of a child’ means
In the physical presence of a child or
Having knowledge that a child is present and may see or hear an act of domestic violence
Illicit drug use in the home

## Slide 76
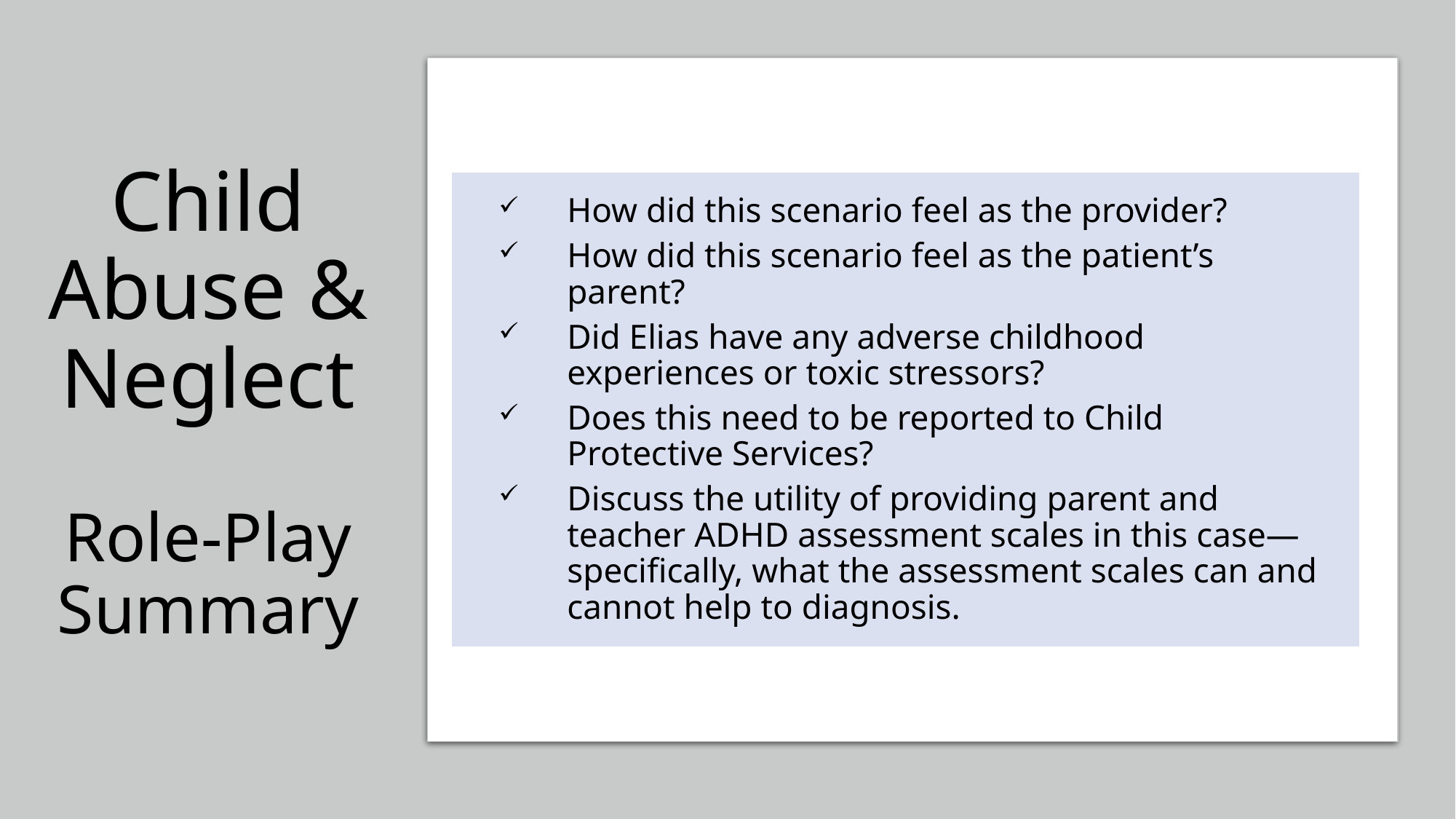

# Child Abuse & Neglect Role-Play Summary
How did this scenario feel as the provider?
How did this scenario feel as the patient’s parent?
Did Elias have any adverse childhood experiences or toxic stressors?
Does this need to be reported to Child Protective Services?
Discuss the utility of providing parent and teacher ADHD assessment scales in this case—specifically, what the assessment scales can and cannot help to diagnosis.

## Slide 77
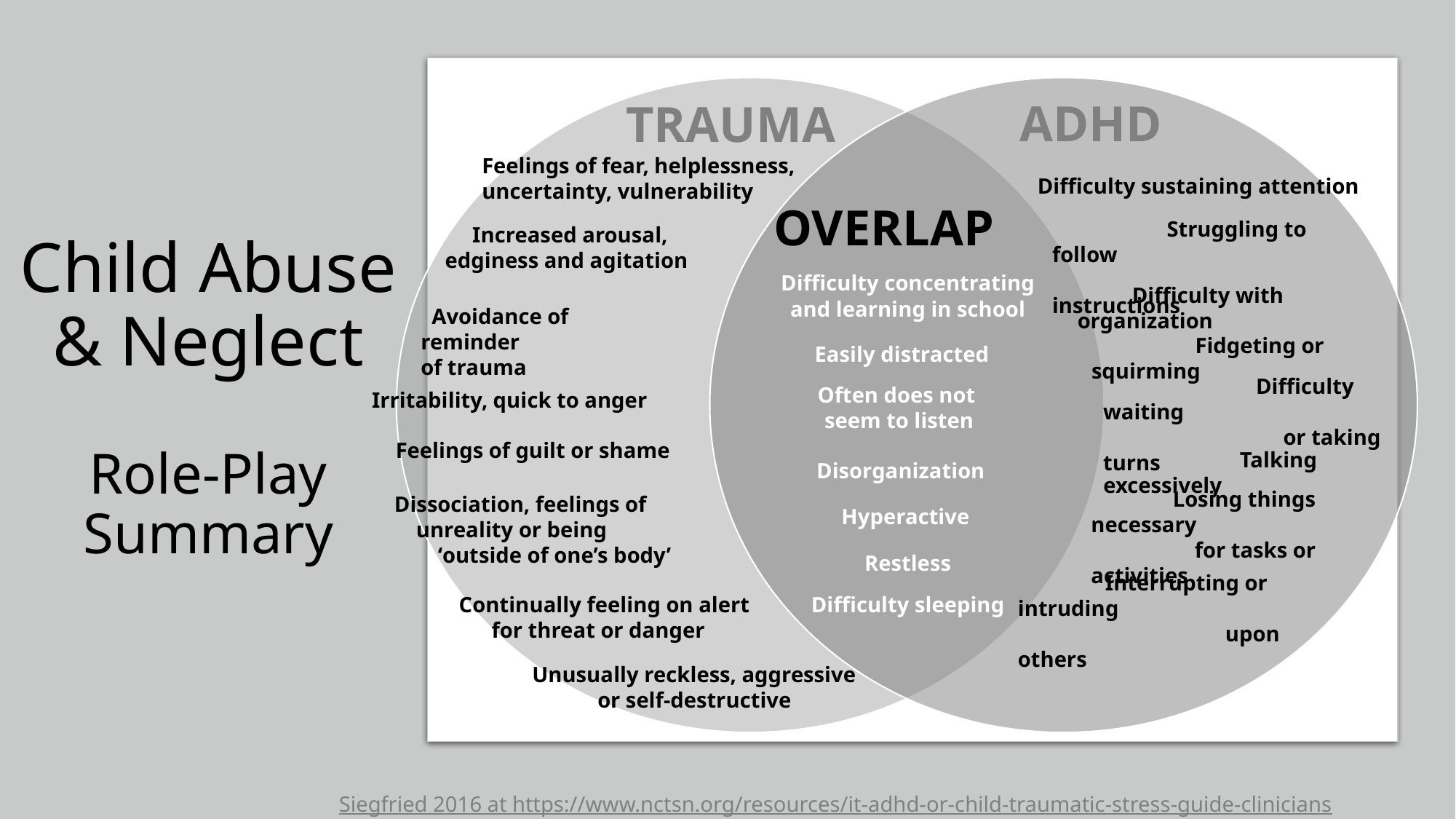

ADHD
TRAUMA
Feelings of fear, helplessness,
uncertainty, vulnerability
Difficulty sustaining attention
OVERLAP
 Struggling to follow
 instructions
 Increased arousal,
edginess and agitation
Difficulty concentrating
and learning in school
 Difficulty with organization
 Avoidance of reminder
of trauma
 Fidgeting or squirming
Easily distracted
 Difficulty waiting
 or taking turns
Often does not
seem to listen
Irritability, quick to anger
Feelings of guilt or shame
 Talking excessively
Disorganization
 Losing things necessary
 for tasks or activities
Dissociation, feelings of
 unreality or being
 ‘outside of one’s body’
Hyperactive
Restless
 Interrupting or intruding
 upon others
Difficulty sleeping
Continually feeling on alert
 for threat or danger
Unusually reckless, aggressive
 or self-destructive
# Child Abuse & Neglect Role-Play Summary
Siegfried 2016 at https://www.nctsn.org/resources/it-adhd-or-child-traumatic-stress-guide-clinicians

## Slide 78
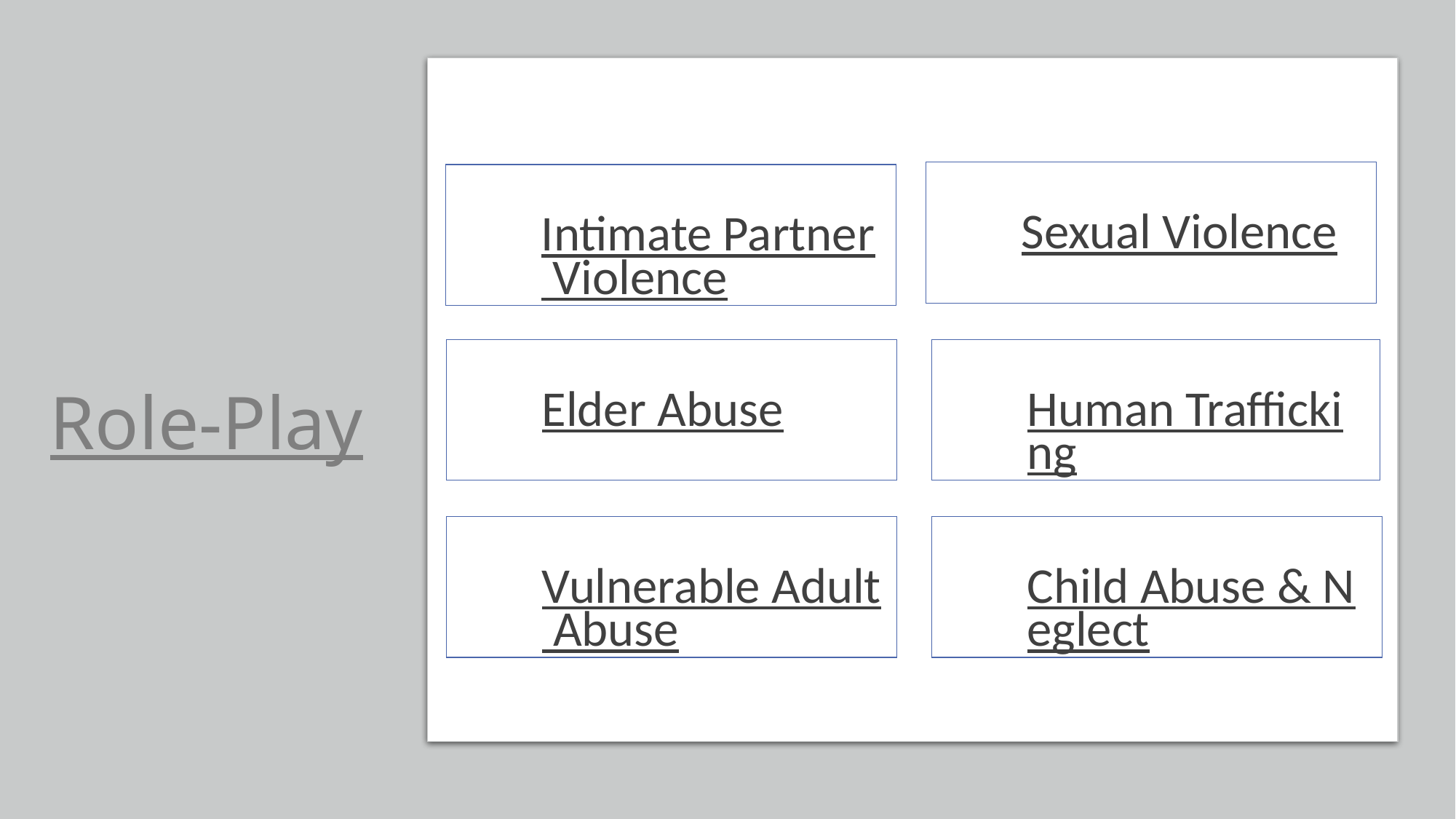

Sexual Violence
Intimate Partner Violence
# Role-Play
Elder Abuse
Human Trafficking
Vulnerable Adult Abuse
Child Abuse & Neglect

## Slide 79
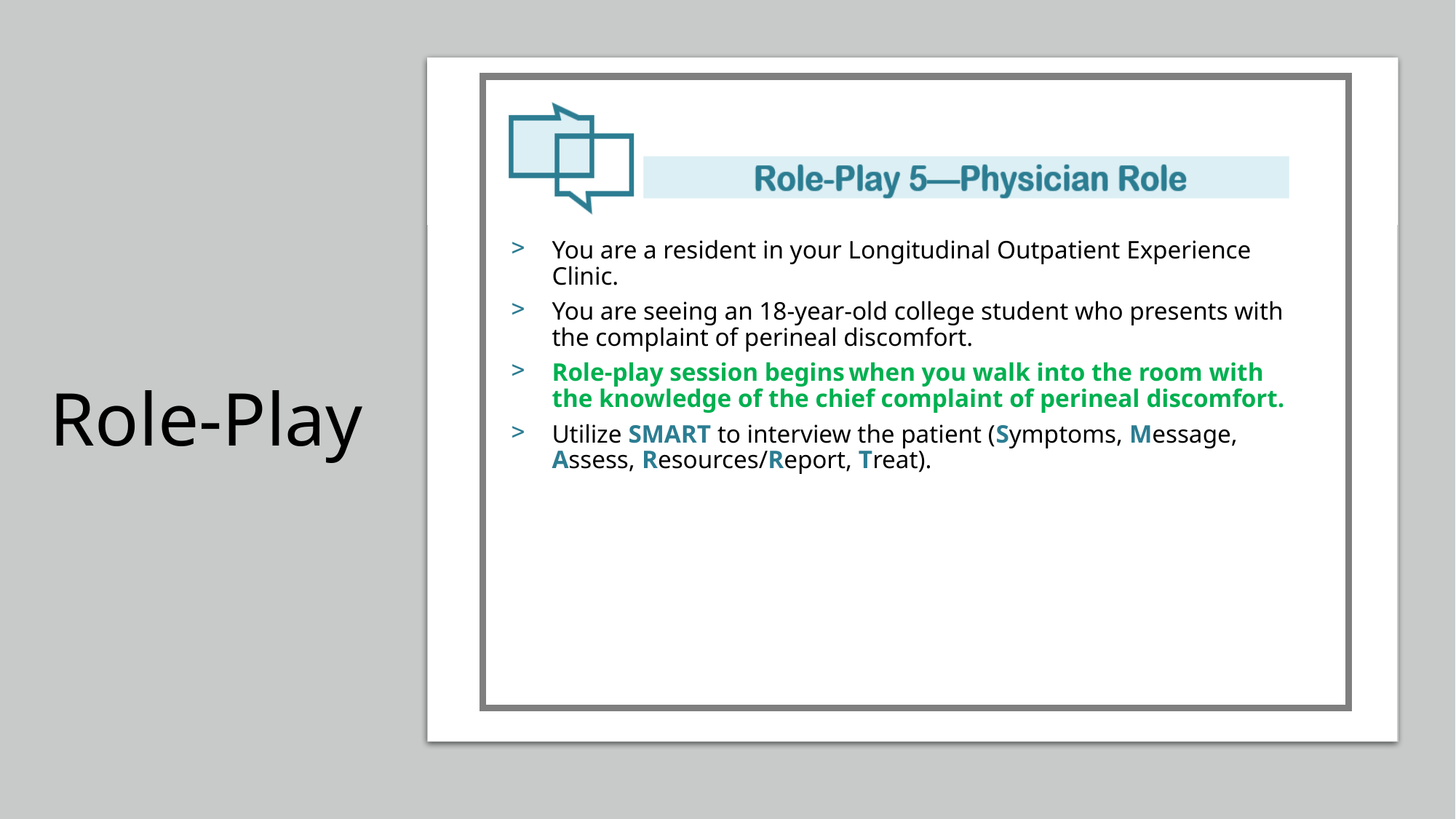

You are a resident in your Longitudinal Outpatient Experience Clinic.
You are seeing an 18-year-old college student who presents with the complaint of perineal discomfort.
Role-play session begins when you walk into the room with the knowledge of the chief complaint of perineal discomfort.
Utilize SMART to interview the patient (Symptoms, Message, Assess, Resources/Report, Treat).
# Role-Play

## Slide 80
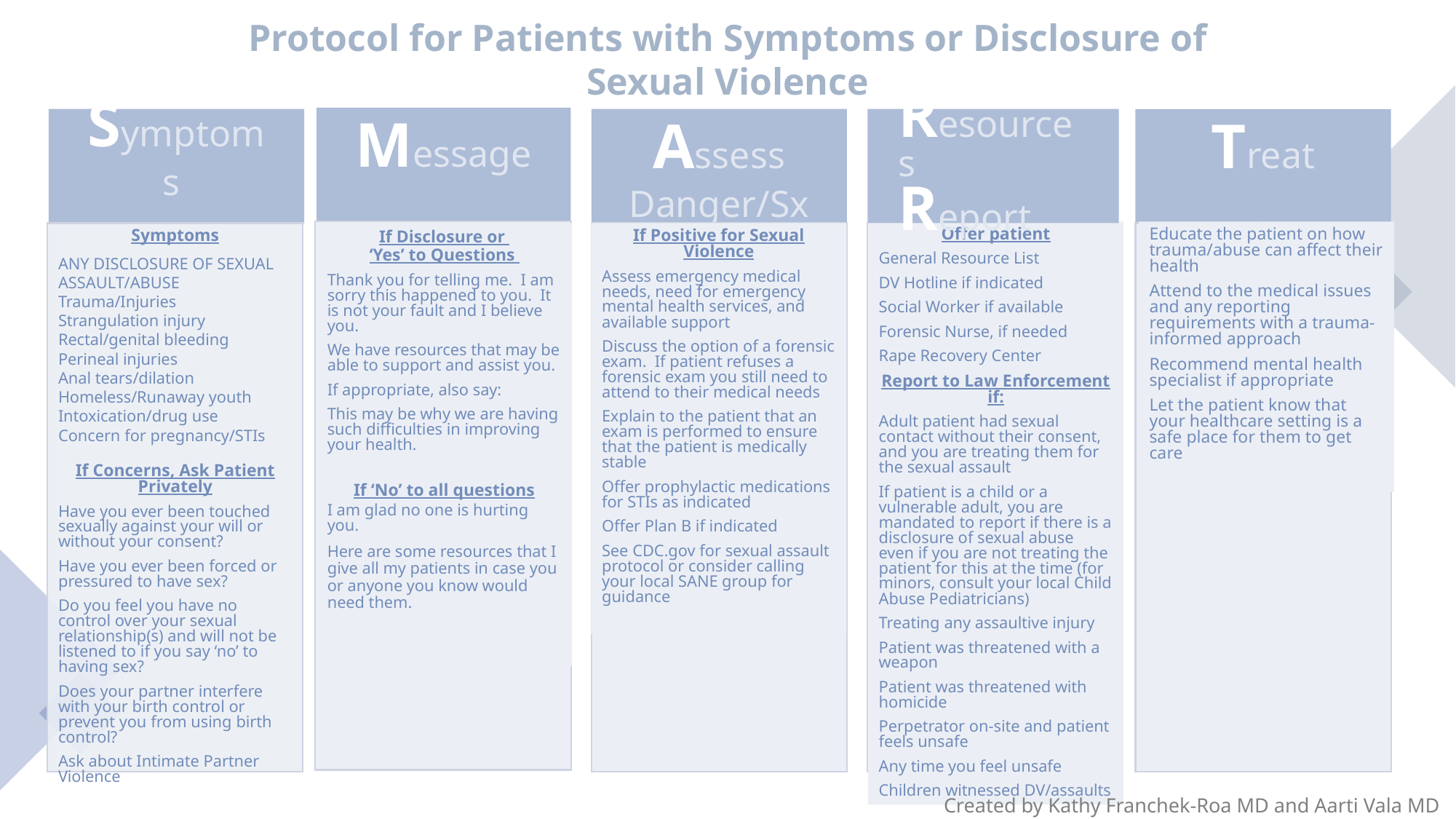

Protocol for Patients with Symptoms or Disclosure of
Sexual Violence
Message
Danger/Sx
Symptoms
Assess
Danger/Sx
Resources
Report
Treat
Danger/Sx
Educate the patient on how trauma/abuse can affect their health
Attend to the medical issues and any reporting requirements with a trauma-informed approach
Recommend mental health specialist if appropriate
Let the patient know that your healthcare setting is a safe place for them to get care
If Disclosure or
‘Yes’ to Questions
Thank you for telling me. I am sorry this happened to you. It is not your fault and I believe you.
We have resources that may be able to support and assist you.
If appropriate, also say:
This may be why we are having such difficulties in improving your health.
If ‘No’ to all questions
I am glad no one is hurting you.
Here are some resources that I give all my patients in case you or anyone you know would need them.
Offer patient
General Resource List
DV Hotline if indicated
Social Worker if available
Forensic Nurse, if needed
Rape Recovery Center
Report to Law Enforcement if:
Adult patient had sexual contact without their consent, and you are treating them for the sexual assault
If patient is a child or a vulnerable adult, you are mandated to report if there is a disclosure of sexual abuse even if you are not treating the patient for this at the time (for minors, consult your local Child Abuse Pediatricians)
Treating any assaultive injury
Patient was threatened with a weapon
Patient was threatened with homicide
Perpetrator on-site and patient feels unsafe
Any time you feel unsafe
Children witnessed DV/assaults
Symptoms
ANY DISCLOSURE OF SEXUAL ASSAULT/ABUSE
Trauma/Injuries
Strangulation injury
Rectal/genital bleeding
Perineal injuries
Anal tears/dilation
Homeless/Runaway youth
Intoxication/drug use
Concern for pregnancy/STIs
If Concerns, Ask Patient Privately
Have you ever been touched sexually against your will or without your consent?
Have you ever been forced or pressured to have sex?
Do you feel you have no control over your sexual relationship(s) and will not be listened to if you say ‘no’ to having sex?
Does your partner interfere with your birth control or prevent you from using birth control?
Ask about Intimate Partner Violence
If Positive for Sexual Violence
Assess emergency medical needs, need for emergency mental health services, and available support
Discuss the option of a forensic exam. If patient refuses a forensic exam you still need to attend to their medical needs
Explain to the patient that an exam is performed to ensure that the patient is medically stable
Offer prophylactic medications for STIs as indicated
Offer Plan B if indicated
See CDC.gov for sexual assault protocol or consider calling your local SANE group for guidance
Created by Kathy Franchek-Roa MD and Aarti Vala MD

## Slide 81
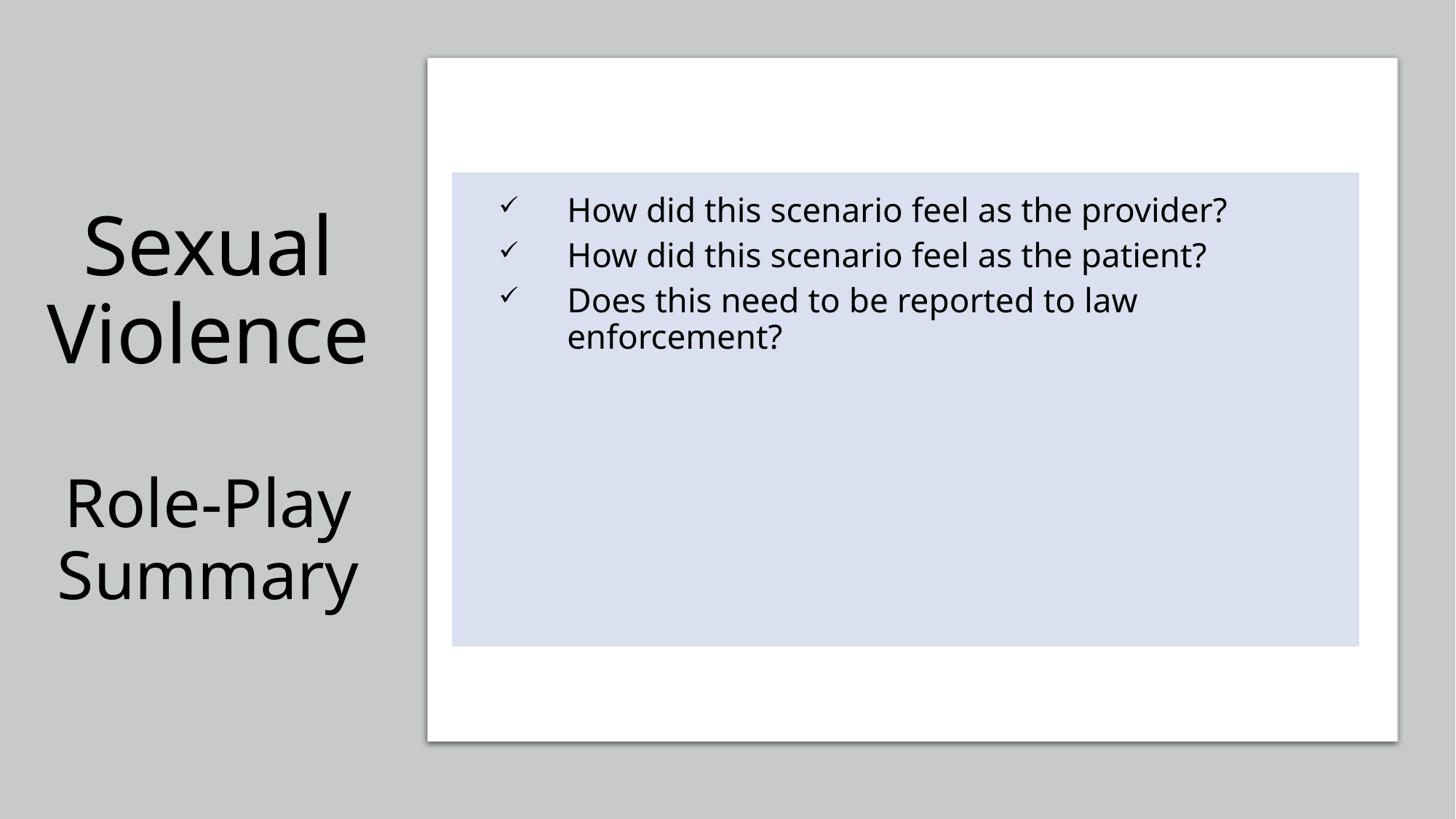

# Sexual Violence Role-Play Summary
How did this scenario feel as the provider?
How did this scenario feel as the patient?
Does this need to be reported to law enforcement?

## Slide 82
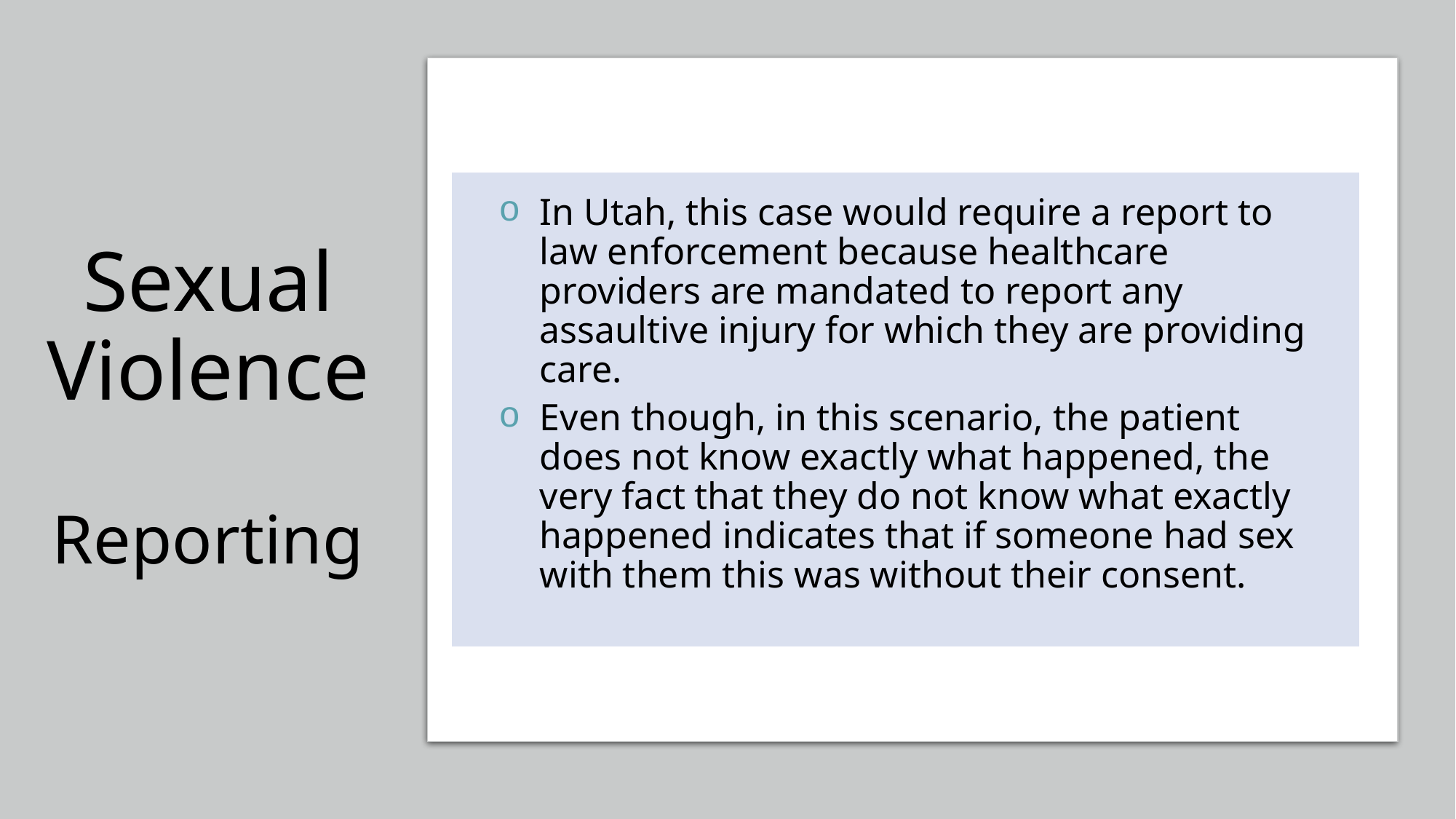

# Sexual Violence Reporting
In Utah, this case would require a report to law enforcement because healthcare providers are mandated to report any assaultive injury for which they are providing care.
Even though, in this scenario, the patient does not know exactly what happened, the very fact that they do not know what exactly happened indicates that if someone had sex with them this was without their consent.

## Slide 83
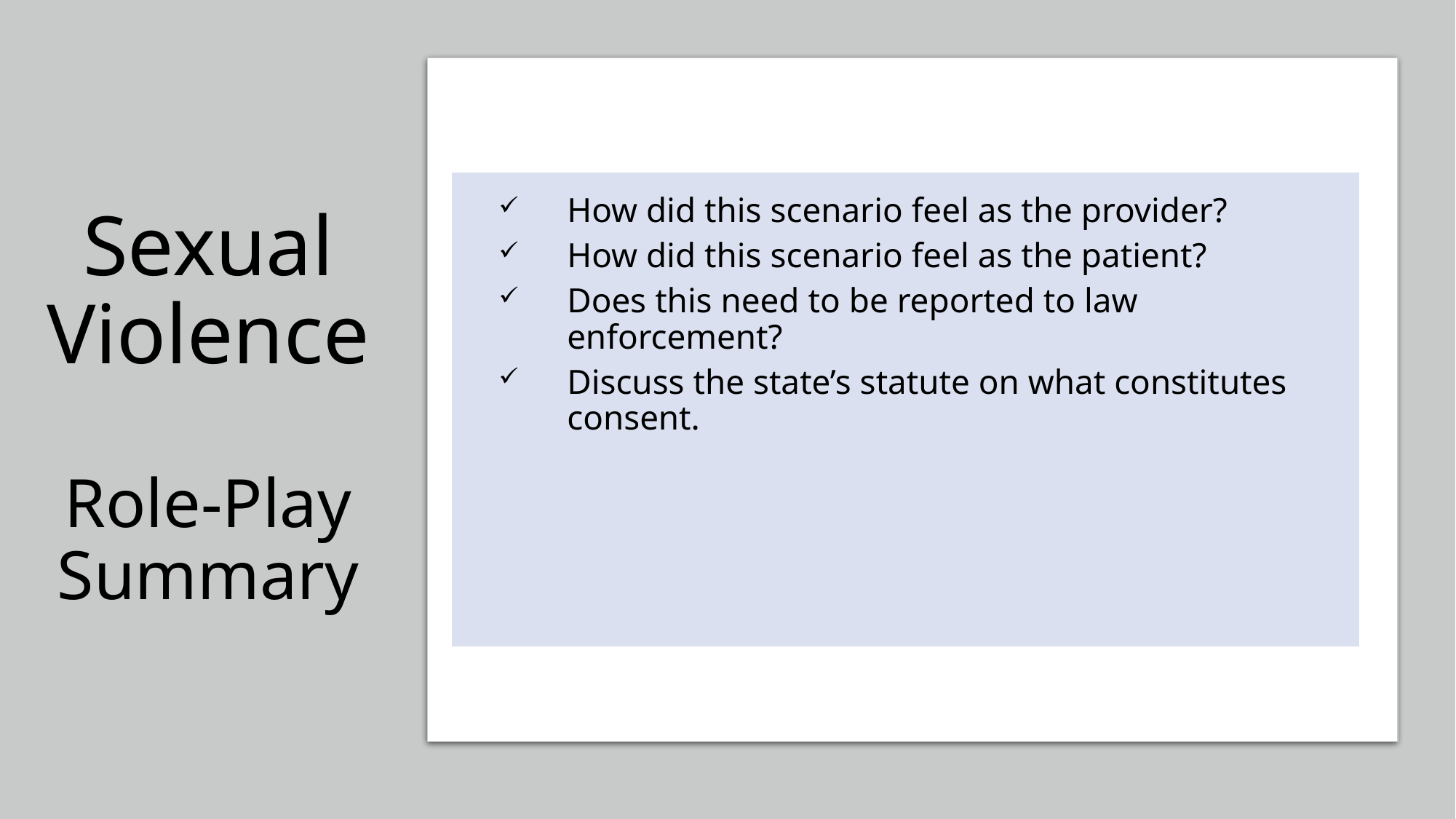

# Sexual Violence Role-Play Summary
How did this scenario feel as the provider?
How did this scenario feel as the patient?
Does this need to be reported to law enforcement?
Discuss the state’s statute on what constitutes consent.

## Slide 84
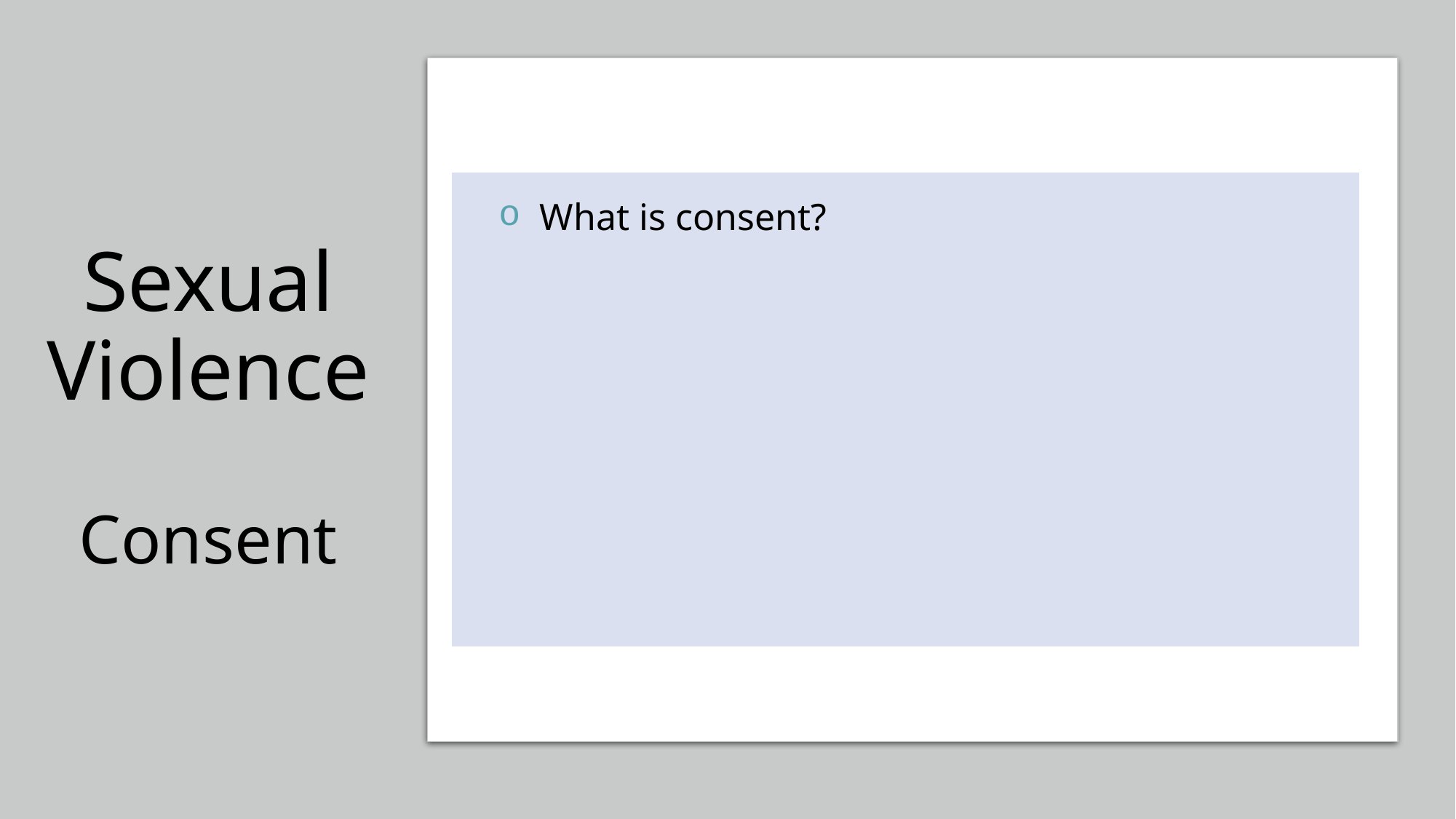

# Sexual Violence Consent
What is consent?

## Slide 85
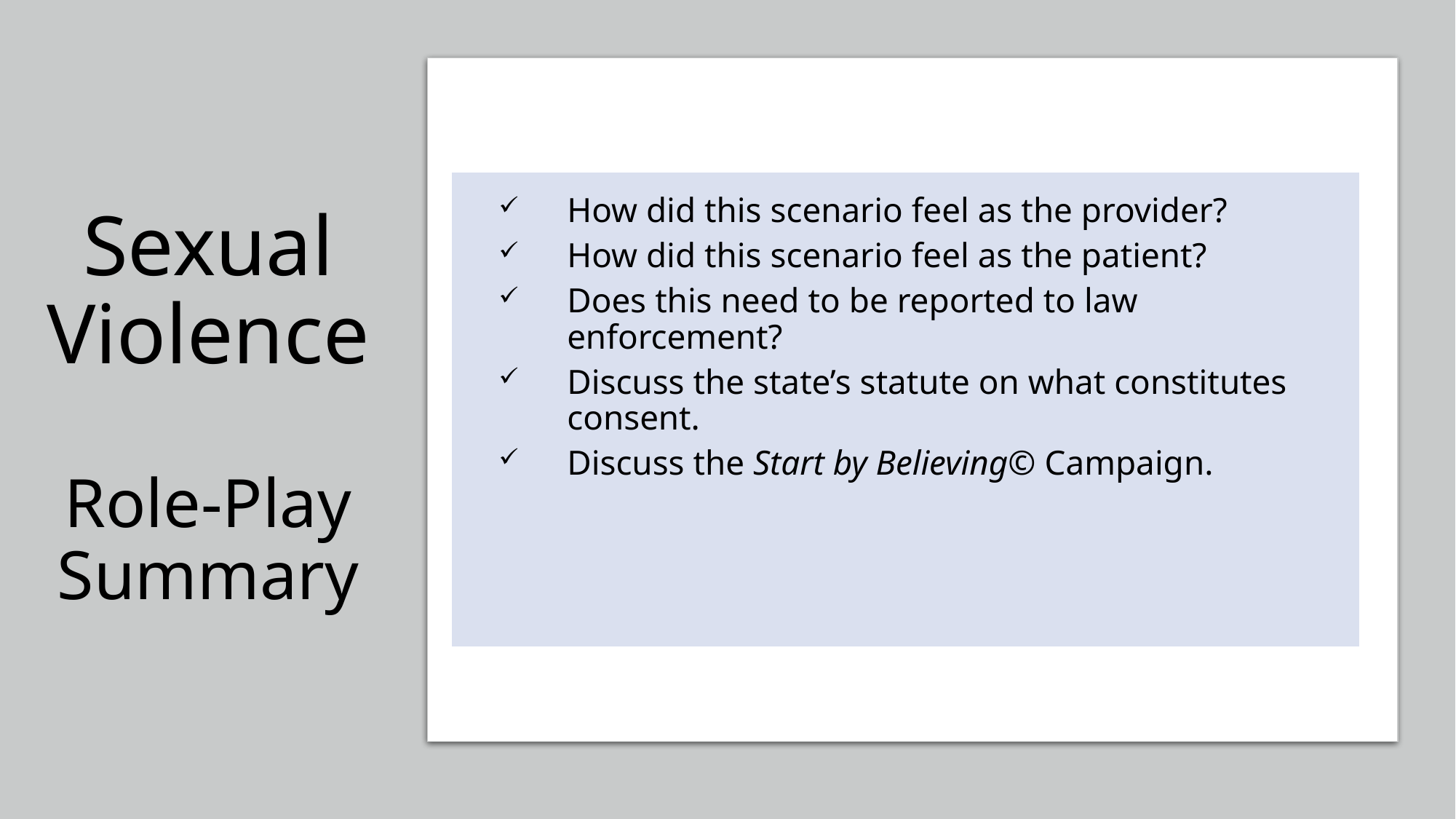

# Sexual Violence Role-Play Summary
How did this scenario feel as the provider?
How did this scenario feel as the patient?
Does this need to be reported to law enforcement?
Discuss the state’s statute on what constitutes consent.
Discuss the Start by Believing© Campaign.

## Slide 86
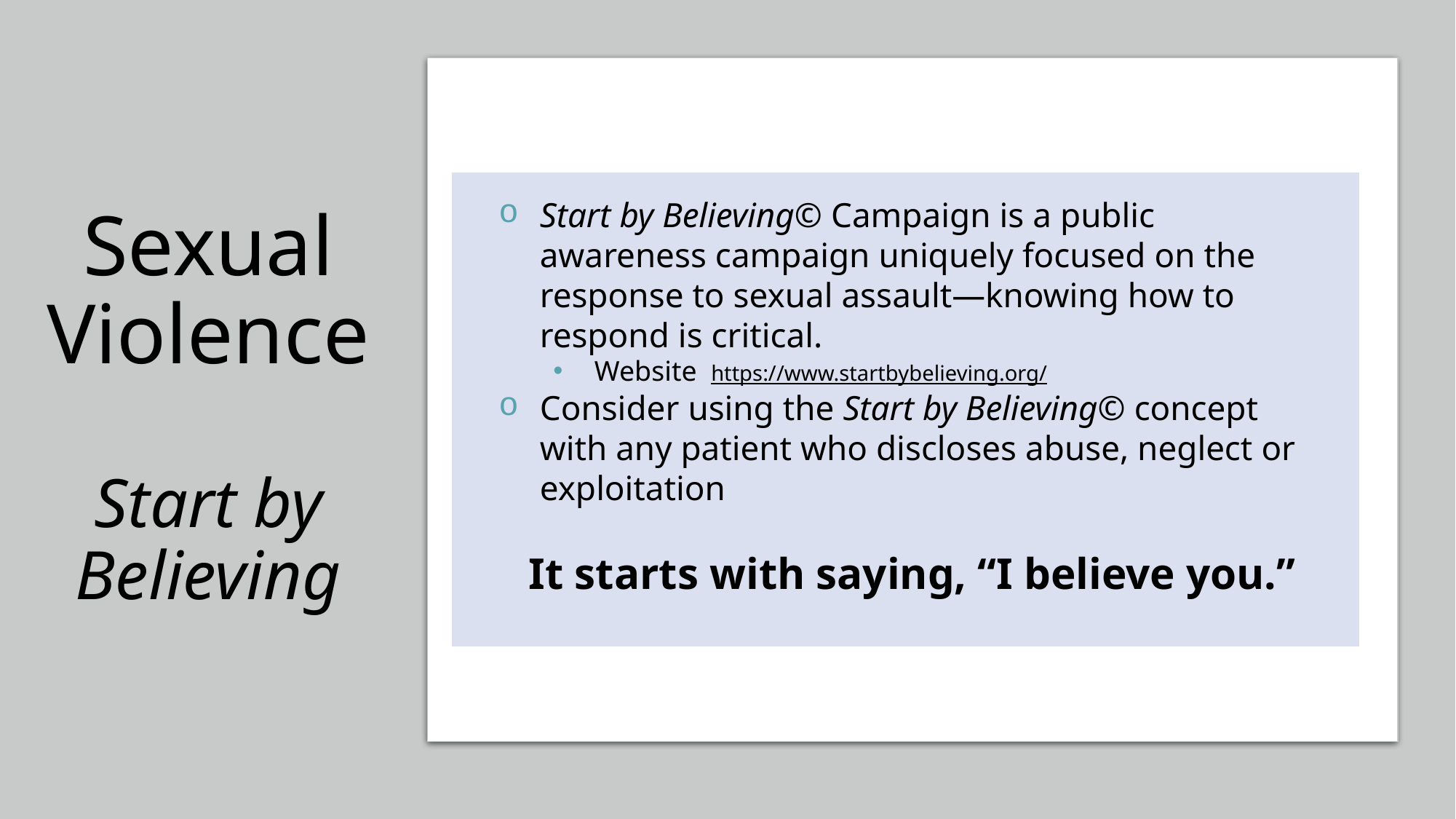

# Sexual Violence Start by Believing
Start by Believing© Campaign is a public awareness campaign uniquely focused on the response to sexual assault—knowing how to respond is critical.
Website https://www.startbybelieving.org/
Consider using the Start by Believing© concept with any patient who discloses abuse, neglect or exploitation
It starts with saying, “I believe you.”

## Slide 87
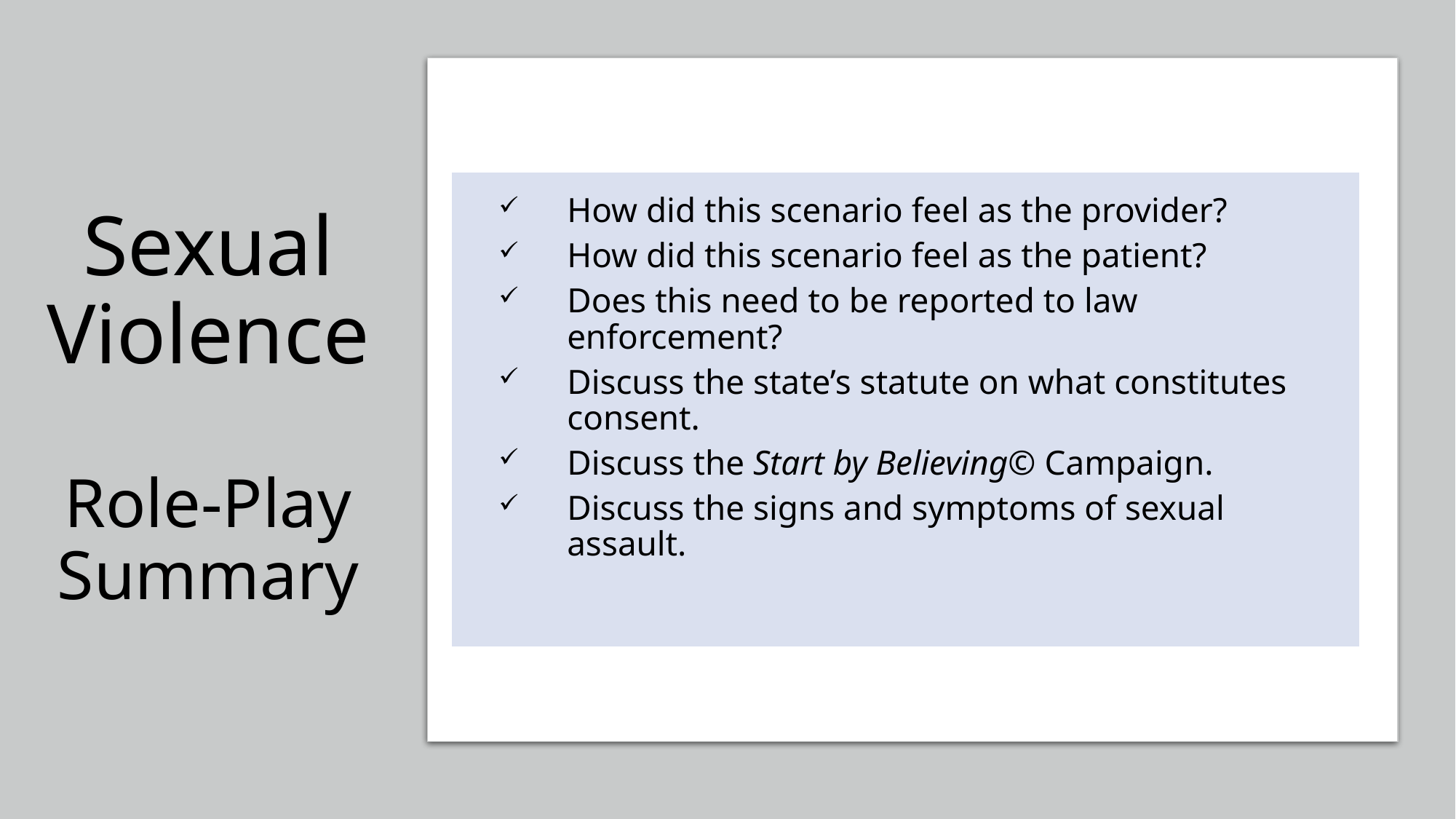

# Sexual Violence Role-Play Summary
How did this scenario feel as the provider?
How did this scenario feel as the patient?
Does this need to be reported to law enforcement?
Discuss the state’s statute on what constitutes consent.
Discuss the Start by Believing© Campaign.
Discuss the signs and symptoms of sexual assault.

## Slide 88
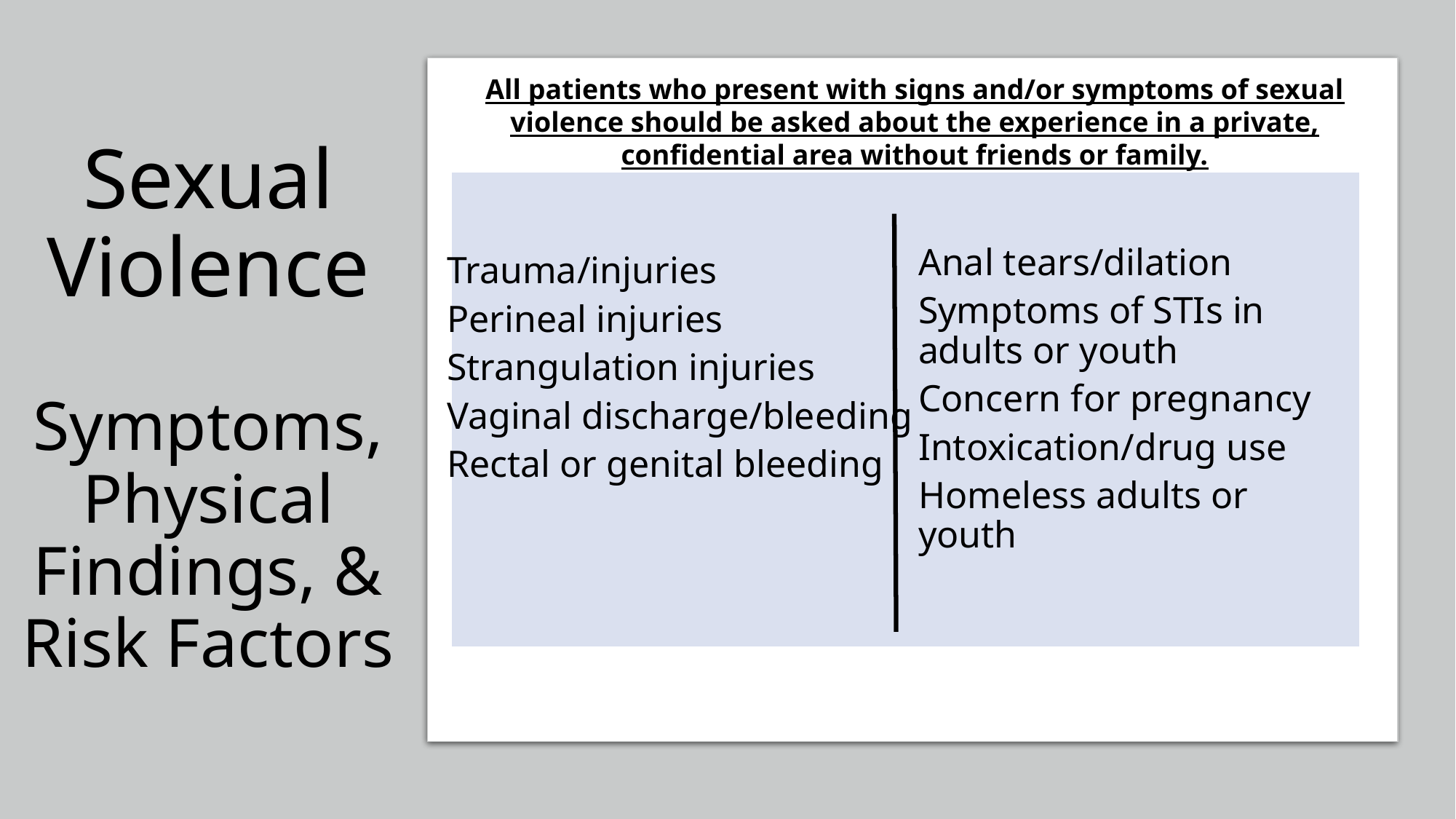

# Sexual Violence Symptoms, Physical Findings, & Risk Factors
All patients who present with signs and/or symptoms of sexual violence should be asked about the experience in a private, confidential area without friends or family.
Anal tears/dilation
Symptoms of STIs in adults or youth
Concern for pregnancy
Intoxication/drug use
Homeless adults or youth
Trauma/injuries
Perineal injuries
Strangulation injuries
Vaginal discharge/bleeding
Rectal or genital bleeding

## Slide 89
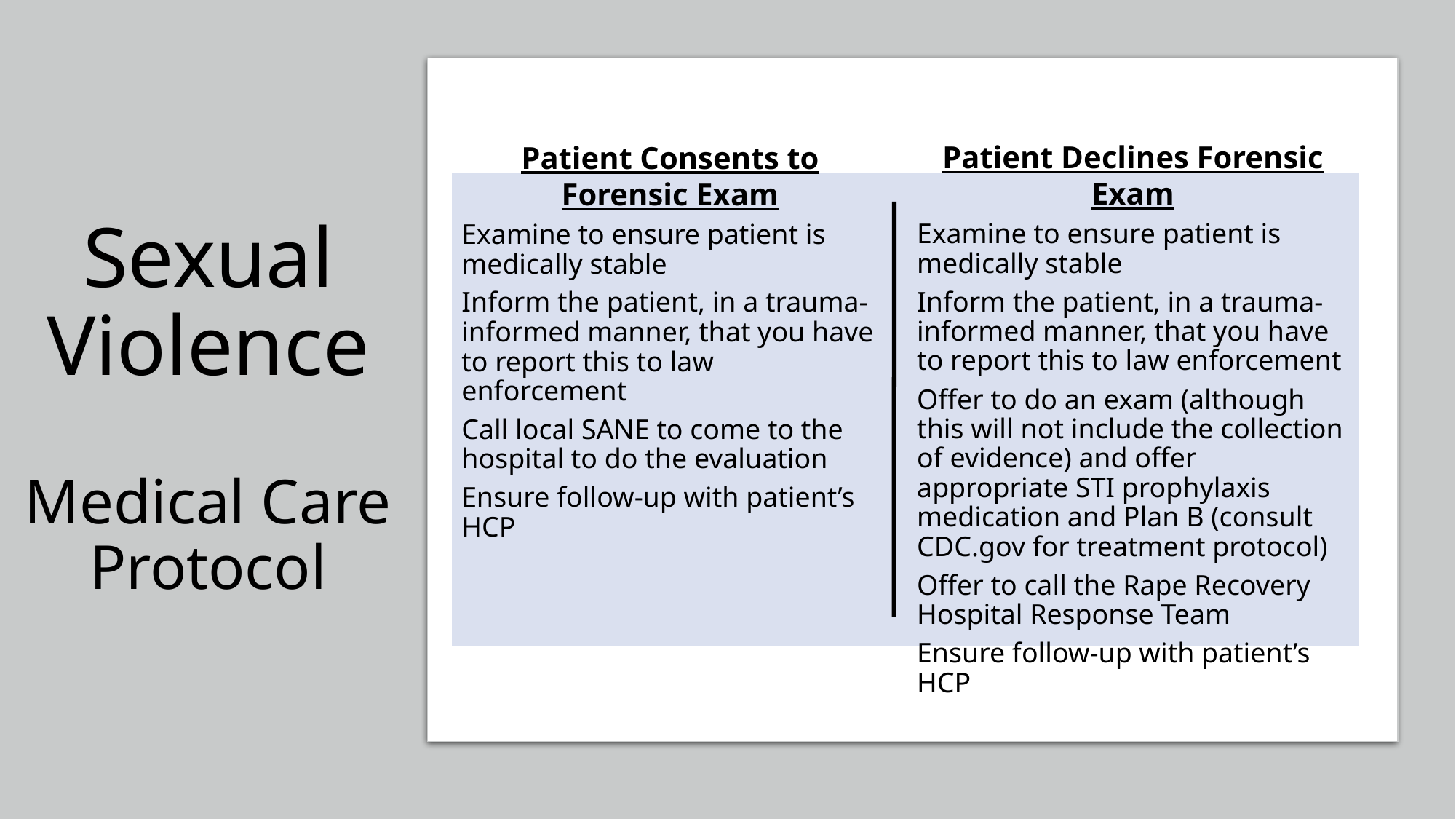

# Sexual Violence Medical Care Protocol
Patient Declines Forensic Exam
Examine to ensure patient is medically stable
Inform the patient, in a trauma-informed manner, that you have to report this to law enforcement
Offer to do an exam (although this will not include the collection of evidence) and offer appropriate STI prophylaxis medication and Plan B (consult CDC.gov for treatment protocol)
Offer to call the Rape Recovery Hospital Response Team
Ensure follow-up with patient’s HCP
Patient Consents to Forensic Exam
Examine to ensure patient is medically stable
Inform the patient, in a trauma-informed manner, that you have to report this to law enforcement
Call local SANE to come to the hospital to do the evaluation
Ensure follow-up with patient’s HCP

## Slide 90
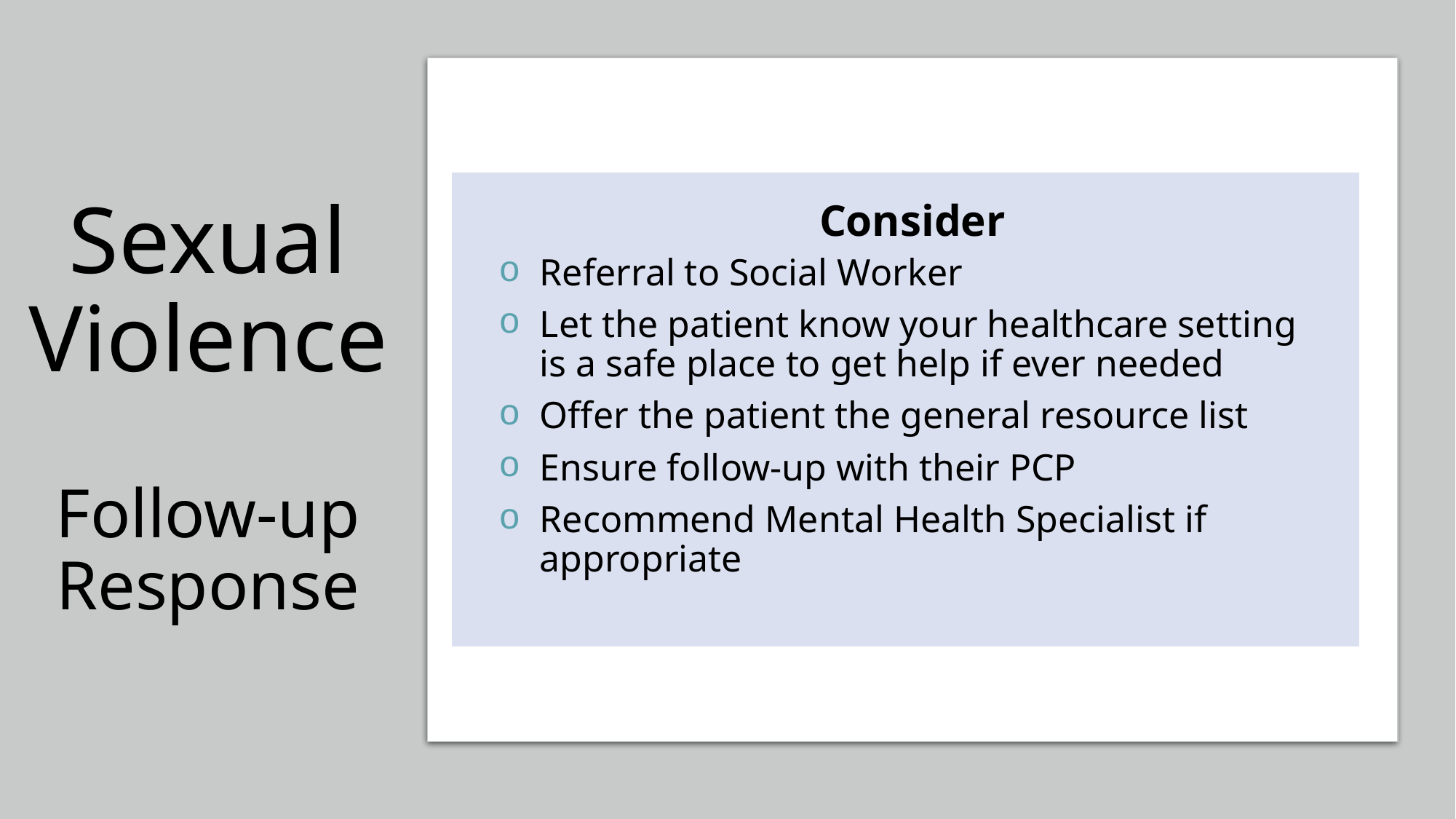

# Sexual Violence Follow-up Response
Consider
Referral to Social Worker
Let the patient know your healthcare setting is a safe place to get help if ever needed
Offer the patient the general resource list
Ensure follow-up with their PCP
Recommend Mental Health Specialist if appropriate

## Slide 91
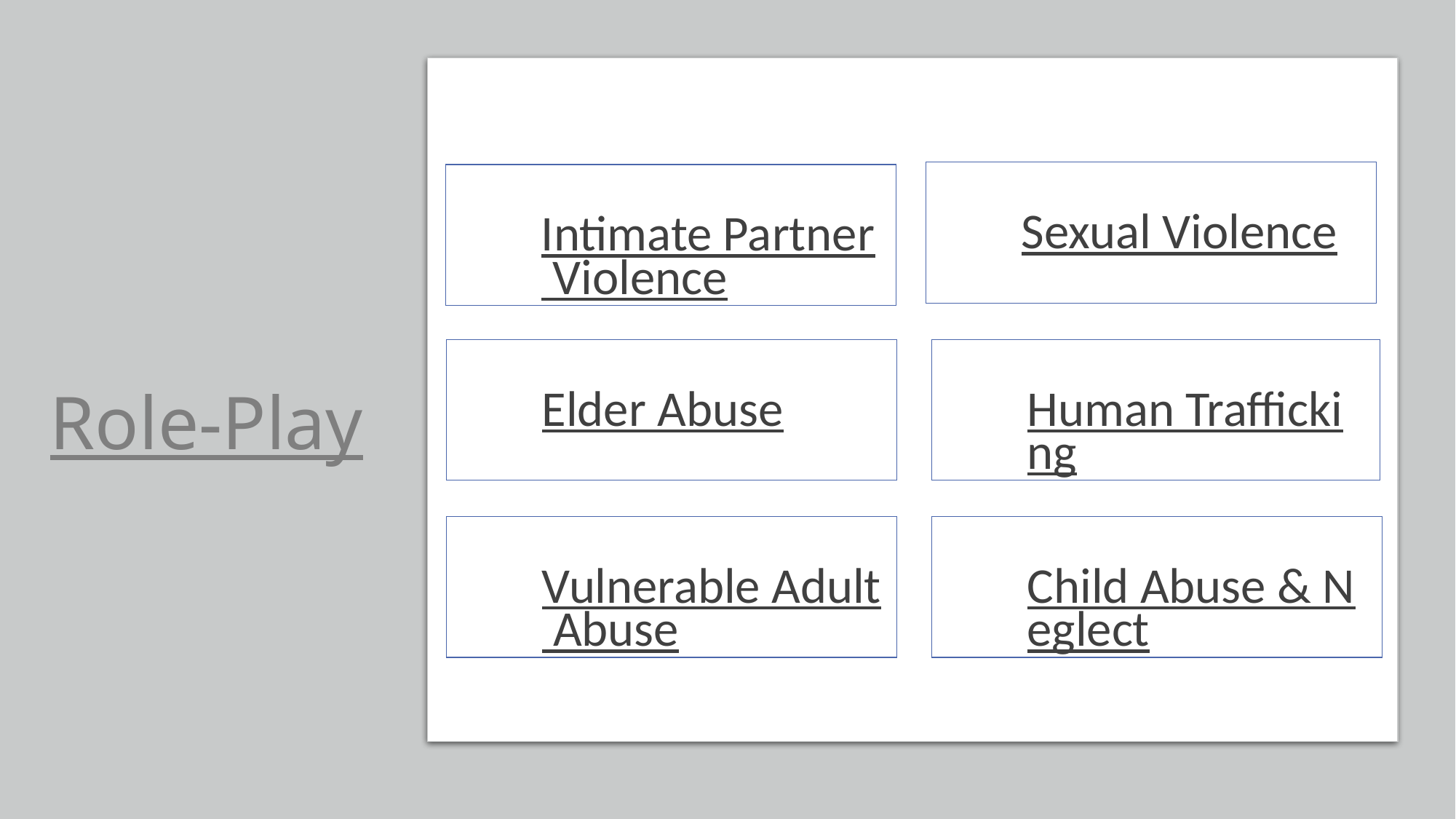

Sexual Violence
Intimate Partner Violence
# Role-Play
Elder Abuse
Human Trafficking
Vulnerable Adult Abuse
Child Abuse & Neglect

## Slide 92
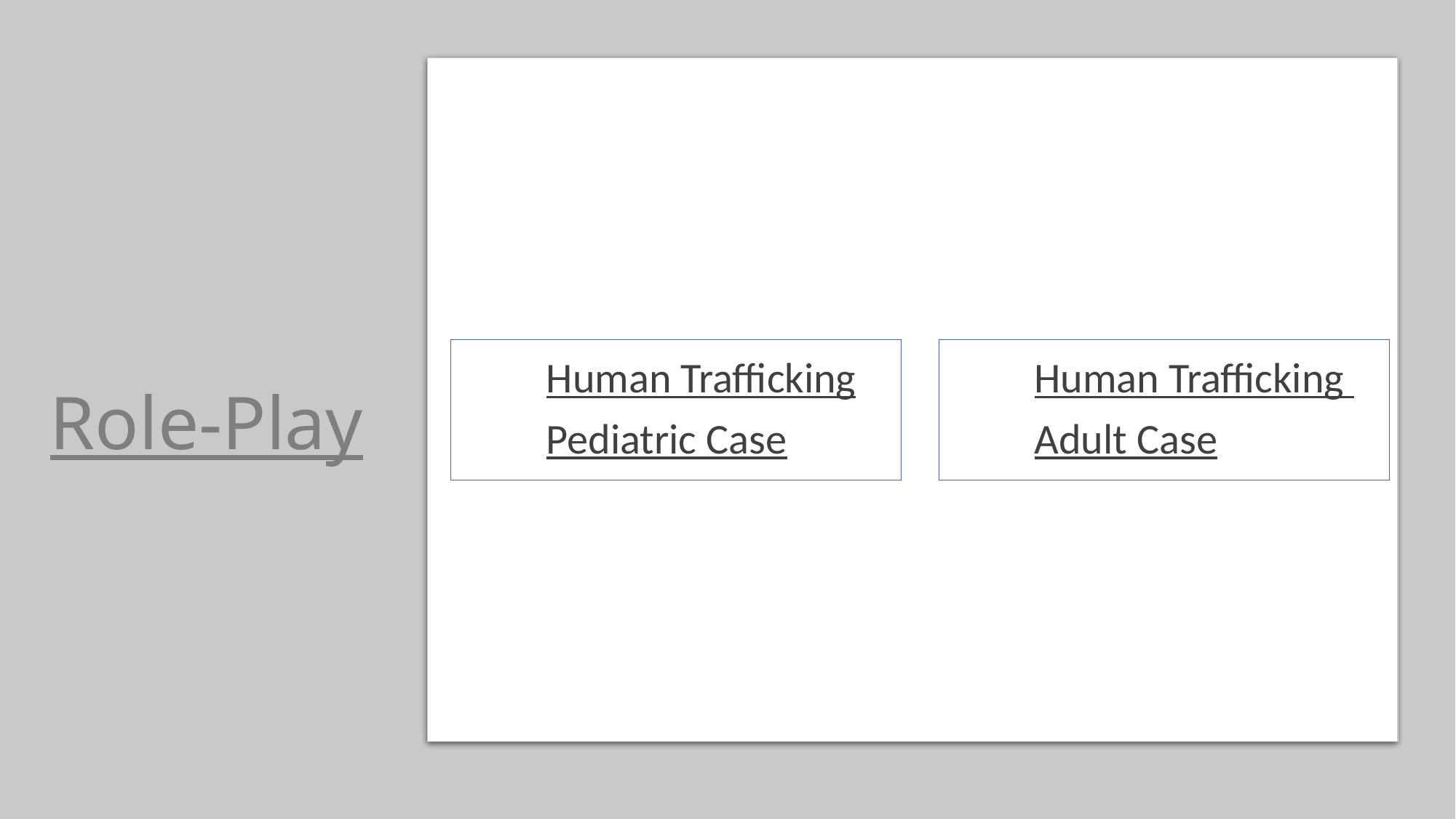

Role-Play
Human Trafficking
Pediatric Case
Human Trafficking
Adult Case

## Slide 93
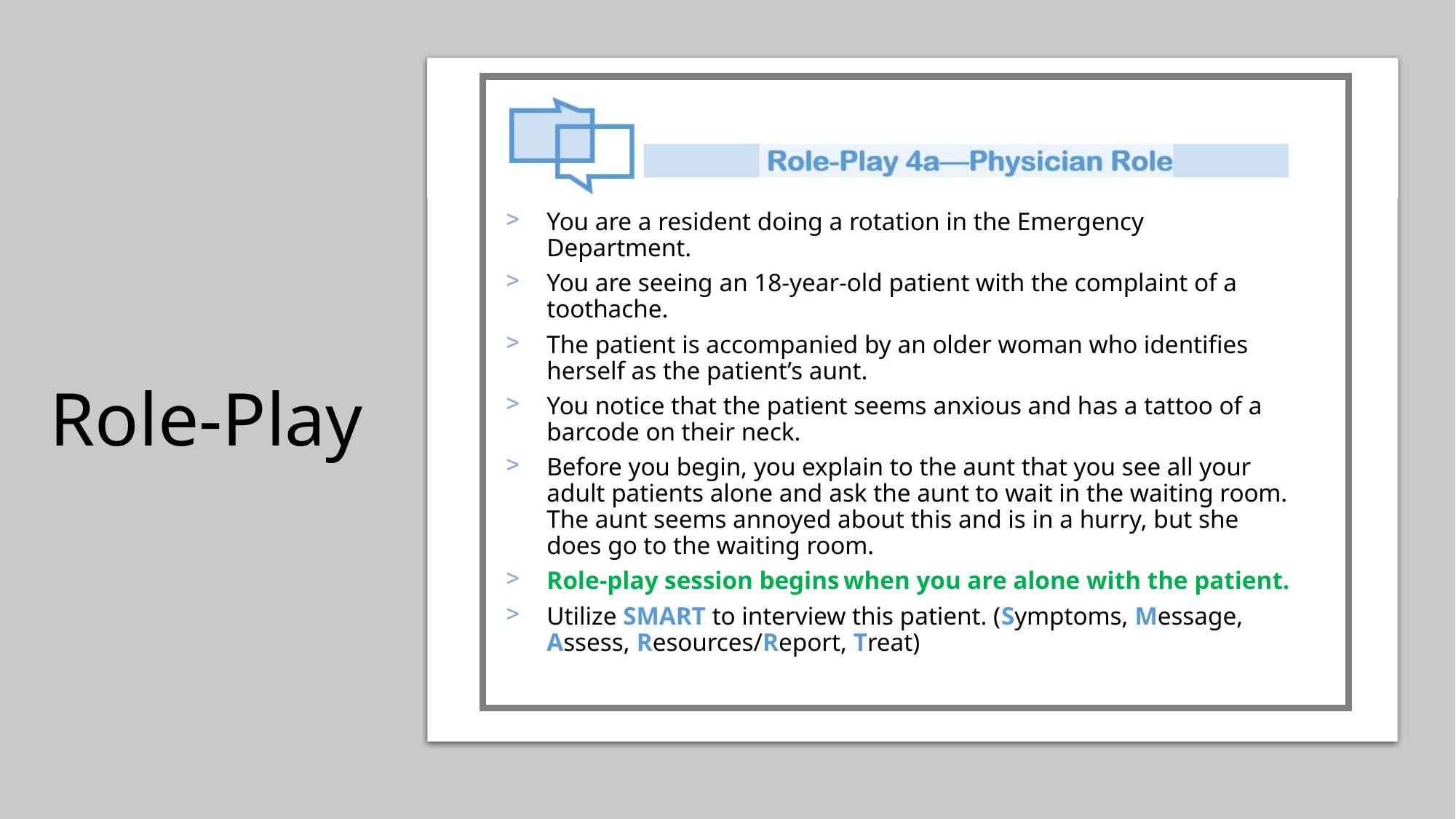

You are a resident doing a rotation in the Emergency Department.
You are seeing an 18-year-old patient with the complaint of a toothache.
The patient is accompanied by an older woman who identifies herself as the patient’s aunt.
You notice that the patient seems anxious and has a tattoo of a barcode on their neck.
Before you begin, you explain to the aunt that you see all your adult patients alone and ask the aunt to wait in the waiting room. The aunt seems annoyed about this and is in a hurry, but she does go to the waiting room.
Role-play session begins when you are alone with the patient.
Utilize SMART to interview this patient. (Symptoms, Message, Assess, Resources/Report, Treat)
# Role-Play

## Slide 94
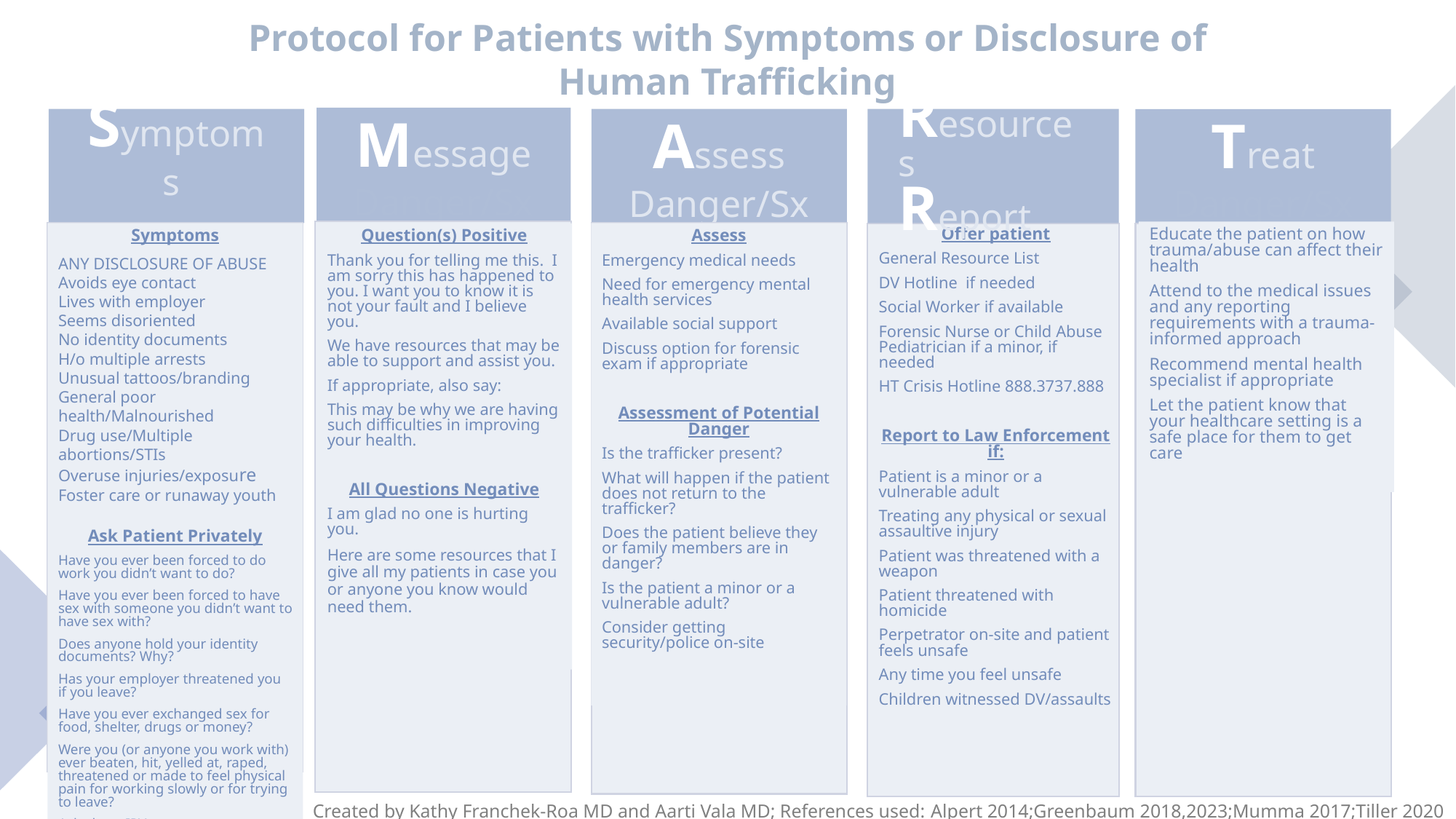

Protocol for Patients with Symptoms or Disclosure of
Human Trafficking
Message
Danger/Sx
Symptoms
Assess
Danger/Sx
Resources
Report
Treat
Danger/Sx
Educate the patient on how trauma/abuse can affect their health
Attend to the medical issues and any reporting requirements with a trauma-informed approach
Recommend mental health specialist if appropriate
Let the patient know that your healthcare setting is a safe place for them to get care
Question(s) Positive
Thank you for telling me this. I am sorry this has happened to you. I want you to know it is not your fault and I believe you.
We have resources that may be able to support and assist you.
If appropriate, also say:
This may be why we are having such difficulties in improving your health.
All Questions Negative
I am glad no one is hurting you.
Here are some resources that I give all my patients in case you or anyone you know would need them.
Offer patient
General Resource List
DV Hotline if needed
Social Worker if available
Forensic Nurse or Child Abuse Pediatrician if a minor, if needed
HT Crisis Hotline 888.3737.888
Report to Law Enforcement if:
Patient is a minor or a vulnerable adult
Treating any physical or sexual assaultive injury
Patient was threatened with a weapon
Patient threatened with homicide
Perpetrator on-site and patient feels unsafe
Any time you feel unsafe
Children witnessed DV/assaults
Assess
Emergency medical needs
Need for emergency mental health services
Available social support
Discuss option for forensic exam if appropriate
Assessment of Potential Danger
Is the trafficker present?
What will happen if the patient does not return to the trafficker?
Does the patient believe they or family members are in danger?
Is the patient a minor or a vulnerable adult?
Consider getting security/police on-site
Symptoms
ANY DISCLOSURE OF ABUSE
Avoids eye contact
Lives with employer
Seems disoriented
No identity documents
H/o multiple arrests
Unusual tattoos/branding
General poor health/Malnourished
Drug use/Multiple abortions/STIs
Overuse injuries/exposure
Foster care or runaway youth
Ask Patient Privately
Have you ever been forced to do work you didn’t want to do?
Have you ever been forced to have sex with someone you didn’t want to have sex with?
Does anyone hold your identity documents? Why?
Has your employer threatened you if you leave?
Have you ever exchanged sex for food, shelter, drugs or money?
Were you (or anyone you work with) ever beaten, hit, yelled at, raped, threatened or made to feel physical pain for working slowly or for trying to leave?
Ask about IPV
Created by Kathy Franchek-Roa MD and Aarti Vala MD; References used: Alpert 2014;Greenbaum 2018,2023;Mumma 2017;Tiller 2020

## Slide 95
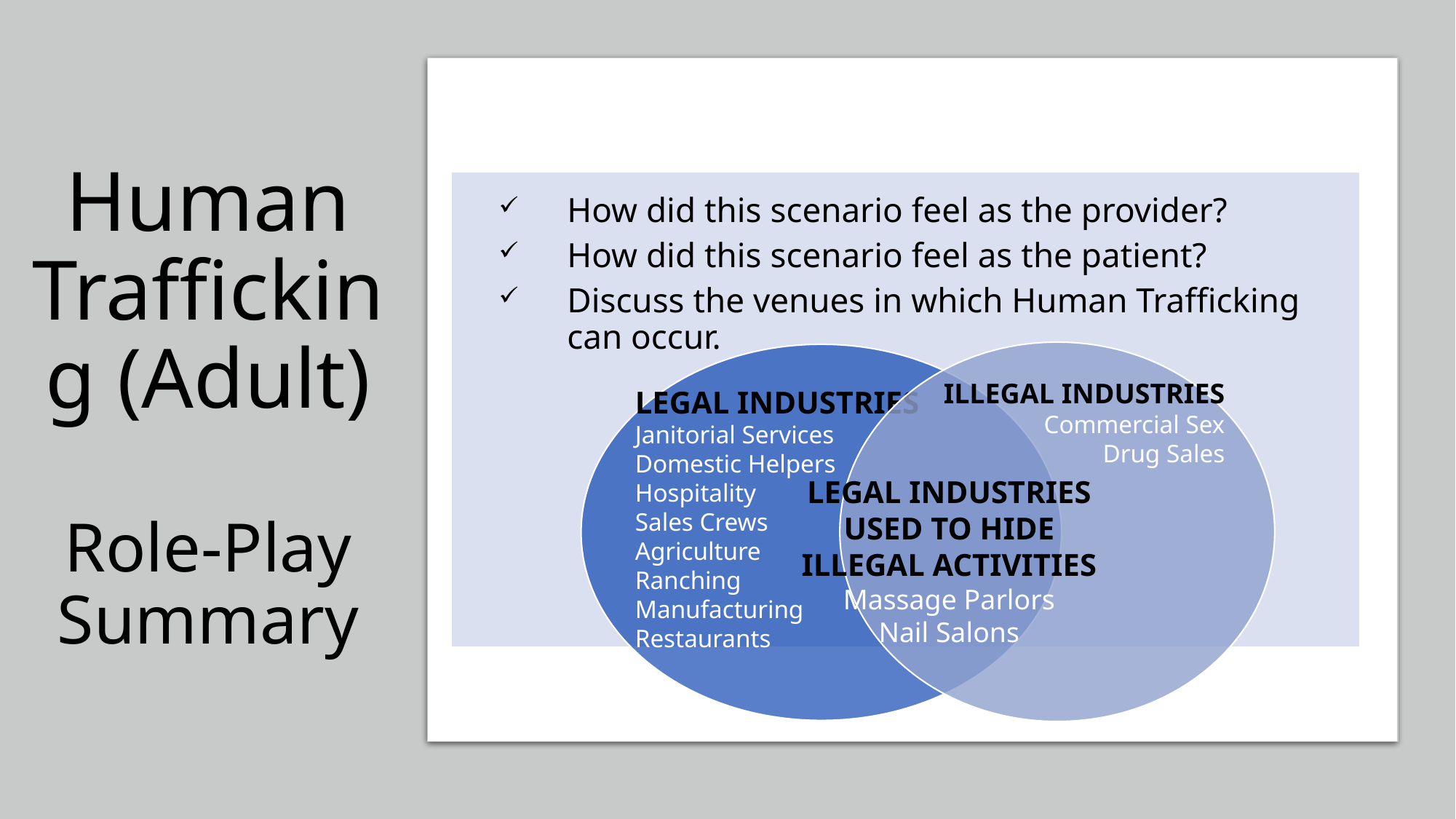

# Human Trafficking (Adult) Role-Play Summary
How did this scenario feel as the provider?
How did this scenario feel as the patient?
Discuss the venues in which Human Trafficking can occur.
Illegal Industries
Commercial Sex
Drug Sales
Legal Industries
Janitorial Services
Domestic Helpers
Hospitality
Sales Crews
Agriculture
Ranching
Manufacturing
Restaurants
Legal Industries
Used to Hide
Illegal Activities
Massage Parlors
Nail Salons

## Slide 96
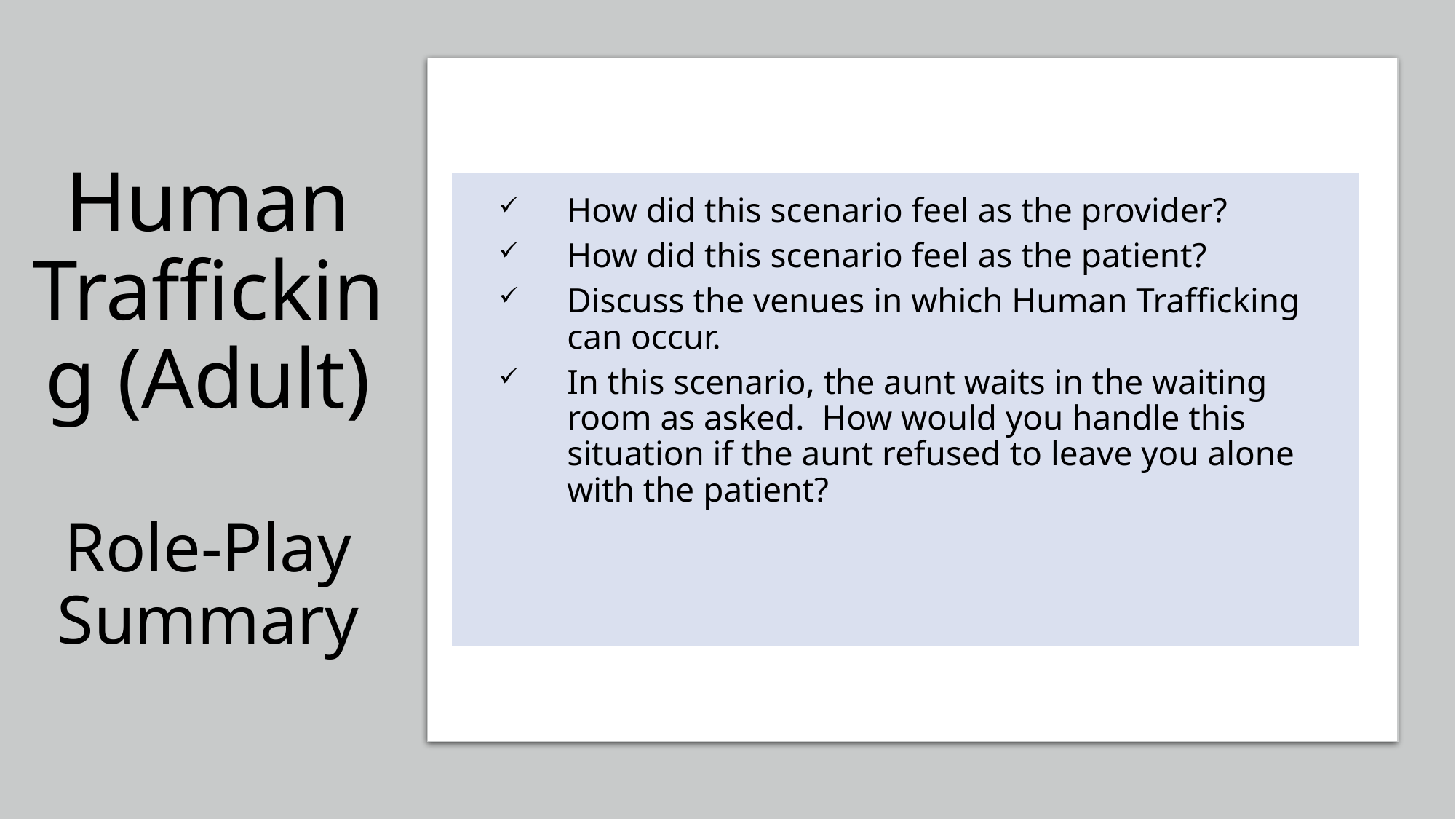

# Human Trafficking (Adult) Role-Play Summary
How did this scenario feel as the provider?
How did this scenario feel as the patient?
Discuss the venues in which Human Trafficking can occur.
In this scenario, the aunt waits in the waiting room as asked. How would you handle this situation if the aunt refused to leave you alone with the patient?

## Slide 97
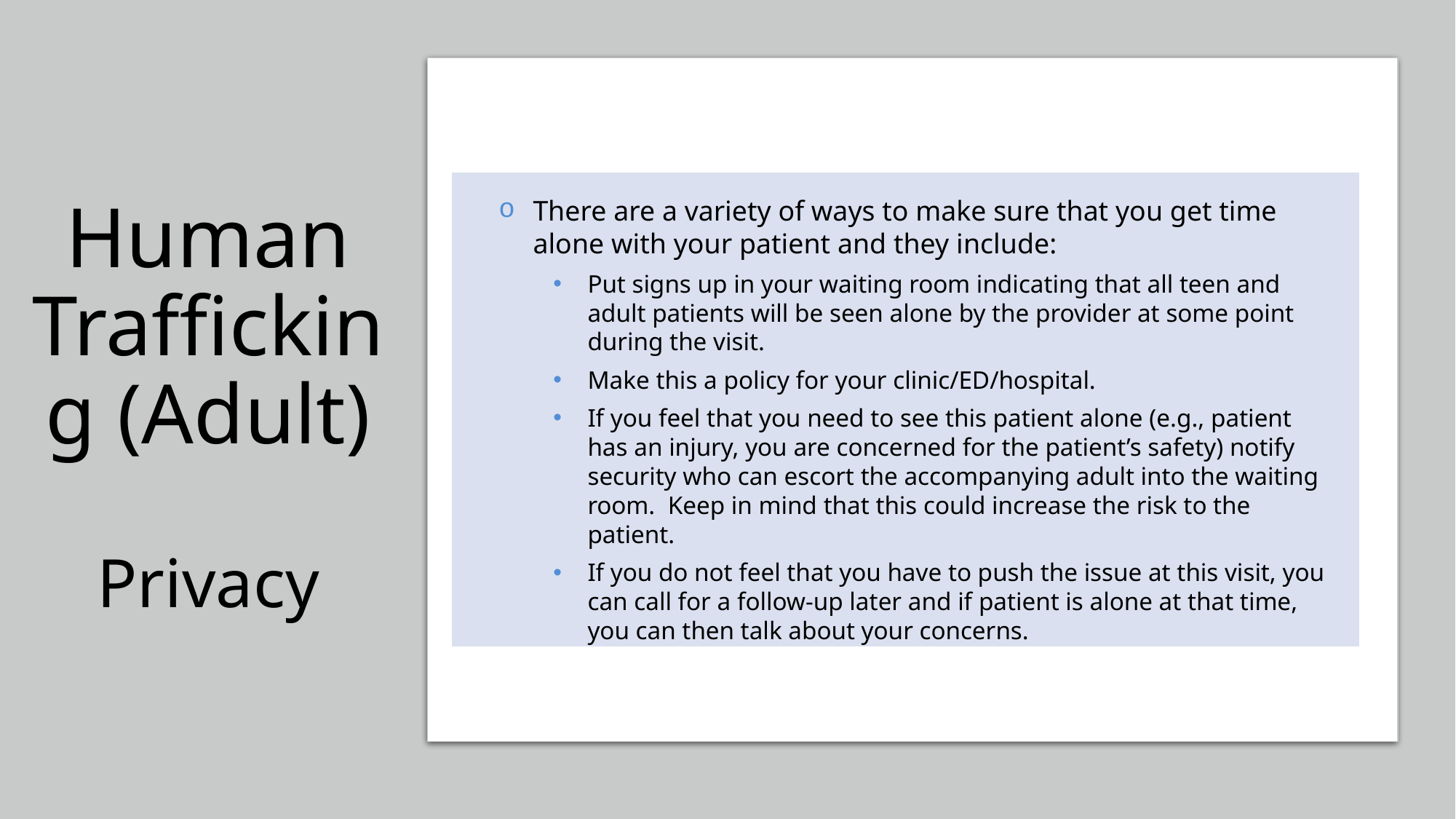

# Human Trafficking (Adult) Privacy
There are a variety of ways to make sure that you get time alone with your patient and they include:
Put signs up in your waiting room indicating that all teen and adult patients will be seen alone by the provider at some point during the visit.
Make this a policy for your clinic/ED/hospital.
If you feel that you need to see this patient alone (e.g., patient has an injury, you are concerned for the patient’s safety) notify security who can escort the accompanying adult into the waiting room. Keep in mind that this could increase the risk to the patient.
If you do not feel that you have to push the issue at this visit, you can call for a follow-up later and if patient is alone at that time, you can then talk about your concerns.

## Slide 98
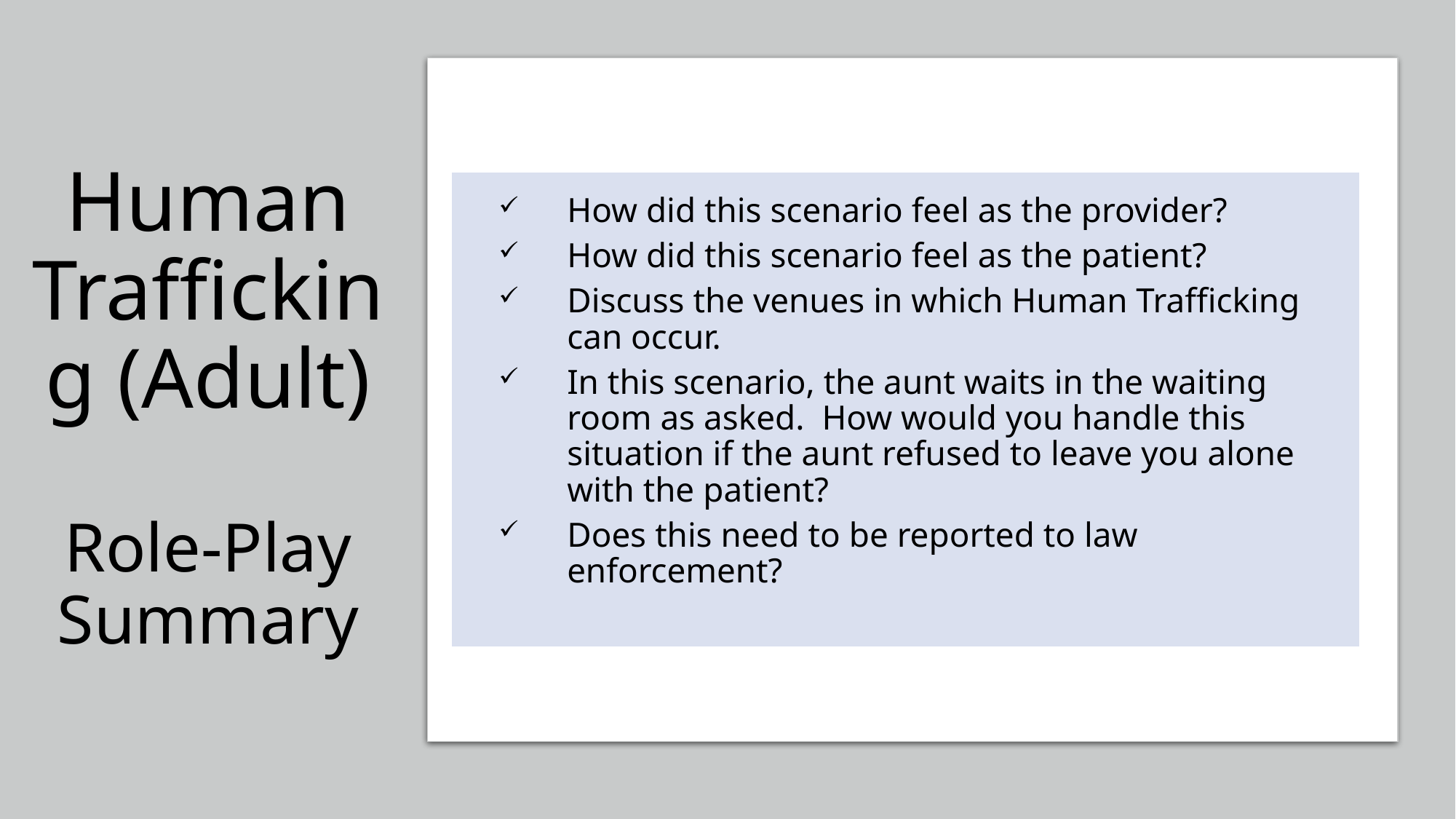

# Human Trafficking (Adult) Role-Play Summary
How did this scenario feel as the provider?
How did this scenario feel as the patient?
Discuss the venues in which Human Trafficking can occur.
In this scenario, the aunt waits in the waiting room as asked. How would you handle this situation if the aunt refused to leave you alone with the patient?
Does this need to be reported to law enforcement?

## Slide 99
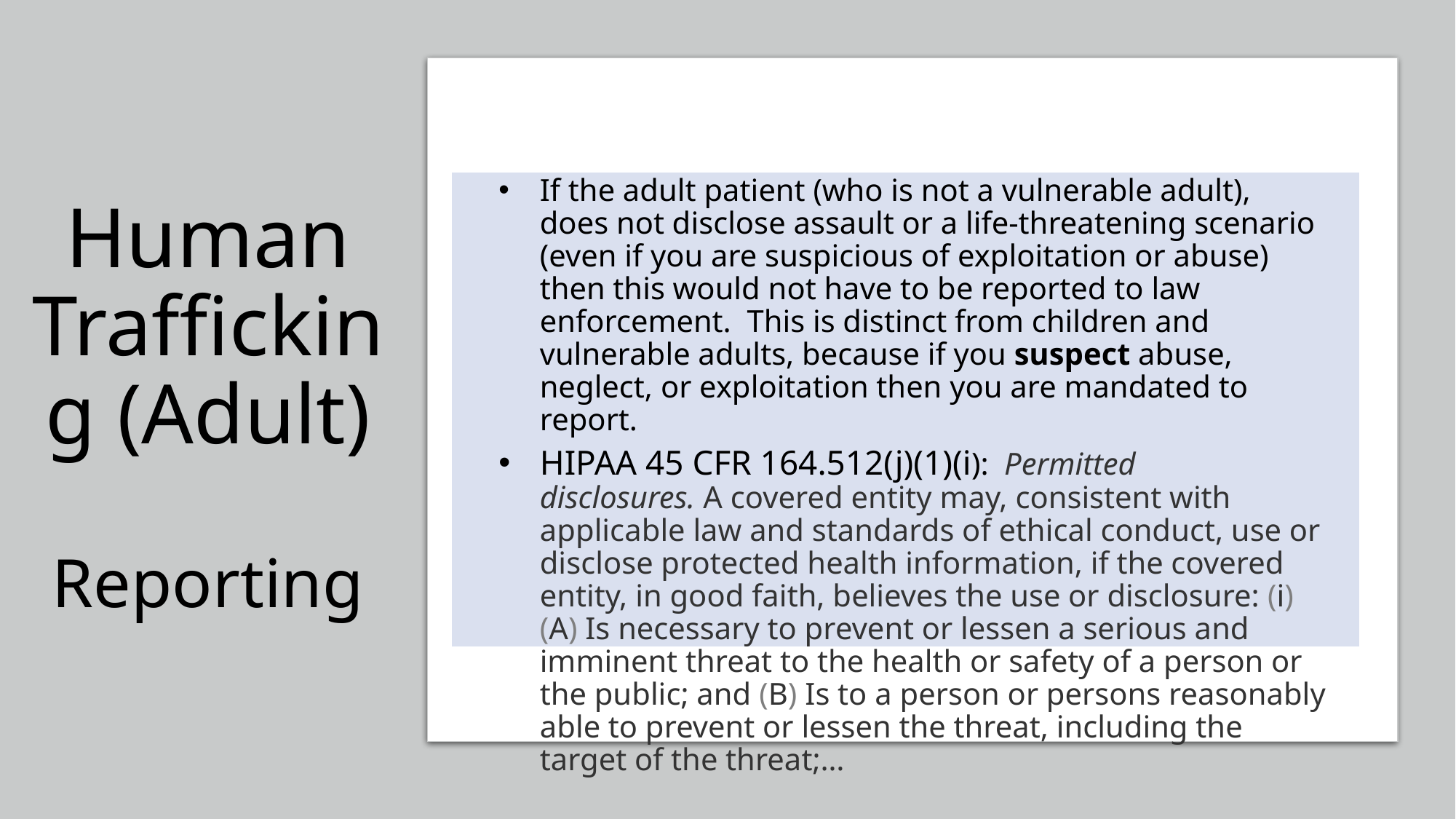

# Human Trafficking (Adult) Reporting
If the adult patient (who is not a vulnerable adult), does not disclose assault or a life-threatening scenario (even if you are suspicious of exploitation or abuse) then this would not have to be reported to law enforcement. This is distinct from children and vulnerable adults, because if you suspect abuse, neglect, or exploitation then you are mandated to report.
HIPAA 45 CFR 164.512(j)(1)(i): Permitted disclosures. A covered entity may, consistent with applicable law and standards of ethical conduct, use or disclose protected health information, if the covered entity, in good faith, believes the use or disclosure: (i) (A) Is necessary to prevent or lessen a serious and imminent threat to the health or safety of a person or the public; and (B) Is to a person or persons reasonably able to prevent or lessen the threat, including the target of the threat;…

## Slide 100
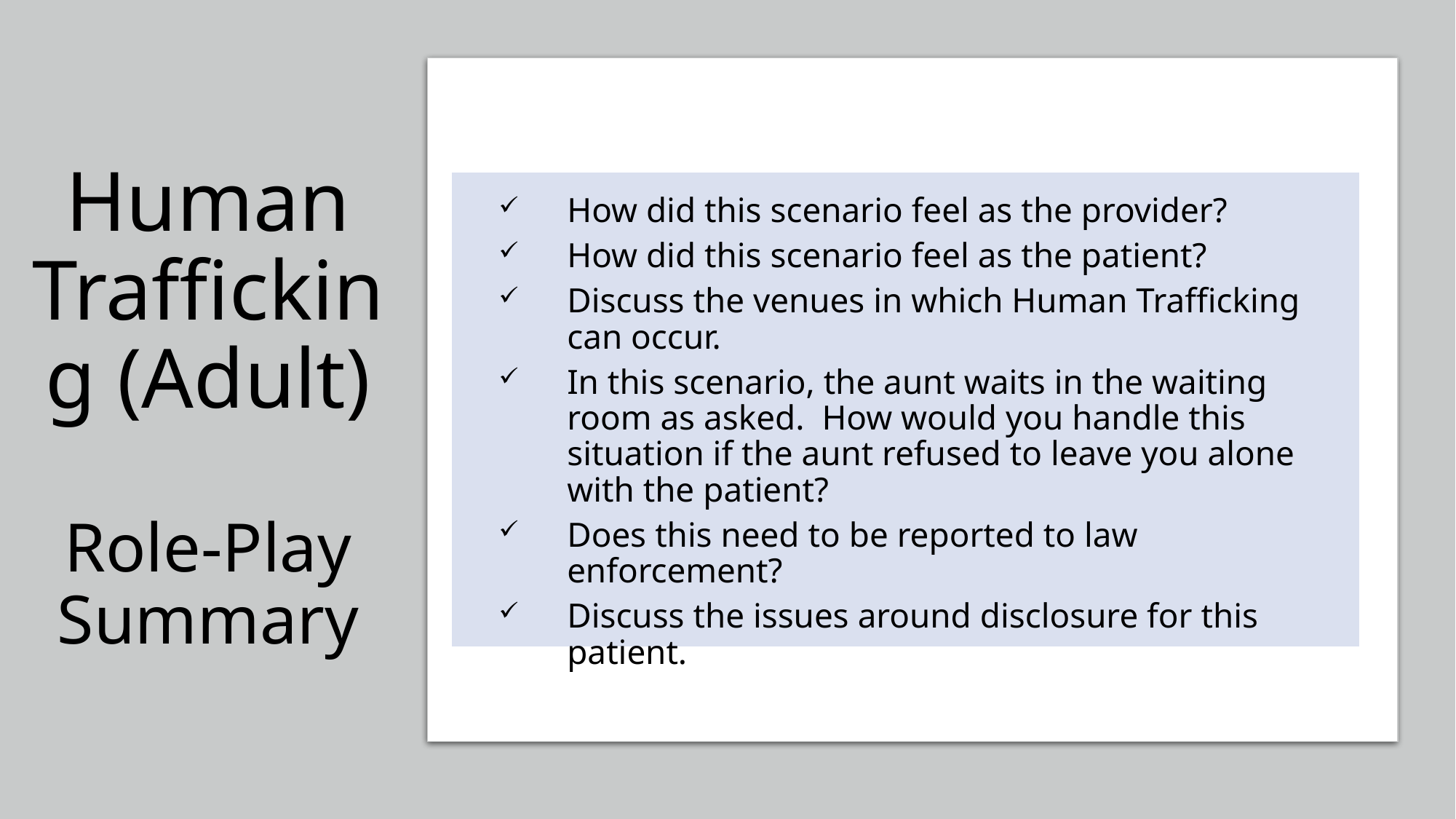

# Human Trafficking (Adult) Role-Play Summary
How did this scenario feel as the provider?
How did this scenario feel as the patient?
Discuss the venues in which Human Trafficking can occur.
In this scenario, the aunt waits in the waiting room as asked. How would you handle this situation if the aunt refused to leave you alone with the patient?
Does this need to be reported to law enforcement?
Discuss the issues around disclosure for this patient.

## Slide 101
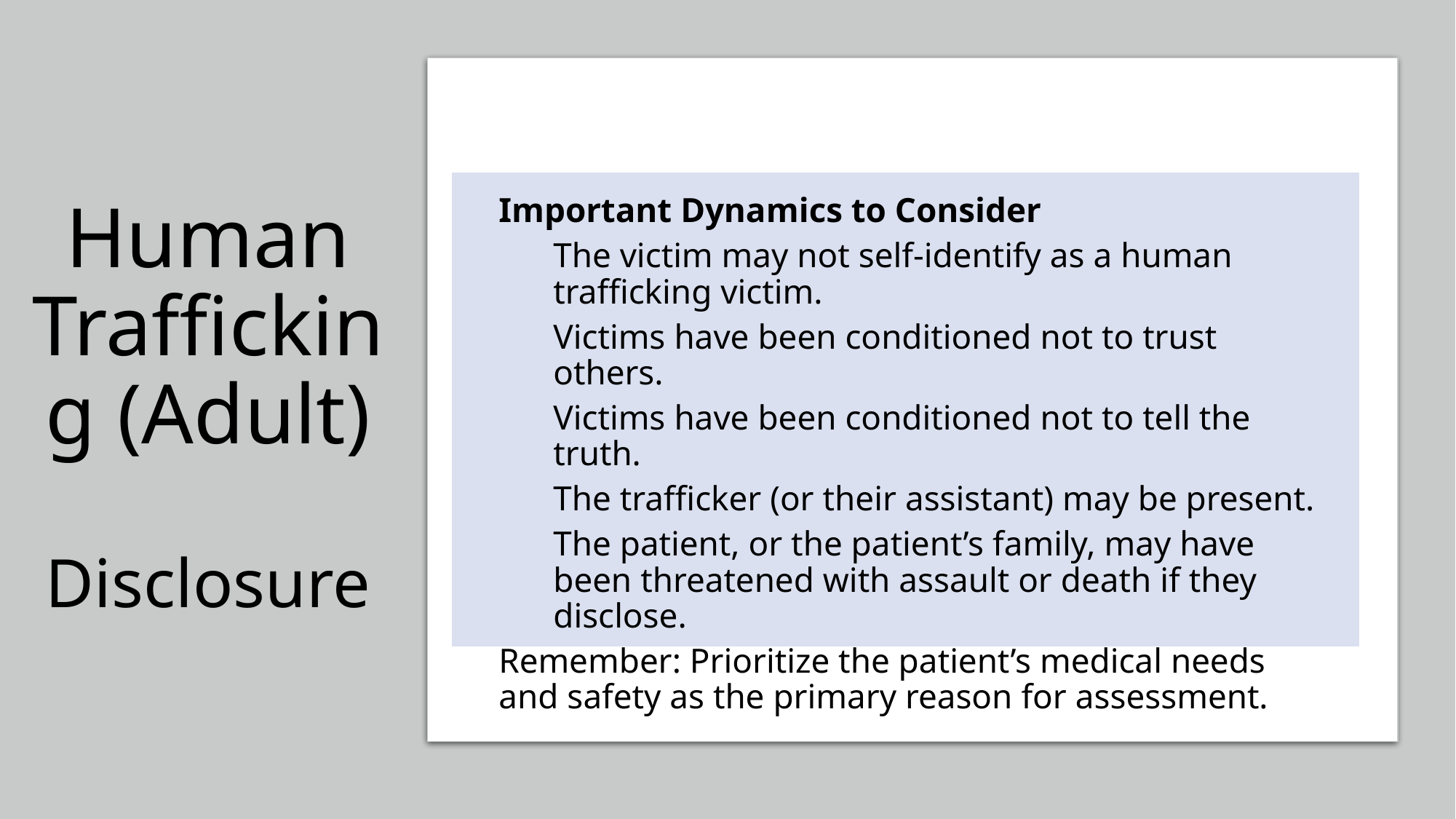

# Human Trafficking (Adult) Disclosure
Important Dynamics to Consider
The victim may not self-identify as a human trafficking victim.
Victims have been conditioned not to trust others.
Victims have been conditioned not to tell the truth.
The trafficker (or their assistant) may be present.
The patient, or the patient’s family, may have been threatened with assault or death if they disclose.
Remember: Prioritize the patient’s medical needs and safety as the primary reason for assessment.

## Slide 102
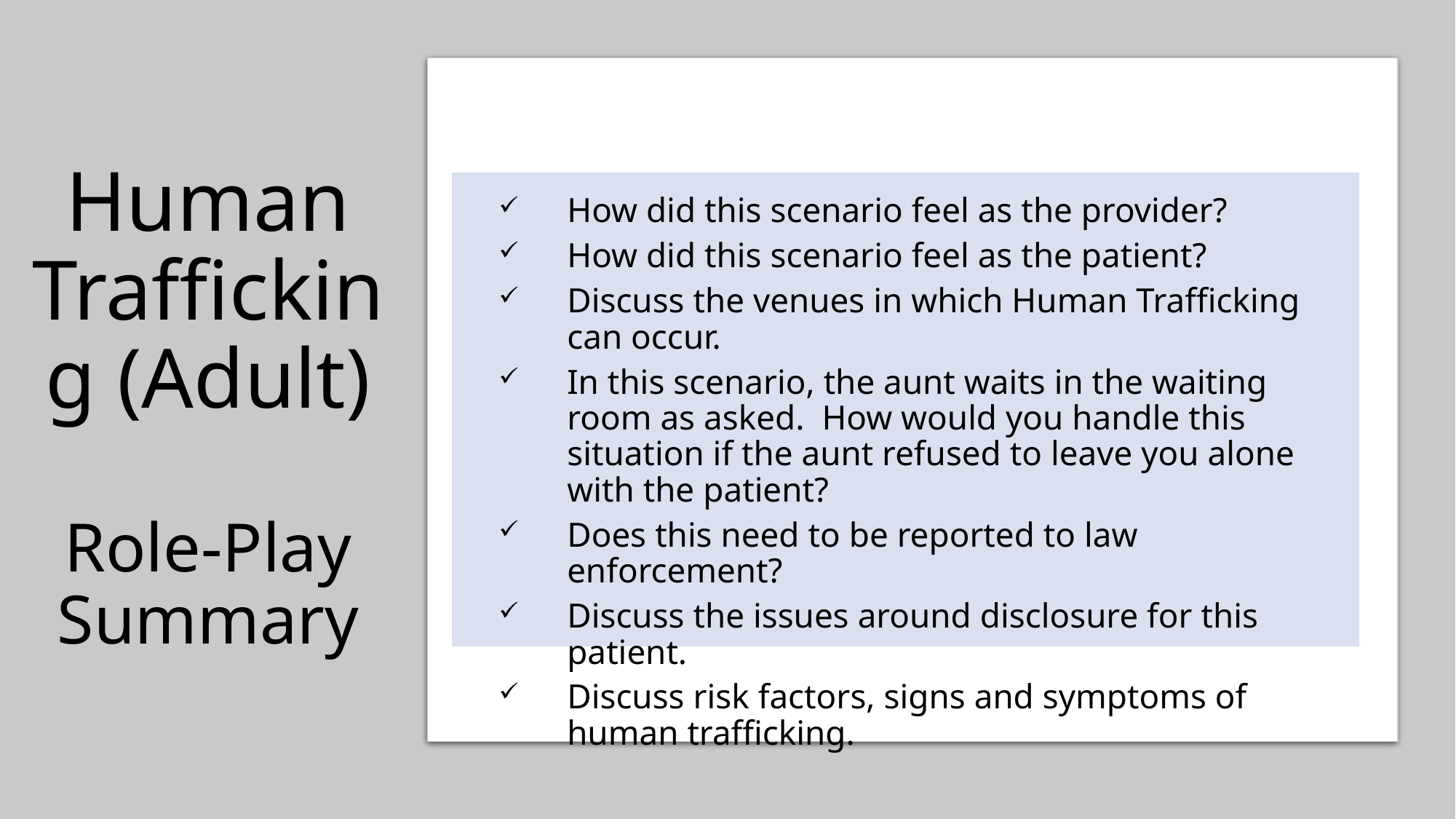

# Human Trafficking (Adult) Role-Play Summary
How did this scenario feel as the provider?
How did this scenario feel as the patient?
Discuss the venues in which Human Trafficking can occur.
In this scenario, the aunt waits in the waiting room as asked. How would you handle this situation if the aunt refused to leave you alone with the patient?
Does this need to be reported to law enforcement?
Discuss the issues around disclosure for this patient.
Discuss risk factors, signs and symptoms of human trafficking.

## Slide 103
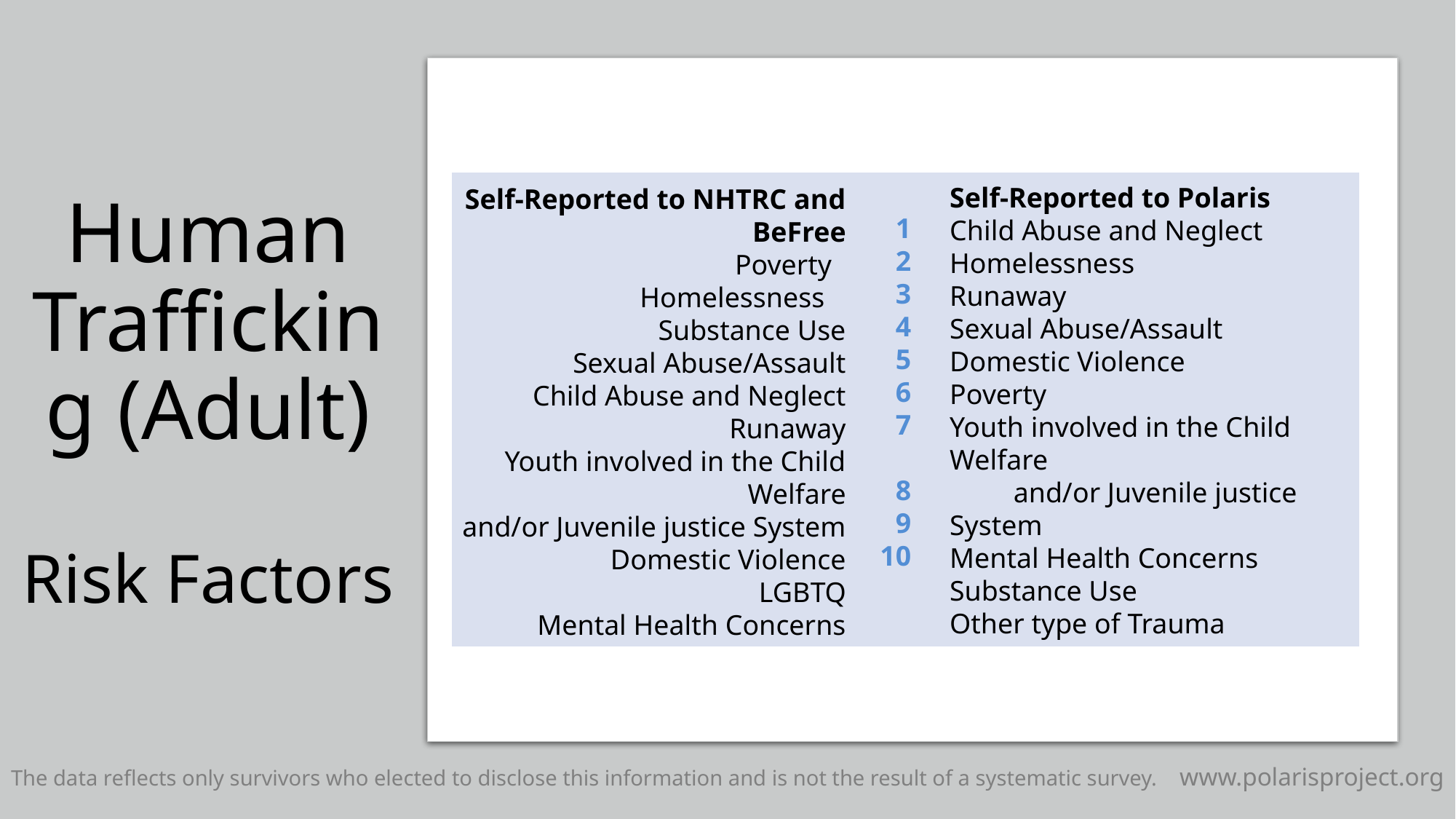

# Human Trafficking (Adult) Risk Factors
Self-Reported to Polaris
Child Abuse and Neglect
Homelessness
Runaway
Sexual Abuse/Assault
Domestic Violence
Poverty
Youth involved in the Child Welfare
 and/or Juvenile justice System
Mental Health Concerns
Substance Use
Other type of Trauma
Self-Reported to NHTRC and BeFree
Poverty
Homelessness
Substance Use
Sexual Abuse/Assault
Child Abuse and Neglect
Runaway
Youth involved in the Child Welfare
and/or Juvenile justice System
Domestic Violence
LGBTQ
Mental Health Concerns
1
2
3
4
5
6
7
8
9
10
The data reflects only survivors who elected to disclose this information and is not the result of a systematic survey. www.polarisproject.org

## Slide 104
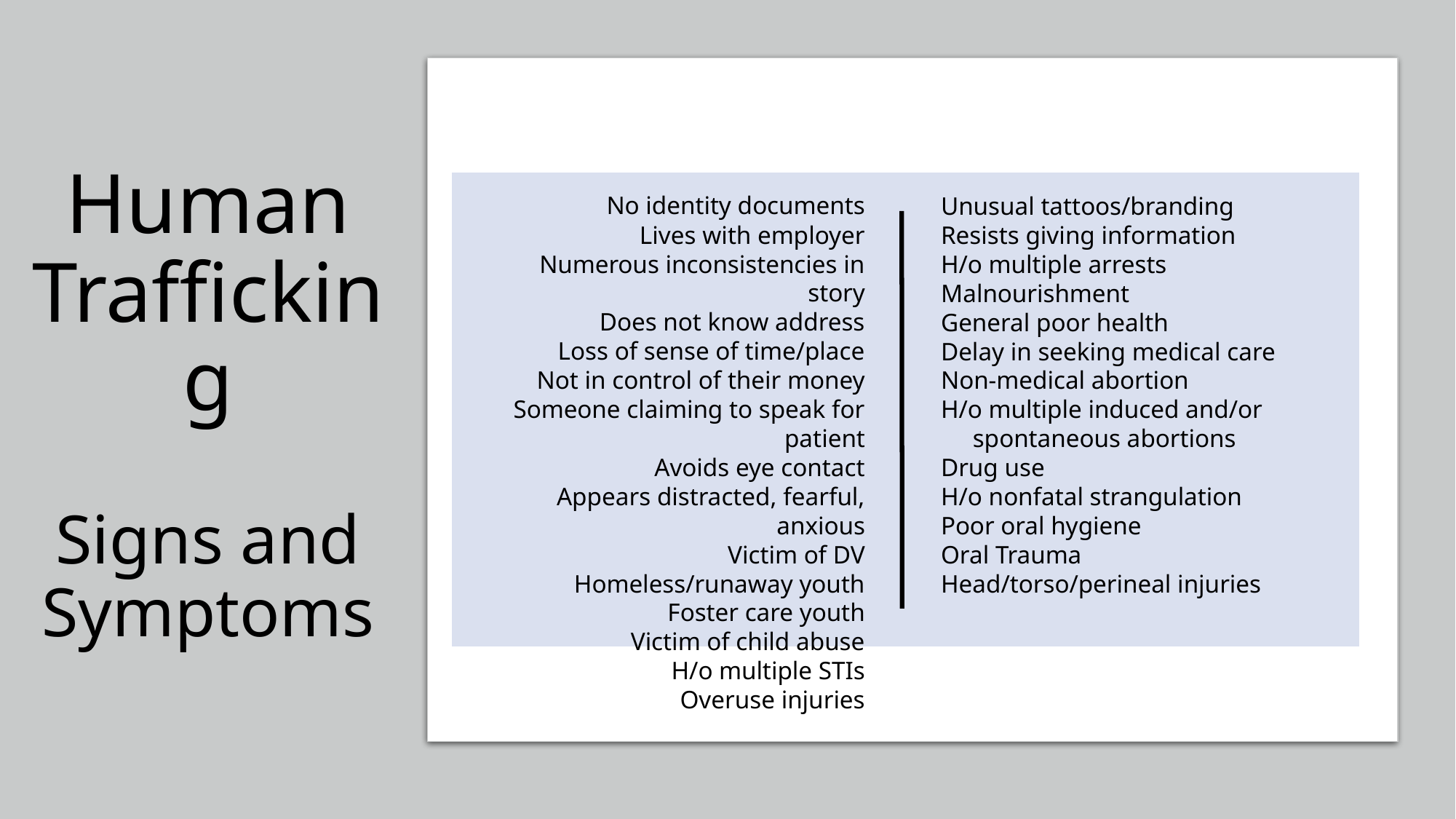

# Human Trafficking Signs and Symptoms
No identity documents
Lives with employer
Numerous inconsistencies in story
Does not know address
Loss of sense of time/place
Not in control of their money
Someone claiming to speak for patient
Avoids eye contact
Appears distracted, fearful, anxious
Victim of DV
Homeless/runaway youth
Foster care youth
Victim of child abuse
H/o multiple STIs
Overuse injuries
Unusual tattoos/branding
Resists giving information
H/o multiple arrests
Malnourishment
General poor health
Delay in seeking medical care
Non-medical abortion
H/o multiple induced and/or
 spontaneous abortions
Drug use
H/o nonfatal strangulation
Poor oral hygiene
Oral Trauma
Head/torso/perineal injuries

## Slide 105
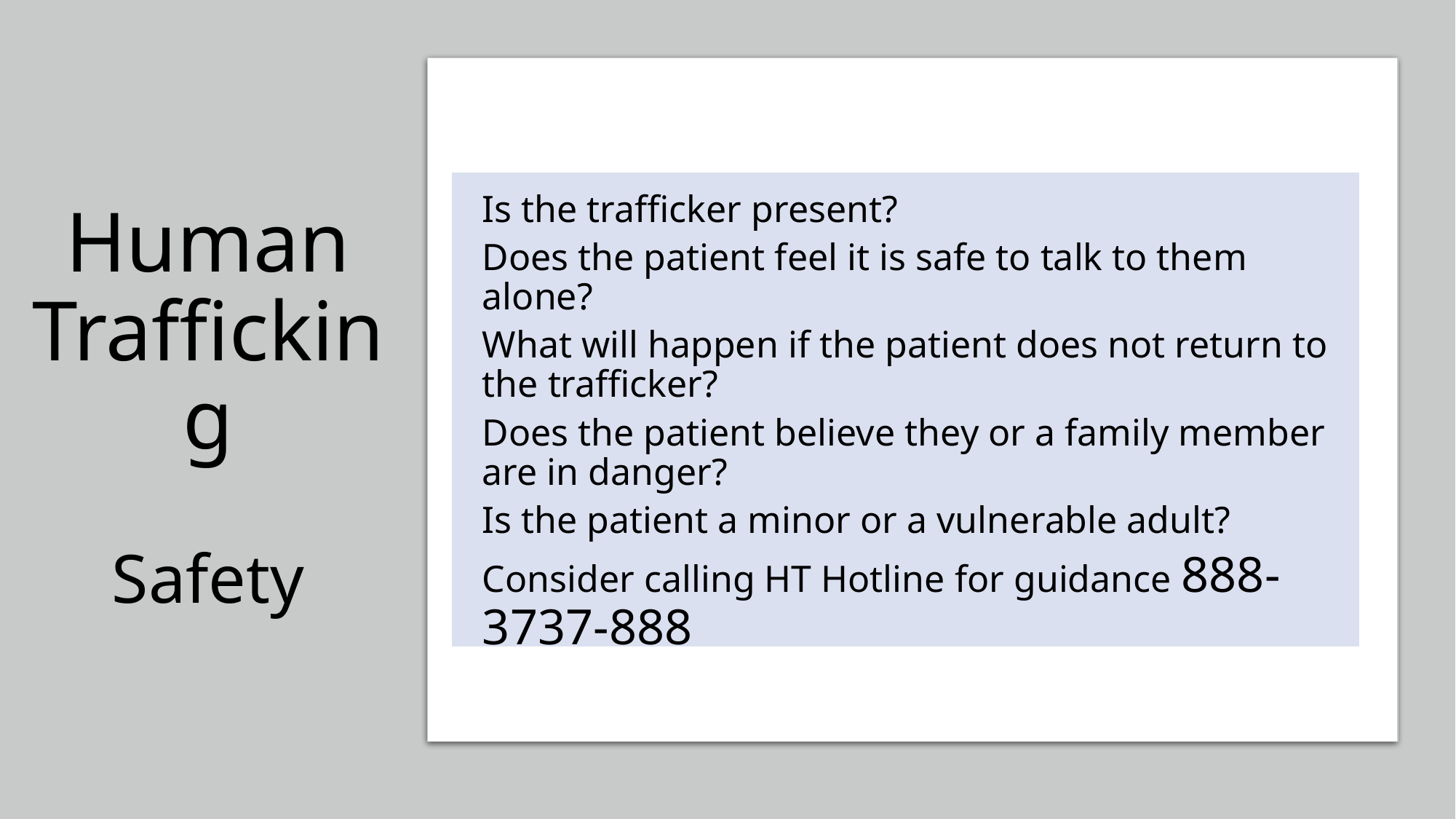

# Human Trafficking Safety
Is the trafficker present?
Does the patient feel it is safe to talk to them alone?
What will happen if the patient does not return to the trafficker?
Does the patient believe they or a family member are in danger?
Is the patient a minor or a vulnerable adult?
Consider calling HT Hotline for guidance 888-3737-888

## Slide 106
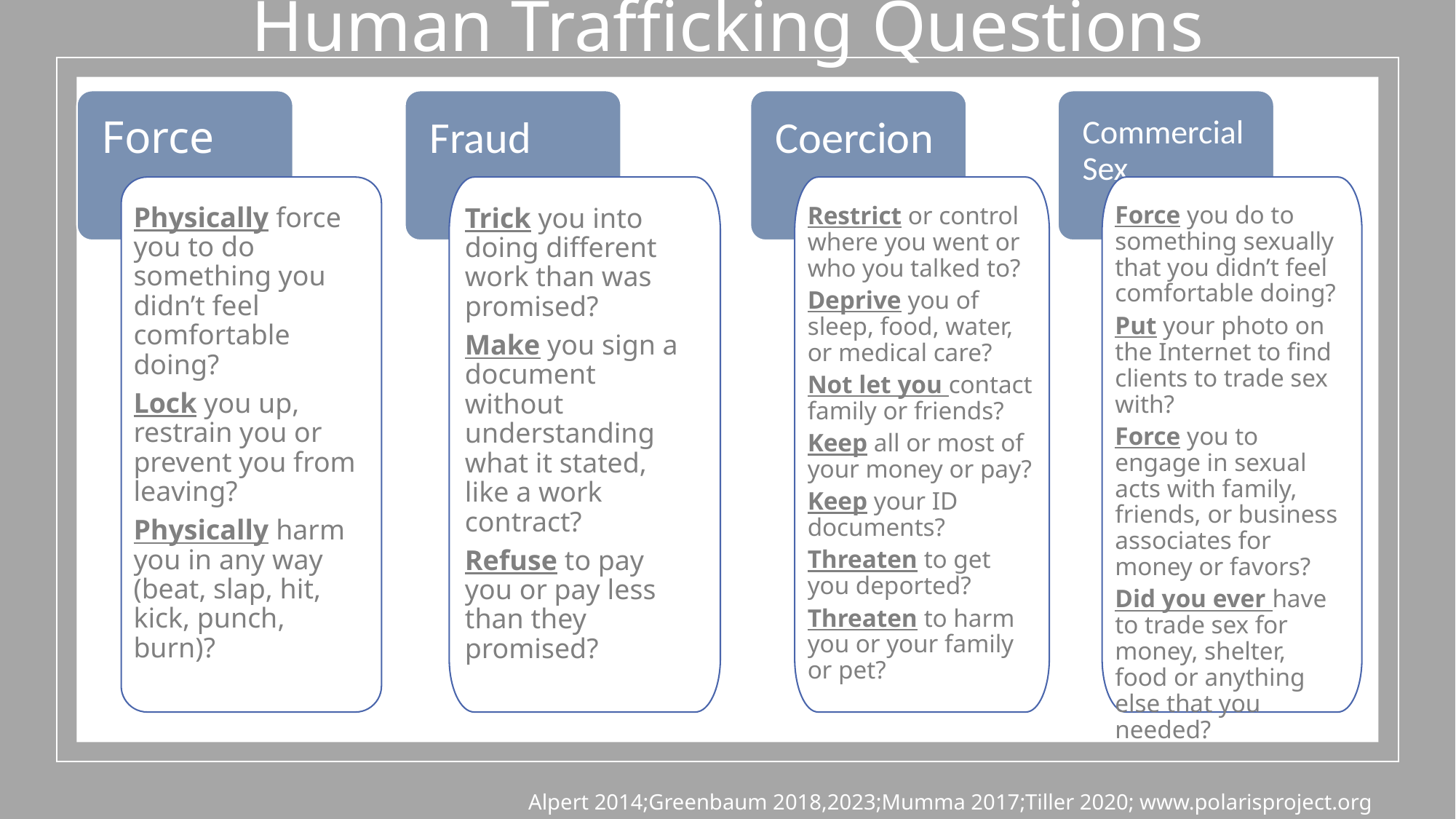

Human Trafficking Questions
Force
Fraud
Coercion
Commercial Sex
Force you do to something sexually that you didn’t feel comfortable doing?
Put your photo on the Internet to find clients to trade sex with?
Force you to engage in sexual acts with family, friends, or business associates for money or favors?
Did you ever have to trade sex for money, shelter, food or anything else that you needed?
Physically force you to do something you didn’t feel comfortable doing?
Lock you up, restrain you or prevent you from leaving?
Physically harm you in any way (beat, slap, hit, kick, punch, burn)?
Restrict or control where you went or who you talked to?
Deprive you of sleep, food, water, or medical care?
Not let you contact family or friends?
Keep all or most of your money or pay?
Keep your ID documents?
Threaten to get you deported?
Threaten to harm you or your family or pet?
Trick you into doing different work than was promised?
Make you sign a document without understanding what it stated, like a work contract?
Refuse to pay you or pay less than they promised?
Alpert 2014;Greenbaum 2018,2023;Mumma 2017;Tiller 2020; www.polarisproject.org

## Slide 107
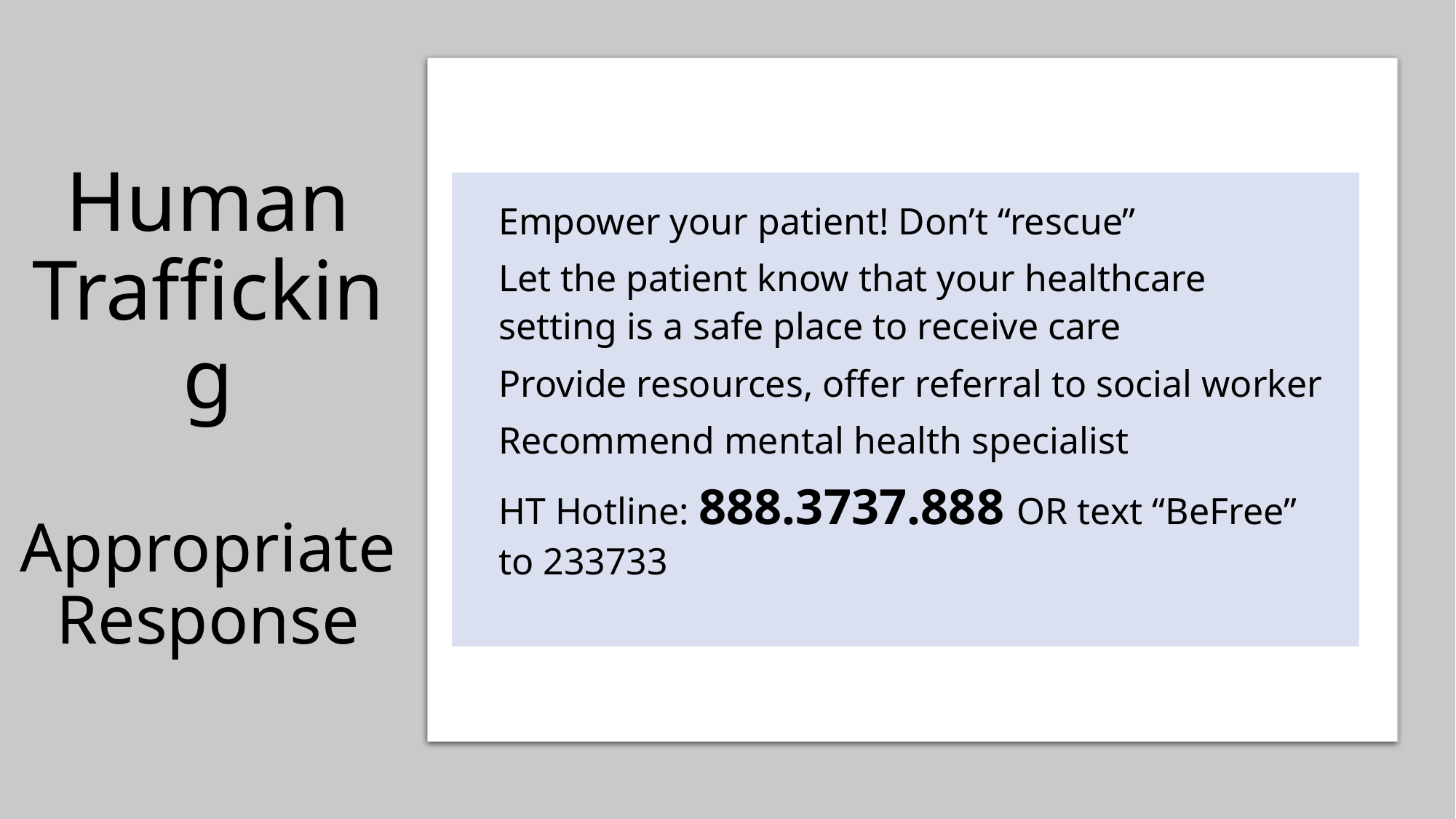

# Human Trafficking Appropriate Response
Empower your patient! Don’t “rescue”
Let the patient know that your healthcare setting is a safe place to receive care
Provide resources, offer referral to social worker
Recommend mental health specialist
HT Hotline: 888.3737.888 OR text “BeFree” to 233733

## Slide 108
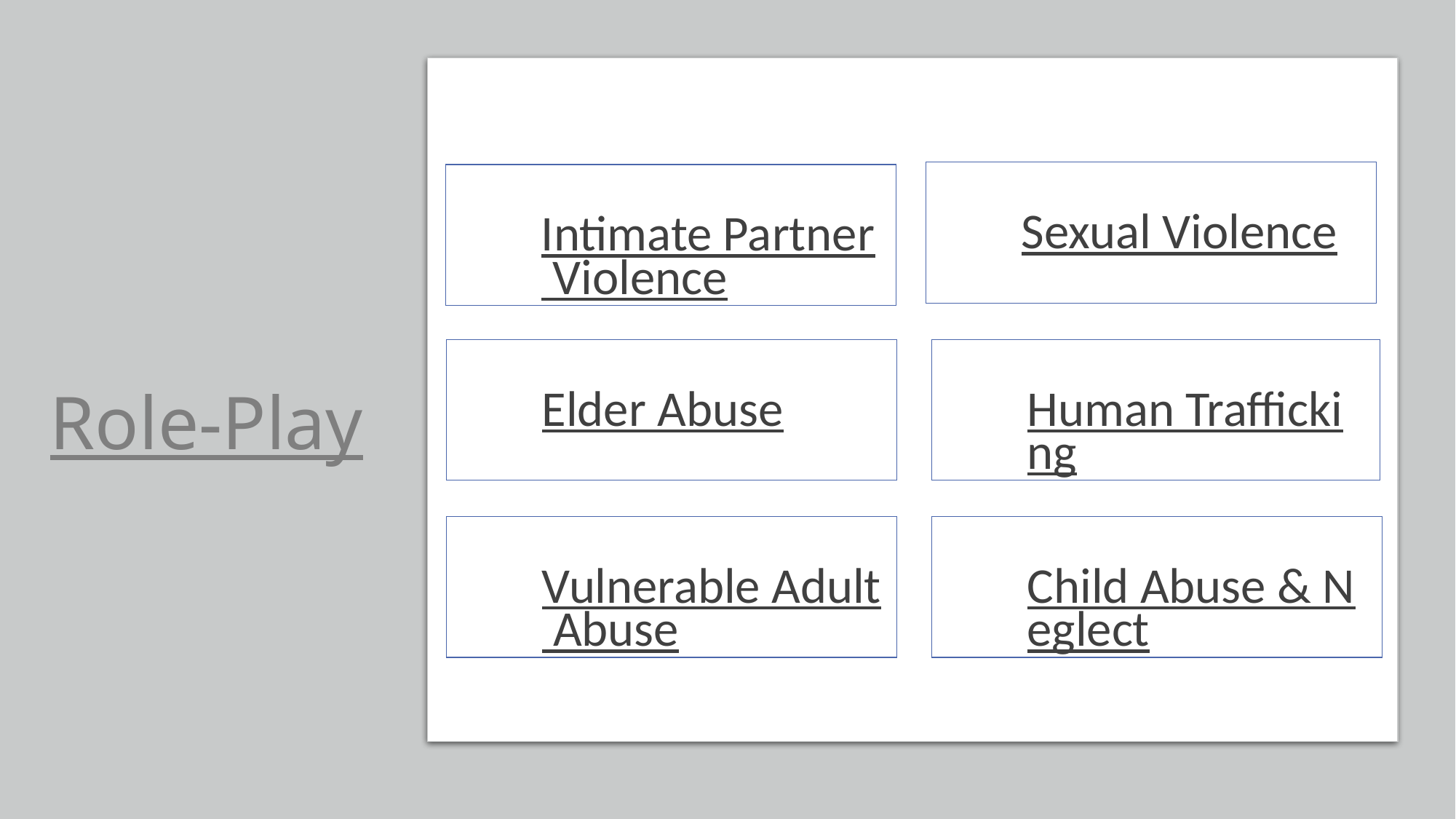

Sexual Violence
Intimate Partner Violence
# Role-Play
Elder Abuse
Human Trafficking
Vulnerable Adult Abuse
Child Abuse & Neglect

## Slide 109
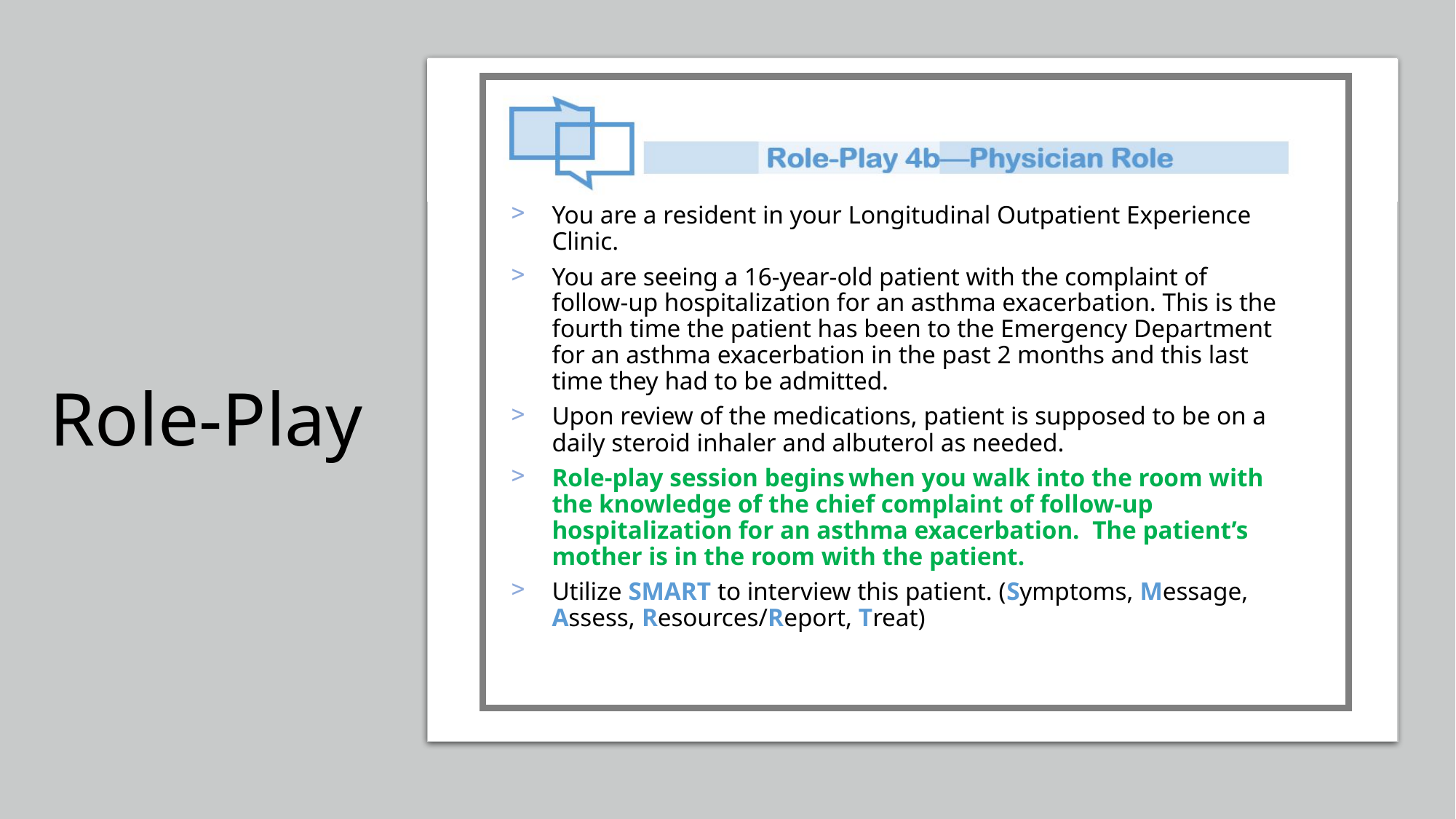

You are a resident in your Longitudinal Outpatient Experience Clinic.
You are seeing a 16-year-old patient with the complaint of follow-up hospitalization for an asthma exacerbation. This is the fourth time the patient has been to the Emergency Department for an asthma exacerbation in the past 2 months and this last time they had to be admitted.
Upon review of the medications, patient is supposed to be on a daily steroid inhaler and albuterol as needed.
Role-play session begins when you walk into the room with the knowledge of the chief complaint of follow-up hospitalization for an asthma exacerbation. The patient’s mother is in the room with the patient.
Utilize SMART to interview this patient. (Symptoms, Message, Assess, Resources/Report, Treat)
# Role-Play

## Slide 110
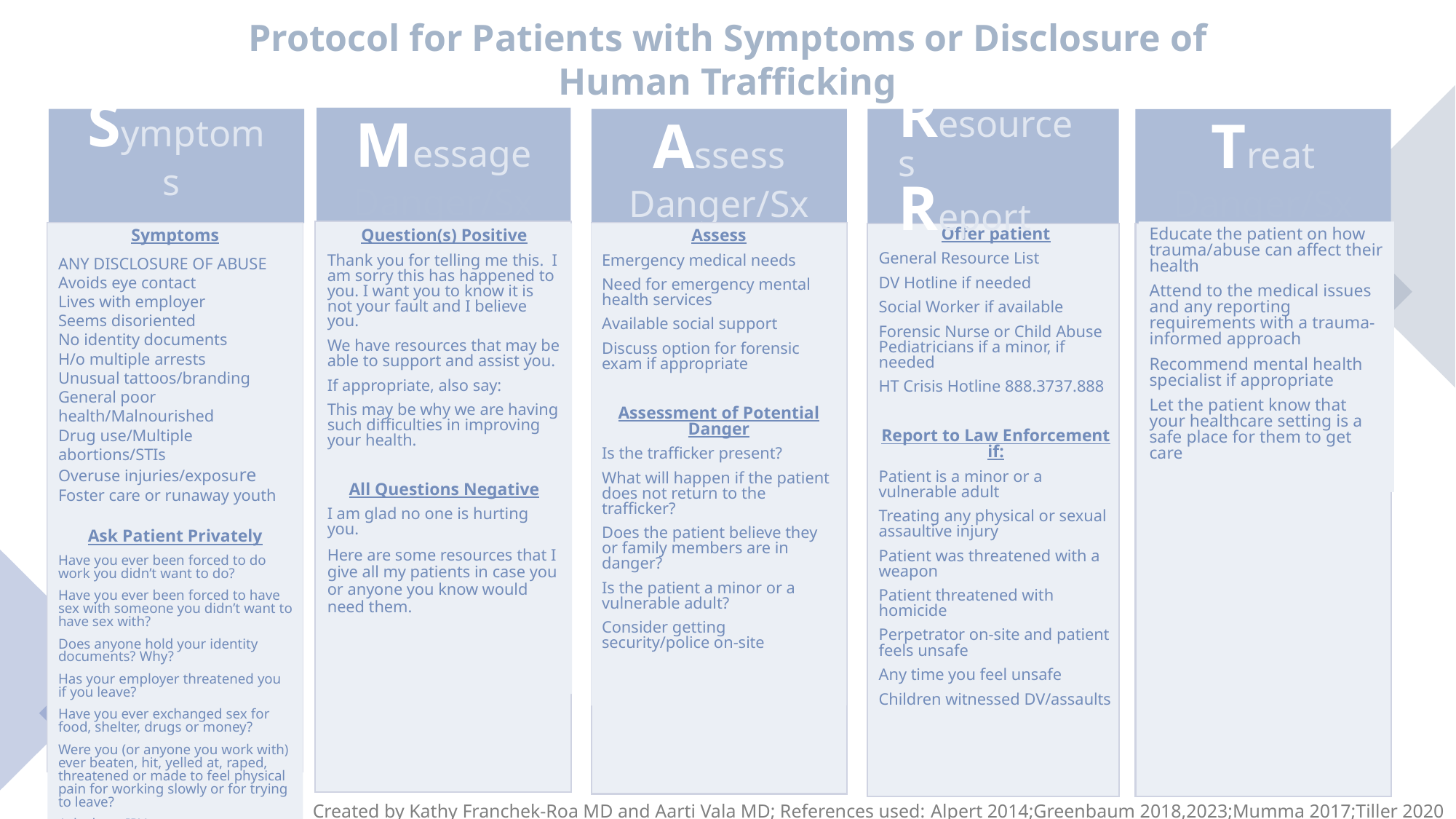

Protocol for Patients with Symptoms or Disclosure of
Human Trafficking
Message
Danger/Sx
Symptoms
Assess
Danger/Sx
Resources
Report
Treat
Danger/Sx
Educate the patient on how trauma/abuse can affect their health
Attend to the medical issues and any reporting requirements with a trauma-informed approach
Recommend mental health specialist if appropriate
Let the patient know that your healthcare setting is a safe place for them to get care
Question(s) Positive
Thank you for telling me this. I am sorry this has happened to you. I want you to know it is not your fault and I believe you.
We have resources that may be able to support and assist you.
If appropriate, also say:
This may be why we are having such difficulties in improving your health.
All Questions Negative
I am glad no one is hurting you.
Here are some resources that I give all my patients in case you or anyone you know would need them.
Offer patient
General Resource List
DV Hotline if needed
Social Worker if available
Forensic Nurse or Child Abuse Pediatricians if a minor, if needed
HT Crisis Hotline 888.3737.888
Report to Law Enforcement if:
Patient is a minor or a vulnerable adult
Treating any physical or sexual assaultive injury
Patient was threatened with a weapon
Patient threatened with homicide
Perpetrator on-site and patient feels unsafe
Any time you feel unsafe
Children witnessed DV/assaults
Assess
Emergency medical needs
Need for emergency mental health services
Available social support
Discuss option for forensic exam if appropriate
Assessment of Potential Danger
Is the trafficker present?
What will happen if the patient does not return to the trafficker?
Does the patient believe they or family members are in danger?
Is the patient a minor or a vulnerable adult?
Consider getting security/police on-site
Symptoms
ANY DISCLOSURE OF ABUSE
Avoids eye contact
Lives with employer
Seems disoriented
No identity documents
H/o multiple arrests
Unusual tattoos/branding
General poor health/Malnourished
Drug use/Multiple abortions/STIs
Overuse injuries/exposure
Foster care or runaway youth
Ask Patient Privately
Have you ever been forced to do work you didn’t want to do?
Have you ever been forced to have sex with someone you didn’t want to have sex with?
Does anyone hold your identity documents? Why?
Has your employer threatened you if you leave?
Have you ever exchanged sex for food, shelter, drugs or money?
Were you (or anyone you work with) ever beaten, hit, yelled at, raped, threatened or made to feel physical pain for working slowly or for trying to leave?
Ask about IPV
Created by Kathy Franchek-Roa MD and Aarti Vala MD; References used: Alpert 2014;Greenbaum 2018,2023;Mumma 2017;Tiller 2020

## Slide 111
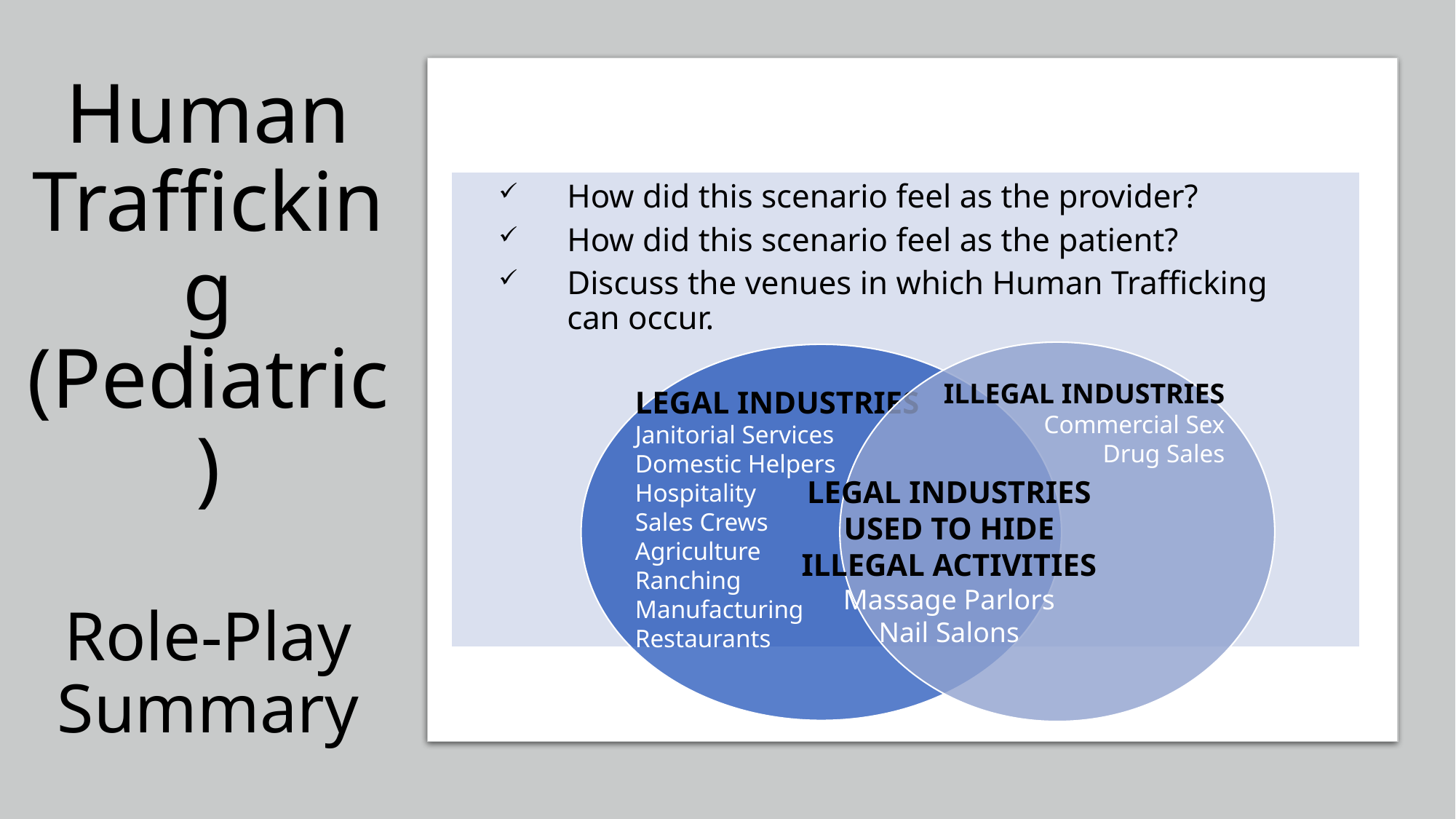

# Human Trafficking (Pediatric) Role-Play Summary
How did this scenario feel as the provider?
How did this scenario feel as the patient?
Discuss the venues in which Human Trafficking can occur.
Illegal Industries
Commercial Sex
Drug Sales
Legal Industries
Janitorial Services
Domestic Helpers
Hospitality
Sales Crews
Agriculture
Ranching
Manufacturing
Restaurants
Legal Industries
Used to Hide
Illegal Activities
Massage Parlors
Nail Salons

## Slide 112
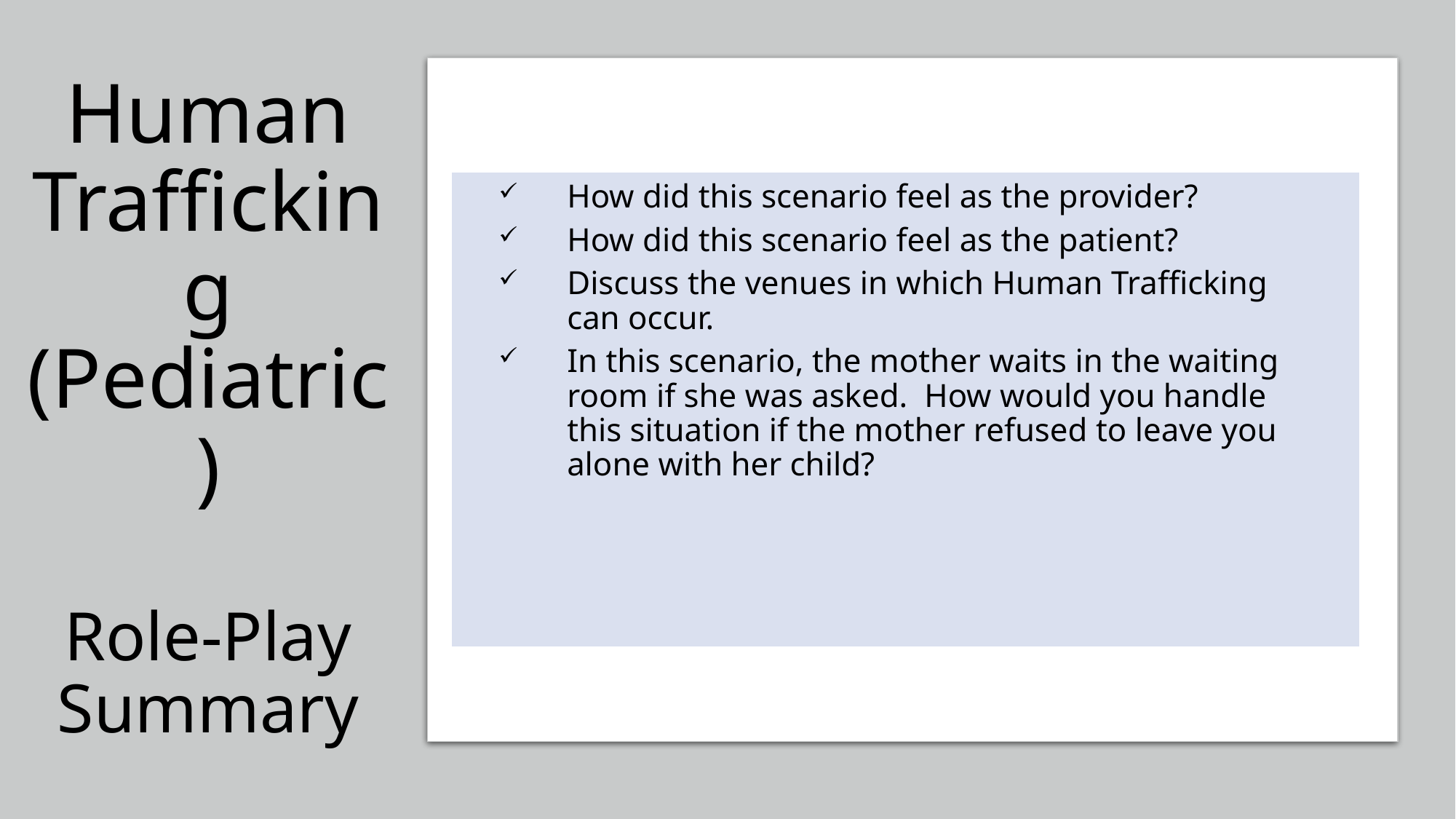

# Human Trafficking (Pediatric) Role-Play Summary
How did this scenario feel as the provider?
How did this scenario feel as the patient?
Discuss the venues in which Human Trafficking can occur.
In this scenario, the mother waits in the waiting room if she was asked. How would you handle this situation if the mother refused to leave you alone with her child?

## Slide 113
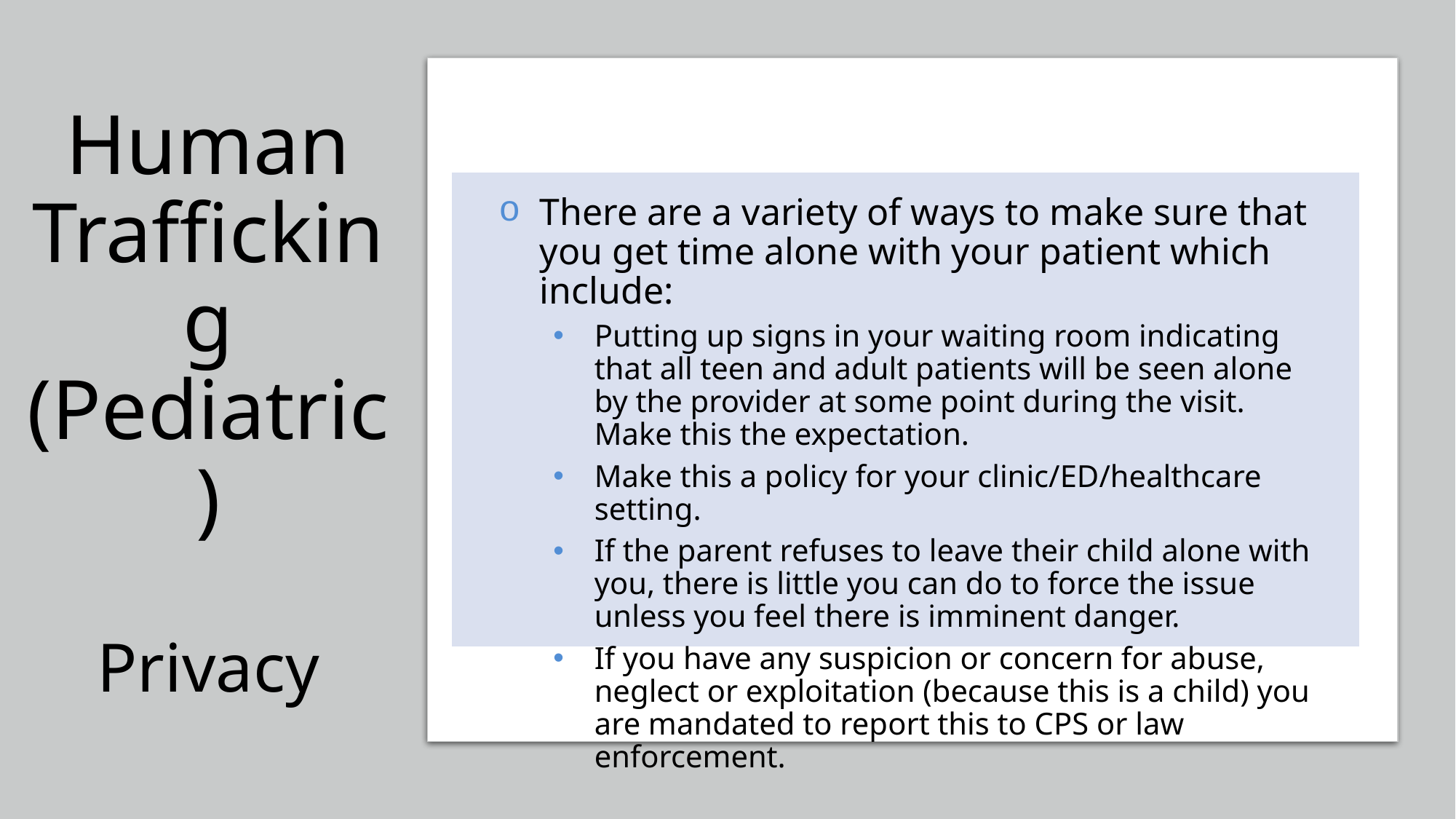

# Human Trafficking (Pediatric) Privacy
There are a variety of ways to make sure that you get time alone with your patient which include:
Putting up signs in your waiting room indicating that all teen and adult patients will be seen alone by the provider at some point during the visit. Make this the expectation.
Make this a policy for your clinic/ED/healthcare setting.
If the parent refuses to leave their child alone with you, there is little you can do to force the issue unless you feel there is imminent danger.
If you have any suspicion or concern for abuse, neglect or exploitation (because this is a child) you are mandated to report this to CPS or law enforcement.

## Slide 114
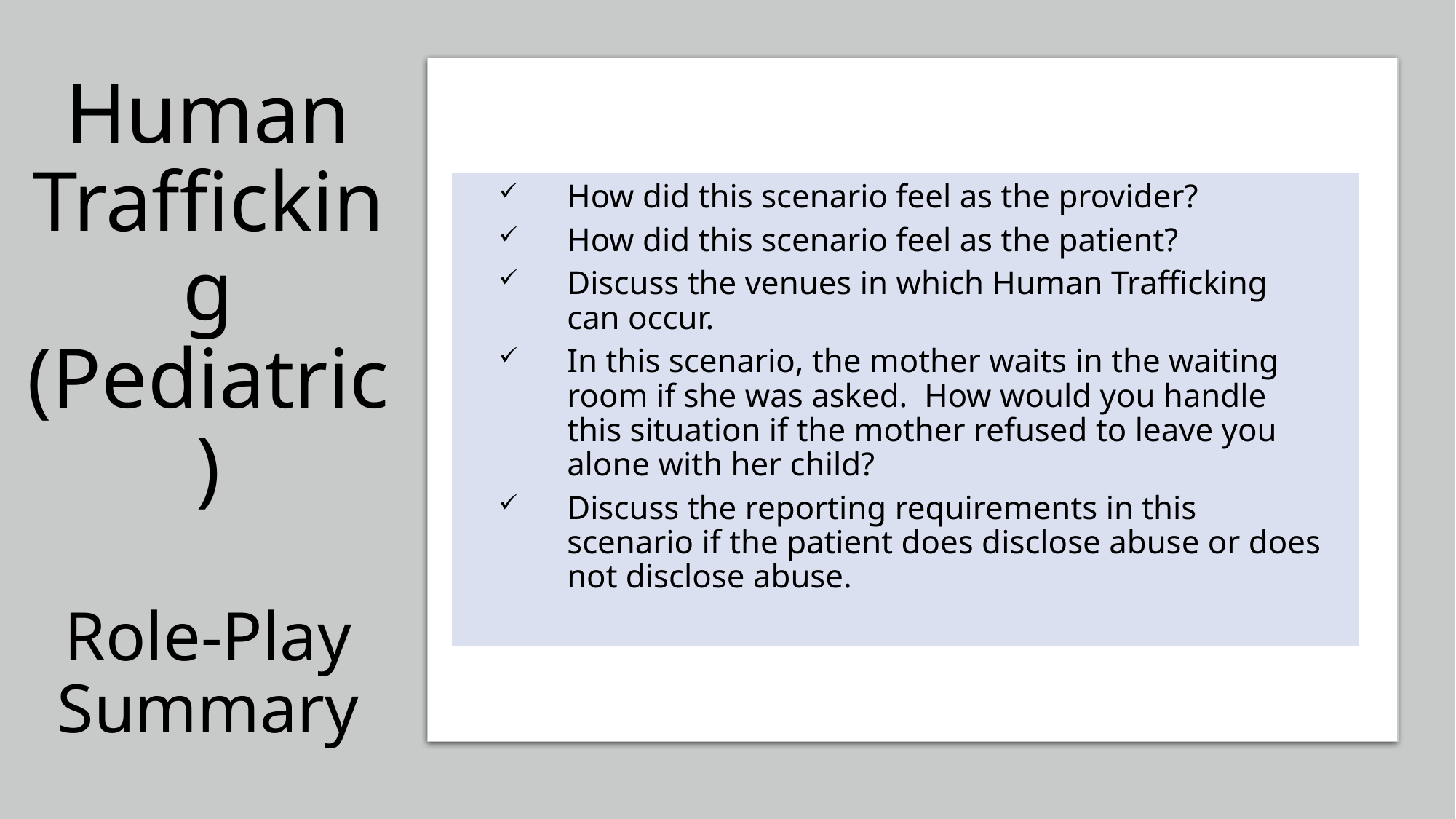

# Human Trafficking (Pediatric) Role-Play Summary
How did this scenario feel as the provider?
How did this scenario feel as the patient?
Discuss the venues in which Human Trafficking can occur.
In this scenario, the mother waits in the waiting room if she was asked. How would you handle this situation if the mother refused to leave you alone with her child?
Discuss the reporting requirements in this scenario if the patient does disclose abuse or does not disclose abuse.

## Slide 115
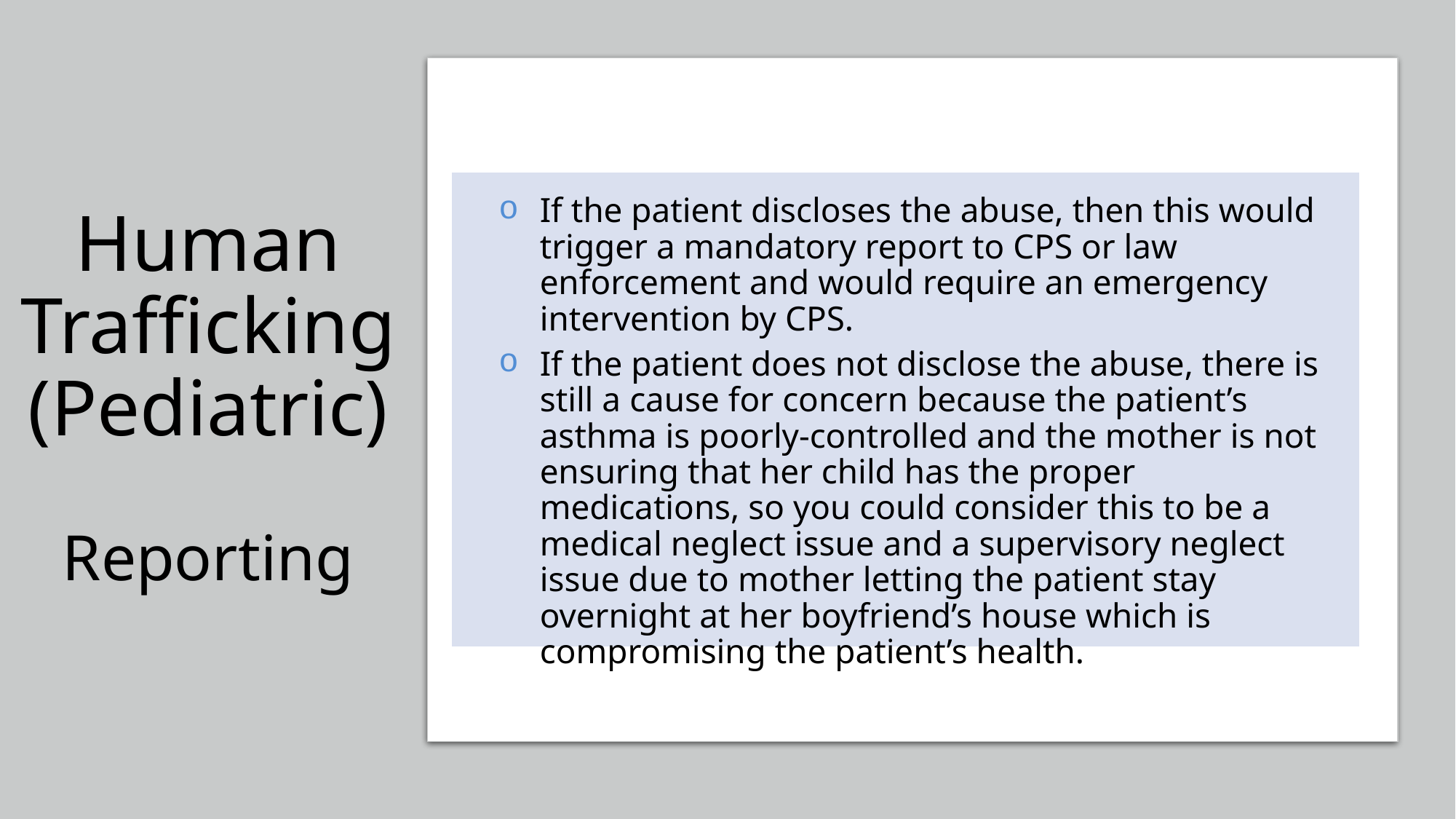

If the patient discloses the abuse, then this would trigger a mandatory report to CPS or law enforcement and would require an emergency intervention by CPS.
If the patient does not disclose the abuse, there is still a cause for concern because the patient’s asthma is poorly-controlled and the mother is not ensuring that her child has the proper medications, so you could consider this to be a medical neglect issue and a supervisory neglect issue due to mother letting the patient stay overnight at her boyfriend’s house which is compromising the patient’s health.
# Human Trafficking (Pediatric) Reporting

## Slide 116
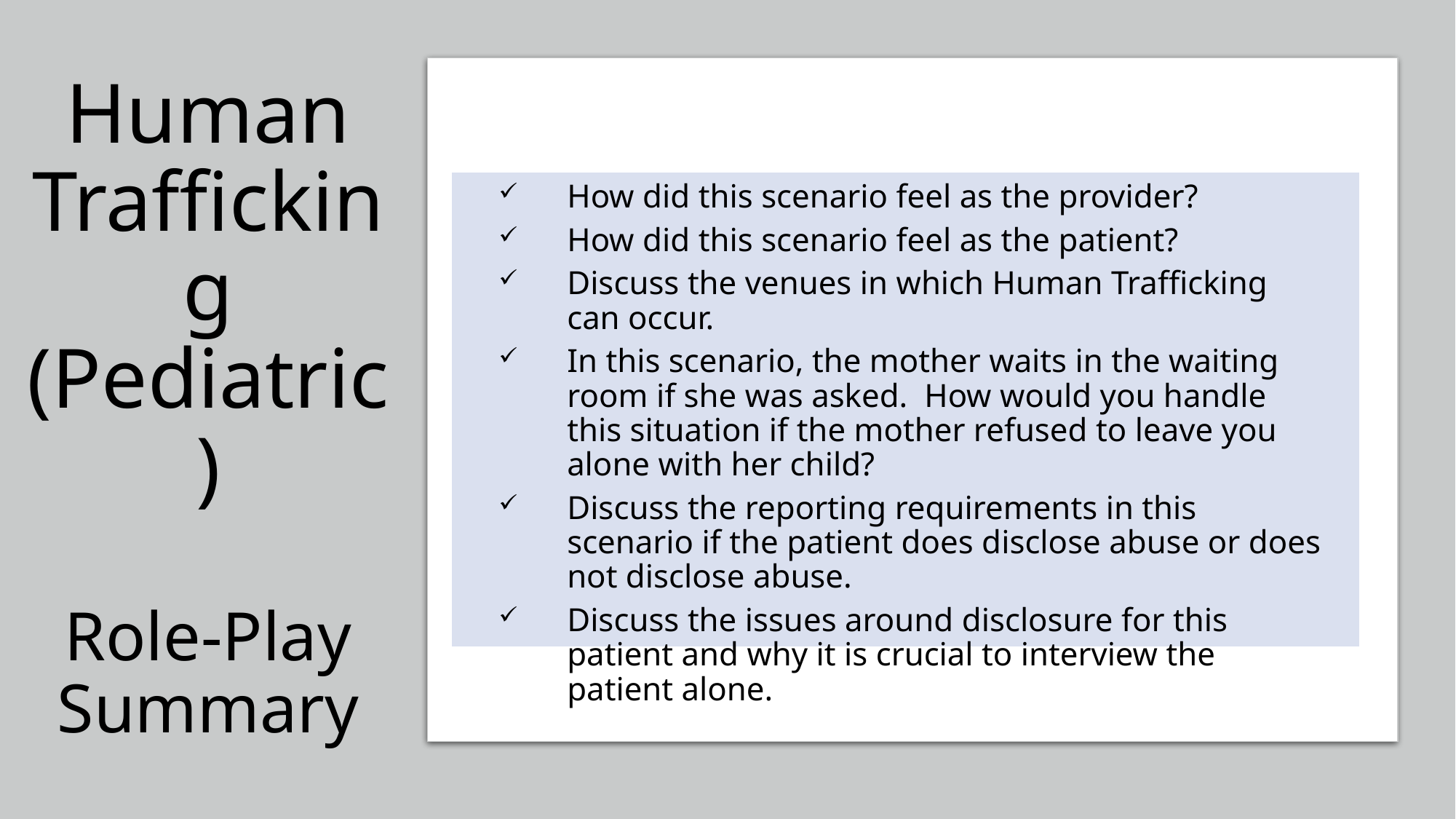

# Human Trafficking (Pediatric) Role-Play Summary
How did this scenario feel as the provider?
How did this scenario feel as the patient?
Discuss the venues in which Human Trafficking can occur.
In this scenario, the mother waits in the waiting room if she was asked. How would you handle this situation if the mother refused to leave you alone with her child?
Discuss the reporting requirements in this scenario if the patient does disclose abuse or does not disclose abuse.
Discuss the issues around disclosure for this patient and why it is crucial to interview the patient alone.

## Slide 117
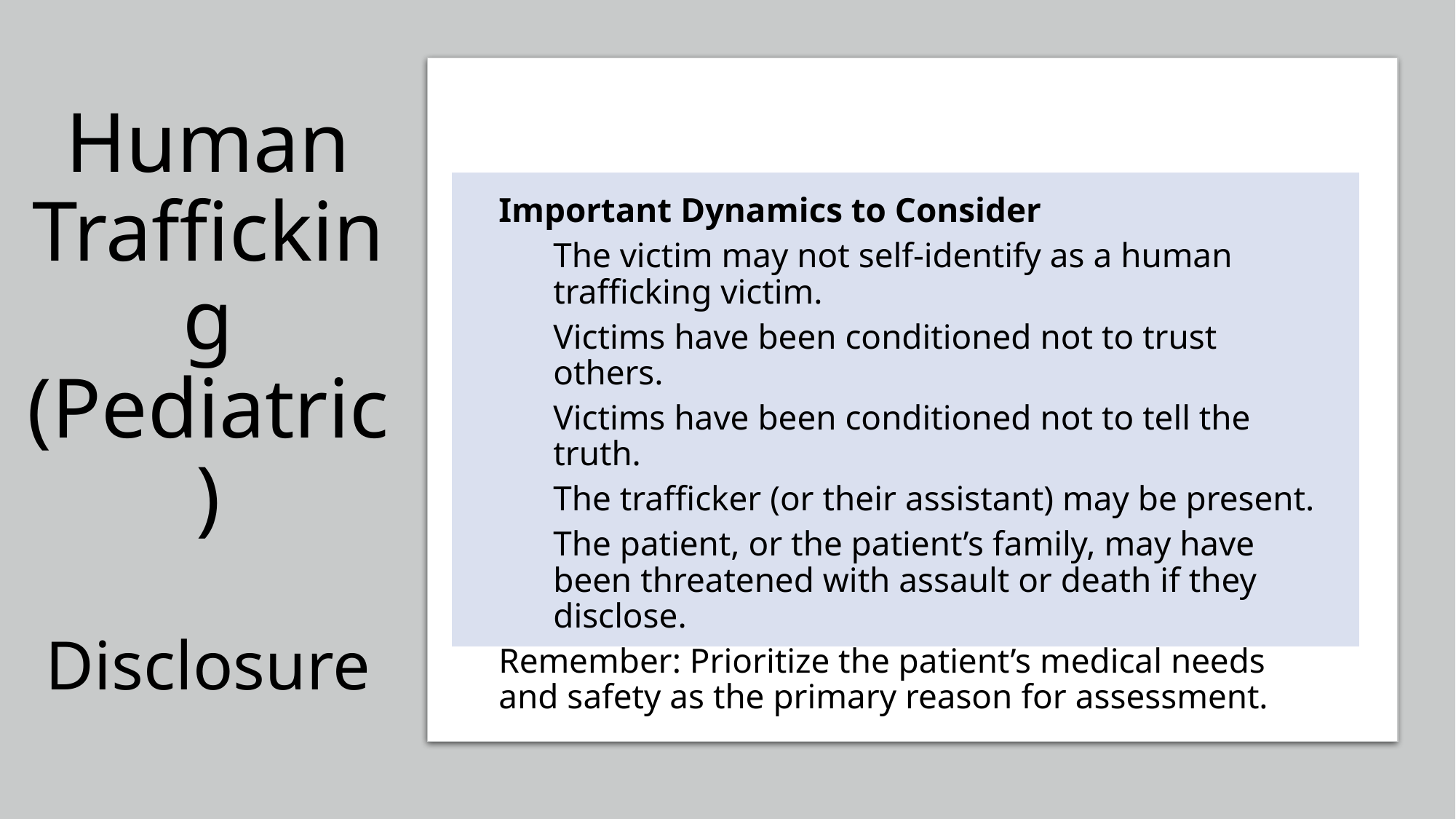

# Human Trafficking (Pediatric) Disclosure
Important Dynamics to Consider
The victim may not self-identify as a human trafficking victim.
Victims have been conditioned not to trust others.
Victims have been conditioned not to tell the truth.
The trafficker (or their assistant) may be present.
The patient, or the patient’s family, may have been threatened with assault or death if they disclose.
Remember: Prioritize the patient’s medical needs and safety as the primary reason for assessment.

## Slide 118
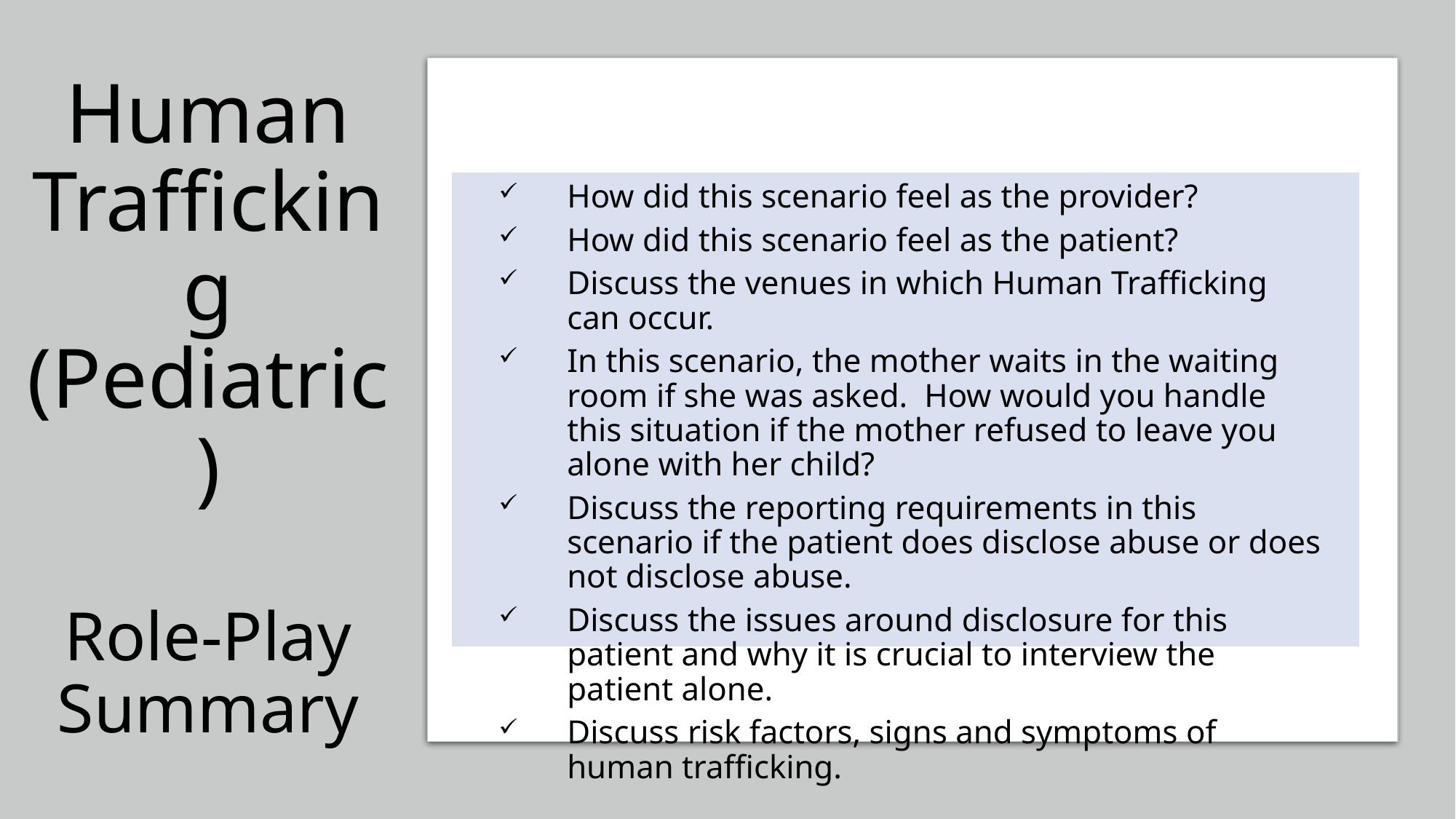

# Human Trafficking (Pediatric) Role-Play Summary
How did this scenario feel as the provider?
How did this scenario feel as the patient?
Discuss the venues in which Human Trafficking can occur.
In this scenario, the mother waits in the waiting room if she was asked. How would you handle this situation if the mother refused to leave you alone with her child?
Discuss the reporting requirements in this scenario if the patient does disclose abuse or does not disclose abuse.
Discuss the issues around disclosure for this patient and why it is crucial to interview the patient alone.
Discuss risk factors, signs and symptoms of human trafficking.

## Slide 119
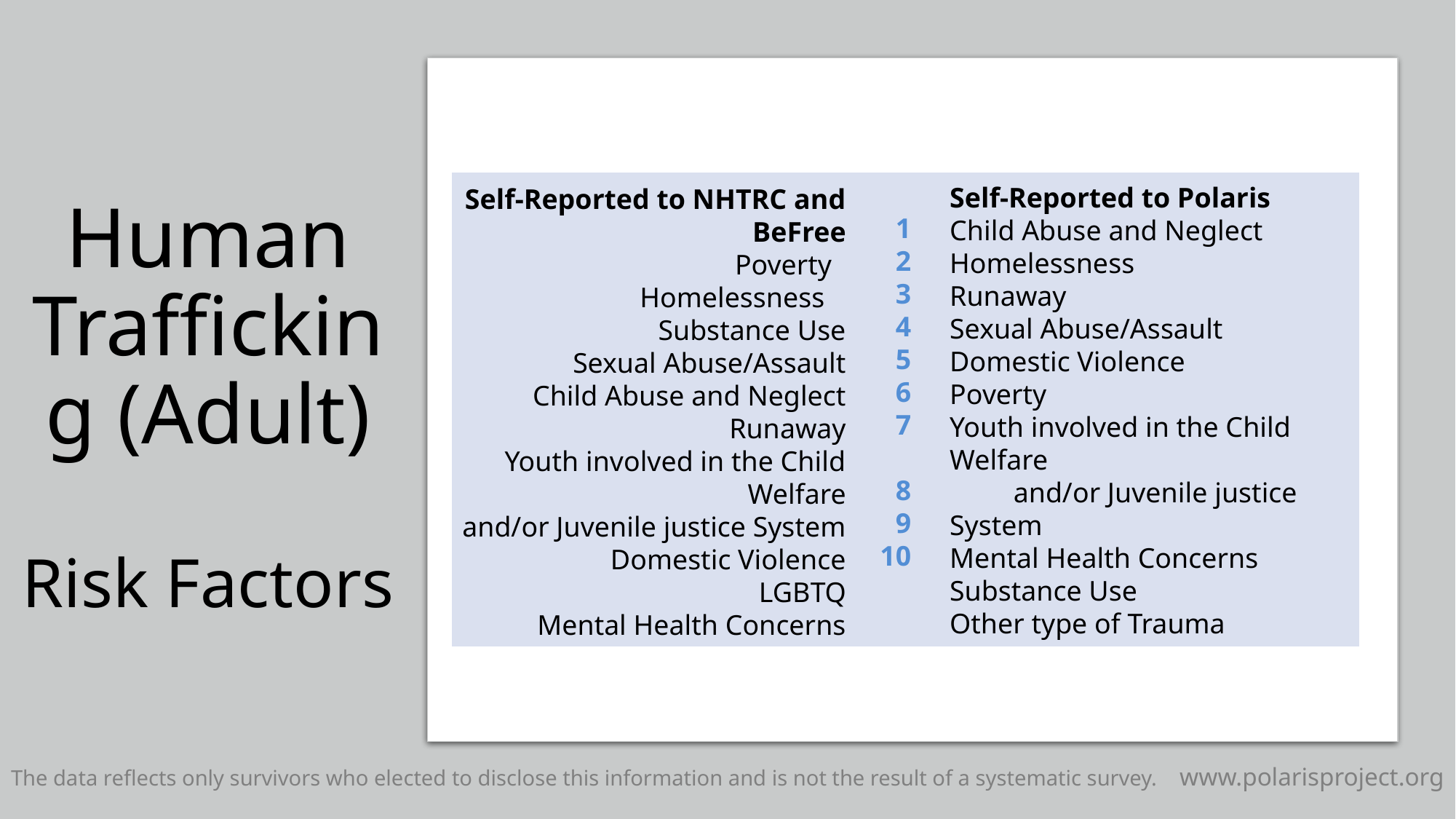

# Human Trafficking (Adult) Risk Factors
Self-Reported to Polaris
Child Abuse and Neglect
Homelessness
Runaway
Sexual Abuse/Assault
Domestic Violence
Poverty
Youth involved in the Child Welfare
 and/or Juvenile justice System
Mental Health Concerns
Substance Use
Other type of Trauma
Self-Reported to NHTRC and BeFree
Poverty
Homelessness
Substance Use
Sexual Abuse/Assault
Child Abuse and Neglect
Runaway
Youth involved in the Child Welfare
and/or Juvenile justice System
Domestic Violence
LGBTQ
Mental Health Concerns
1
2
3
4
5
6
7
8
9
10
The data reflects only survivors who elected to disclose this information and is not the result of a systematic survey. www.polarisproject.org

## Slide 120
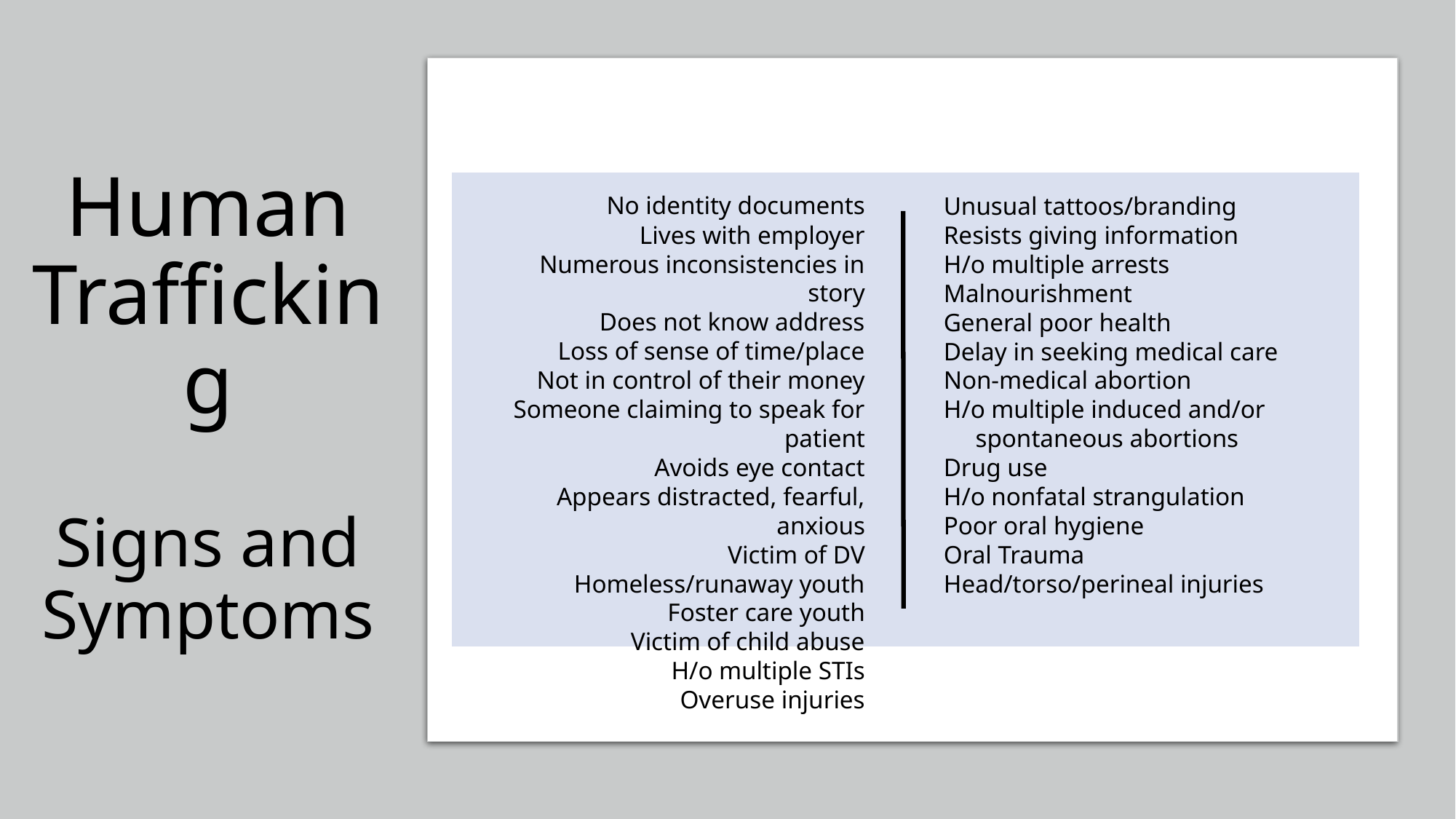

# Human Trafficking Signs and Symptoms
No identity documents
Lives with employer
Numerous inconsistencies in story
Does not know address
Loss of sense of time/place
Not in control of their money
Someone claiming to speak for patient
Avoids eye contact
Appears distracted, fearful, anxious
Victim of DV
Homeless/runaway youth
Foster care youth
Victim of child abuse
H/o multiple STIs
Overuse injuries
Unusual tattoos/branding
Resists giving information
H/o multiple arrests
Malnourishment
General poor health
Delay in seeking medical care
Non-medical abortion
H/o multiple induced and/or
 spontaneous abortions
Drug use
H/o nonfatal strangulation
Poor oral hygiene
Oral Trauma
Head/torso/perineal injuries

## Slide 121
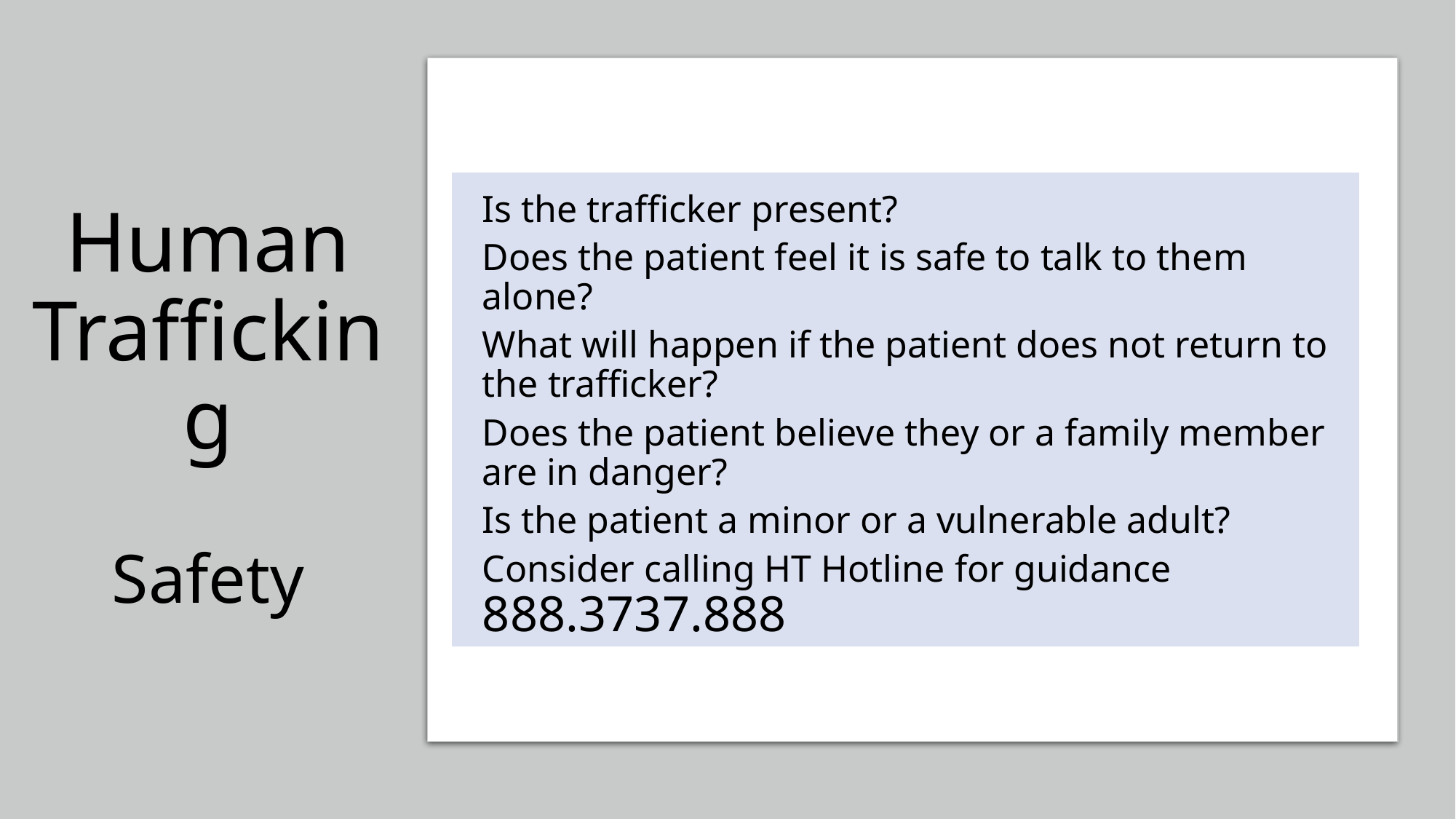

# Human Trafficking Safety
Is the trafficker present?
Does the patient feel it is safe to talk to them alone?
What will happen if the patient does not return to the trafficker?
Does the patient believe they or a family member are in danger?
Is the patient a minor or a vulnerable adult?
Consider calling HT Hotline for guidance 888.3737.888

## Slide 122
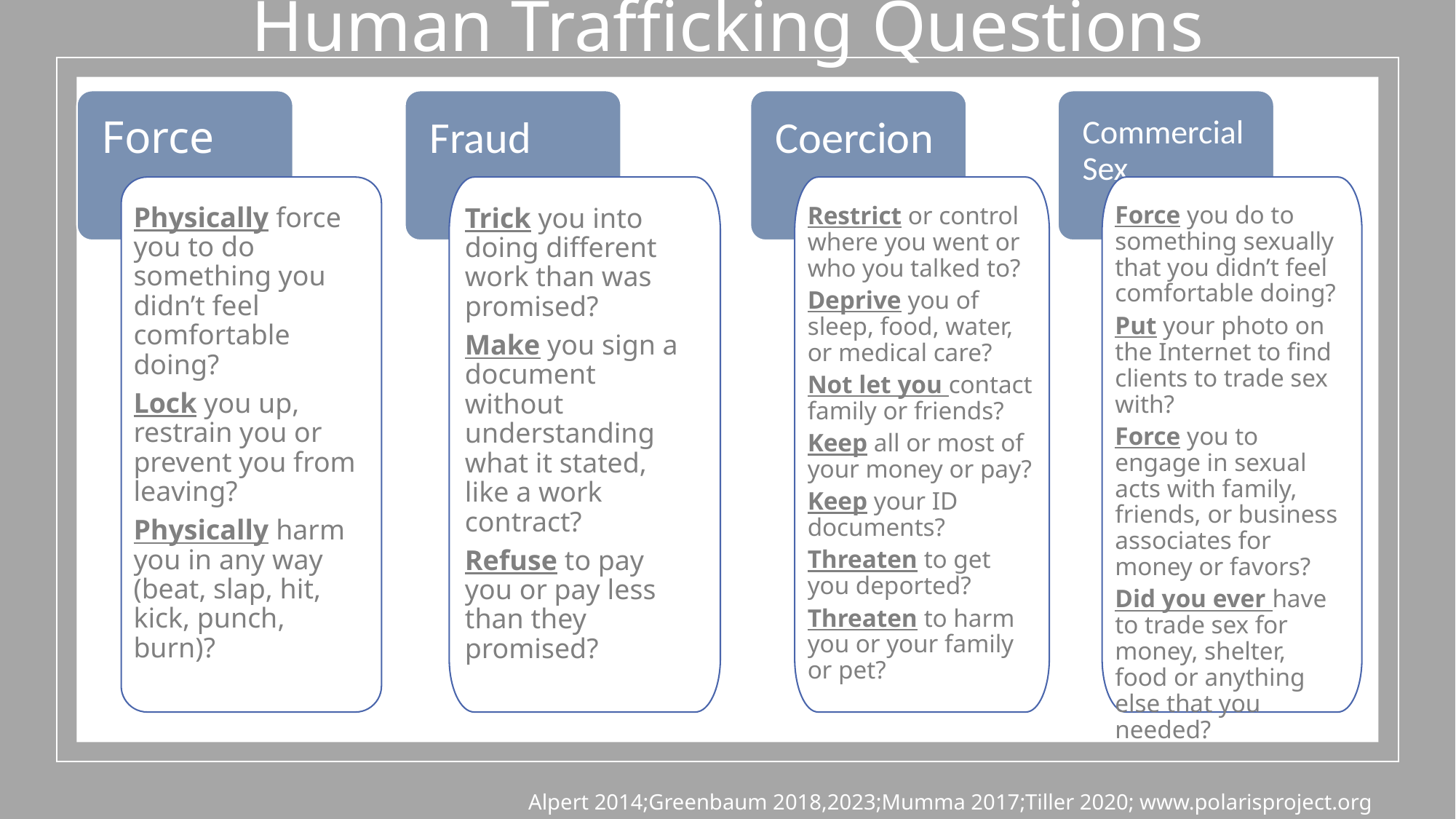

Human Trafficking Questions
Force
Fraud
Coercion
Commercial Sex
Force you do to something sexually that you didn’t feel comfortable doing?
Put your photo on the Internet to find clients to trade sex with?
Force you to engage in sexual acts with family, friends, or business associates for money or favors?
Did you ever have to trade sex for money, shelter, food or anything else that you needed?
Physically force you to do something you didn’t feel comfortable doing?
Lock you up, restrain you or prevent you from leaving?
Physically harm you in any way (beat, slap, hit, kick, punch, burn)?
Restrict or control where you went or who you talked to?
Deprive you of sleep, food, water, or medical care?
Not let you contact family or friends?
Keep all or most of your money or pay?
Keep your ID documents?
Threaten to get you deported?
Threaten to harm you or your family or pet?
Trick you into doing different work than was promised?
Make you sign a document without understanding what it stated, like a work contract?
Refuse to pay you or pay less than they promised?
Alpert 2014;Greenbaum 2018,2023;Mumma 2017;Tiller 2020; www.polarisproject.org

## Slide 123
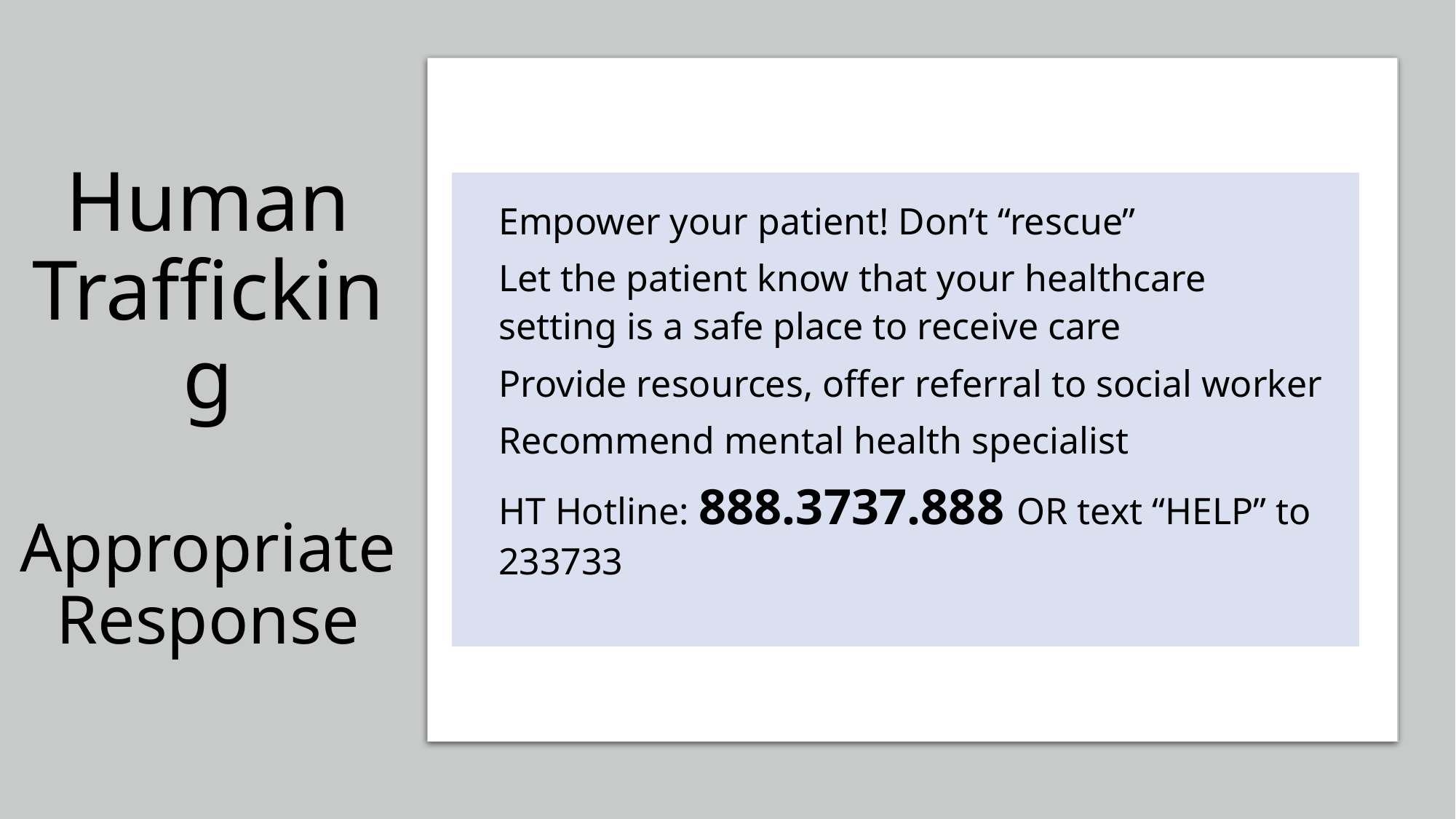

# Human Trafficking Appropriate Response
Empower your patient! Don’t “rescue”
Let the patient know that your healthcare setting is a safe place to receive care
Provide resources, offer referral to social worker
Recommend mental health specialist
HT Hotline: 888.3737.888 OR text “HELP” to 233733

## Slide 124
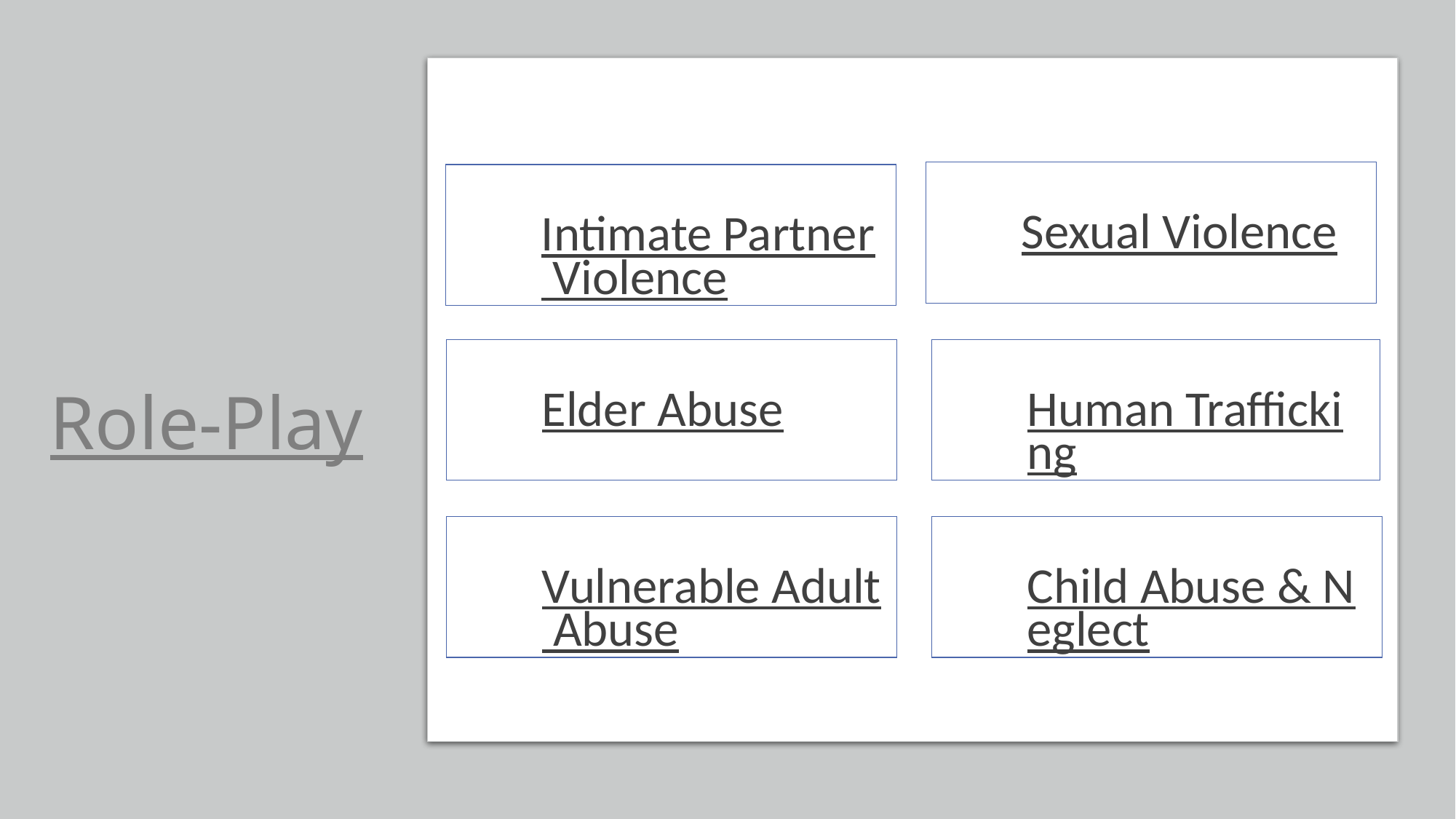

Sexual Violence
Intimate Partner Violence
# Role-Play
Elder Abuse
Human Trafficking
Vulnerable Adult Abuse
Child Abuse & Neglect

## Slide 125
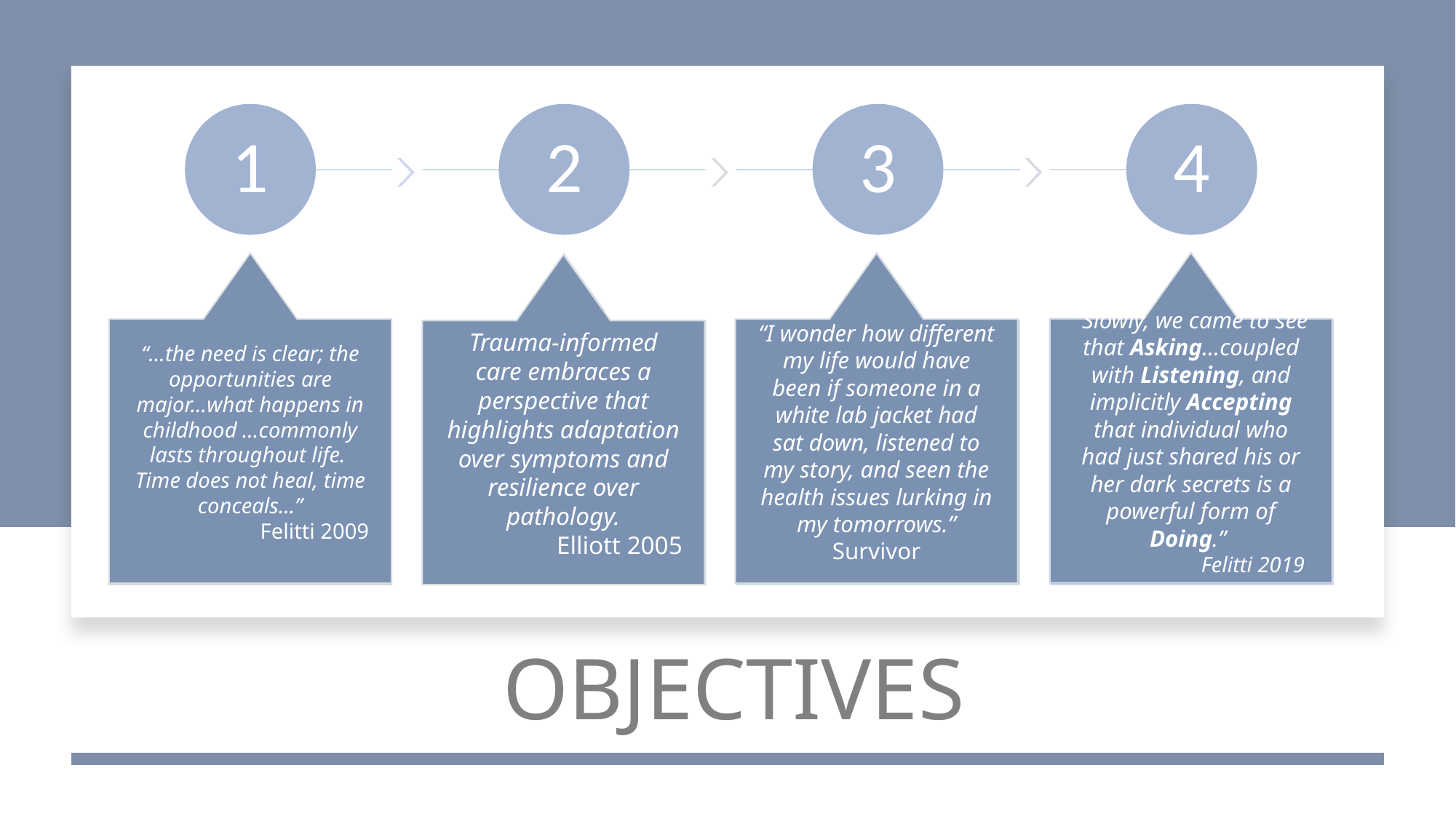

2
Utilize a trauma-informed approach when interacting with patients to reduce or mitigate the consequences of these adverse experiences
3
Discuss the prevalence of abuse, neglect, and/or exploitation in terms of the public health impact to healthcare
4
Employ best practices when evaluating patients who are victims of abuse, neglect, and/or exploitation
1
Explain the link between childhood adversity and risk for poor health across the lifespan
“Slowly, we came to see that Asking…coupled with Listening, and implicitly Accepting that individual who had just shared his or her dark secrets is a powerful form of Doing.”
Felitti 2019
“I wonder how different my life would have been if someone in a white lab jacket had sat down, listened to my story, and seen the health issues lurking in my tomorrows.” Survivor
“…the need is clear; the opportunities are major…what happens in childhood …commonly lasts throughout life. Time does not heal, time conceals...”
Felitti 2009
Trauma-informed care embraces a perspective that highlights adaptation over symptoms and resilience over pathology.
Elliott 2005
OBJECTIVES

## Slide 126
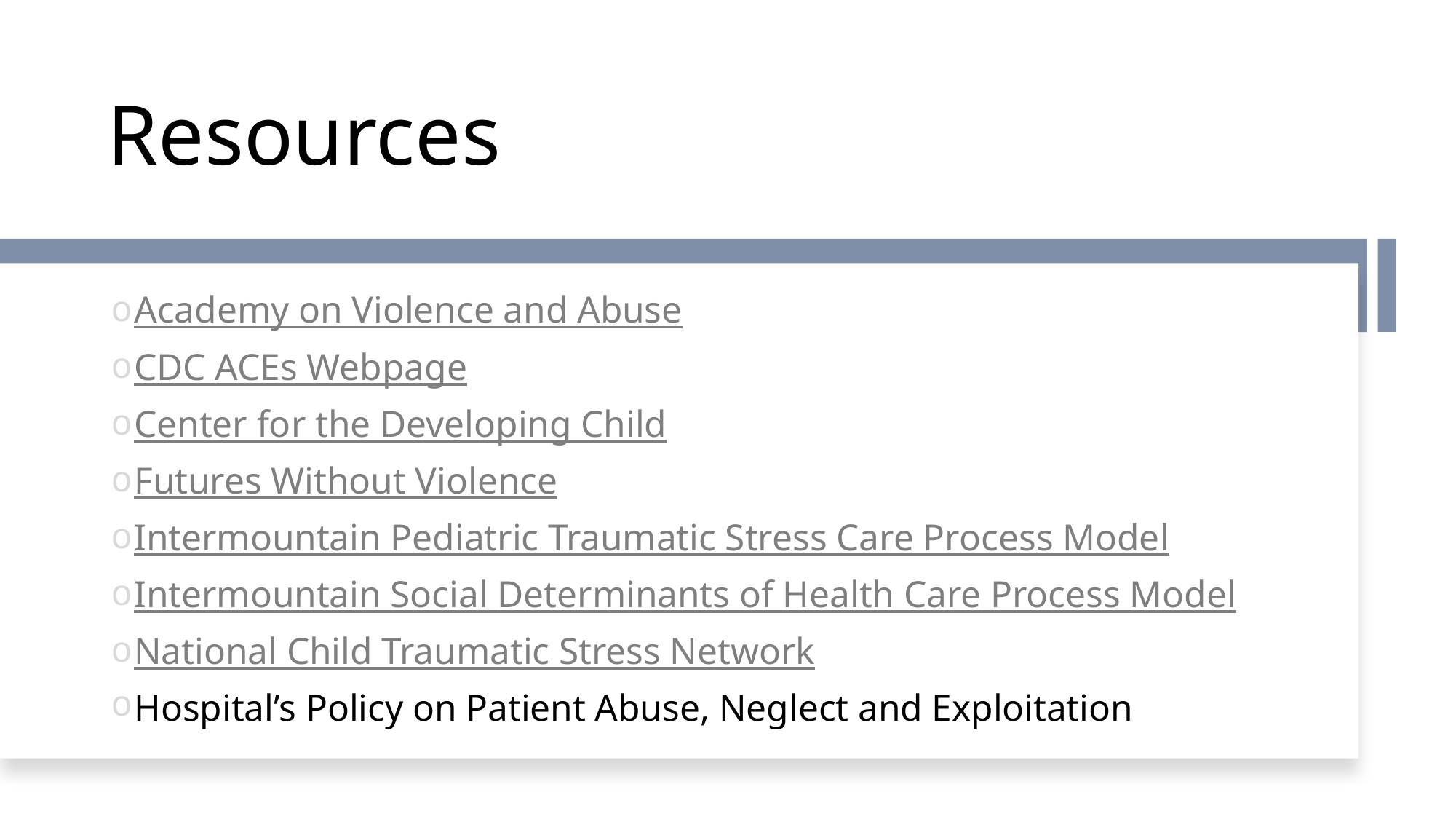

# Resources
Academy on Violence and Abuse
CDC ACEs Webpage
Center for the Developing Child
Futures Without Violence
Intermountain Pediatric Traumatic Stress Care Process Model
Intermountain Social Determinants of Health Care Process Model
National Child Traumatic Stress Network
Hospital’s Policy on Patient Abuse, Neglect and Exploitation

## Slide 127
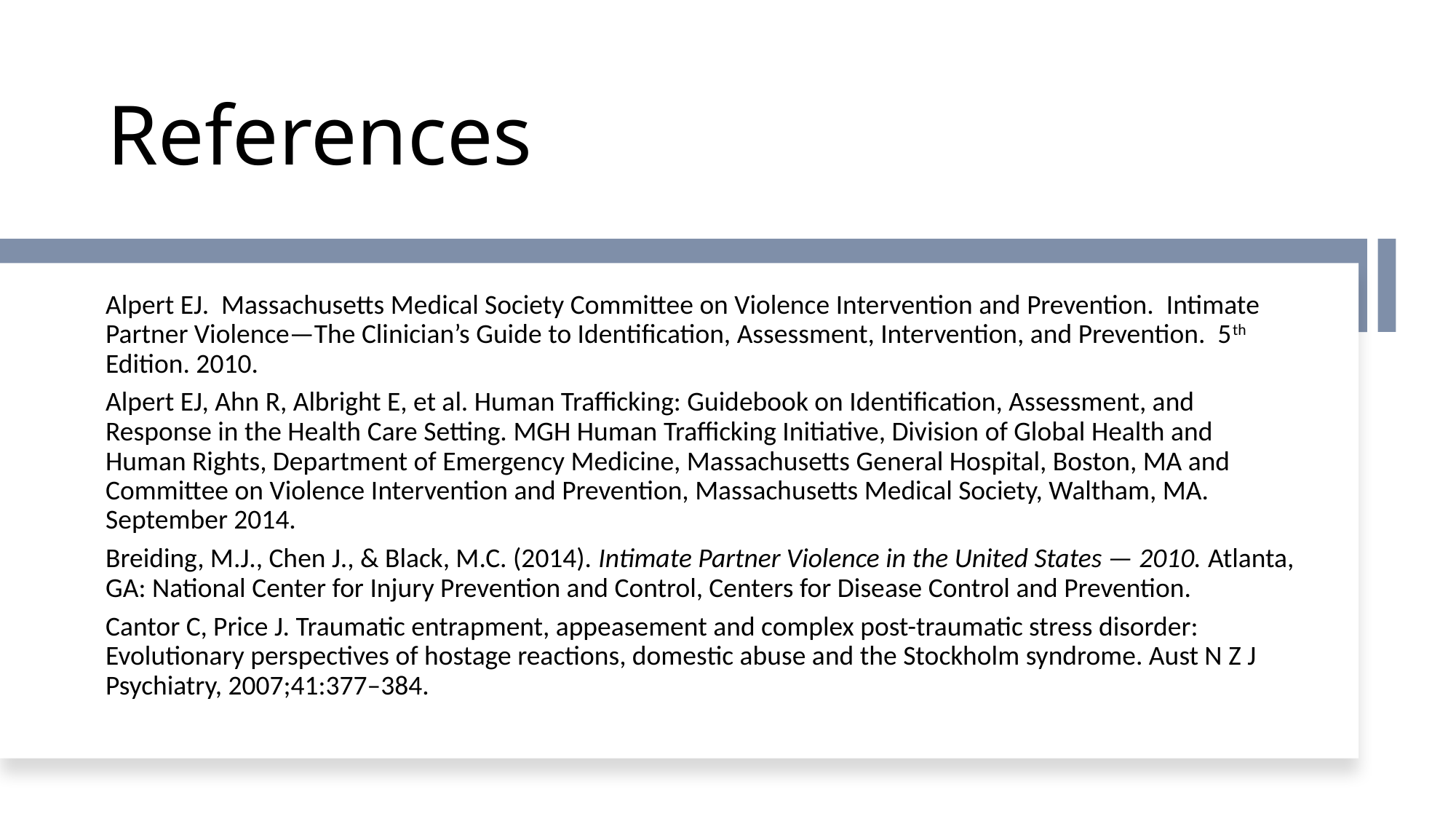

# References
Alpert EJ. Massachusetts Medical Society Committee on Violence Intervention and Prevention. Intimate Partner Violence—The Clinician’s Guide to Identification, Assessment, Intervention, and Prevention. 5th Edition. 2010.
Alpert EJ, Ahn R, Albright E, et al. Human Trafficking: Guidebook on Identification, Assessment, and Response in the Health Care Setting. MGH Human Trafficking Initiative, Division of Global Health and Human Rights, Department of Emergency Medicine, Massachusetts General Hospital, Boston, MA and Committee on Violence Intervention and Prevention, Massachusetts Medical Society, Waltham, MA. September 2014.
Breiding, M.J., Chen J., & Black, M.C. (2014). Intimate Partner Violence in the United States — 2010. Atlanta, GA: National Center for Injury Prevention and Control, Centers for Disease Control and Prevention.
Cantor C, Price J. Traumatic entrapment, appeasement and complex post-traumatic stress disorder: Evolutionary perspectives of hostage reactions, domestic abuse and the Stockholm syndrome. Aust N Z J Psychiatry, 2007;41:377–384.

## Slide 128
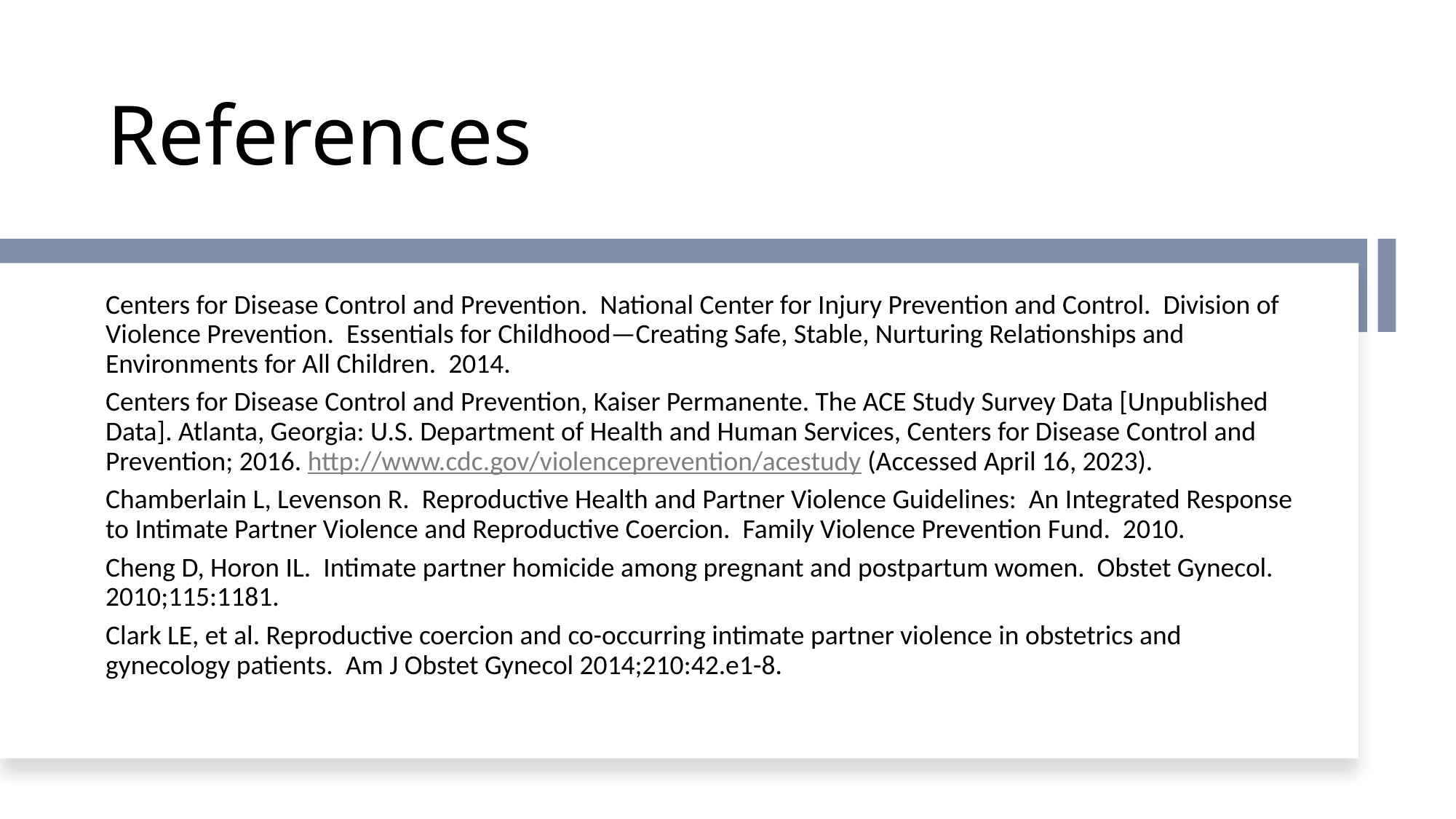

# References
Centers for Disease Control and Prevention. National Center for Injury Prevention and Control. Division of Violence Prevention. Essentials for Childhood—Creating Safe, Stable, Nurturing Relationships and Environments for All Children. 2014.
Centers for Disease Control and Prevention, Kaiser Permanente. The ACE Study Survey Data [Unpublished Data]. Atlanta, Georgia: U.S. Department of Health and Human Services, Centers for Disease Control and Prevention; 2016. http://www.cdc.gov/violenceprevention/acestudy (Accessed April 16, 2023).
Chamberlain L, Levenson R. Reproductive Health and Partner Violence Guidelines: An Integrated Response to Intimate Partner Violence and Reproductive Coercion. Family Violence Prevention Fund. 2010.
Cheng D, Horon IL. Intimate partner homicide among pregnant and postpartum women. Obstet Gynecol. 2010;115:1181.
Clark LE, et al. Reproductive coercion and co-occurring intimate partner violence in obstetrics and gynecology patients. Am J Obstet Gynecol 2014;210:42.e1-8.

## Slide 129
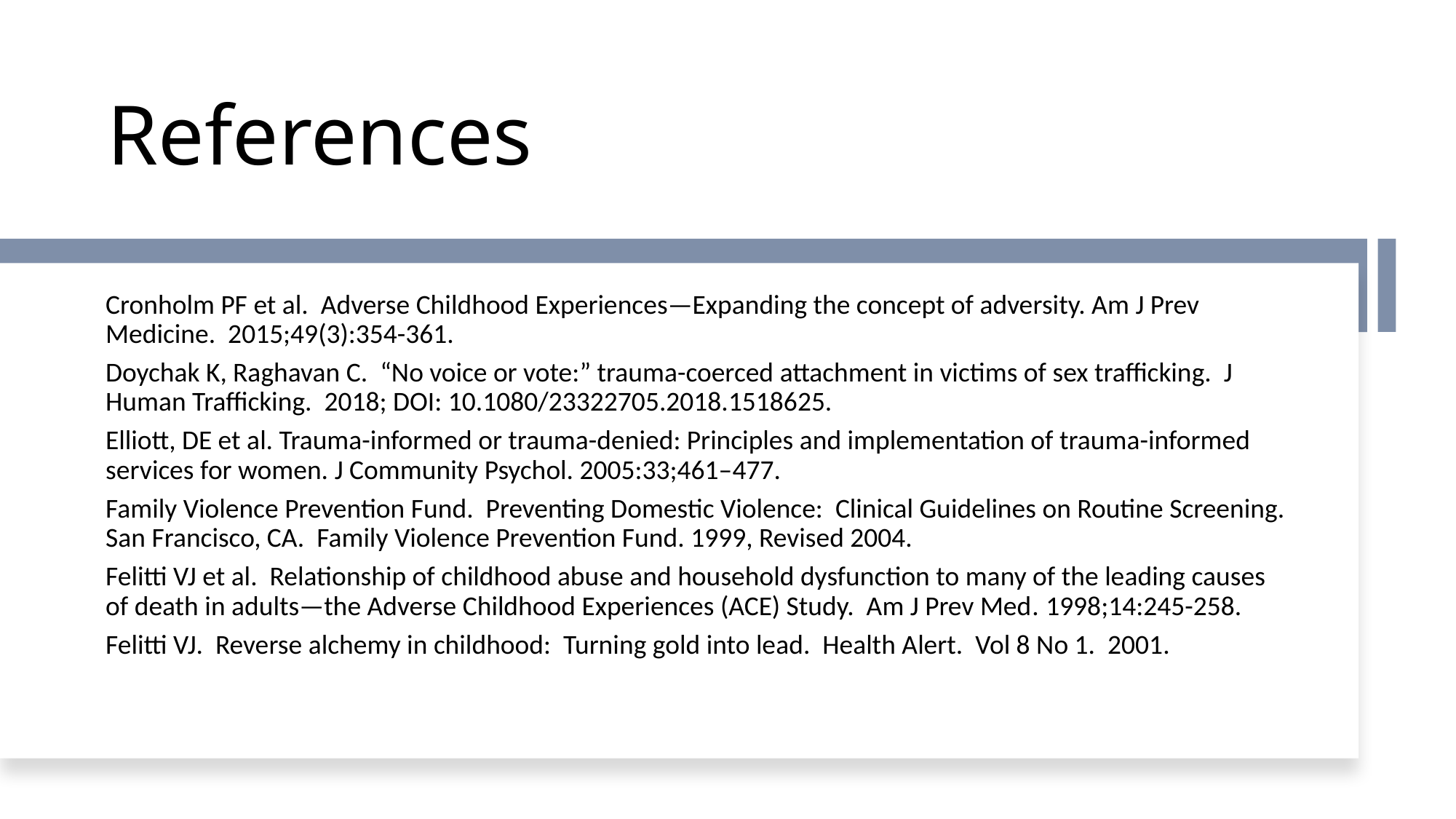

# References
Cronholm PF et al. Adverse Childhood Experiences—Expanding the concept of adversity. Am J Prev Medicine. 2015;49(3):354-361.
Doychak K, Raghavan C. “No voice or vote:” trauma-coerced attachment in victims of sex trafficking. J Human Trafficking. 2018; DOI: 10.1080/23322705.2018.1518625.
Elliott, DE et al. Trauma-informed or trauma-denied: Principles and implementation of trauma-informed services for women. J Community Psychol. 2005:33;461–477.
Family Violence Prevention Fund. Preventing Domestic Violence: Clinical Guidelines on Routine Screening. San Francisco, CA. Family Violence Prevention Fund. 1999, Revised 2004.
Felitti VJ et al. Relationship of childhood abuse and household dysfunction to many of the leading causes of death in adults—the Adverse Childhood Experiences (ACE) Study. Am J Prev Med. 1998;14:245-258.
Felitti VJ. Reverse alchemy in childhood: Turning gold into lead. Health Alert. Vol 8 No 1. 2001.

## Slide 130
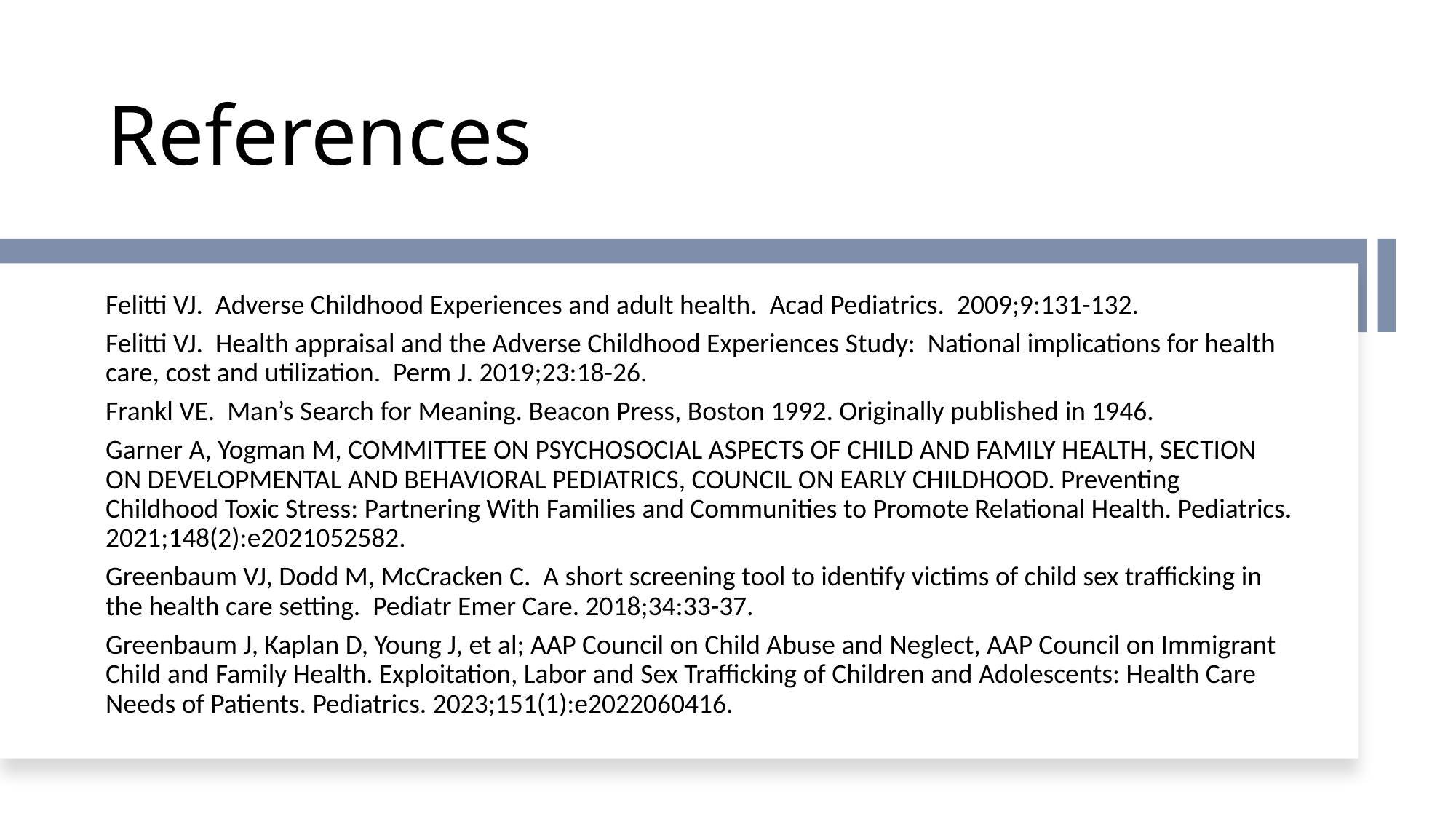

# References
Felitti VJ. Adverse Childhood Experiences and adult health. Acad Pediatrics. 2009;9:131-132.
Felitti VJ. Health appraisal and the Adverse Childhood Experiences Study: National implications for health care, cost and utilization. Perm J. 2019;23:18-26.
Frankl VE. Man’s Search for Meaning. Beacon Press, Boston 1992. Originally published in 1946.
Garner A, Yogman M, COMMITTEE ON PSYCHOSOCIAL ASPECTS OF CHILD AND FAMILY HEALTH, SECTION ON DEVELOPMENTAL AND BEHAVIORAL PEDIATRICS, COUNCIL ON EARLY CHILDHOOD. Preventing Childhood Toxic Stress: Partnering With Families and Communities to Promote Relational Health. Pediatrics. 2021;148(2):e2021052582.
Greenbaum VJ, Dodd M, McCracken C. A short screening tool to identify victims of child sex trafficking in the health care setting. Pediatr Emer Care. 2018;34:33-37.
Greenbaum J, Kaplan D, Young J, et al; AAP Council on Child Abuse and Neglect, AAP Council on Immigrant Child and Family Health. Exploitation, Labor and Sex Trafficking of Children and Adolescents: Health Care Needs of Patients. Pediatrics. 2023;151(1):e2022060416.

## Slide 131
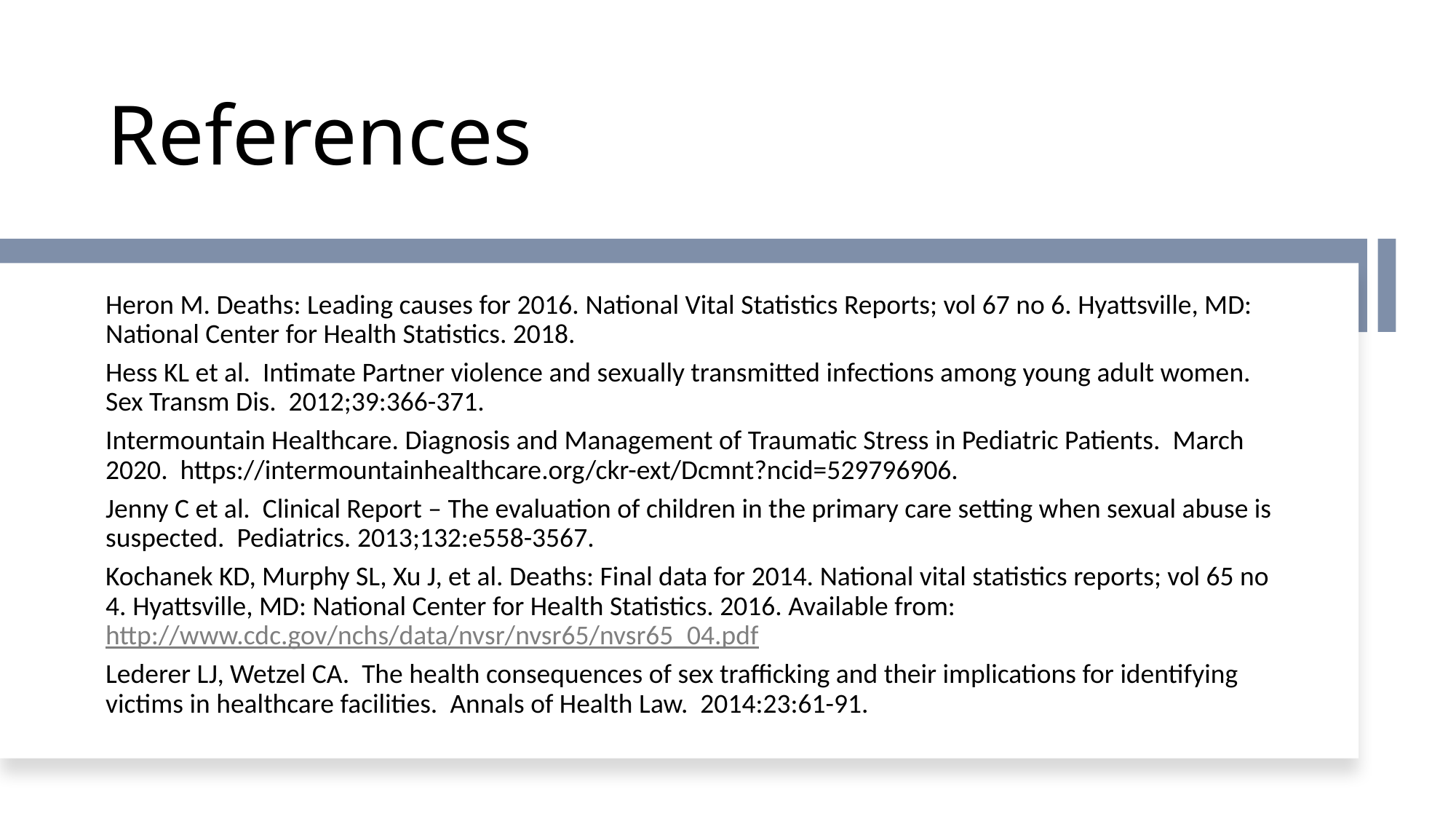

# References
Heron M. Deaths: Leading causes for 2016. National Vital Statistics Reports; vol 67 no 6. Hyattsville, MD: National Center for Health Statistics. 2018.
Hess KL et al. Intimate Partner violence and sexually transmitted infections among young adult women. Sex Transm Dis. 2012;39:366-371.
Intermountain Healthcare. Diagnosis and Management of Traumatic Stress in Pediatric Patients. March 2020. https://intermountainhealthcare.org/ckr-ext/Dcmnt?ncid=529796906.
Jenny C et al. Clinical Report – The evaluation of children in the primary care setting when sexual abuse is suspected. Pediatrics. 2013;132:e558-3567.
Kochanek KD, Murphy SL, Xu J, et al. Deaths: Final data for 2014. National vital statistics reports; vol 65 no 4. Hyattsville, MD: National Center for Health Statistics. 2016. Available from: http://www.cdc.gov/nchs/data/nvsr/nvsr65/nvsr65_04.pdf
Lederer LJ, Wetzel CA. The health consequences of sex trafficking and their implications for identifying victims in healthcare facilities. Annals of Health Law. 2014:23:61-91.

## Slide 132
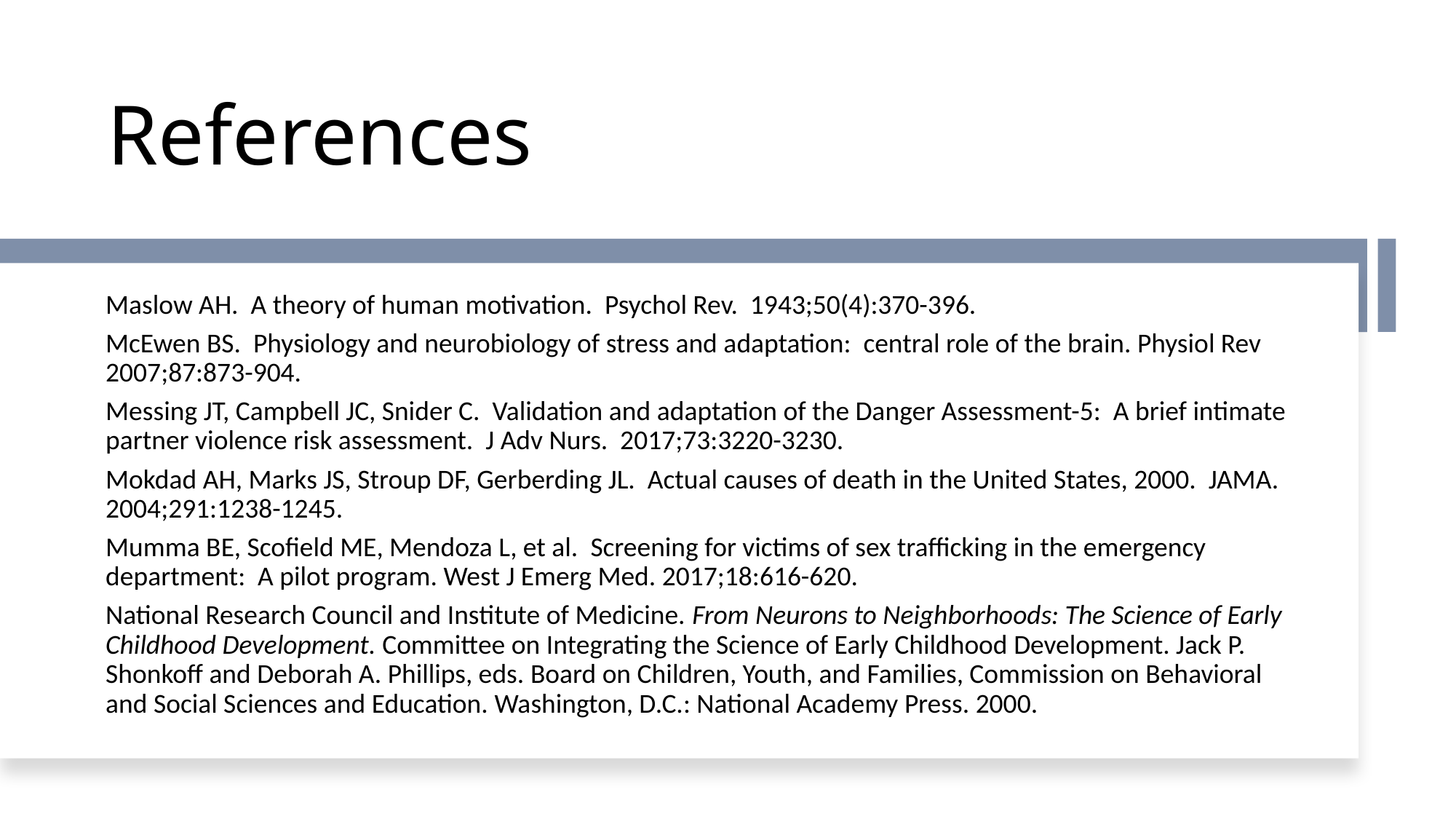

# References
Maslow AH. A theory of human motivation. Psychol Rev. 1943;50(4):370-396.
McEwen BS. Physiology and neurobiology of stress and adaptation: central role of the brain. Physiol Rev 2007;87:873-904.
Messing JT, Campbell JC, Snider C. Validation and adaptation of the Danger Assessment-5: A brief intimate partner violence risk assessment. J Adv Nurs. 2017;73:3220-3230.
Mokdad AH, Marks JS, Stroup DF, Gerberding JL. Actual causes of death in the United States, 2000. JAMA. 2004;291:1238-1245.
Mumma BE, Scofield ME, Mendoza L, et al. Screening for victims of sex trafficking in the emergency department: A pilot program. West J Emerg Med. 2017;18:616-620.
National Research Council and Institute of Medicine. From Neurons to Neighborhoods: The Science of Early Childhood Development. Committee on Integrating the Science of Early Childhood Development. Jack P. Shonkoff and Deborah A. Phillips, eds. Board on Children, Youth, and Families, Commission on Behavioral and Social Sciences and Education. Washington, D.C.: National Academy Press. 2000.

## Slide 133
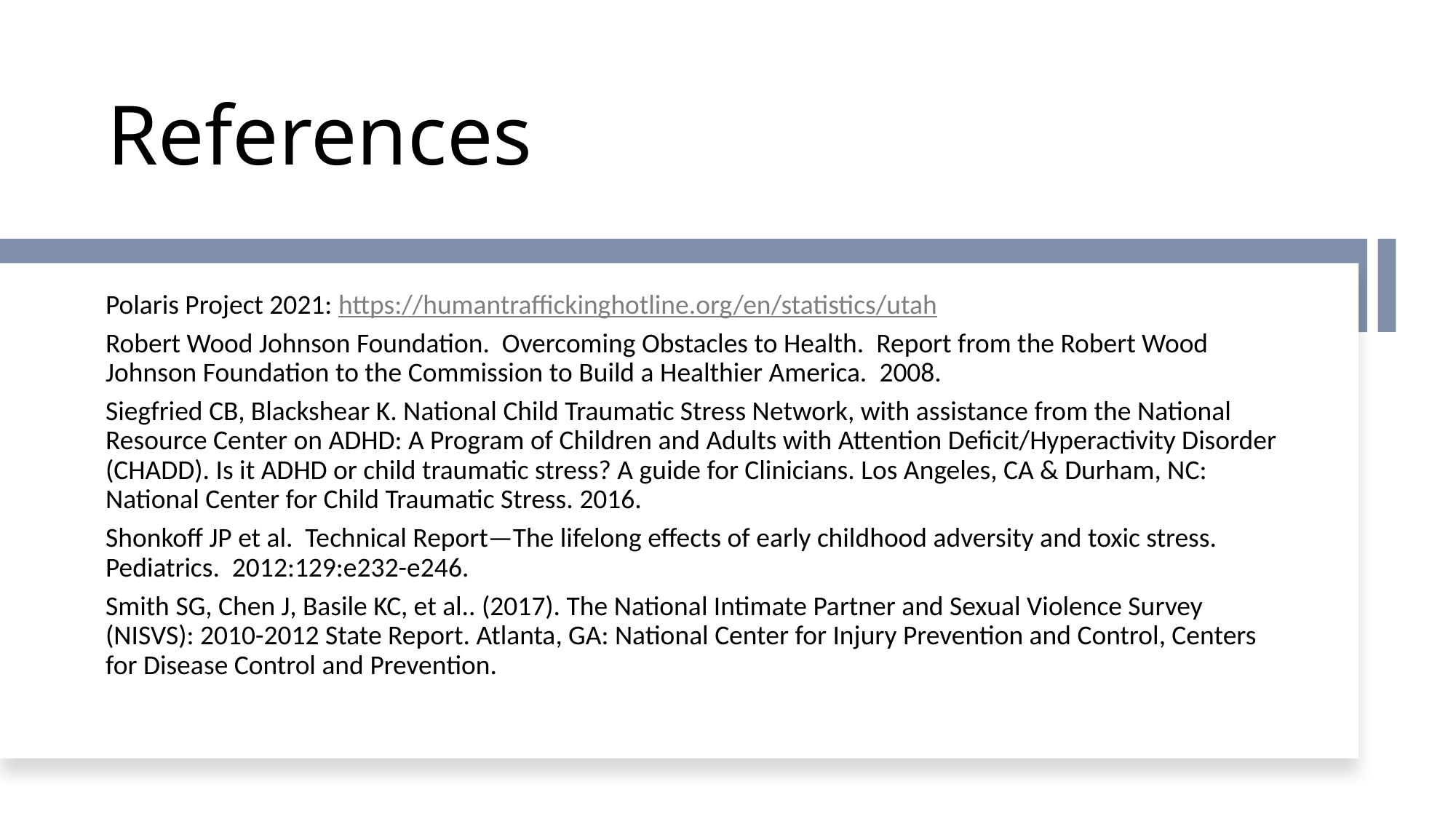

# References
Polaris Project 2021: https://humantraffickinghotline.org/en/statistics/utah
Robert Wood Johnson Foundation. Overcoming Obstacles to Health. Report from the Robert Wood Johnson Foundation to the Commission to Build a Healthier America. 2008.
Siegfried CB, Blackshear K. National Child Traumatic Stress Network, with assistance from the National Resource Center on ADHD: A Program of Children and Adults with Attention Deficit/Hyperactivity Disorder (CHADD). Is it ADHD or child traumatic stress? A guide for Clinicians. Los Angeles, CA & Durham, NC: National Center for Child Traumatic Stress. 2016.
Shonkoff JP et al. Technical Report—The lifelong effects of early childhood adversity and toxic stress. Pediatrics. 2012:129:e232-e246.
Smith SG, Chen J, Basile KC, et al.. (2017). The National Intimate Partner and Sexual Violence Survey (NISVS): 2010-2012 State Report. Atlanta, GA: National Center for Injury Prevention and Control, Centers for Disease Control and Prevention.

## Slide 134
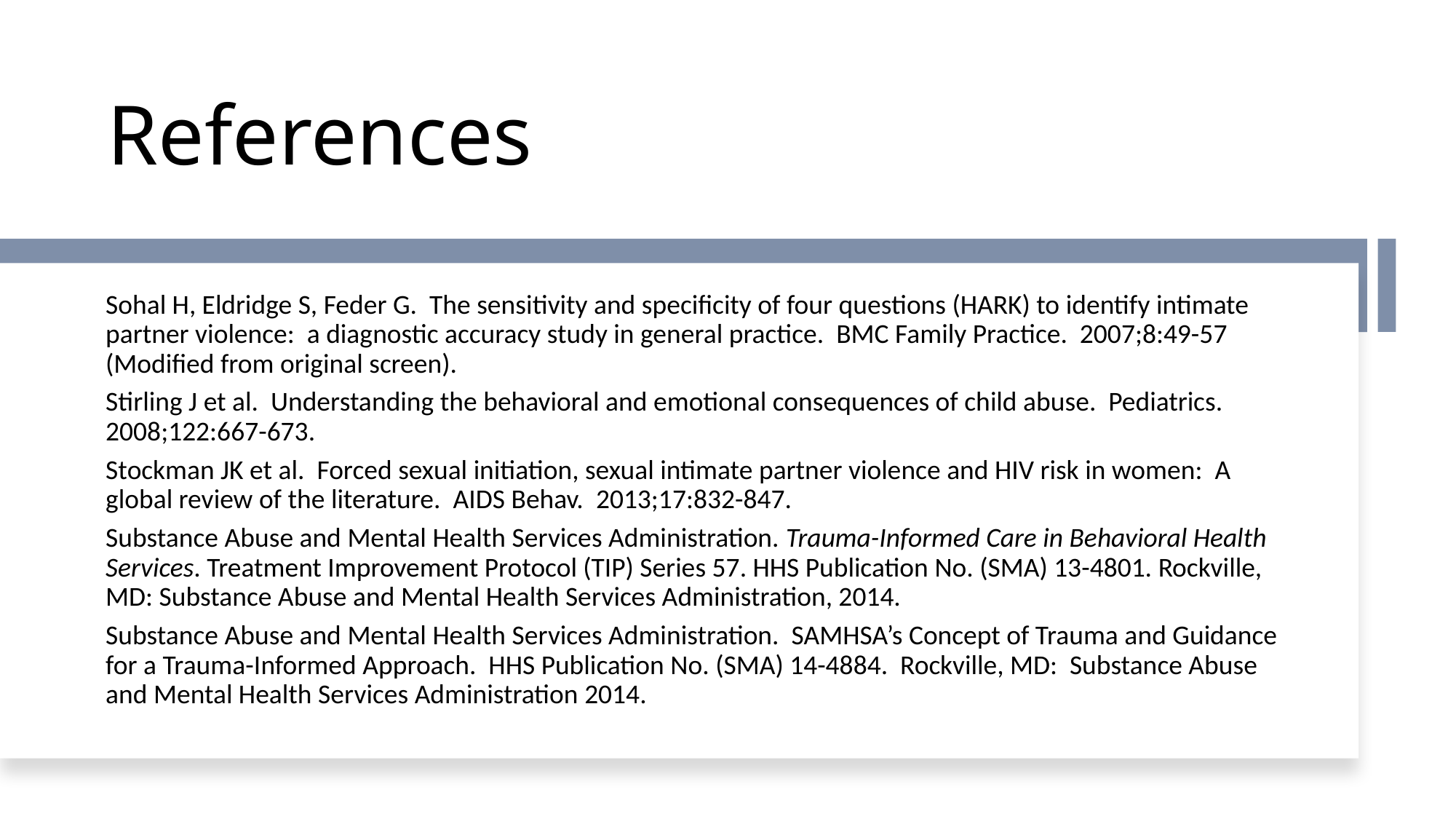

# References
Sohal H, Eldridge S, Feder G. The sensitivity and specificity of four questions (HARK) to identify intimate partner violence: a diagnostic accuracy study in general practice. BMC Family Practice. 2007;8:49-57 (Modified from original screen).
Stirling J et al. Understanding the behavioral and emotional consequences of child abuse. Pediatrics. 2008;122:667-673.
Stockman JK et al. Forced sexual initiation, sexual intimate partner violence and HIV risk in women: A global review of the literature. AIDS Behav. 2013;17:832-847.
Substance Abuse and Mental Health Services Administration. Trauma-Informed Care in Behavioral Health Services. Treatment Improvement Protocol (TIP) Series 57. HHS Publication No. (SMA) 13-4801. Rockville, MD: Substance Abuse and Mental Health Services Administration, 2014.
Substance Abuse and Mental Health Services Administration. SAMHSA’s Concept of Trauma and Guidance for a Trauma-Informed Approach. HHS Publication No. (SMA) 14-4884. Rockville, MD: Substance Abuse and Mental Health Services Administration 2014.

## Slide 135
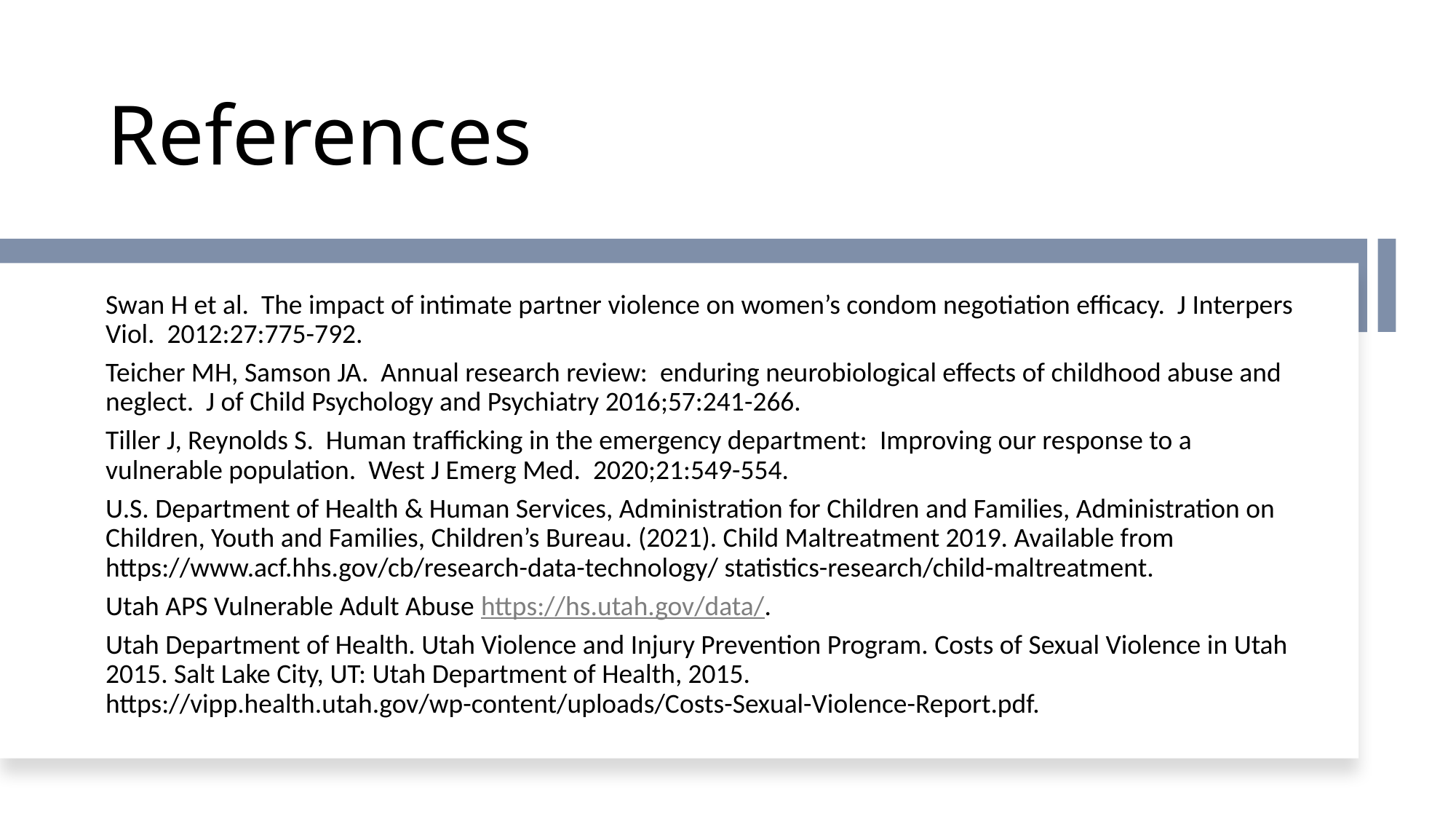

# References
Swan H et al. The impact of intimate partner violence on women’s condom negotiation efficacy. J Interpers Viol. 2012:27:775-792.
Teicher MH, Samson JA. Annual research review: enduring neurobiological effects of childhood abuse and neglect. J of Child Psychology and Psychiatry 2016;57:241-266.
Tiller J, Reynolds S. Human trafficking in the emergency department: Improving our response to a vulnerable population. West J Emerg Med. 2020;21:549-554.
U.S. Department of Health & Human Services, Administration for Children and Families, Administration on Children, Youth and Families, Children’s Bureau. (2021). Child Maltreatment 2019. Available from https://www.acf.hhs.gov/cb/research-data-technology/ statistics-research/child-maltreatment.
Utah APS Vulnerable Adult Abuse https://hs.utah.gov/data/.
Utah Department of Health. Utah Violence and Injury Prevention Program. Costs of Sexual Violence in Utah 2015. Salt Lake City, UT: Utah Department of Health, 2015. https://vipp.health.utah.gov/wp-content/uploads/Costs-Sexual-Violence-Report.pdf.

## Slide 136
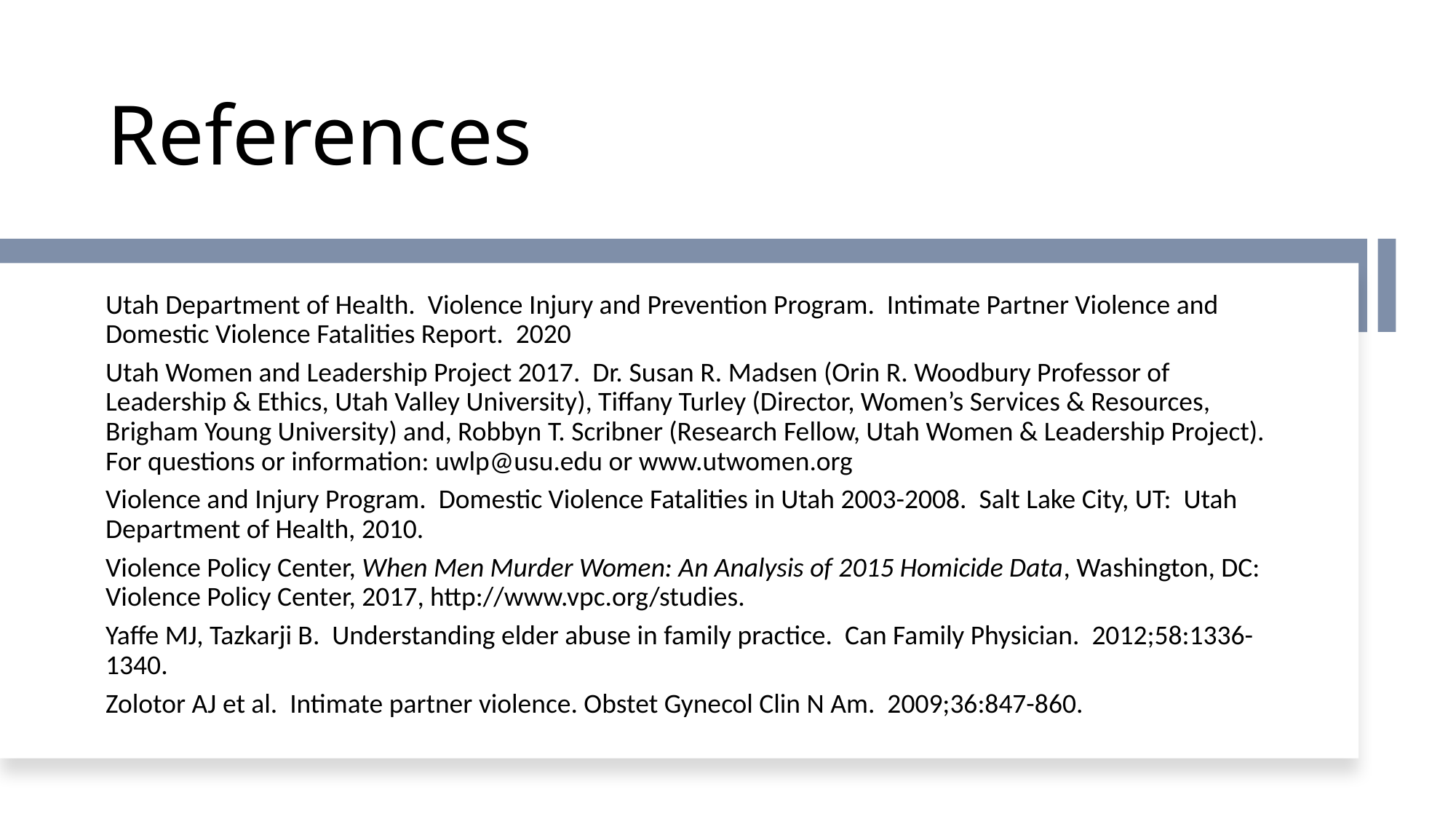

# References
Utah Department of Health. Violence Injury and Prevention Program. Intimate Partner Violence and Domestic Violence Fatalities Report. 2020
Utah Women and Leadership Project 2017. Dr. Susan R. Madsen (Orin R. Woodbury Professor of Leadership & Ethics, Utah Valley University), Tiffany Turley (Director, Women’s Services & Resources, Brigham Young University) and, Robbyn T. Scribner (Research Fellow, Utah Women & Leadership Project). For questions or information: uwlp@usu.edu or www.utwomen.org
Violence and Injury Program. Domestic Violence Fatalities in Utah 2003-2008. Salt Lake City, UT: Utah Department of Health, 2010.
Violence Policy Center, When Men Murder Women: An Analysis of 2015 Homicide Data, Washington, DC: Violence Policy Center, 2017, http://www.vpc.org/studies.
Yaffe MJ, Tazkarji B. Understanding elder abuse in family practice. Can Family Physician. 2012;58:1336-1340.
Zolotor AJ et al. Intimate partner violence. Obstet Gynecol Clin N Am. 2009;36:847-860.
