# Supplementary material for: Patient Abuse, Neglect, and Exploitation: Why Physicians Need to Be Trauma-Informed
Source: MedEdPORTAL. 2024 Apr 23;20:11391. doi: 10.15766/mep_2374-8265.11391 (PMC11035495; doi:10.15766/mep_2374-8265.11391)
Supplement: Supplementary file 1 — Prework Articles.docxDidactic.pptxRole-Playing Facilitator Guide.docxSMART Tool.docxPretest-Posttest Survey.docxPostsession Materials.docx [file mep_2374-8265.11391-s001.zip › F. Postsession Materials.docx]

Appendix F Post-Session Information

***Note to Facilitator****: This information is sent to the residents AFTER the educational session to provide the link to the evaluation survey, a copy of the PowerPoint didactic portion of the session, additional articles to read, an example of a General Resource List, and a summary of the electronic medical record safeguards for documenting trauma in a patient’s chart.*

1. A link to the Evaluation Survey (Appendix E)
2. Copy of the PowerPoint used in the Didactic Portion of the Educational Intervention (Appendix B)
3. Access to the original ACE Study: Felitti VJ, Anda RF, Nordenberg D, Williamson DF, Spitz AM, Edwards V, Koss MP, Marks JS. Relationship of childhood abuse and household dysfunction to many of the leading causes of death in adults: The Adverse Childhood Experiences (ACE) Study. Am J Prev Med. 1998;14(4):245-258.
4. Access to a 2019 article by VJ Felitti on the origins of the ACE Study: Felitti VJ. Origins of the ACE Study. Am J Prev Med. 2019;56(6):787-789.
5. A copy of a 2001 article published in Health Alert, in which VJ Felitti summarizes the findings of the ACE Study: Felitti VJ. Reverse Alchemy in Childhood.: Turning Gold into Lead. Health Alert. Family Violence Prevention Fund. 2001;8(1)1-4. [reserve_alchemy.pdf (futureswithoutviolence.org)](https://www.futureswithoutviolence.org/userfiles/file/HealthCare/reserve_alchemy.pdf)
6. A copy of the General Resource List used in our institution.
7. Summary of our EMR safeguards for documenting about trauma in the patient record (See below)

EMR Safeguards for Documentation

Below is a list of potential areas that we identified within our system where access to a patient’s chart by their perpetrator could theoretically happen.

**Paperwork given to patients after clinic visit or hospital discharge**

In Epic, this is called the After Visit Summary (AVS) and includes information such as the Vital Signs, Growth Charts, Diagnoses, future appointments, etc. Therefore, be mindful of the information that is given to the patient in terms of diagnoses and problems that may show up on the After Visit Summary and in the Patient Portal.

**Chart Access**

The creator of the Progress Note can block the note from being accessed through the Patient Portal. However, with the enactment of the 21^st^ Century Cures Act, Epic only allows certain reasons for preventing a note from going into the Patient Portal.

If preventing a note from being shared with a patient meets the specified options, know that this does not block the note from being released when a request for medical records is made.

**Billing statements**

As of now, the bill from our healthcare system does not list diagnoses.

Epic has an option called the ‘Confidential Guarantor.’ This allows the patient to have their bill sent somewhere other than their permanent address. An example would be a victim of IPV may want to keep the bill unknown to her partner, so the patient can give the address of a trusted friend, etc. This is additional

work and knowledge about Epic that most healthcare providers do not have so

reach out to the DV Committee if you have questions. However, this information

can be included in training your physicians and staff.

**Proxy Limits**

Epic provides a way for providers to stipulate proxy limits which can prevent the assigned proxy from seeing certain aspects of the medical record. Patients may give proxy access to their partners, so this is a safeguard in enhancing patient safety.

**Request for Medical Records**

Our Health Information Management (HIM) group and the University of Utah Health Domestic Violence Committee developed a process that involves sending a message to HIM to notify them that a particular progress note is not to be released with the routine request for medical records.

The protocol involves the provider sending a message to HIM, through the EMR (Electronic Medical Record), which is labeled IPV/DV (but can be used for any of the forms of abuse/assault/neglect). The provider documents the medical record number, the date of the progress note, and their name. This flags the note for the HIM staff to contact the provider when routine request of the medical record is made.
