# Supplementary material for: Patient Abuse, Neglect, and Exploitation: Why Physicians Need to Be Trauma-Informed
Source: MedEdPORTAL. 2024 Apr 23;20:11391. doi: 10.15766/mep_2374-8265.11391 (PMC11035495; doi:10.15766/mep_2374-8265.11391)
Supplement: Supplementary file 1 — Prework Articles.docxDidactic.pptxRole-Playing Facilitator Guide.docxSMART Tool.docxPretest-Posttest Survey.docxPostsession Materials.docx [file mep_2374-8265.11391-s001.zip › _Educational Summary Report_11391.pdf]

MedEdPORTAL® | The AAMC Journal of Teaching and Learning Resources

Original Publication | Open Access

Patient Abuse, Neglect, and Exploitation: Why Physicians Need to Be Trauma-Informed

Kathleen Franchek-Roa, MD*, Aarti Vala, MD, Jennifer Goldman, MD, MRP, Adam Dell, MD, MBA, Angela P. Presson, PhD, Kaleb Eppich, MS, Wendy L. Hobson, MD, MSPH

*Corresponding author: Kathy.Franchek@hsc.utah.edu

Abstract

Introduction: Many people experience trauma, and its cumulative effects throughout the life span can alter health, development, and well-being. Despite this, few publications focusing on interpersonal trauma include a holistic understanding of the nature and widespread exposure of trauma experiences for patients. We developed an educational resource to teach residents about identifying and intervening with patients who experience trauma across the life span using a trauma-informed care (TIC) perspective. **Methods:** We created a 4-hour educational session for residents that included didactics, a virtual visit with a domestic violence shelter, a discussion with a person who had experienced trauma, and role-playing. A pretest/posttest retrospective survey assessed resident confidence level in identifying and intervening with patients who may have experienced trauma. We used the Wilcoxon signed rank test to compare pretest and posttest scores and the Kruskal-Wallis test to compare responses by residency type and year. Free-text questions were analyzed for thematic content. **Results:** During the 2021-2022 academic year, 72 of 90 residents (80%) from four residency programs attended and evaluated the session. More than 90% of respondents reported the session met their educational needs and provided them with new ideas, information, and practical suggestions to use in their clinical endeavors. The results demonstrated significantly increased confidence on most of the metrics measured. **Discussion:** This session significantly improved residents' confidence in identifying and intervening with patients who have had trauma experiences using a TIC perspective, which may lead them to provide improved patient care to those who have experienced trauma.

Keywords
Human Trafficking, Sexual Violence, Trauma-Informed Care, Vulnerable Adult Abuse, Child Abuse, Elder Abuse, Intimate Partner Violence, Social Determinants of Health, Diversity, Equity, Inclusion

Educational Objectives

By the end of the session, learners will be able to:

1. Explain the link between childhood adversity and risk for poor health across the life span.
2. Utilize a trauma-informed approach when interacting with patients to reduce or mitigate the consequences of these adverse experiences.
3. Discuss the prevalence of abuse, neglect, and/or exploitation in terms of the public health impact on health care.
4. Employ best practices when evaluating patients who are victims of abuse, neglect, and/or exploitation.

Citation:
Franchek-Roa K, Vala A, Goldman J, et al. Patient abuse, neglect, and exploitation: why physicians need to be trauma-informed. *MedEdPORTAL*. 2024;20:11391.
https://doi.org/10.15766/mep_2374-8265.11391

Introduction

Trauma exposures are a common experience for many people. The Substance Abuse and Mental Health Services Administration (SAMHSA) defines individual trauma as

> an event, series of events, or set of circumstances that is experienced by an individual as physically or emotionally harmful or life threatening and that has lasting adverse effects on the individual's functioning and mental, physical, social, emotional, or spiritual well-being.[1]

The key to understanding a patient's trauma experiences and providing appropriate support and interventions is embedded in SAMHSA's definition of trauma in that the experience and effect of trauma are unique to each individual.

Copyright © 2024 Franchek-Roa et al. This is an open-access publication distributed under the terms of the Creative Commons Attribution license.

The cumulative effects of traumatic experiences throughout the life span can alter health, development, and well-being,[2,3] yet medicine has been slow to embrace the concept of trauma as a medical issue. Over the past 6 decades,[4-10] the medical community has pieced together an understanding of the nature and widespread occurrence of trauma experiences for patients. However, the different forms of family and interpersonal trauma have been treated independently in terms of research, education, and policy development.[11] This narrow focus has overlooked the interconnectedness of different forms of trauma, and as a result, the medical community has failed to understand the cumulative and profound negative impacts of trauma on lifelong health and the increased risk for future revictimization for those who experience polyvictimization in childhood.[2,12] The 1998 Adverse Childhood Experiences Study[2] and subsequent studies[13] have added to our understanding of the impact of adversity during childhood on lifelong health outcomes. Given the pervasive effects of trauma, the trauma-informed care (TIC)[1] approach has become an essential component in trauma curricula for health care providers.

Since research has shown that (1) trauma is common, (2) individual trauma experience often involves multiple types of victimization, and (3) trauma can adversely impact an individual's health and well-being across their life span, we developed an educational session at the University of Utah to educate residents in four residency programs (internal medicine, medicine-pediatrics, pediatrics, and family medicine) on the identification and intervention of patient abuse, neglect, and exploitation using a trauma-informed perspective. These residency programs acknowledged a need to integrate this comprehensive education into the training of their residents. Of the most relevant *MedEdPORTAL* publications addressing interpersonal trauma, only three[14-16] include multiple forms of trauma. However, these publications do not feature all the components that our educational session incorporates, specifically, a trauma-informed component. In addition, unlike this resource, none model their educational sessions on an institutional policy that has helped to provide an integrative component to the education.

Our educational model is unique in that it integrates a more comprehensive understanding of trauma; is modeled on the University of Utah Health—Policy on Abuse, Neglect, and Exploitation, which is actively used as guidance for physicians and staff within our health care system; and involves community partners who aid in educating the residents.

Methods

We developed a 4-hour educational session, held during the 2021-2022 academic year, for residents in four residency programs (internal medicine, medicine-pediatrics, pediatrics, and family medicine). Due to COVID-19 pandemic restrictions, the sessions were held in person on campus, without a visit to the domestic violence shelter.

The residency programs scheduled residents to attend during an outpatient or advocacy rotation, with a group size of two to eight residents to facilitate role-playing and group discussion. Residents received no incentive to participate or to complete the evaluation. Once scheduled, residents received articles to review prior to meeting (Appendix A). The in-person session included the following elements:

- Didactic lecture (60 minutes)
- Break (15 minutes)
- Virtual tour of the local domestic violence shelter (15 minutes)
- Discussion with a survivor of trauma (45 minutes)
- Break (15 minutes)
- Role-playing session and closure (90 minutes)

Didactic Lecture

The session started with a 60-minute PowerPoint didactic component (Appendix B). Its first 32 slides covered Educational Objectives 1-3 and set the learners up for success in role-playing activities covering all four objectives. Slides 3-11 defined and discussed trauma, adverse childhood experiences, and other forms of toxic stressors and associated poor health across the life span. This discussion included the science of how extrinsic and intrinsic factors playing a role in brain development during childhood were important in laying the foundations for a child's cognitive, physical, and socioemotional developmental responses to stress.[17] To enhance understanding of these concepts, there was broad discussion regarding how advancements in the science of epigenetics, neurodevelopment, and developmental psychology provided plausible biological pathways between early experiences and future development and health.[18] Slides 12-18 emphasized the universal approach of treating all patients with a trauma-informed perspective. Examples illustrated the use of a TIC perspective and emphasized that the goal was not to obtain a disclosure but to provide trauma-informed medical care and resources. Slides 19-32 highlighted the public health impact of trauma experiences. Slides 34-125 were used for the role-playing exercise.

Copyright © 2024 Franchek-Roa et al. This is an open-access publication distributed under the terms of the Creative Commons Attribution license.

Use of Community Partners in the Education of Residents

We partnered with a local shelter and family justice center (FJC) to provide contact with service providers and advocates. The FJC director gave a 15-minute virtual tour of the local shelter and FJC services via Zoom. Next, residents had a 45-minute interactive discussion, via Zoom, with a survivor of trauma who spoke of their experience with trauma and their interaction with health care systems during and after their trauma experiences. This gave the residents an opportunity to utilize the survivor's expertise in discussing how health care could improve the response to patients who have experienced trauma. It also provided the residents with a patient perspective on health care, especially that of a patient who had experienced trauma.

Faculty and Survivor Advocate Preparation

We recommend that faculty who wish to use this educational session be trained in trauma-informed practices, familiarize themselves with student/resident support systems in their institution, be trained in responding to a disclosure of trauma from a student/resident, and have access to faculty who can provide mental health services if needed by the learners. Author Kathleen Franchek-Roa was the chair of the University of Utah Health Domestic Violence Committee, which had written the policy for health care providers regarding interventions for persons experiencing abuse, neglect, and exploitation. The residents were made aware of the policy and available resources. Importantly, the survivor advocates had experience educating medical providers and others about their experiences and volunteered to participate in this resident training.

Role-Playing Session

Role-playing has been shown to improve communication skills[19] and increase self-efficacy[20] of learners. Role-playing as an educational method is regarded as an effective form of simulation, due in part to the social[21] and experiential[22] context in which learning takes place. Additionally, when students role-play as patients, they may garner a more empathetic perspective towards patients' concerns.[21] Therefore, we added a role-play component to the session to enhance learning and skill development.

The role-playing session adhered to Kolb and Fry's experiential learning model[22] by (1) providing a concrete experience for the residents in practicing how to respond to a trauma disclosure, (2) involving the residents who were not role-playing as observers to enrich the discussion, and (3) creating generalizable concepts by dissecting the case into its component parts. The fourth component, testing implications in new situations, would occur subsequently as the residents applied the skills and knowledge gained in the session in direct patient care in the clinic or hospital setting.

We created seven role-playing scenarios: an intimate partner violence/abuse case, a child abuse case, pediatric and adult human trafficking cases, vulnerable adult abuse cases (both elder and nonelder), and a sexual assault case (Appendix C). Two to three role-playing scenarios were utilized during each 90-minute session. Scenarios were selected according to which residency programs were participating. The role-playing scripts had a definite beginning and end to simulate how these narratives could play out in a real case scenario and gave the residents a chance to use the SMART (screen vs. ask, message, assess symptoms and danger, resources and report, treat) tool (Appendix D) to help guide the role-play to its conclusion. The debriefing portion adhered to principles of debriefing,[23] including establishing group norms, giving participants and observers an opportunity to reflect on the scenario, applying knowledge previously learned (Appendix B) to the role-playing scenarios, and allowing participants to discuss the value of this experience either verbally during the discussion or by providing written anonymous comments via the evaluation survey after the session.

The role-playing was guided by Appendix B's PowerPoint slides 34-125, which provided our state-specific reporting requirements, the SMART tool (Appendix D), and the discussion of the cases. Authors Kathleen Franchek-Roa and Aarti Vala developed the SMART tool to help learners navigate these difficult patient encounters. We designed the SMART tool more as a retrieval aid than as a core-learning strategy.[24]

Relevant principles adhered to in providing a psychologically safe learning environment[25] and enhancing communication during the role-playing session[21] included providing prework articles and objectives prior to the educational session; offering the didactic portion of the session before role-playing to provide background knowledge; utilizing the SMART tool (Appendix D) to aid residents in navigating difficult conversations; promoting empathy towards patients and each other, which could lead to enhancing the humanistic response (e.g., by emphasizing the understanding that high-risk health behaviors might be a coping strategy for patients who have experienced trauma,[2] by having residents play the patient role,[21] and by incorporating the understanding of the holistic approach to medicine); encouraging the residents to be comfortable in their uncertainty[26] because many of these patient encounters might not lead to a satisfactory conclusion; and drawing on SAMHSA's trauma-informed principles[1] in creating a safe space for learners. These concepts helped to enhance group norms among the learners as they shared these

Copyright © 2024 Franchek-Roa et al. This is an open-access publication distributed under the terms of the Creative Commons Attribution license.

experiences. These educational concepts added an important dimension to what it meant to be a doctor.[25,27] Faculty were available for residents if further discussion was needed. In addition, including a small-group role-playing session aided in providing peer support for the residents. Although most of the learners were interns, numerous sessions included second- and third-year residents, who provided the interns with constructive suggestions informed by their own interactions with patients who had experienced trauma.

Postsession Materials
After the session, residents received a link to the electronic evaluation survey (Appendix E) and additional materials (Appendix F).

Evaluation
The evaluation survey (Appendix E) measured whether the session met residents' educational needs and provided them with new ideas/information and practical suggestions to use in their clinical endeavors (choices were yes, somewhat, and no). The retrospective pretest/posttest evaluated residents' confidence in their ability to meet the objectives (choices were no confidence, low confidence, moderate confidence, and high confidence). The retrospective pretest/posttest had learners rate their level of baseline ability (e.g., knowledge, attitudes, behaviors) simultaneously with their posteducational intervention ability. Howard and colleagues[28] undertook a rigorous evaluation of the traditional versus retrospective pretest/posttest design and found that the retrospective pretest/posttest design provided a measure of self-reported change more in line with objective changes observed. Our evaluation survey also included demographic questions and two free-text questions about behavioral change and ways to improve the session. The evaluation survey was implemented electronically and anonymously collected through REDCap, a self-service application subsidized for University of Utah research by the Clinical Translational Science Institute. Residents were free to choose whether to fill out the survey or not.

We summarized demographics and residency information using median and interquartile range for age as a continuous variable and counts and percentages for gender, residency type, and residency year. Likert plots were constructed for the survey questions both before and after the training using a retrospective pretest/posttest design. The pretest and posttest survey responses were summarized using percentages (in the Likert plots), means, and standard deviations and compared using Wilcoxon signed rank tests. The difference between the pretest and posttest survey responses was calculated for each survey question, and the Kruskal-Wallis test was used to compare the difference in responses by residency type and year. Year of residency was divided into two groups (first-year residents and second-/third-/fourth-year residents) to determine if year of residency, as a proxy for experience, impacted the change in resident confidence in the parameters measured. We also analyzed the free-text questions for thematic content. Statistical significance was assessed at the $p < .05$ level, and all tests were two-tailed. Statistical analyses were performed using R version 4.2.1 (R Foundation).

Results

Ninety residents out of 106 eligible participated in the educational sessions during the 2021-2022 academic year, and 72 of the 90 (80%) completed the evaluation. The median age was 27 years, 48% of the respondents were female, 49% were internal medicine residents, and most (68%) were in their first year of residency (Table 1).

The overwhelming majority of respondents (≥90%) felt that the session met their educational needs, provided them with new ideas and information, and gave them practical suggestions that they could use in their clinical endeavors.

Table 2 shows that scores improved significantly for all items ($p < .001$). Importantly, there was no difference in score improvement (posttest minus pretest) between residency type and residency year except for one item. Score improvement for "Employ best practices when evaluating patients who are victims of abuse, neglect, and/or exploitation" was significantly higher for first-year residents relative to the combined group of second-/third-/fourth-year residents ($p = .02$).

The Figure shows Likert plots for each question illustrating the differences between the retrospective pretest/posttest responses. For example, the statement "Rate your confidence in being able to identify and intervene with patients who are victims of abuse, neglect, and exploitation" shows that 31% rated their pretraining confidence as moderate or high as opposed to 100% rating their posttraining confidence as moderate or high. The remaining statements can be interpreted similarly.

The thematic content for the question "What changes will you incorporate into your future clinical endeavors as a result of the knowledge acquired during this activity?" is represented in Table 3. The most common themes included recognizing the signs and symptoms of potential abuse (42%), screening for intimate partner violence as per the US Preventive Services Task Force recommendation[29] (35%), and using the General

Copyright © 2024 Franchek-Roa et al. This is an open-access publication distributed under the terms of the Creative Commons Attribution license.

Table 1. Survey Demographics

Characteristic	*Mdn* (Interquartile Range)	No. (%) Respondents[a]	No. Missing	No. Trained[b]	Response Rate (%)
Age (in years)	27 (26, 29)		7		
Sex			6		
Female		32 (48)			
Male		33 (50)			
Would prefer not to answer		1 (2)			
Residency			0		
Family medicine		10 (14)		15	67
Internal medicine		35 (49)		42	83
Medicine/pediatrics		4 (6)		6	67
Pediatrics		23 (32)		27	85
Year of residency			0		
First		49 (68)			
Second		11 (15)			
Third		10 (14)			
Fourth		2 (3)			

[a]$N = 72$.
[b]$N = 90$.

Resource List (27%). The two most common themes in response to the question “How can we improve the session?” were “no improvement needed” (48%) and “more role-playing” (14%).

Discussion

As the significance of the impact of trauma experiences on health has weaved its way into the health care arena, there is a need to develop medical education that improves future health care professionals’ ability to address patients’ trauma experiences and their impact. Many trauma-related medical education publications focus on only one form of interpersonal trauma. Since many people experience multiple types of trauma across their life span, offering this education as a holistic session, as our project does, is crucial in providing appropriate and effective care to patients.

The finding that participants’ confidence was significantly improved after the educational session indicates that the session was effective in improving residents’ confidence in their ability to care for patients who have experienced trauma. In addition, finding that only one-third of participants felt confident in their ability to intervene with patients who have experienced trauma before the session while all (100%) felt confident in this ability after the session suggests that residents would benefit from structured educational sessions on how to care for patients who have experienced trauma.

We undertook an analysis that included comparing residency type and year with confidence improvement to determine if different residency programs and year in residency (as a proxy for experience) influenced the results. We found that the improvement in confidence in caring for patients who have experienced trauma did not differ between residency types, suggesting that this session can be taught to multiple groups of residents. Regarding year in residency, we found that the improvement in confidence for four of the five survey questions did not differ between the first-year group and

Table 2. Retrospective Pretest/Posttest Survey Responses and Average Difference for Residency Type and Year ($N = 72$)

Survey Question[a]	Pretest *M* (*SD*)	Posttest *M* (*SD*)	p[b]	Residency Type p[c]	Residency Year p[d]
Rate your confidence in being able to identify and intervene with patients who are victims of abuse, neglect, and exploitation.	2.3 (0.6)	3.4 (0.5)	<.001	.89	.13
Please rate your confidence in your ability to:					
Explain the link between childhood adversity and risk for poor health across the life span.	2.9 (0.8)	3.6 (0.5)	<.001	.41	.25
Utilize a trauma-informed approach when interacting with patients to reduce or mitigate the consequences of these adverse experiences.	2.4 (0.6)	3.4 (0.5)	<.001	.51	.27
Discuss the prevalence of abuse, neglect, and/or exploitation in terms of the public health impact to health care.	2.4 (0.7)	3.5 (0.6)	<.001	.63	.48
Employ best practices when evaluating patients who are victims of abuse, neglect, and/or exploitation.	2.3 (0.6)	3.4 (0.5)	<.001	.27	.02

[a]Rated on a 4-point scale (1 = *no confidence*, 2 = *low confidence*, 3 = *moderate confidence*, 4 = *high confidence*).
[b]Wilcoxon signed rank test with continuity correction.
[c]Kruskal-Wallis test comparing the average difference (posttest minus pretest) between residency type.
[d]Kruskal-Wallis test comparing the average difference (posttest minus pretest) between residency year (year 1 vs. years 2-4).

Copyright © 2024 Franchek-Roa et al. This is an open-access publication distributed under the terms of the Creative Commons Attribution license.

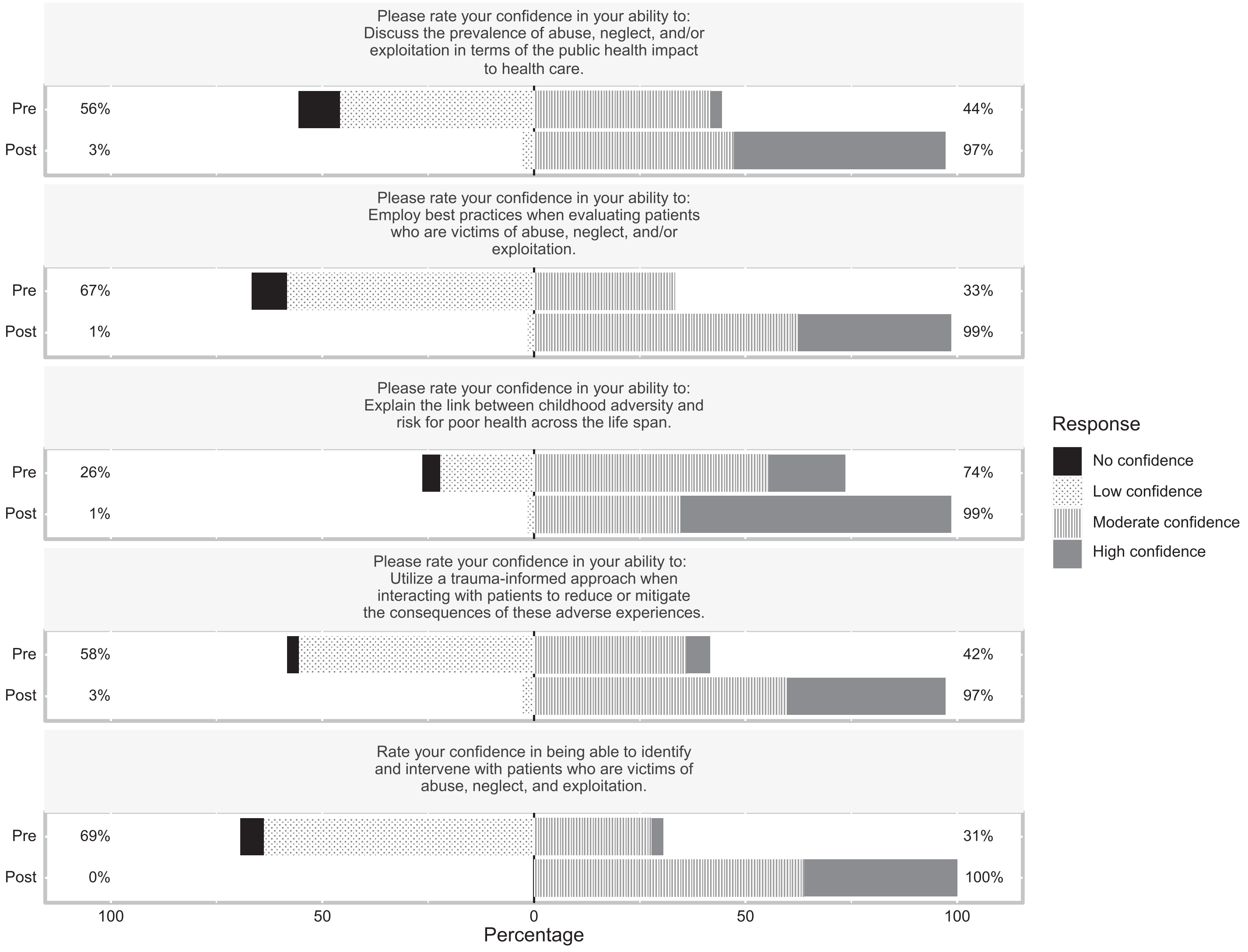

Figure. Likert plots for each survey question.

the combined second-/third-/fourth-year group. This may suggest that structured educational interventions, including skill acquisition through role-playing scenarios, are effective across all experience levels.

Our resource is unique because it provides a broadened approach to interpersonal trauma by incorporating trauma experiences across the life span, illustrating the interconnectedness of different trauma experiences for patients, including the TIC perspective, and being based on a policy at our institution. This comprehensive understanding of patients' life experiences aids in developing effective interventions. With intimate partner violence at least, developing an integrated systems-based approach has been shown to be crucial to providing the care and resources that patients exposed to this type of trauma need.[30] It is reasonable to extrapolate that integrating a systemwide approach would also benefit patients experiencing other forms of trauma.

This session included educational methods derived from adult learning principles,[22,31] such as making the content relevant to the work residents are engaged in, utilizing multiple educational techniques that support different learning styles, providing experiential opportunities to practice skills in a safe environment, offering faculty and peer feedback during the learning session, and providing content that learners can immediately use when they interact with patients (e.g., SMART tool). Skills taught can serve these residents in many inpatient and outpatient

Copyright © 2024 Franchek-Roa et al. This is an open-access publication distributed under the terms of the Creative Commons Attribution license.

Table 3. Thematic Content for the Reflective Question “What Changes Will You Incorporate Into Your Future Clinical Endeavors as a Result of the Knowledge Acquired During This Activity?”(N = 71; Single Responses Not Shown)

Thematic Content	No.[a]	Selected Comments
Recognizing signs and symptoms of potential abuse	30	“I will be looking out for the signs more often. Also listening to the patients and knowing which resources are available.”
Screening for intimate partner violence as per US Preventive Services Task Force recommendations	25	“Screening women of childbearing age for domestic violence.”<br>“I will feel more confident screening for trauma and abuse and feel better equipped to address it when it does come up. The speaker was also very impactful and will make me rethink my approach to medical interactions that may likely be influenced by past or current trauma.”
Using the General Resource List	19	“Learning how best to find resources for victims of abuse was very helpful, also good to know who we as providers can contact for help.”
Using the knowledge of the impact of adversity on health	15	“Integrating adverse events as something that may be contributing to a person’s physical symptoms.”<br>“I will connect adverse experiences better to current symptoms and hard to treat conditions.”<br>“Being aware that trauma can impact health outcomes beyond psych.”
Utilizing SMART	12	“I think the SMART method is a great and simple way to remember goal-oriented interaction.”
Using trauma-informed principles	11	“I will be more trauma-informed in my discussions with families.”
Improved knowledge of reporting requirements	8	“Was helpful to know the exact reporting guidelines.”
Asking about the social history	5	“I will take more time to intentionally ask patients about social situations or relationships that could be impacting their life/health.”
N/A	4	

Abbreviation: SMART, screen versus ask, message, assess symptoms and danger, resources and report, treat.
[a]Numbers do not add up to 71 because several participants gave multiple answers.

experiences. Although our session focused on interpersonal trauma, discussions after the role-playing scenarios expanded this concept of trauma to include other forms of stressors that patients and their families experience, such as social determinants of health, as well as trauma-informed principles that should be integrated into all patient encounters.

The weekly availability of sessions enabled us to engage with multiple residency programs. Allowing each of the residency programs to carve out a time that worked for its residents was key in being able to provide this education to a broad swath of primary care medical specialties within our medical school.

We have demonstrated the short-term effectiveness of this resource. The thematic content qualitatively showed that residents had begun to internalize what they had learned and were already envisioning ways to incorporate it into their practice. Over a quarter of the residents (27%) specifically mentioned the utility of a resource list when working with patients who have experienced trauma, and one-third (36%) indicated that they would be screening for intimate partner violence in women of reproductive age. This is a critical first step in providing care to patients.

Of the topics covered in this resource, the only screening recommendation from the US Preventive Services Task Force is for intimate partner violence in women of reproductive age.[29] The lack of recommendations for screening for violence is emphasized in our session (and our institutional policy) by recommending that health care providers screen for intimate partner violence in women of reproductive age but also ask any patient with signs or symptoms suggestive of trauma. This is an important distinction because asking about trauma experiences in patients who have symptoms suggestive of trauma can yield a more accurate diagnosis, thereby avoiding misdiagnoses that can lead to mismanagement of symptoms.[32,33] Recognizing trauma symptoms is critical in identifying patients who would benefit from evidence-based, trauma-specific treatments.[34,35]

Although our session was effective in increasing residents’ confidence in their ability to identify and intervene with patients who have experienced trauma, there are several limitations. We implemented the session at only one institution, with four residency programs. Results may not be the same with other types of learners or at other institutions. We documented short-term improvement in the indices measured, but we do not yet have data to show that the improvements in knowledge and behavior were sustained. Our evaluation relied on self-reported, not observed, change, although using the retrospective pretest/posttest evaluation led to a more accurate assessment of change.[28] In addition, it might be of value in the future to provide a multiple-choice survey of learners’ understanding of these concepts, which could delineate areas of misunderstanding and lack of knowledge and thus enhance the educational sessions. Using trauma-informed principles is critical to providing a safe learning environment, and utilizing near-peer facilitators who have previously participated in the training might provide additional benefit to learners, especially those in their intern year. We learned valuable lessons by allowing the residents to provide anonymous comments via the evaluation survey.[23] For example, resident comments included how to improve using the

Copyright © 2024 Franchek-Roa et al. This is an open-access publication distributed under the terms of the Creative Commons Attribution license.

expert's lived experience to maximize the learning benefit; how having the session at the shelter, once COVID restrictions are eased, might be more impactful; and how, since the role-playing enhanced learning, more time should be allotted to this portion of the educational session.

Due to COVID-19 restrictions, we had a virtual visit with the domestic violence shelter. We surmise that holding sessions at a domestic violence shelter could enhance residents' experience. Engaging community partners in the education of residents strengthens relationships between health care and community resources, which is vital in providing support to patients. The discussion with the experts enriched the session by providing a relationship, albeit brief, between the residents and a survivor, which is difficult to achieve if not in real time or in person. It helped to put a face to these difficult issues.

Implementing this educational model is labor intensive, requiring dedicated faculty to provide numerous sessions so as to involve residents from different residency programs and rotations. The sessions are difficult to run with larger groups of participants because having fewer participants per session allows for more experiential opportunities, especially regarding the role-playing. Finally, a 4-hour session can be tiresome for participants and difficult to fit into a busy residency schedule. The session could be broken into smaller sessions, by trauma or activity, while still preserving the holistic approach to patient trauma experiences.

Our educational model was successful, in the short term, in educating residents to respond to patients who have experienced trauma. More research is needed to inform effective prevention and intervention efforts for patients with trauma experiences and to provide guidance on how to implement this into medical education. Training the next generation of health care providers in understanding how to identify and support patients suffering the lifelong effects of trauma, with a TIC perspective, is the first step towards ensuring a healthier future for everyone and is a pressing need in medical education.

Appendices

A. Prework Articles.docx
B. Didactic.pptx
C. Role-Playing Facilitator Guide.docx
D. SMART Tool.docx
E. Pretest-Posttest Survey.docx
F. Postsession Materials.docx

All appendices are peer reviewed as integral parts of the Original Publication.

Kathleen Franchek-Roa, MD: Associate Professor, Department of Pediatrics, University of Utah School of Medicine; ORCID: https://orcid.org/0000-0002-7906-6373

Aarti Vala, MD: Lead Physician, Pediatrics, Mission Neighborhood Health Center, San Francisco, CA

Jennifer Goldman, MD, MRP: Associate Professor, Department of Pediatrics, University of Utah School of Medicine

Adam Dell, MD, MBA: Adjunct Associate Professor, Department of Pediatrics, University of Utah School of Medicine

Angela P. Presson, PhD: Research Professor, Division of Epidemiology, University of Utah School of Medicine

Kaleb Eppich, MS: Biostatistician, Department of Internal Medicine, University of Utah School of Medicine

Wendy L. Hobson, MD, MSPH: Professor, Department of Pediatrics, and Associate Vice President of Health Sciences Education, University of Utah School of Medicine

Disclosures
None to report.

Funding/Support
This investigation was supported by the University of Utah Study Design and Biostatistics Center, with funding in part from the National Center for Research Resources and the National Center for Advancing Translational Sciences, National Institutes of Health, through grant UM1TR004409 (formerly UL1TR002538).

Ethical Approval
The University of Utah Institutional Review Board deemed further review of this project not necessary.

References

1. Substance Abuse and Mental Health Services Administration. *SAMHSA's Concept of Trauma and Guidance for a Trauma-informed Approach.* Substance Abuse and Mental Health Services Administration; 2014. HHS publication (SMA) 14-4884. Accessed March 1, 2024. https://store.samhsa.gov/sites/default/files/sma14-4884.pdf
2. Felitti VJ, Anda RF, Nordenberg D, et al. Relationship of childhood abuse and household dysfunction to many of the leading causes of death in adults: the Adverse Childhood

Copyright © 2024 Franchek-Roa et al. This is an open-access publication distributed under the terms of the Creative Commons Attribution license.

Experiences (ACE) Study. *Am J Prev Med*. 1998;14(4):245-258. https://doi.org/10.1016/S0749-3797(98)00017-8

3. Putnam KT, Harris WW, Putnam FW. Synergistic childhood adversities and complex adult psychopathology. *J Trauma Stress*. 2013;26(4):435-442. https://doi.org/10.1002/jts.21833
4. American Hospital Association. *ICD-10-CM Coding for Human Trafficking*. American Hospital Association; 2018. Accessed March 1, 2024. https://aha.org/system/files/media/file/2019/04/AHA-Fact-Sheet-icd-10-code-human-trafficking.updated_1.pdf
5. Chisolm-Straker M, Stoklosa H, eds. *Human Trafficking Is a Public Health Issue: A Paradigm Expansion in the United States.* Springer Cham; 2017.
6. Dahlberg LL, Mercy JA. History of violence as a public health problem. *Virtual Mentor.* 2009;11(2):167-172. https://doi.org/10.1001/virtualmentor.2009.11.2.mhst1-0902
7. Heath JM, Dyer CB, Kerzner LJ, Mosqueda L, Murphy C. Four models of medical education about elder mistreatment. *Acad Med*. 2002;77(11):1101-1106. https://doi.org/10.1097/00001888-200211000-00007
8. Kempe CH, Silverman FN, Steele BF, Droegemueller W, Silver HK. The battered-child syndrome. *JAMA*. 1962;181(1):17-24. https://doi.org/10.1001/jama.1962.03050270019004
9. Tjaden P, Thoennes N. *Prevalence, Incidence, and Consequences of Violence Against Women: Findings From the National Violence Against Women Survey.* National Institute of Justice; 1998. NJC 172837.
10. *Healthy People: The Surgeon General's Report on Health Promotion and Disease Prevention.* US Department of Health, Education, and Welfare; 1979. DHEW (PS) publication 79-55071.
11. Renner LM. The co-occurrence of child maltreatment and intimate partner violence: a commentary on the special issue. *Child Maltreat.* 2021;26(4):464-469. https://doi.org/10.1177/10775595211034430
12. Finkelhor D, Ormrod RK, Turner HA. Poly-victimization: a neglected component in child victimization. *Child Abuse Negl*. 2007;31(1):7-26. https://doi.org/10.1016/j.chiabu.2006.06.008
13. Adverse childhood experiences resources. Centers for Disease Control and Prevention. Accessed March 1, 2024. https://www.cdc.gov/violenceprevention/aces/resources.html
14. Clithero A, Albright D, Bissell E, et al. Addressing interpersonal violence as a health policy question using interprofessional community educators. *MedEdPORTAL*. 2016;12:10516. https://doi.org/10.15766/mep_2374-8265.10516
15. Pais S, Laskey A, Graves A, Griffith D, Fife R, Litzelman D. Family violence interactive experience. *MedEdPORTAL*. 2009;5:7890. https://doi.org/10.15766/mep_2374-8265.7890
16. Song AY, Poythress EL, Bocchini CE, Kass JS. Reorienting orientation: introducing the social determinants of health to first-year medical students. *MedEdPORTAL*. 2018;14:10752. https://doi.org/10.15766/mep_2374-8265.10752
17. Johnson SB, Riley AW, Granger DA, Riis J. The science of early life toxic stress for pediatric practice and advocacy. *Pediatrics*. 2013;131(2):319-327. https://doi.org/10.1542/peds.2012-0469
18. Garner A, Yogman M; Committee on Psychosocial Aspects of Child and Family Health, Section on Developmental and Behavioral Pediatrics, Council on Early Childhood. Preventing childhood toxic stress: partnering with families and communities to promote relational health. *Pediatrics*. 2021;148(2): e2021052582. https://doi.org/10.1542/peds.2021-052582
19. Koponen J, Pyörälä E, Isotalus P. Comparing three experiential learning methods and their effect on medical students' attitudes to learning communication skills. *Med Teach*. 2012;34(3): e198-e207. https://doi.org/10.3109/0142159X.2012.642828
20. Bosse HM, Schultz JH, Nickel M, et al. The effect of using standardized patients or peer role play on ratings of undergraduate communication training: a randomized controlled trial. *Patient Educ Couns*. 2012:87(3):300-306. https://doi.org/10.1016/j.pec.2011.10.007
21. Nestel D, Tierney T. Role-play for medical students learning about communication: guidelines for maximising benefits. *BMC Med Educ*. 2007;7:3. https://doi.org/10.1186/1472-6920-7-3
22. Kolb DA, Fry R. Toward an applied theory of experiential learning. In: Cooper CL, ed. *Theories of Group Process*. Wiley; 1975: 33-57.
23. Harder N, Lemoine J, Chernomas W, Osachuk T. Developing a trauma-informed psychologically safe debriefing framework for emotionally stressful simulation events. *Clin Simul Nurs*. 2021;51: 1-9. https://doi.org/10.1016/j.ecns.2020.11.007
24. Putnam AL. Mnemonics in education: current research and applications. *Transl Issues Psychol Sci*. 2015;1(2):130-139. https://doi.org/10.1037/tps0000023
25. Khachadoorian-Elia HR, Bush LW, Rider EA, et al. When I say… lived curriculum. *Med Educ.* 2023;57(7):609-611. https://doi.org/10.1111/medu.15084
26. Simpkin AL, Schwartzstein RM. Tolerating uncertainty—the next medical revolution? *N Engl J Med*. 2016;375(18):1713-1715. https://doi.org/10.1056/NEJMp1606402
27. Roze des Ordons AL, Ellaway RH, Eppich W. The many spaces of psychological safety in health professions education. *Med Educ*. 2022;56(11):1060-1063. https://doi.org/10.1111/medu.14919
28. Howard GS, Ralph KM, Gulanick NA, Maxwell SE, Nance DW, Gerber SK. Internal invalidity in pretest-posttest self-report evaluations and a re-evaluation of retrospective pretests. *Appl Psychol Meas*. 1979;3(1):1-23. https://doi.org/10.1177/014662167900300101
29. US Preventive Services Task Force. Screening for intimate partner violence, elder abuse, and abuse of vulnerable adults: US Preventive Services Task Force final recommendation statement.

Copyright © 2024 Franchek-Roa et al. This is an open-access publication distributed under the terms of the Creative Commons Attribution license.

JAMA. 2018;320(16):1678-1687. https://doi.org/10.1001/jama.2018.14741

30. Miller E, McCaw B, Humphreys BL, Mitchell C. Integrating intimate partner violence assessment and intervention into healthcare in the United States: a systems approach. *J Womens Health (Larchmt)*. 2015;24(1):92-99. https://doi.org/10.1089/jwh.2014.4870
31. Mukhalalati BA, Taylor A. Adult learning theories in context: a quick guide for healthcare professional educators. *J Med Educ Curric Dev*. 2019;6:2382120519840332. https://doi.org/10.1177/2382120519840332
32. Boissy AR, Ford PJ. A touch of MS: therapeutic mislabeling. *Neurology*. 2012;78(24):1981-1985. https://doi.org/10.1212/WNL.0b013e318259e0ec
33. Weleff J, Potter D. Key updates to understanding roles of childhood trauma in overall health. *AMA J Ethics.* 2023;25(2): E116-E122. https://doi.org/10.1001/amajethics.2023.116
34. Keeshin B, Byrne K, Thorn B, Shepard L. Screening for trauma in pediatric primary care. *Curr Psychiatry Rep.* 2020;22(11):60. https://doi.org/10.1007/s11920-020-01183-y
35. Lewis-O'Connor A, Warren A, Lee JV, et al. The state of the science on trauma inquiry. *Womens Health (Lond)*. 2019;15: 1745506519861234. https://doi.org/10.1177/1745506519861234

Received: August 31, 2023
Accepted: December 18, 2023
Published: April 23, 2024

Copyright © 2024 Franchek-Roa et al. This is an open-access publication distributed under the terms of the Creative Commons Attribution license.
